# Supplementary material for: Longitudinal analysis reveals that delayed bystander CD8+ T cell activation and early immune pathology distinguish severe COVID-19 from mild disease
Source: Immunity. 2021 Jun 8;54(6):1257–1275.e8. doi: 10.1016/j.immuni.2021.05.010 (PMC8125900; doi:10.1016/j.immuni.2021.05.010)
Supplement: Document S1. Figures S1–S7, Tables S1 and S2, Data S1–S4, and the list of CITIID-NIHR COVID Bioresource Collaboration members, subdivided by author contribution, and their affiliations [file mmc1.pdf]

Immunity, Volume *54*

Supplemental information

Longitudinal analysis reveals that delayed bystander CD8+ T cell activation and early immune pathology distinguish severe COVID-19 from mild disease

Laura Bergamaschi, Federica Mescia, Lorinda Turner, Aimee L. Hanson, Prasanti Kotagiri, Benjamin J. Dunmore, Hélène Ruffieux, Aloka De Sa, Oisín Huhn, Michael D. Morgan, Pehuén Pereyra Gerber, Mark R. Wills, Stephen Baker, Fernando J. Calero-Nieto, Rainer Doffinger, Gordon Dougan, Anne Elmer, Ian G. Goodfellow, Ravindra K. Gupta, Myra Hosmillo, Kelvin Hunter, Nathalie Kingston, Paul J. Lehner, Nicholas J. Matheson, Jeremy K. Nicholson, Anna M. Petrunkina, Sylvia Richardson, Caroline Saunders, James E.D. Thaventhiran, Erik J.M. Toonen, Michael P. Weekes, Cambridge Institute of Therapeutic Immunology and Infectious Disease-National Institute of Health Research (CITIID-NIHR) COVID BioResource Collaboration, Berthold Göttgens, Mark Toshner, Christoph Hess, John R. Bradley, Paul A. Lyons, and Kenneth G.C. Smith

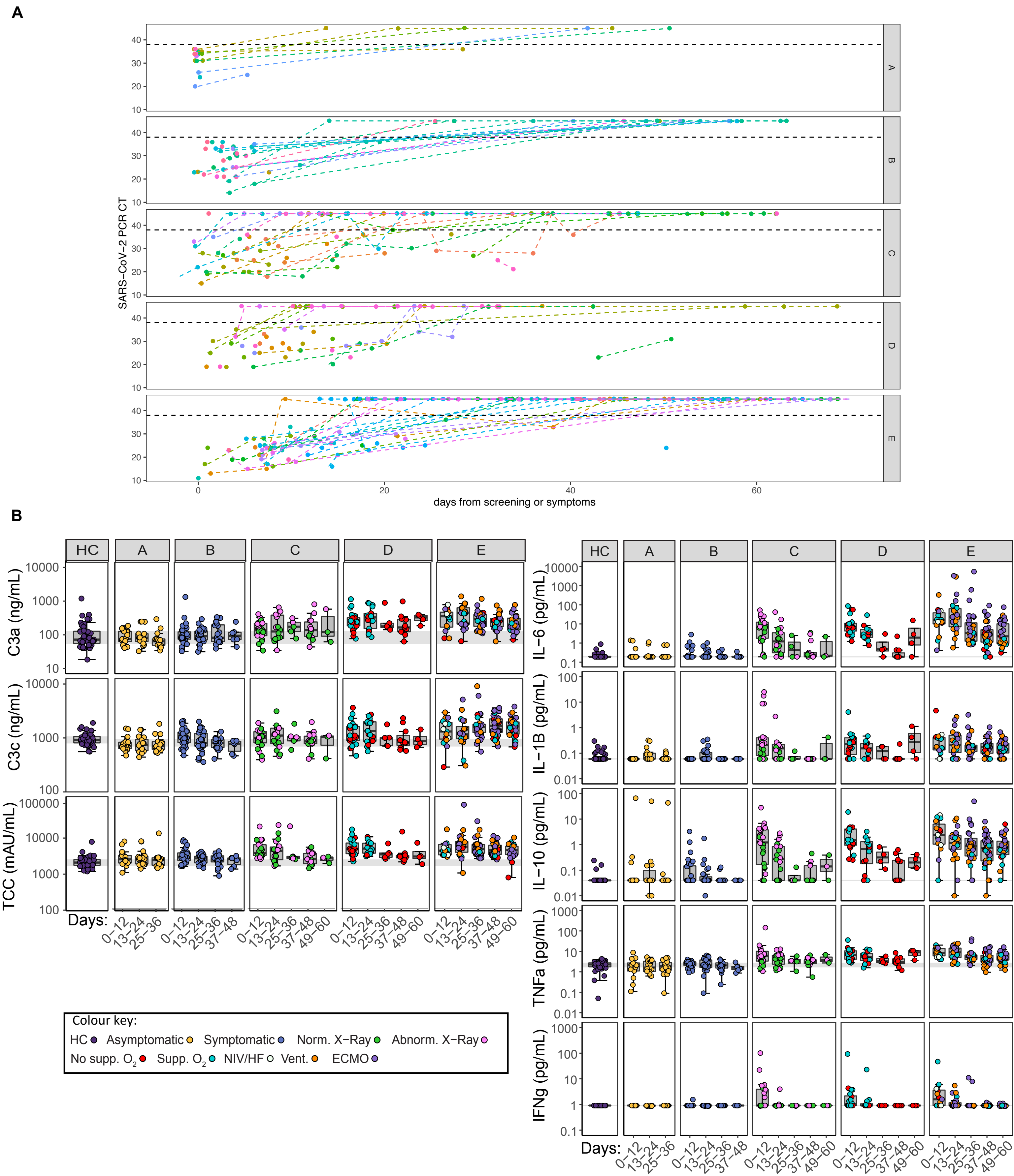

A
SARS−CoV−2 PCR CT
days from screening or symptoms
B
HC
A
B
C
D
E
C3a (ng/mL)
C3c (ng/mL)
TCC (mAU/mL)
IL−6 (pg/mL)
IL−1B (pg/mL)
IL−10 (pg/mL)
TNFa (pg/mL)
IFNg (pg/mL)
Days:
0−12
13−24
25−36
37−48
49−60
Colour key:
HC Asymptomatic Symptomatic Norm. X−Ray Abnorm. X−Ray
No supp. O2 Supp. O2 NIV/HF Vent. ECMO

Figure S1: Inflammation and viral load changes over time in COVID-19 patients, related to Figure 1

A) SARS-CoV-2 PCR CT values over time across patient severity groups. CT values ≤ 38 (dashed horizontal line) were reported as a positive result. A discretional CT number of 45 was assigned to samples with no detectable SARS-CoV-2 RNA. Each colour corresponds to a different subject. Repeat measures for a single participant are linked by dashed line. **B)** Boxplots showing complement components and cytokine concentrations for samples collected within 12-day time bins. Grey band indicates the interquartile range of the corresponding measure in HCs. Points are coloured based on asymptomatic or symptomatic classification for categories A and B respectively, normal or abnormal chest radiology (group C), and type of respiratory support at sampling (group D and E), as colour key provided. NIV/HF, non-invasive ventilation/high-flow oxygen; vent, mechanical ventilation; ECMO, extracorporeal membrane oxygenation.

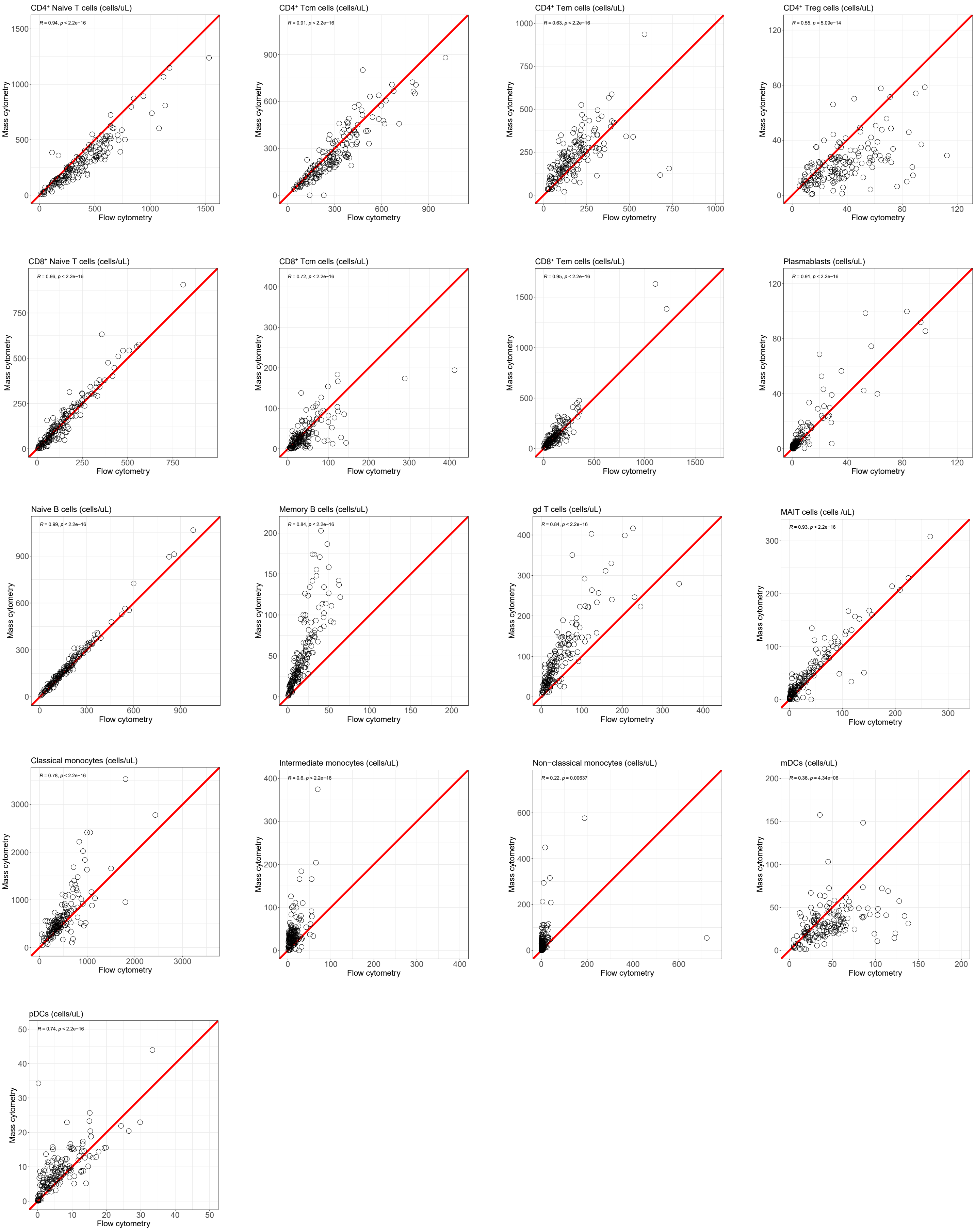
CD4+ Naive T cells (cells/uL)
R = 0.94, p < 2.2e−16
CD4+ Tcm cells (cells/uL)
R = 0.91, p < 2.2e−16
CD4+ Tem cells (cells/uL)
R = 0.63, p < 2.2e−16
CD4+ Treg cells (cells/uL)
R = 0.55, p = 5.09e−14
CD8+ Naive T cells (cells/uL)
R = 0.96, p < 2.2e−16
CD8+ Tcm cells (cells/uL)
R = 0.72, p < 2.2e−16
CD8+ Tem cells (cells/uL)
R = 0.95, p < 2.2e−16
Plasmablasts (cells/uL)
R = 0.91, p < 2.2e−16
Naive B cells (cells/uL)
R = 0.99, p < 2.2e−16
Memory B cells (cells/uL)
R = 0.84, p < 2.2e−16
gd T cells (cells/uL)
R = 0.84, p < 2.2e−16
MAIT cells (cells /uL)
R = 0.93, p < 2.2e−16
Classical monocytes (cells/uL)
R = 0.78, p < 2.2e−16
Intermediate monocytes (cells/uL)
R = 0.6, p < 2.2e−16
Non−classical monocytes (cells/uL)
R = 0.22, p = 0.00637
mDCs (cells/uL)
R = 0.36, p = 4.34e−06
pDCs (cells/uL)
R = 0.74, p < 2.2e−16
Mass cytometry
Flow cytometry

Figure S2: Comparison of absolute cell counts derived by flow and mass cytometry, related to STAR methods. Scatter plots showing the correlation between cell populations quantified by both flow and mass cytometry (cells/uL). Pearson correlation R value and p-values of correlation test are reported for each comparison.

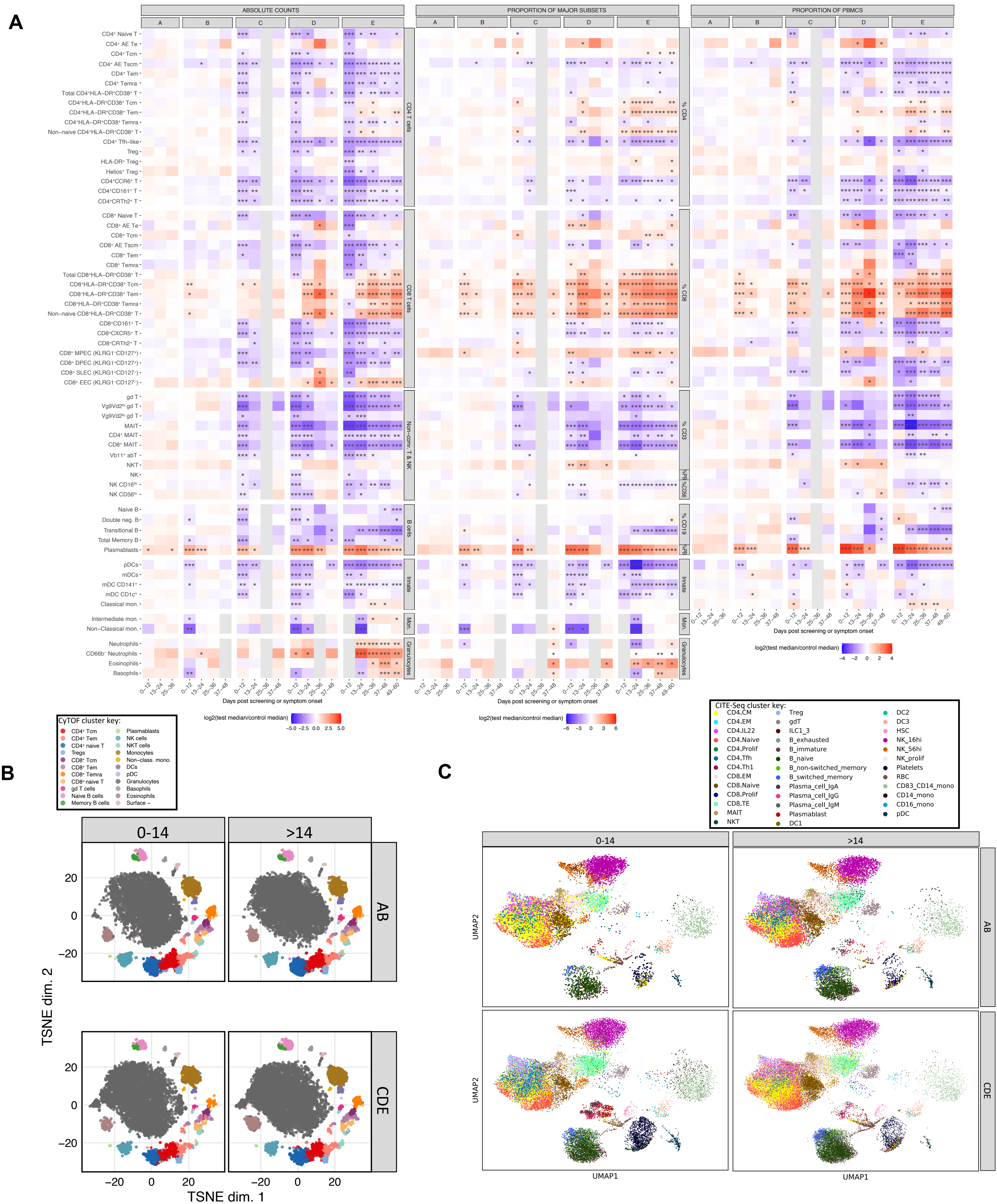
A
ABSOLUTE COUNTS
PROPORTION OF MAJOR SUBSETS
PROPORTION OF PBMCS
A
B
C
D
E
CD4 T cells
CD8 T cells
Non−conv. T & NK
B cells
Innate
Mon.
Granulocytes
% CD4
% CD8
% CD3
% CD19
Days post screening or symptom onset
log2(test median/control median)
CyTOF cluster key:
CD4⁺ Tcm
CD4⁺ Tem
CD4⁺ naive T
Tregs
CD8⁺ Tcm
CD8⁺ Tem
CD8⁺ Temra
CD8⁺ naive T
gd T cells
Naive B cells
Memory B cells
Plasmablasts
NK cells
NKT cells
Monocytes
Non−class. mono.
DCs
pDC
Granulocytes
Basophils
Eosinophils
Surface −
B
0-14
>14
AB
CDE
TSNE dim. 1
TSNE dim. 2
C
CITE-Seq cluster key:
CD4.CM
CD4.EM
CD4.IL22
CD4.Naive
CD4.Prolif
CD4.Tfh
CD4.Th1
CD8.EM
CD8.Naive
CD8.Prolif
CD8.TE
MAIT
NKT
Treg
gdT
ILC1_3
B_exhausted
B_immature
B_naive
B_non-switched_memory
B_switched_memory
Plasma_cell_IgA
Plasma_cell_IgG
Plasma_cell_IgM
Plasmablast
DC1
DC2
DC3
HSC
NK_16hi
NK_56hi
NK_prolif
Platelets
RBC
CD83_CD14_mono
CD14_mono
CD16_mono
pDC
UMAP1
UMAP2

Figure S3: Cellular changes observed over time in COVID-19 patients including absolute cell counts and proportion, related to Figure 2

A) Heatmap showing the log2 fold change in median absolute cell count between COVID-19 patients and healthy controls, within severity categories and across 12-day time bins post screening (group A) or symptom onset (groups B-E). Missing data are shown in grey. Wilcoxon test FDR adjusted p-values: *<0.05, **<0.005, *** <0.0005. **B)** tSNE **and C)** UMAP plots comparing groups A and B to groups C, D and E within 14 or later (>14) days post screening (group A) or symptom onset (groups B-E) for **B)** CyTOF and **C)** CITE-Seq dataset. Cell clusters are coloured as key provide.

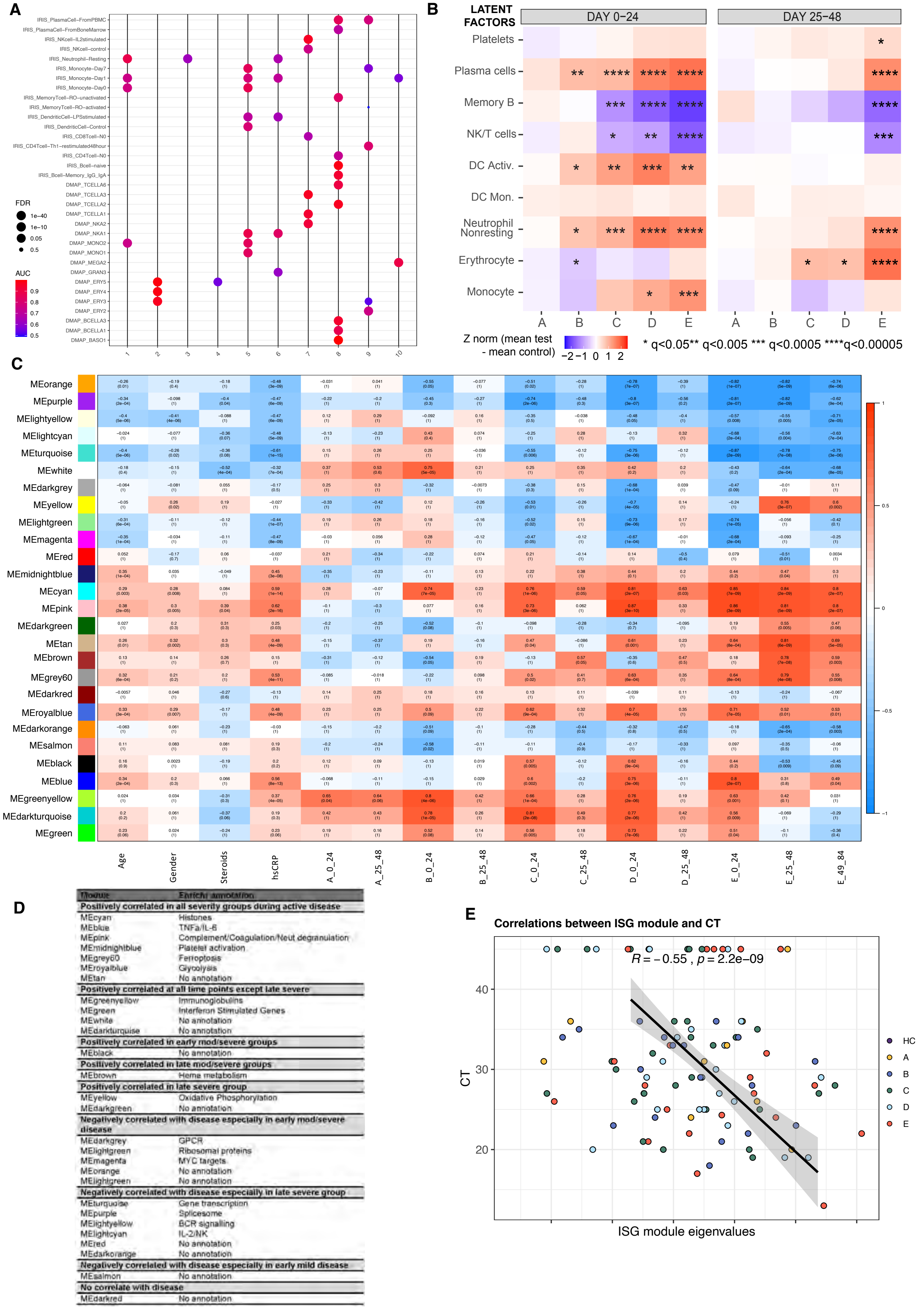

Module	Enrichr annotation
**Positively correlated in all severity groups during active disease**	
MEcyan	Histones
MEblue	TNFa/IL-6
MEpink	Complement/Coagulation/Neut degranulation
MEmidnightblue	Platelet activation
MEgrey60	Ferroptosis
MEroyalblue	Glycolysis
MEtan	No annotation
**Positively correlated at all time points except late severe**	
MEgreenyellow	Immunoglobulins
MEgreen	Interferon Stimulated Genes
MEwhite	No annotation
MEdarkturquoise	No annotation
**Positively correlated in early mod/severe groups**	
MEblack	No annotation
**Positively correlated in late mod/severe groups**	
MEbrown	Heme metabolism
**Positively correlated in late severe group**	
MEyellow	Oxidative Phosphorylation
MEdarkgreen	No annotation
**Negatively correlated with disease especially in early mod/severe disease**	
MEdarkgrey	GPCR
MElightgreen	Ribosomal proteins
MEmagenta	MYC targets
MEorange	No annotation
MElightgreen	No annotation
**Negatively correlated with disease especially in late severe group**	
MEturquoise	Gene transcription
MEpurple	Splicesome
MElightyellow	BCR signalling
MElightcyan	IL-2/NK
MEred	No annotation
MEdarkorange	No annotation
**Negatively correlated with disease especially in early mild disease**	
MEsalmon	No annotation
**No correlate with disease**	
MEdarkred	No annotation

Figure S4: Cell subset deconvolution of whole blood transcriptome and transcriptional signatures observed over time in COVID-19, related to Figure 3. A) Annotation of Latent Factors used to perform the cell subset deconvolution shown in **B). B)** Cell subset deconvolution performed using PLIER, leveraging off prior knowledge of cell specific pathways. COVID-19 cases split by severity categories and 24-day time bins. Latent factor expression compared with HCs, FDR adjusted p-value: *<0.05, **<0.005, ***<0.0005. **C)** Heatmap illustrating the correlation among whole blood co-expression gene modules derived from WGCNA (coloured blocks, y axis) and age, gender, steroid treatment, CRP concentrations, and the comparison between HCs and COVID-19 cases split by severity in 24 days bins (x axis). Pearson correlation and FDR corrected p-values are shown for each comparison. The full list of genes in each module can be found in **Table S3**. **D)** Annotation by EnrichR of modules that correlate with disease and/or severity. **E)** Correlation between SARS-CoV-2 PCR CT values and Interferon Signature Genes (IGS) module eigenvalues (samples, n=248).

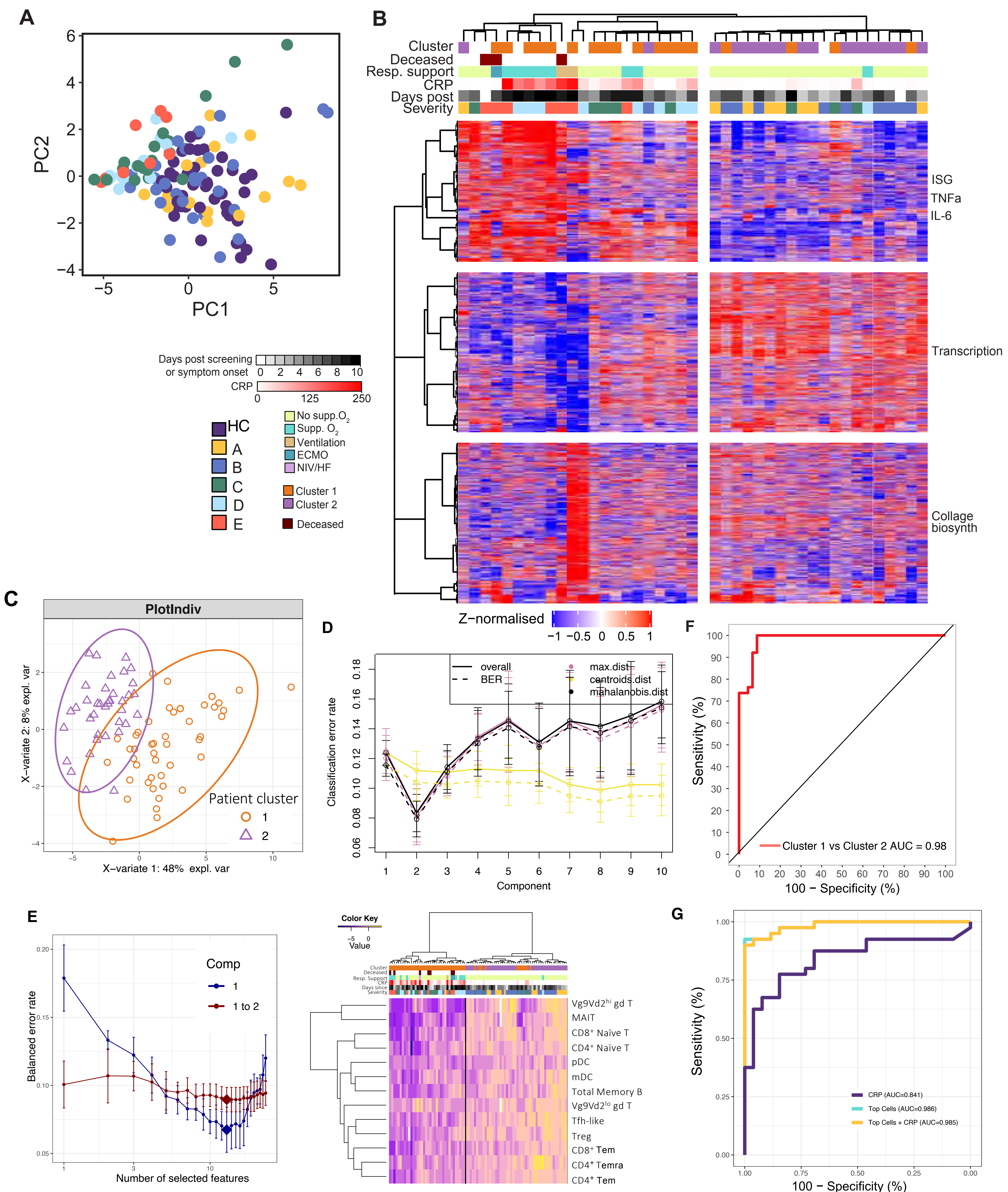

A
PC2
PC1
Days post screening
or symptom onset
CRP
HC
A
B
C
D
E
No supp.O2
Supp. O2
Ventilation
ECMO
NIV/HF
Cluster 1
Cluster 2
Deceased
B
Cluster
Deceased
Resp. support
CRP
Days post
Severity
ISG
TNFa
IL-6
Transcription
Collage
biosynth
Z−normalised
C
PlotIndiv
X−variate 2: 8% expl. var
X−variate 1: 48% expl. var
Patient cluster
D
Classification error rate
Component
overall
BER
max.dist
centroids.dist
mahalanobis.dist
F
Sensitivity (%)
100 − Specificity (%)
Cluster 1 vs Cluster 2 AUC = 0.98
E
Balanced error rate
Number of selected features
Comp
1
1 to 2
Color Key
Value
Vg9Vd2hi gd T
MAIT
CD8+ Naïve T
CD4+ Naïve T
pDC
mDC
Total Memory B
Vg9Vd2lo gd T
Tfh-like
Treg
CD8+ Tem
CD4+ Temra
CD4+ Tem
G
Sensitivity (%)
100 − Specificity (%)
CRP (AUC=0.841)
Top Cells (AUC=0.986)
Top Cells + CRP (AUC=0.985)

Figure S5: Multivariate analysis of immune-cell populations in early disease, related to Figure 4

A) Principal component analysis of peripheral blood absolute cell counts for 24 key cell subsets from HCs and COVID-19 cases, for samples taken ≤10 days from screening (group A) or symptom onset (groups B-E). Points are coloured according to severity category. **B)** K-means clustering of 18357 whole blood transcripts from COVID-19 samples taken ≤10 days from screening or symptom onset. Gene clusters are annotated for enriched signatures, samples are annotated according to corresponding cluster membership in **Figure 4A** where possible. **C)** Variable selection by sPLS-DA showing discrimination of patient clusters 1 (orange, n=46) and 2 (purple, n=38) derived in **Figure 4A**. **D)** Associated classification error rate of the predictive model across 10 iterations of 5-fold cross validation for components 1-10. **E)** Feature selection on components 1 and 2, determining 13 cell subsets as key contributors to cluster discrimination with minimal error. Unsupervised clustering of 13 selected cell types (normalised to the median of HCs), with original sample clusters and patient characteristics indicated. Error bars in **D)** and **E)** indicate standard deviations (SD). **F) and G)** AUROC curves showing sensitivity of cluster group prediction at varying specificity thresholds, based on **F)** absolute counts of 13 selected cell types, or **G)** CRP alone and combined with the absolute counts of 13 selected cell types.

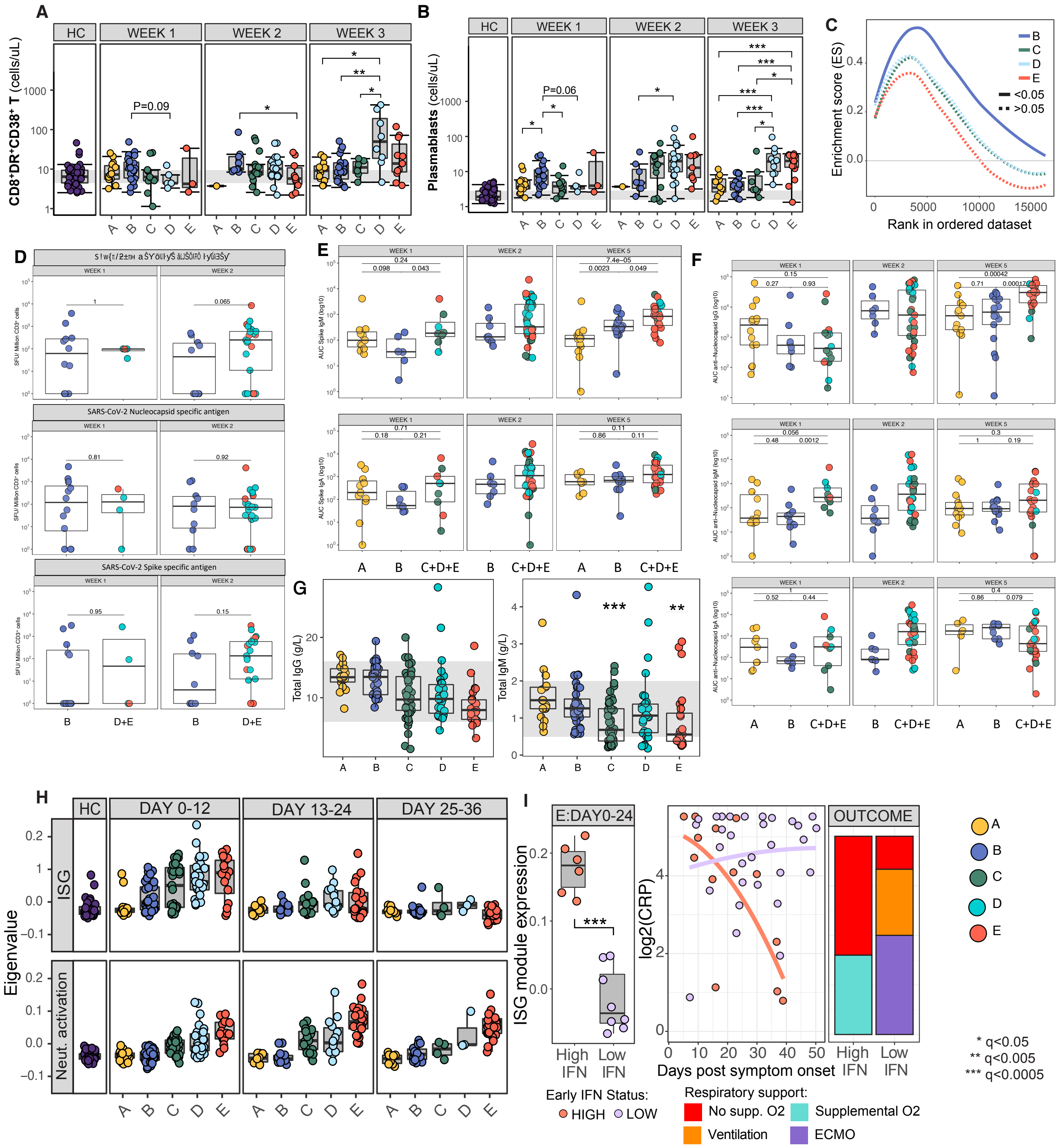
A
HC
WEEK 1
WEEK 2
WEEK 3
CD8⁺DR⁺CD38⁺ T (cells/uL)
P=0.09
A B C D E
B
Plasmablasts (cells/uL)
P=0.06
C
Enrichment score (ES)
Rank in ordered dataset
<0.05
>0.05
D
SARS-CoV-2 Nucleocapsid specific antigen
SARS-CoV-2 Spike specific antigen
SFU/ Million CD3⁺ cells
B
D+E
E
WEEK 5
AUC Spike IgM (log10)
AUC Spike IgA (log10)
A
B
C+D+E
F
AUC anti-Nucleocapsid IgG (log10)
AUC anti-Nucleocapsid IgM (log10)
AUC anti-Nucleocapsid IgA (log10)
G
Total IgG (g/L)
Total IgM (g/L)
H
DAY 0-12
DAY 13-24
DAY 25-36
ISG
Neut. activation
Eigenvalue
I
E:DAY0-24
ISG module expression
High IFN
Low IFN
log2(CRP)
Days post symptom onset
OUTCOME
Early IFN Status:
HIGH
LOW
Respiratory support:
No supp. O2
Supplemental O2
Ventilation
ECMO
* q<0.05
** q<0.005
*** q<0.0005

Figure S6: Early immune changes associated with mild disease and outcome extended data, related to Figure 5

A and B): Boxplots showing **A)** non-naive $CD8^+HLA\text{-}DR^+CD38^+$ T cell and **B)** plasmablast absolute cell counts (cells/uL) across severity categories at weeks 1-3 post screening (group A) or symptom onset (groups B-E). Wilcoxon test FDR adjusted p-value: *<0.05, **<0.005, ***<0.0005. Grey bar represents the interquartile range of the same cell populations in HCs. **C)** Enrichment score for $CD8^+$ T-cell activation signature as determined by GSEA in groups B-E for samples taken <24 days from symptom onset. **D)** Spot forming unit (SFU) numbers of $CD3^+$ T cells secreting IFN-γ in response to membrane, nucleocapsid and spike SARS-CoV-2 antigen stimulations, in patient samples from groups B (n=22), and D and E combined (n=25), one or two weeks post symptom onset. Kruskal-Wallis test p-values. **E)** Area under the curve for SARS-CoV-2 spike-specific IgM and IgA titres at 1, 2 and 5 weeks post screening (group A) or symptom onset (groups B-E). Groups C, D and E are combined for increased statistical power. Wilcoxon test p-values. **F)** Area under the curve for SARS-CoV-2 nucleocapsid-specific IgG, IgM and IgA titres. Groups, timepoints and p-values as in **E)**. **G)** Total IgG and IgM concentrations across severity groups, within 3 weeks post screening (group A) or symptom onset (groups B-E). Grey band corresponds to 5-95th centile ranges based on UK Caucasian population, as published in the Protein Reference Unit Handbook (9th Edition). P-values from comparisons with the reference range using Pearson's chi-square test, annotated as in **A)**. **H)** Boxplots capturing expression of interferon stimulated genes (ISG) and neutrophil activation-related transcriptomic eigenvalues across disease severity and time(samples, n=248). **I)** Stratification of group E samples taken <24 days post symptom onset into high and low expression of ISG, with persisting and resolving CRP concentrations and final respiratory status reported within 12 weeks shown by bar charts.

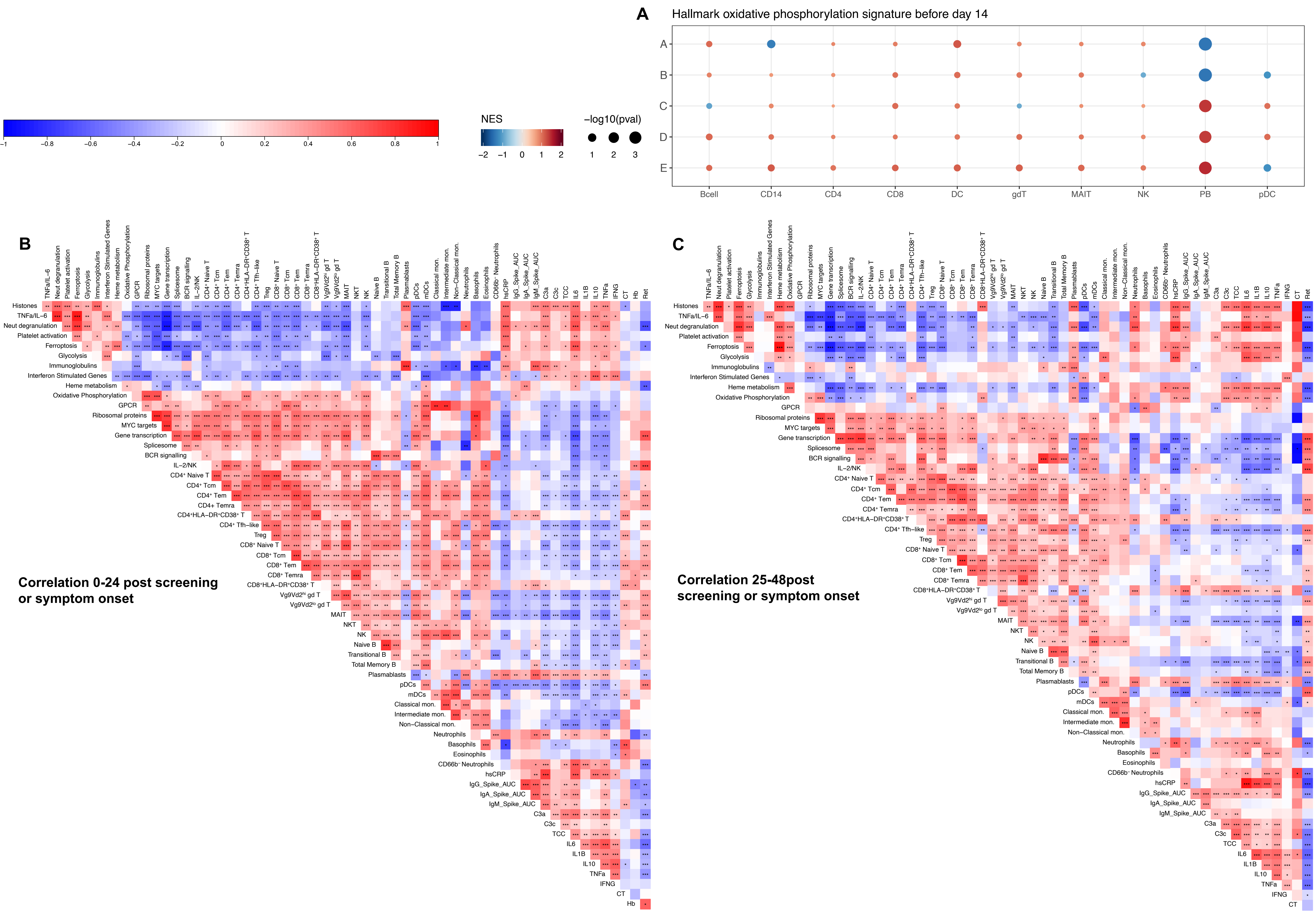
A
Hallmark oxidative phosphorylation signature before day 14
NES
−log10(pval)
Bcell
CD14
CD4
CD8
DC
gdT
MAIT
NK
PB
pDC
B
Correlation 0-24 post screening or symptom onset
C
Correlation 25-48post screening or symptom onset
Histones
TNFa/IL−6
Neut degranulation
Platelet activation
Ferroptosis
Glycolysis
Immunoglobulins
Interferon Stimulated Genes
Heme metabolism
Oxidative Phosphorylation
GPCR
Ribosomal proteins
MYC targets
Gene transcription
Splicesome
BCR signalling
IL−2/NK
CD4+ Naive T
CD4+ Tcm
CD4+ Tem
CD4+ Temra
CD4+HLA−DR+CD38+ T
CD4+ Tfh−like
Treg
CD8+ Naive T
CD8+ Tcm
CD8+ Tem
CD8+ Temra
CD8+HLA−DR+CD38+ T
Vg9Vd2hi gd T
Vg9Vd2lo gd T
MAIT
NKT
NK
Naive B
Transitional B
Total Memory B
Plasmablasts
pDCs
mDCs
Classical mon.
Intermediate mon.
Non−Classical mon.
Neutrophils
Basophils
Eosinophils
CD66b− Neutrophils
hsCRP
IgG_Spike_AUC
IgA_Spike_AUC
IgM_Spike_AUC
C3a
C3c
TCC
IL6
IL1B
IL10
TNFa
IFNG
CT
Hb
Ret

Figure S7: Transcriptional changes in prolonged disease were independent of cell subset composition, related to Figures 7

A) GSEA assessing HALLMARK oxidative phosphorylation geneset in different cell type identify by CITE-Seq against HCs in COVID-19 patients collected within 14 days post screening (group A) or symptom onset (groups B-E). FDR adjusted p-value is shown by circle diameter, with colour representing normalised enrichment score. **B) and C):** Heatmap showing the correlation between gene expression eigenvalues derived from whole blood RNA-Seq, absolute cell counts and inflammatory characteristics in COVID-19 patients collected within **B)** the first 24 days, or **D)** between 25-48 days post screening (group A) or symptom onset (groups B-E). Pearson correlation p-values: *p<0.05, **p<0.01 and ***p<0.001.

Table S1: Clinical features of study participants, stratified by group A-E, related to Figure 1

	A	B	C	D	E
**n**	18	40	46	37	60
**Gender (% male)**	22.2%	22.5%	54.3%	64.9%	75.0%
**Age (years, mean (SD))**	32.9 (12.7)	36.0 (11.8)	58.0 (16.9)	64.4 (15.1)	57.0 (14.9)
**Days from COVID-19 symptoms to enrollment (days, mean (SD))**	NA	6.5 (2.9)	11.4 (6.7)	10.6 (8.1)	24.6 (14.3)
**COVID-19 chest radiology**	NA	NA	50.0%	89.2%	100%
**Non-COVID19 admissions**	NA	NA	30.2%	8.1%	6.7%
**Haemoglobin (g/L, mean (SD))**	NA	NA	124.8 (16.0)	121.6 (18.0)	95.2 (16.8)
**Serum creatinine (µmol/L, mean (SD))**	NA	NA	82.9 (40.1)	117.5 (154.7)	103.5 (129.3)
**Serum albumin (g/L, mean (SD))**	NA	NA	32.4 (7.1)	28.0 (6.3)	24.4 (7.2)
**LOS (days, median (IQR))**	NA	NA	4 (1.25-10)	10 (6-16)	44 (33.7-63.2)
**Admitted to ITU**	NA	NA	0%	13.5%	90.0%
**Deceased in hospital**	NA	NA	2.2%	0.0%	30.0%
**Hypertension**	NA	NA	47.8%	43.2%	48.3%
**CAD**	NA	NA	8.7%	24.3%	16.7%
**Other heart condition**	NA	NA	10.9%	18.9%	13.3%
**Diabetes mellitus**	NA	NA	26.1%	29.7%	43.3%
**CKD**	NA	NA	8.7%	16.2%	8.3%
**PVD**	NA	NA	6.5%	8.1%	8.3%
**CVA/TIA**	NA	NA	10.9%	2.7%	6.7%
**COPD**	NA	NA	6.5%	18.9%	5.0%
**Asthma**	NA	NA	21.7%	10.8%	10.0%
**Other lung disease**	NA	NA	10.9%	16.2%	10.0%
**Cancer**	NA	NA	4.4%	5.4%	1.7%
**Haematological cancer**	NA	NA	2.2%	5.4%	0.0%
**Corticosteroids**	NA	NA	19.6%	10.8%	10.0%
**Immunosuppressive treatment**	NA	NA	17.4%	16.2%	5.0%

SD is standard deviation, and IQR is interquartile range.
COVID-19 chest radiology: chest X-ray/ CT scan showed changes compatible with COVID-19, as opposed to normal findings or lung changes diagnostic of other conditions.
Non-COVID19 admissions: cases where COVID-19 was diagnosed during the hospital stay in patients initially admitted to hospital for reasons unrelated to COVID-19
Haemoglobin, serum albumin and serum creatinine: results from routine lab tests on the day of study enrollment, or closest result up to 2 days before. The included test results are available for at least for 75% of each severity group.
LOS: length of hospital stay (days from hospital admission to discharge, transfer or death in hospital)
Hypertension: history of hypertension, defined as blood pressure ≥140/80 on multiple occasions, or on treatment with any medication explicitly employed to reduce blood pressure
CAD: history of coronary artery disease, defined as myocardial infarction, angina, coronary artery stenting or coronary artery bypass grafting
Other heart condition: history of any other chronic cardiac disease (not CAD/hypertension), e.g. heart failure, congenital heart disease, cardiomyopathy, rheumatic heart disease
CKD: history of chronic kidney disease, defined as any of estimated glomerular filtration rate < 60 mL/min/1.73m^2, dialysis or kidney transplant
PVD: history of peripheral vascular disease, defined as intermittent claudication or past bypass for chronic arterial insufficiency, history of gangrene or acute arterial insufficiency, or thoracic/abdominal aneurysm (≥6 cm)
CVA/TIA: history of a cerebrovascular accident or transient ischemic attacks
COPD: history of chronic obstructive pulmonary disease
Other lung disease: history of other chronic pulmonary disease (non asthma/COPD), e.g. cystic fibrosis, bronchiectasis, interstitial lung disease
Cancer: current solid organ malignancy (active or in the last 5 years), except non-melanoma skin cancers
Corticosteroids: history of treatment with systemic corticosteroids in the 14 days prior to hospital admission/presentation
Immunosuppressive treatment: history of treatment with immunosuppressants (excluding corticosteroids) in the 14 days prior to hospital admission/presentation, or chemotherapy/biologic drugs in the previous 6 months

Table S2: patients excluded because of extreme confounding comorbidities, related to STAR Methods

Study ID	Patient profile
CV0266	Metastatic lung adenocarcinoma on immunotherapy and chemotherapy, presentation with new onset heart failure and pulmonary oedema, borderline positive COVID-19 PCR on nasopharyngeal swab, but no other clinical features of COVID-19
CV0258	Prolonged hospital admission for new diagnosis of acute myeloid leukemia with suspected leukemic lung infiltration and fungal chest infection, commenced on chemotherapy. COVID-19 PCR negative at hospital admission and subsequently positive.
CV0143	Mild COVID-19 symptoms, admitted for extensive necrotizing fasciitis/mediastinitis, treated with surgical debridement and complicated by massive haemorrhage. Enrolled in the study in the ITU after surgery.
CV0192	Emergency splenectomy following trauma. Fever and pneumonia in the post-op, with radiology in keeping with aspiration. Borderline positive COVID-19 PCR on nasopharyngeal swab.
CV0033	Lymphoma on palliative chemotherapy, initially admitted for interstitial pneumonia and neutropenia and treated as Pneumocystis pneumonia. COVID-19 PCR initially negative 4x and subsequently positive after 2 weeks.
CV0313	End-stage alcoholic cirrhosis with variceal bleeding and hepatic encephalopathy, admitted for transjugular intrahepatic portosystemic shunt procedure. Positive COVID-19 PCR on nasopharyngeal swab, initially with normal chest X-ray. Subsequently developed severe chest infection with bronchoalveolar lavage positive for Gram negative organisms and negative for COVID-19 PCR.

Data S1: Time course plots for hospitalized patients (groups C/D/E), related to STAR Methods

Swimmer plots for hospitalized cases in groups **A)** C, **B)** D, and **C)** E. Arrows indicate days since start of COVID-19 symptoms (or since positive SARS-CoV-2 PCR test, if asymptomatic). Black boxes indicate the duration of hospital stay whilst enrolled in the study. If patients were transferred from other hospitals (as indicated by the triangle symbol), information relative to the time before transfer to one of the study hospitals may be missing. Research blood collection timepoints are indicated by open circle, and time of death by red cross for deceased patients. Degree of respiratory support is indicated by colour gradient as per the key provided.

Legend

no supplemental oxygen
supplemental oxygen
NIV/HF
invasive ventilation
ECMO
research bloods
* asymptomatic
admitted to other hospital
transferred to other hospital
X deceased

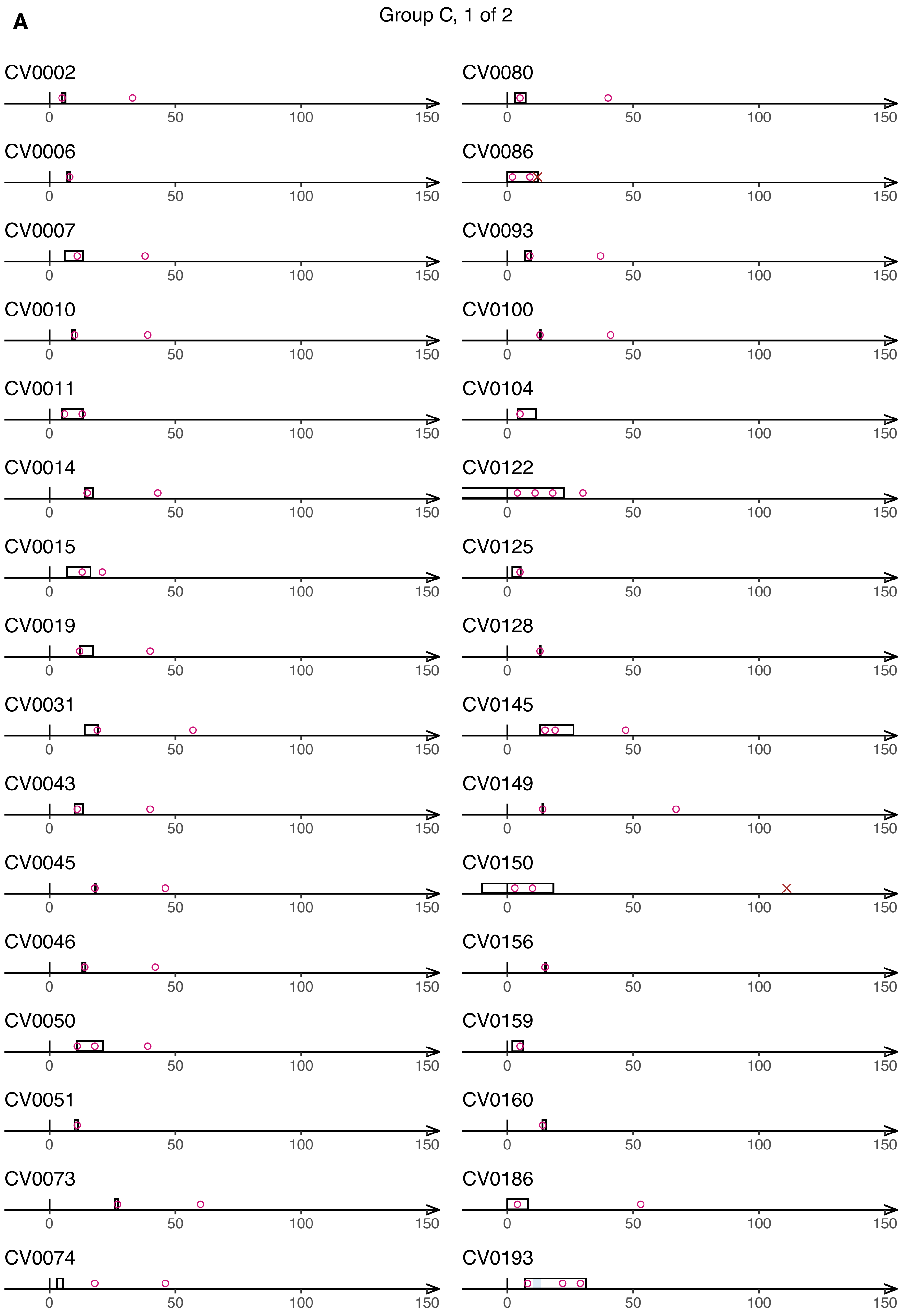
A
Group C, 1 of 2
CV0002
CV0006
CV0007
CV0010
CV0011
CV0014
CV0015
CV0019
CV0031
CV0043
CV0045
CV0046
CV0050
CV0051
CV0073
CV0074
CV0080
CV0086
CV0093
CV0100
CV0104
CV0122
CV0125
CV0128
CV0145
CV0149
CV0150
CV0156
CV0159
CV0160
CV0186
CV0193
0 50 100 150

Group C, 2 of 2

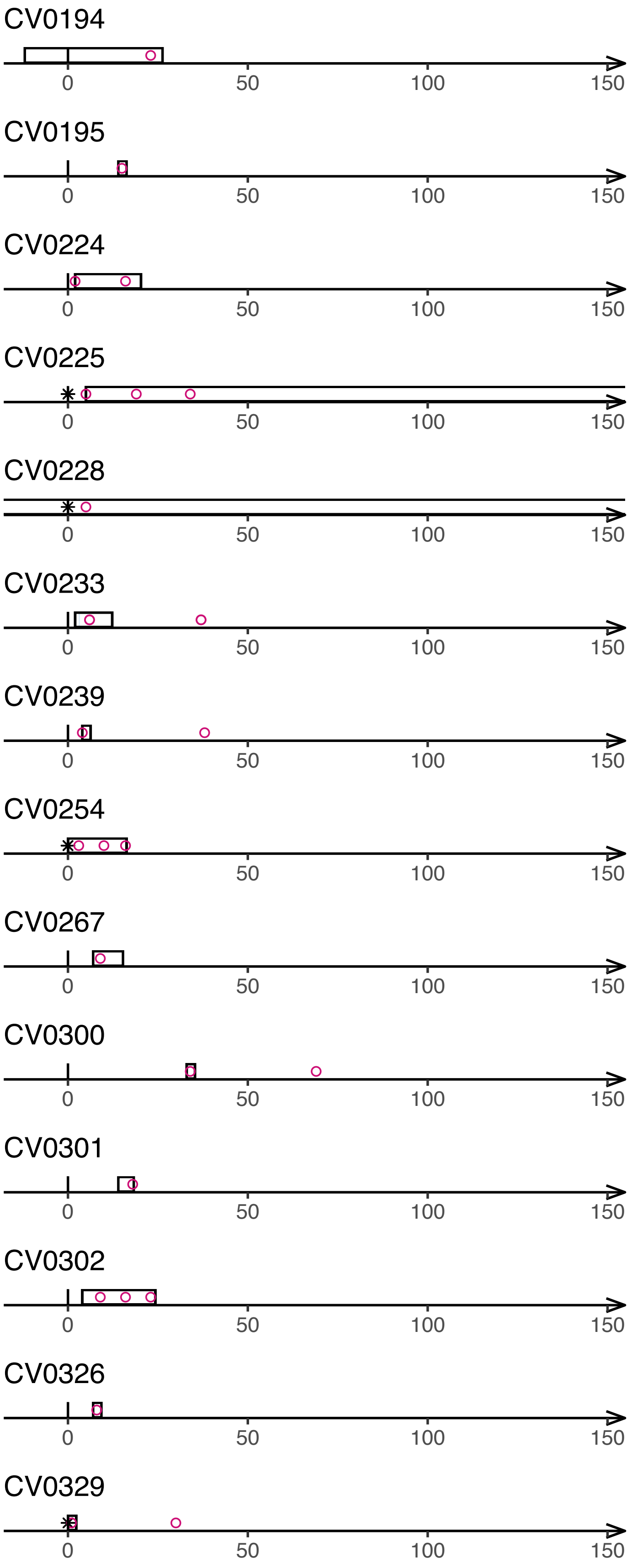
CV0194
0 50 100 150
CV0195
0 50 100 150
CV0224
0 50 100 150
CV0225
0 50 100 150
CV0228
0 50 100 150
CV0233
0 50 100 150
CV0239
0 50 100 150
CV0254
0 50 100 150
CV0267
0 50 100 150
CV0300
0 50 100 150
CV0301
0 50 100 150
CV0302
0 50 100 150
CV0326
0 50 100 150
CV0329
0 50 100 150

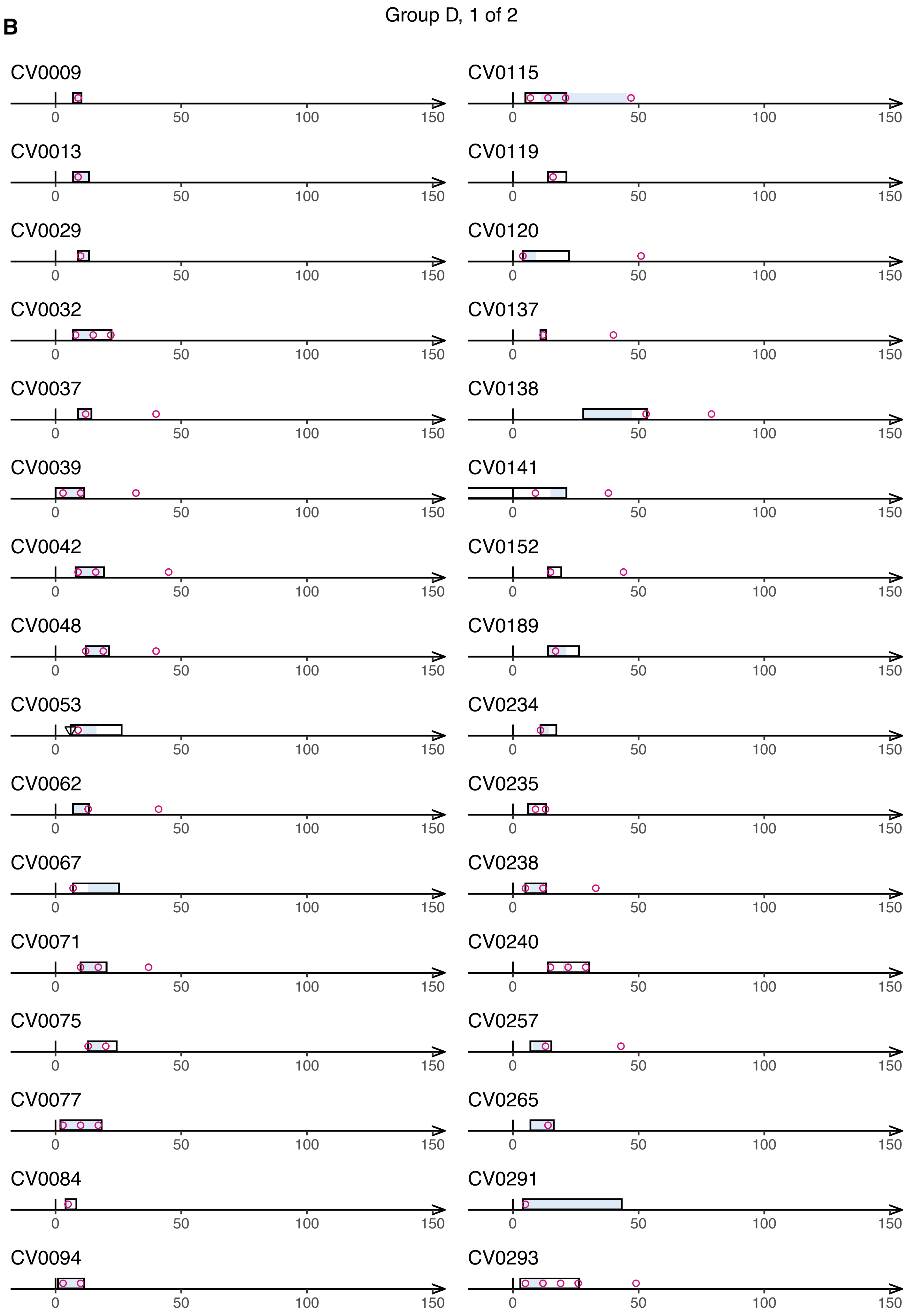
B
Group D, 1 of 2
CV0009
CV0013
CV0029
CV0032
CV0037
CV0039
CV0042
CV0048
CV0053
CV0062
CV0067
CV0071
CV0075
CV0077
CV0084
CV0094
CV0115
CV0119
CV0120
CV0137
CV0138
CV0141
CV0152
CV0189
CV0234
CV0235
CV0238
CV0240
CV0257
CV0265
CV0291
CV0293
0
50
100
150

Group D, 2 of 2

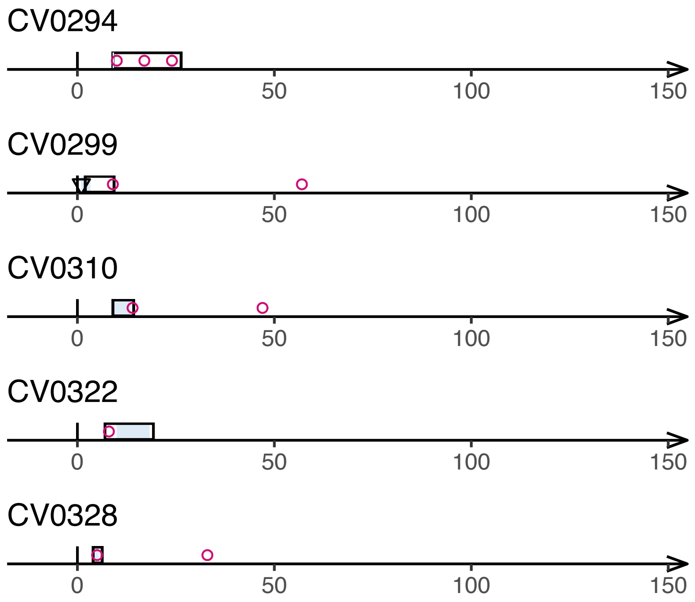
CV0294
0
50
100
150
CV0299
0
50
100
150
CV0310
0
50
100
150
CV0322
0
50
100
150
CV0328
0
50
100
150

C

Group E, 1 of 2

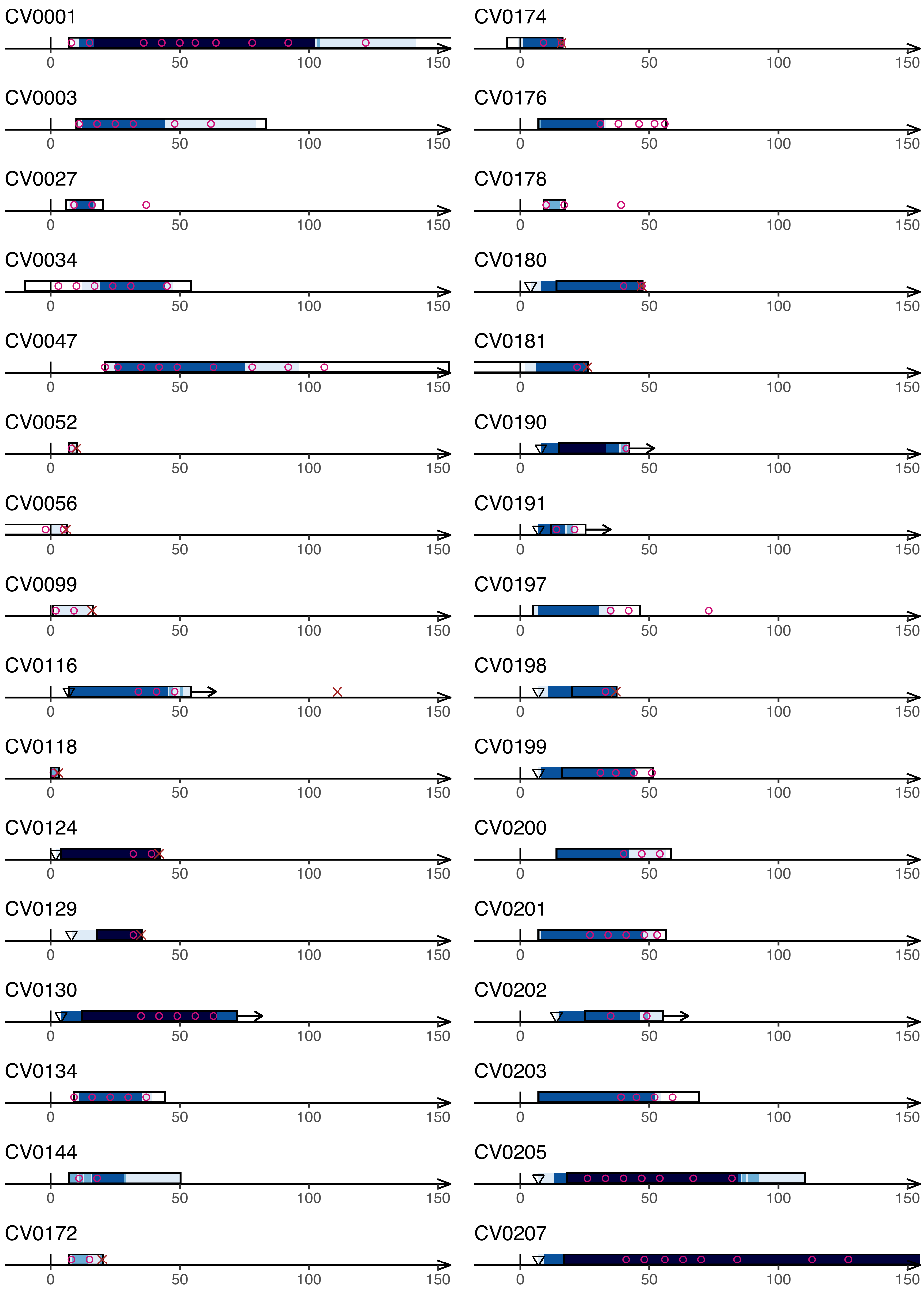

Group E, 2 of 2

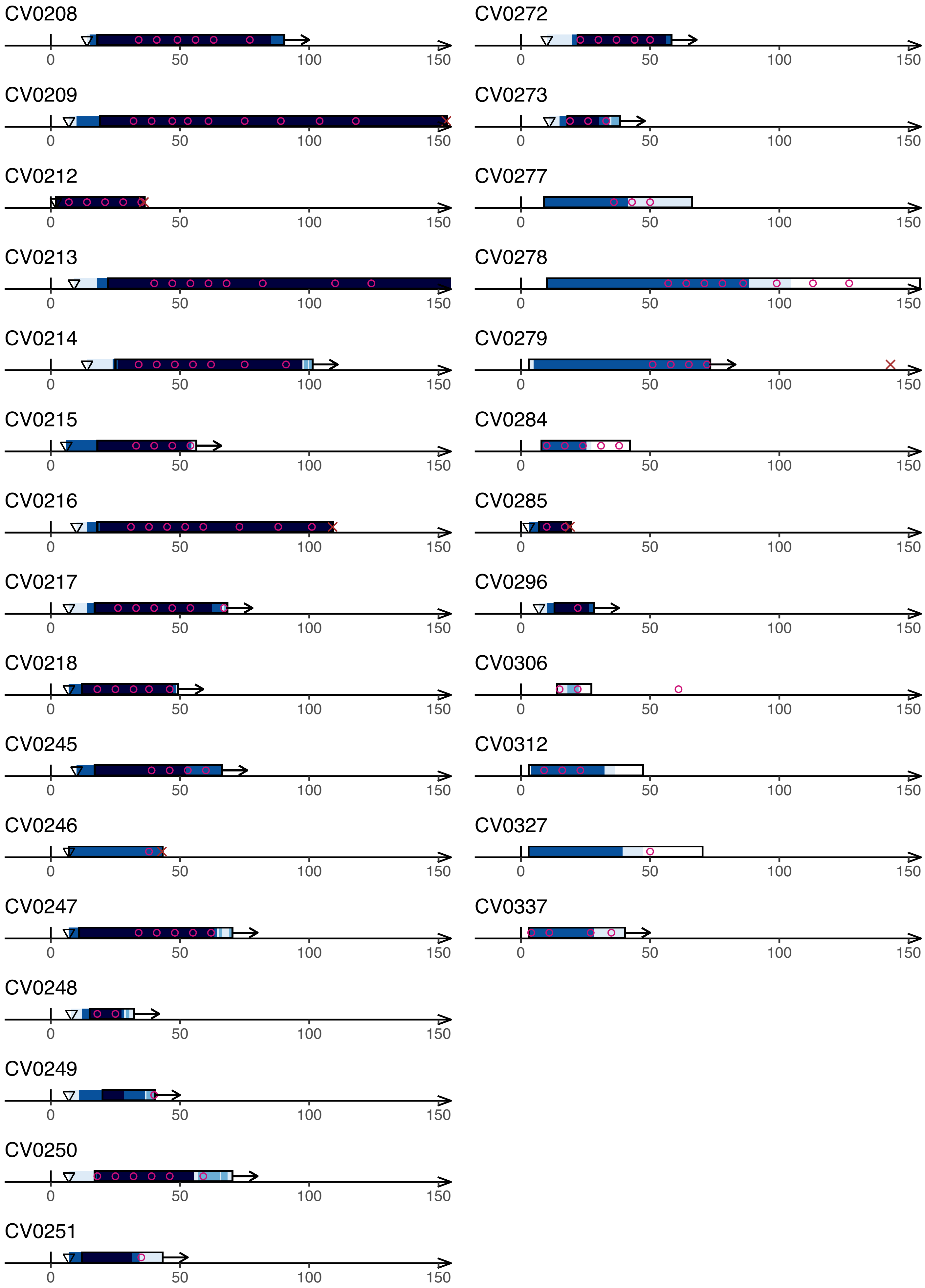
CV0208
CV0209
CV0212
CV0213
CV0214
CV0215
CV0216
CV0217
CV0218
CV0245
CV0246
CV0247
CV0248
CV0249
CV0250
CV0251
CV0272
CV0273
CV0277
CV0278
CV0279
CV0284
CV0285
CV0296
CV0306
CV0312
CV0327
CV0337
0
50
100
150

Data S2: Plots for the completed cell data, related to Figure 2

A) Boxplots showing absolute counts (cells/uL) for all the cell populations, split by severity categories and 12-day time bins post screening (group A) or symptom onset (groups B-E). Points are coloured based on asymptomatic or symptomatic classification for categories A and B respectively, normal or abnormal chest radiology (group C), and type of respiratory support at time of sampling (groups D and E), as per the key colour provided. **B)** Mixed-effects model with quadratic time trend showing the longitudinal trajectories of all the cell populations over time, grouped by severity. Nominal and adjusted p-values for the time x severity group interaction term are reported. Grey band in **A)** and **B)** indicates interquartile range of the corresponding population in healthy controls. NIV/HF, non-invasive ventilation/high-flow oxygen; vent, mechanical ventilation; ECMO, extracorporeal membrane oxygenation.

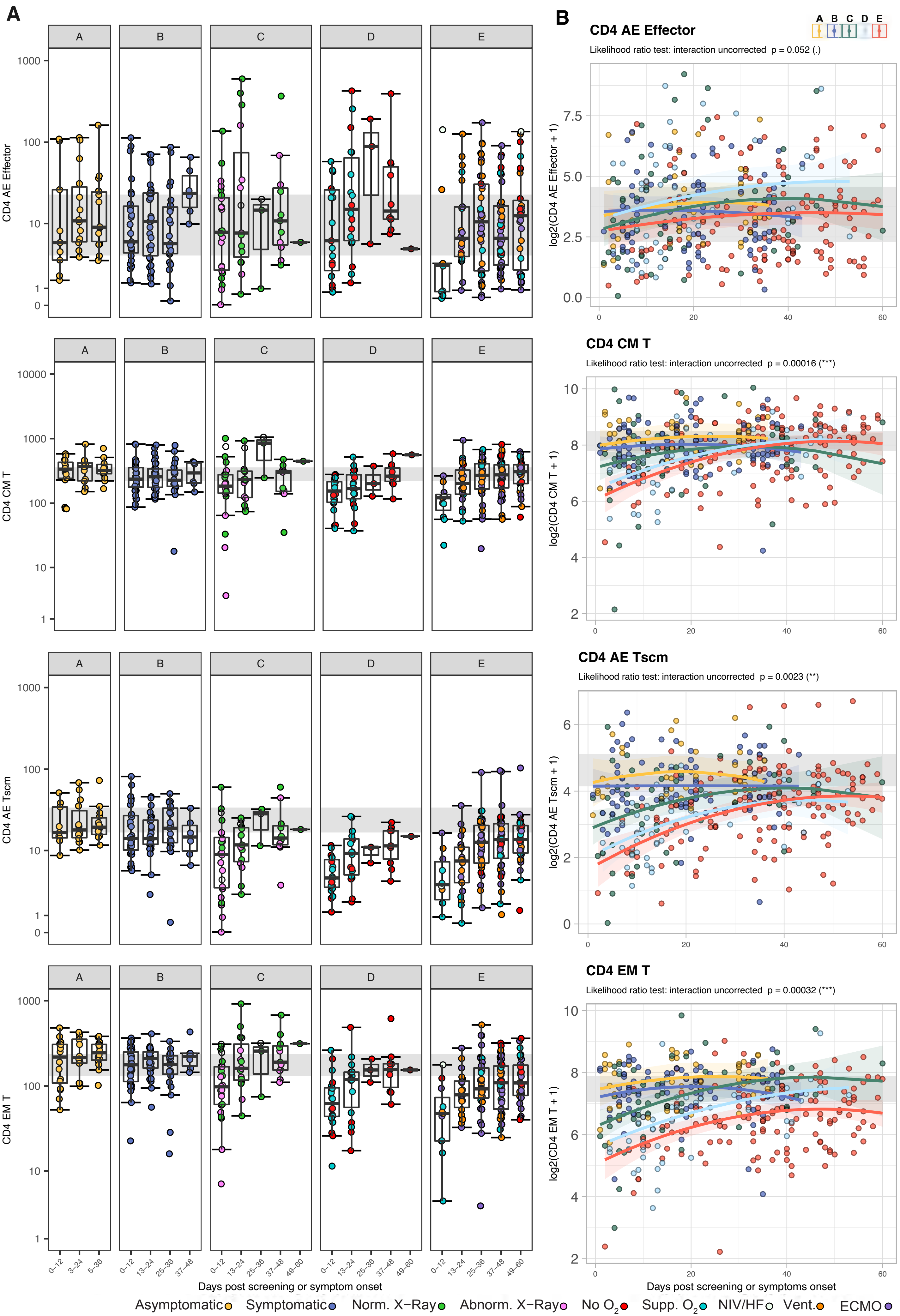

A
B
CD4 AE Effector
Likelihood ratio test: interaction uncorrected p = 0.052 (.)
log2(CD4 AE Effector + 1)
CD4 CM T
Likelihood ratio test: interaction uncorrected p = 0.00016 (***)
log2(CD4 CM T + 1)
CD4 AE Tscm
Likelihood ratio test: interaction uncorrected p = 0.0023 (**)
log2(CD4 AE Tscm + 1)
CD4 EM T
Likelihood ratio test: interaction uncorrected p = 0.00032 (***)
log2(CD4 EM T + 1)
0–12 13–24 25–36 37–48 49–60
Days post screening or symptom onset
Days post screening or symptoms onset
Asymptomatic Symptomatic Norm. X−Ray Abnorm. X−Ray No O2 Supp. O2 NIV/HFO Vent. ECMO

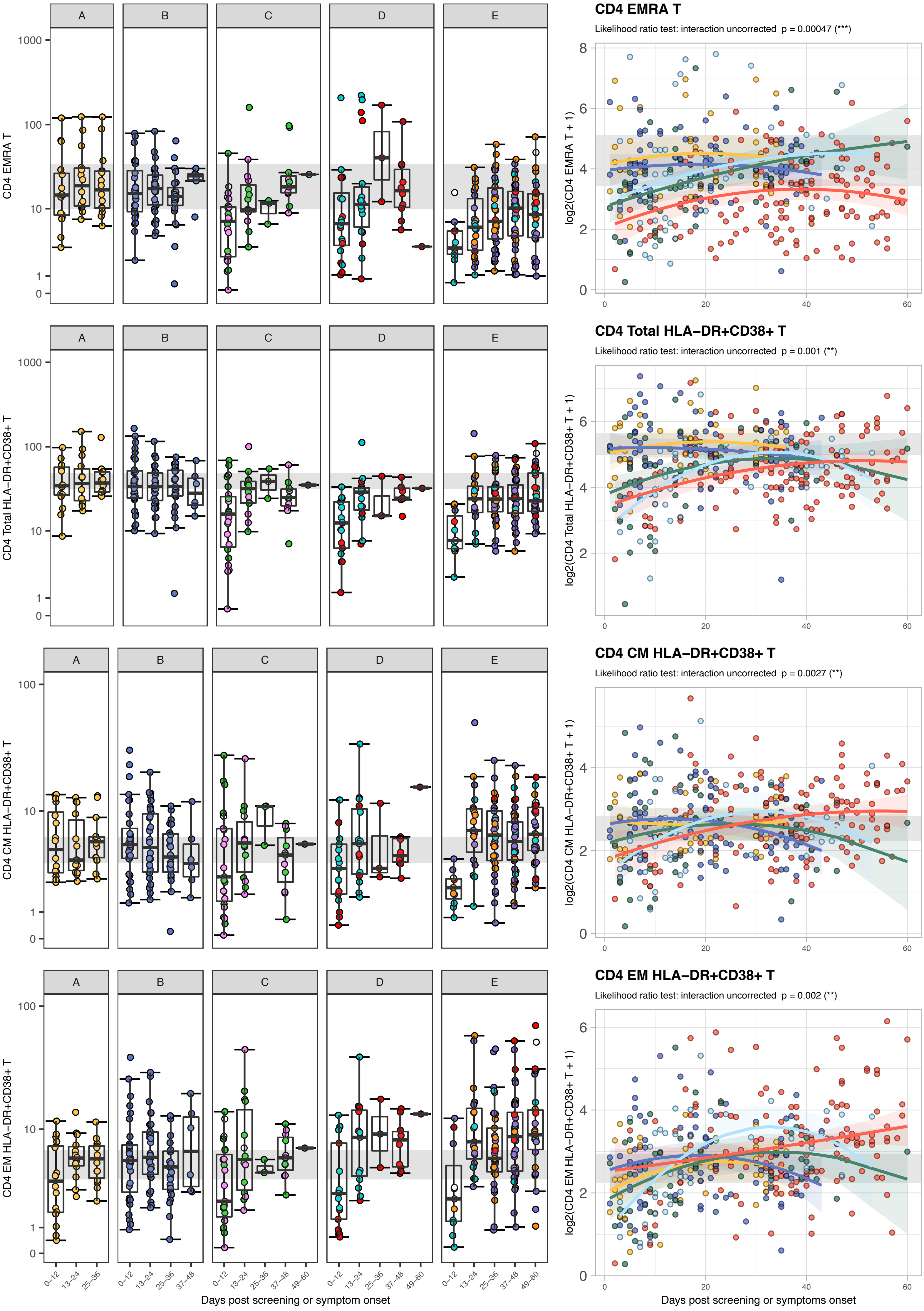

CD4 EMRA T
Likelihood ratio test: interaction uncorrected p = 0.00047 (***)
log2(CD4 EMRA T + 1)
CD4 Total HLA−DR+CD38+ T
Likelihood ratio test: interaction uncorrected p = 0.001 (**)
log2(CD4 Total HLA−DR+CD38+ T + 1)
CD4 CM HLA−DR+CD38+ T
Likelihood ratio test: interaction uncorrected p = 0.0027 (**)
log2(CD4 CM HLA−DR+CD38+ T + 1)
CD4 EM HLA−DR+CD38+ T
Likelihood ratio test: interaction uncorrected p = 0.002 (**)
log2(CD4 EM HLA−DR+CD38+ T + 1)
A
B
C
D
E
0−12
13−24
25−36
37−48
49−60
Days post screening or symptom onset
Days post screening or symptoms onset

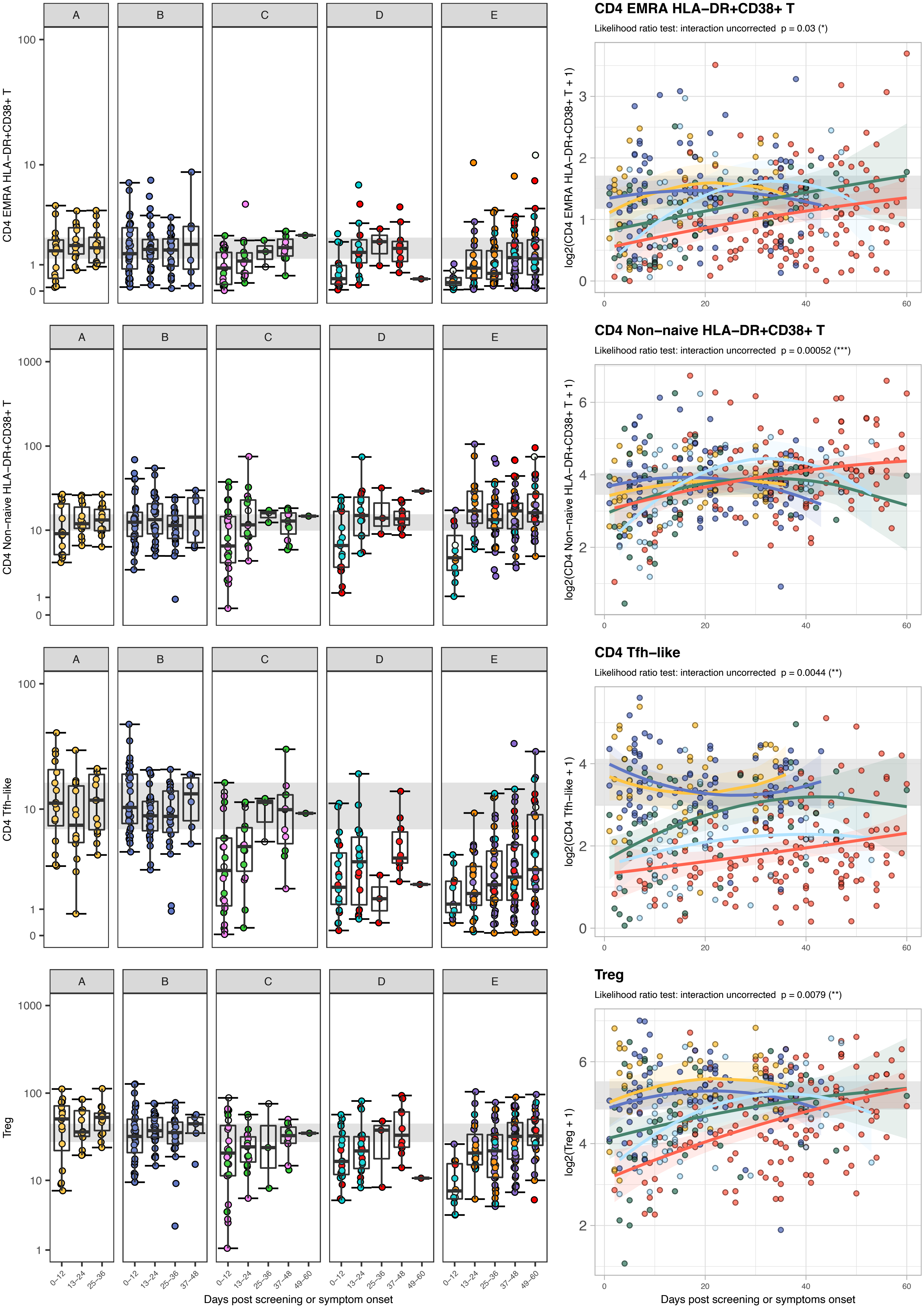

CD4 EMRA HLA−DR+CD38+ T
Likelihood ratio test: interaction uncorrected p = 0.03 (*)
log2(CD4 EMRA HLA−DR+CD38+ T + 1)
CD4 Non−naive HLA−DR+CD38+ T
Likelihood ratio test: interaction uncorrected p = 0.00052 (***)
log2(CD4 Non−naive HLA−DR+CD38+ T + 1)
CD4 Tfh−like
Likelihood ratio test: interaction uncorrected p = 0.0044 (**)
log2(CD4 Tfh−like + 1)
Treg
Likelihood ratio test: interaction uncorrected p = 0.0079 (**)
log2(Treg + 1)
A
B
C
D
E
CD4 Tfh−like
Days post screening or symptom onset
Days post screening or symptoms onset

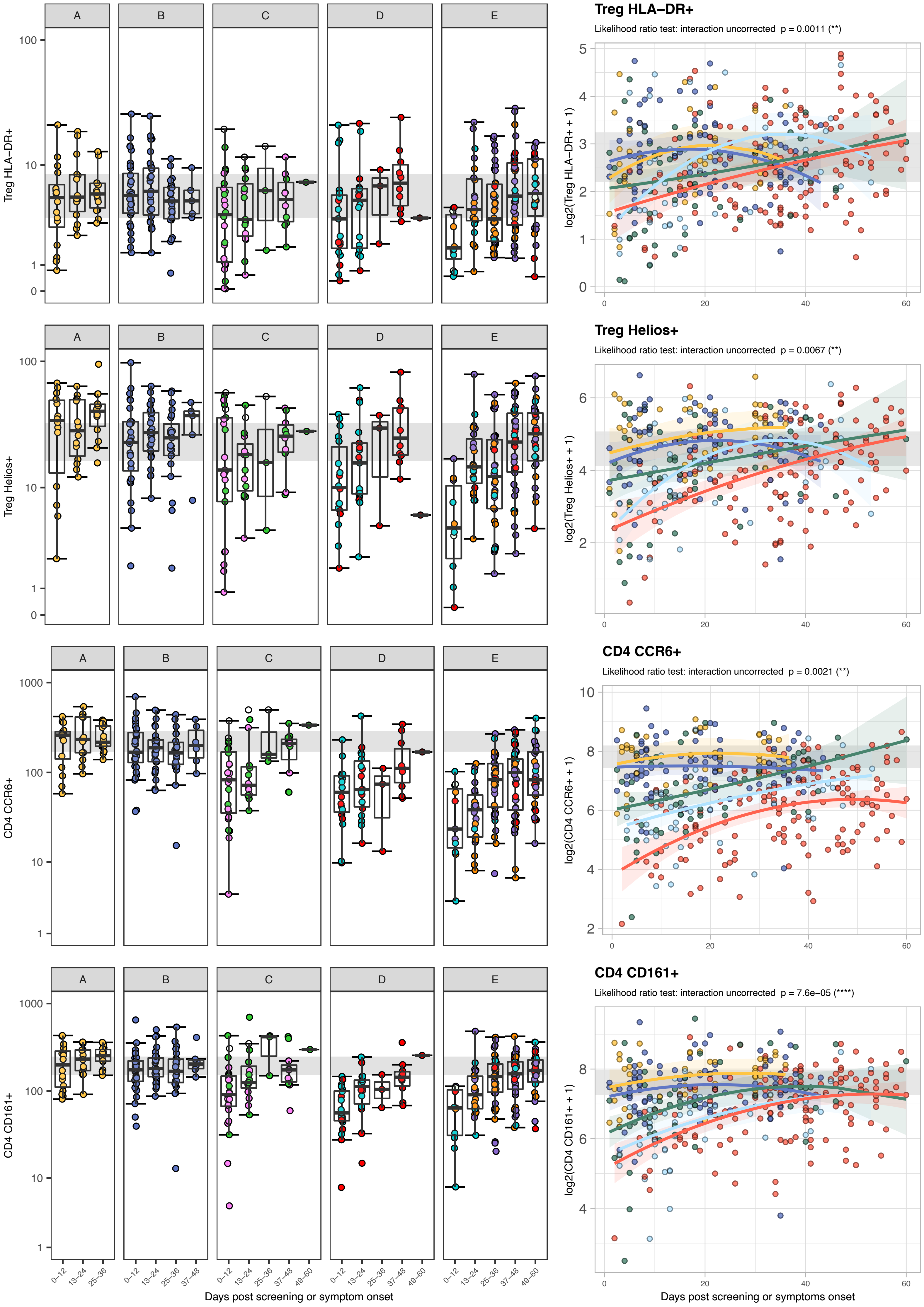

Treg HLA−DR+
Likelihood ratio test: interaction uncorrected p = 0.0011 (**)
log2(Treg HLA−DR+ + 1)
Treg Helios+
Likelihood ratio test: interaction uncorrected p = 0.0067 (**)
log2(Treg Helios+ + 1)
CD4 CCR6+
Likelihood ratio test: interaction uncorrected p = 0.0021 (**)
log2(CD4 CCR6+ + 1)
CD4 CD161+
Likelihood ratio test: interaction uncorrected p = 7.6e−05 (****)
log2(CD4 CD161+ + 1)
Days post screening or symptoms onset
A
B
C
D
E
Treg HLA−DR+
Treg Helios+
CD4 CCR6+
CD4 CD161+
0−12
13−24
25−36
37−48
49−60
Days post screening or symptom onset

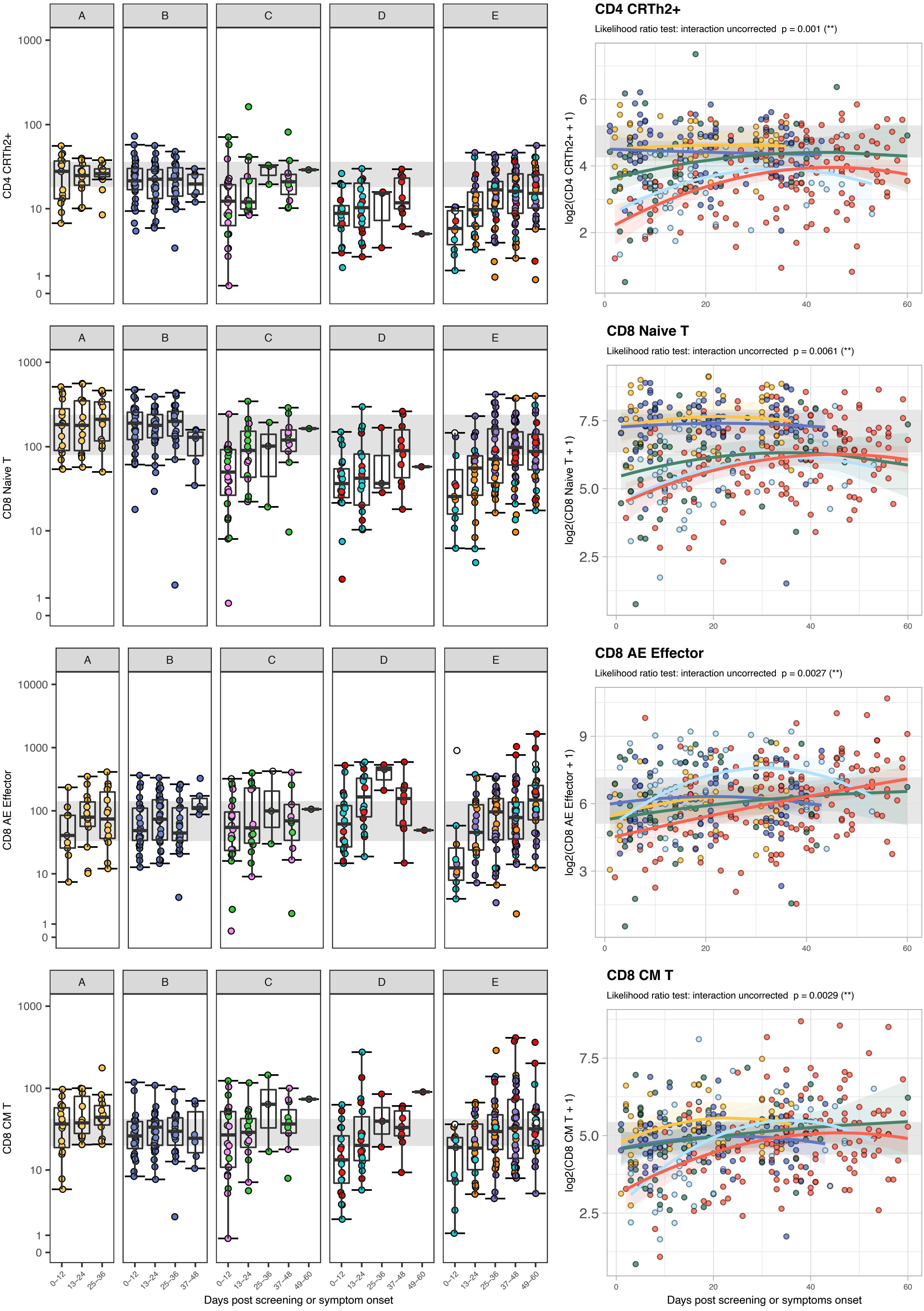

CD4 CRTh2+
Likelihood ratio test: interaction uncorrected p = 0.001 (**)
log2(CD4 CRTh2+ + 1)
CD8 Naive T
Likelihood ratio test: interaction uncorrected p = 0.0061 (**)
log2(CD8 Naive T + 1)
CD8 AE Effector
Likelihood ratio test: interaction uncorrected p = 0.0027 (**)
log2(CD8 AE Effector + 1)
CD8 CM T
Likelihood ratio test: interaction uncorrected p = 0.0029 (**)
log2(CD8 CM T + 1)
A
B
C
D
E
0–12
13–24
25–36
37–48
49–60
Days post screening or symptom onset
Days post screening or symptoms onset

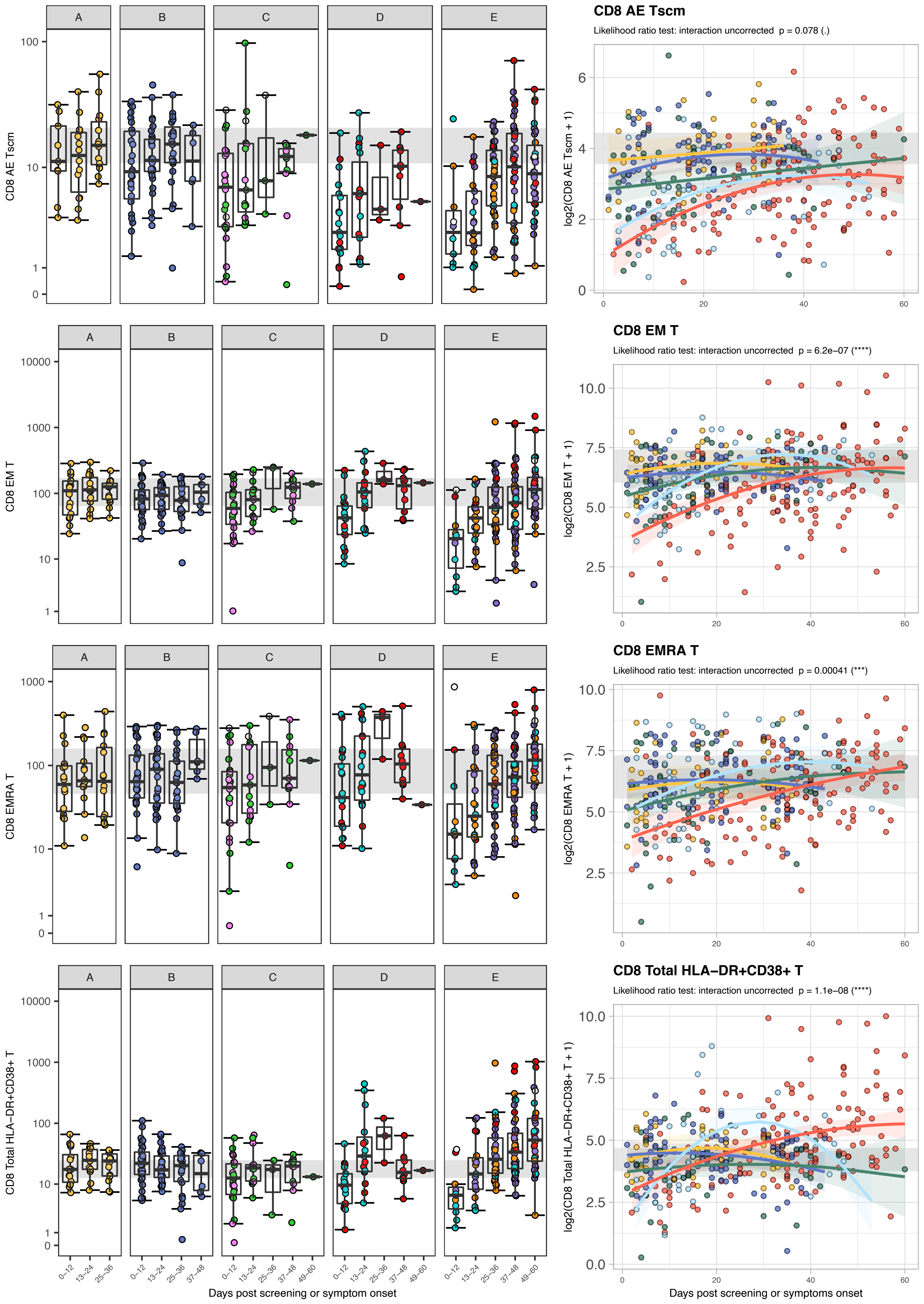
A
B
C
D
E
CD8 AE Tscm
CD8 AE Tscm
Likelihood ratio test: interaction uncorrected p = 0.078 (.)
log2(CD8 AE Tscm + 1)
CD8 EM T
CD8 EM T
Likelihood ratio test: interaction uncorrected p = 6.2e−07 (****)
log2(CD8 EM T + 1)
CD8 EMRA T
CD8 EMRA T
Likelihood ratio test: interaction uncorrected p = 0.00041 (***)
log2(CD8 EMRA T + 1)
CD8 Total HLA−DR+CD38+ T
CD8 Total HLA−DR+CD38+ T
Likelihood ratio test: interaction uncorrected p = 1.1e−08 (****)
log2(CD8 Total HLA−DR+CD38+ T + 1)
0–12
13–24
25–36
37–48
49–60
Days post screening or symptom onset
Days post screening or symptoms onset

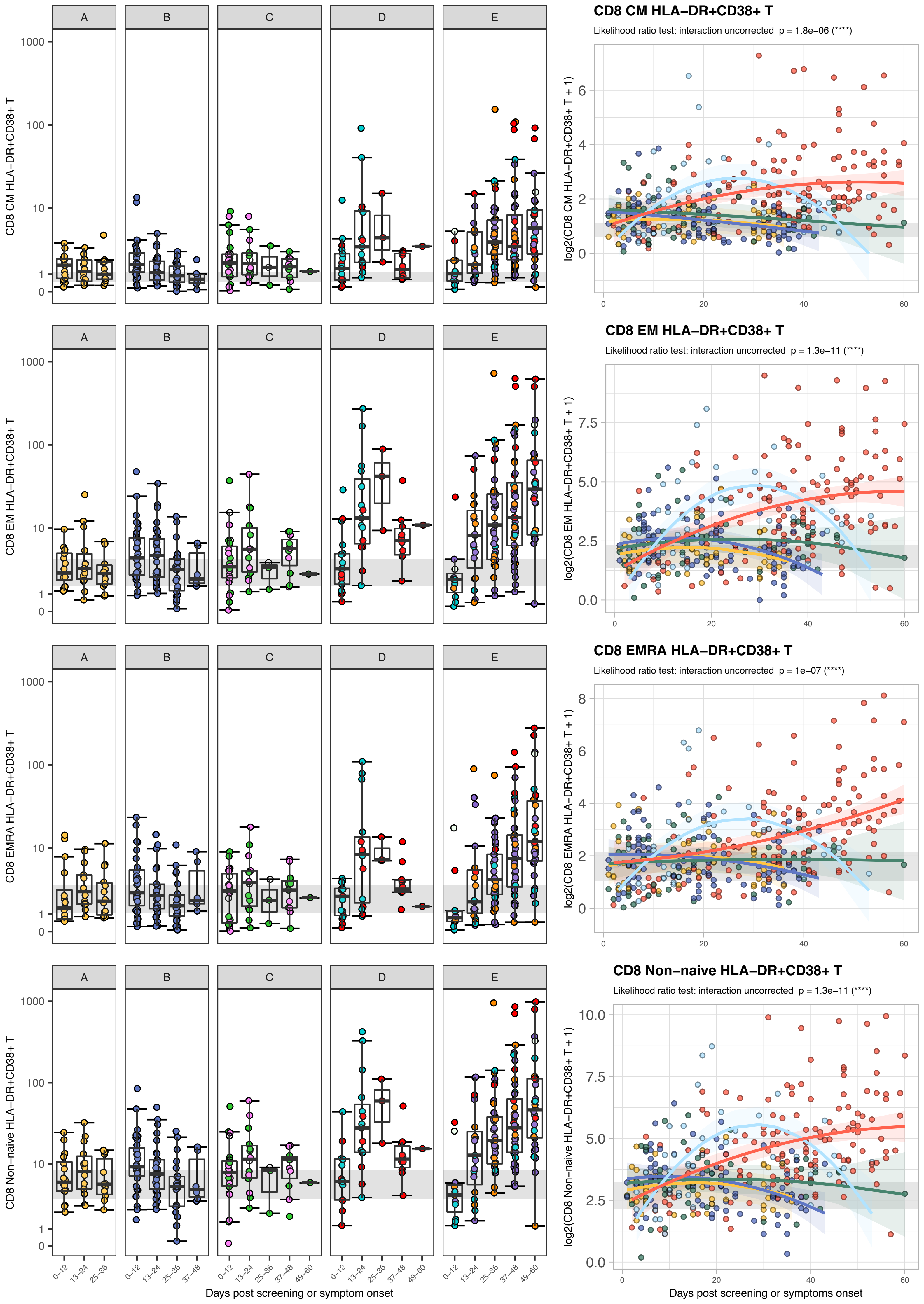

CD8 CM HLA−DR+CD38+ T
Likelihood ratio test: interaction uncorrected p = 1.8e−06 (****)
log2(CD8 CM HLA−DR+CD38+ T + 1)
CD8 CM HLA−DR+CD38+ T
CD8 EM HLA−DR+CD38+ T
Likelihood ratio test: interaction uncorrected p = 1.3e−11 (****)
log2(CD8 EM HLA−DR+CD38+ T + 1)
CD8 EM HLA−DR+CD38+ T
CD8 EMRA HLA−DR+CD38+ T
Likelihood ratio test: interaction uncorrected p = 1e−07 (****)
log2(CD8 EMRA HLA−DR+CD38+ T + 1)
CD8 EMRA HLA−DR+CD38+ T
CD8 Non−naive HLA−DR+CD38+ T
Likelihood ratio test: interaction uncorrected p = 1.3e−11 (****)
log2(CD8 Non−naive HLA−DR+CD38+ T + 1)
CD8 Non−naive HLA−DR+CD38+ T
A
B
C
D
E
0−12
13−24
25−36
37−48
49−60
Days post screening or symptom onset
Days post screening or symptoms onset

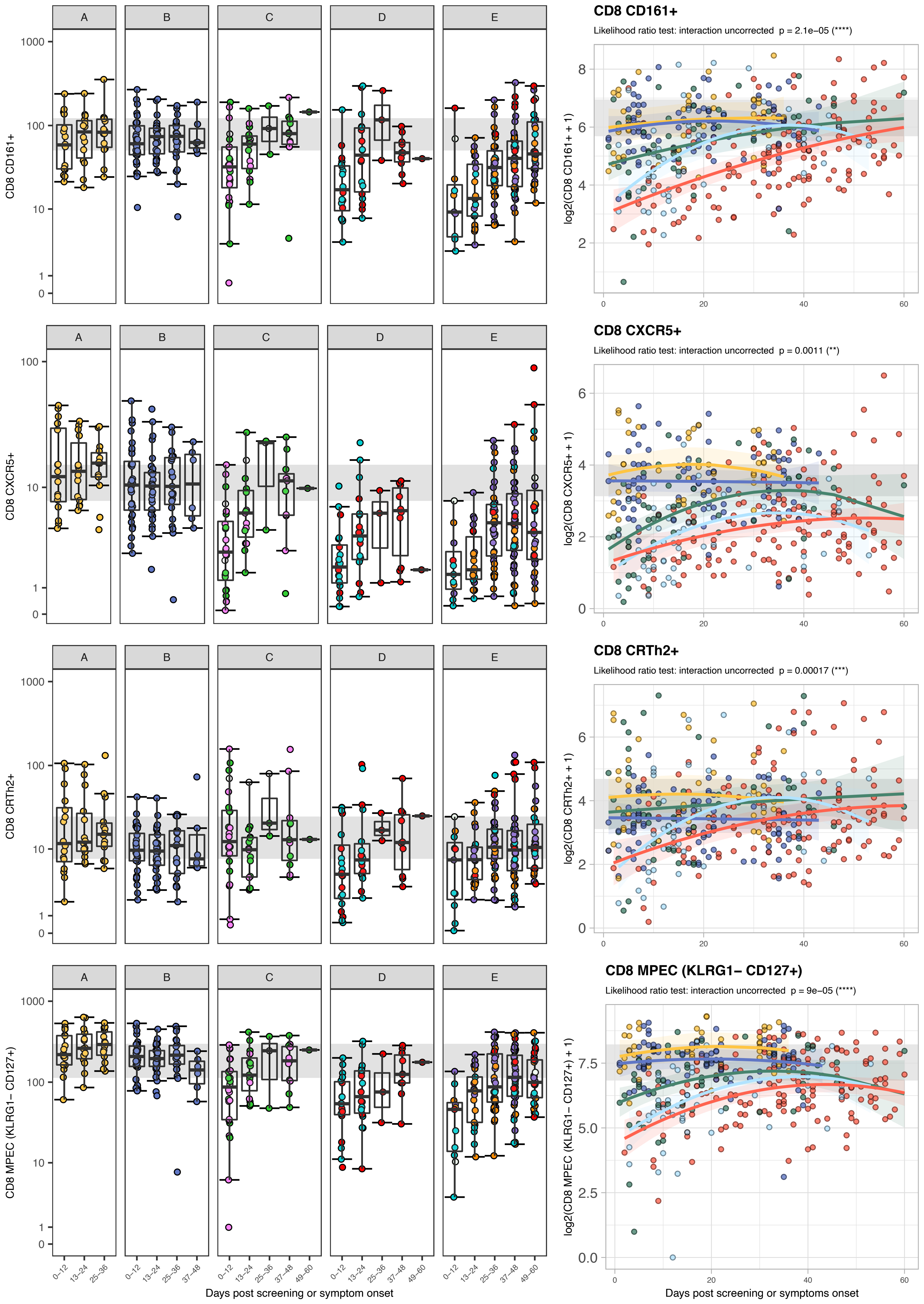

CD8 CD161+
Likelihood ratio test: interaction uncorrected p = 2.1e−05 (****)
log2(CD8 CD161+ + 1)
CD8 CXCR5+
Likelihood ratio test: interaction uncorrected p = 0.0011 (**)
log2(CD8 CXCR5+ + 1)
CD8 CRTh2+
Likelihood ratio test: interaction uncorrected p = 0.00017 (***)
log2(CD8 CRTh2+ + 1)
CD8 MPEC (KLRG1− CD127+)
Likelihood ratio test: interaction uncorrected p = 9e−05 (****)
log2(CD8 MPEC (KLRG1− CD127+) + 1)
A
B
C
D
E
0–12
13–24
25–36
37–48
49–60
Days post screening or symptom onset
Days post screening or symptoms onset

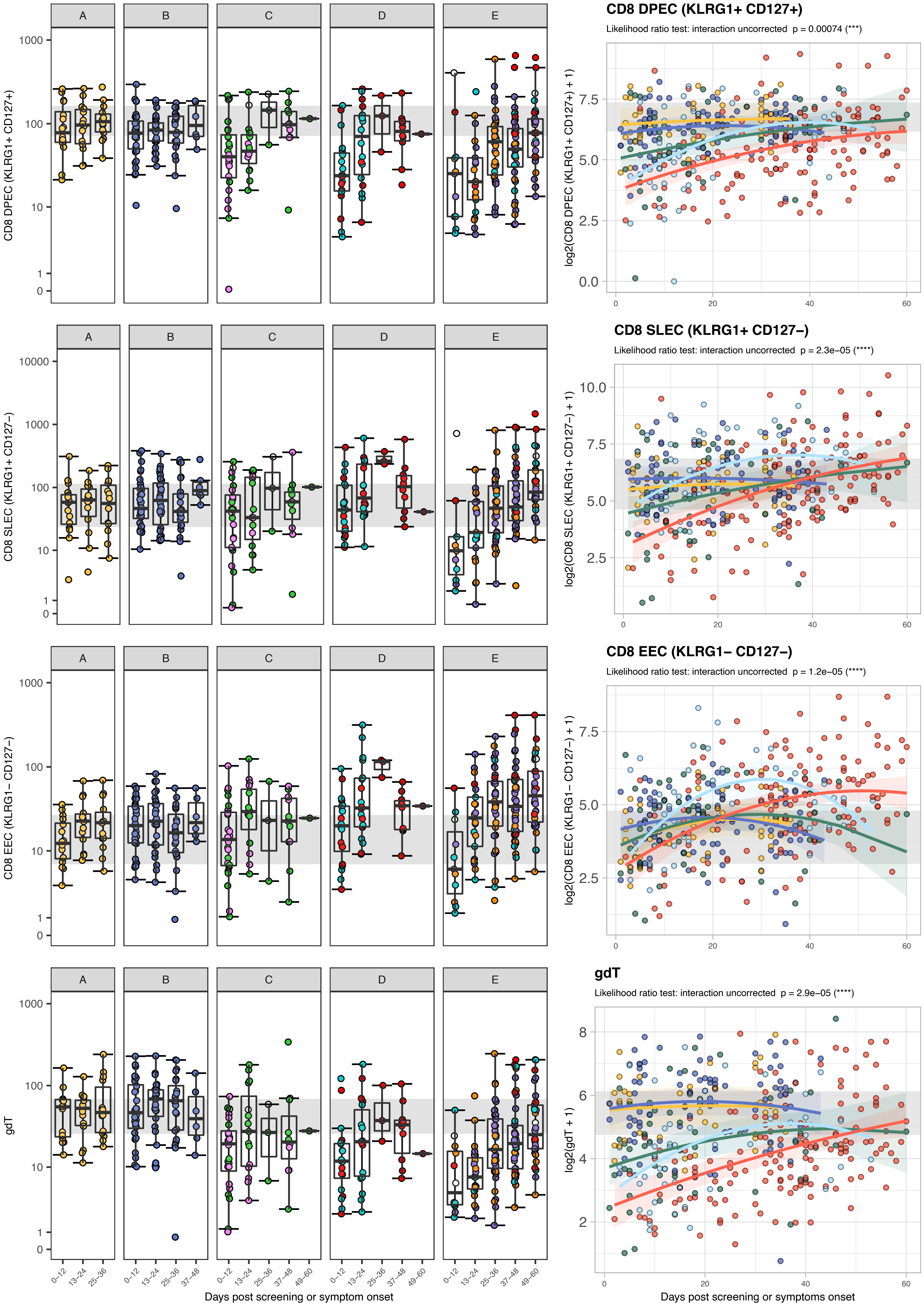
CD8 DPEC (KLRG1+ CD127+)
Likelihood ratio test: interaction uncorrected p = 0.00074 (***)
CD8 DPEC (KLRG1+ CD127+)
log2(CD8 DPEC (KLRG1+ CD127+) + 1)
CD8 SLEC (KLRG1+ CD127−)
Likelihood ratio test: interaction uncorrected p = 2.3e−05 (****)
CD8 SLEC (KLRG1+ CD127−)
log2(CD8 SLEC (KLRG1+ CD127−) + 1)
CD8 EEC (KLRG1− CD127−)
Likelihood ratio test: interaction uncorrected p = 1.2e−05 (****)
CD8 EEC (KLRG1− CD127−)
log2(CD8 EEC (KLRG1− CD127−) + 1)
gdT
Likelihood ratio test: interaction uncorrected p = 2.9e−05 (****)
gdT
log2(gdT + 1)
A
B
C
D
E
0–12
13–24
25–36
37–48
49–60
Days post screening or symptom onset
Days post screening or symptoms onset

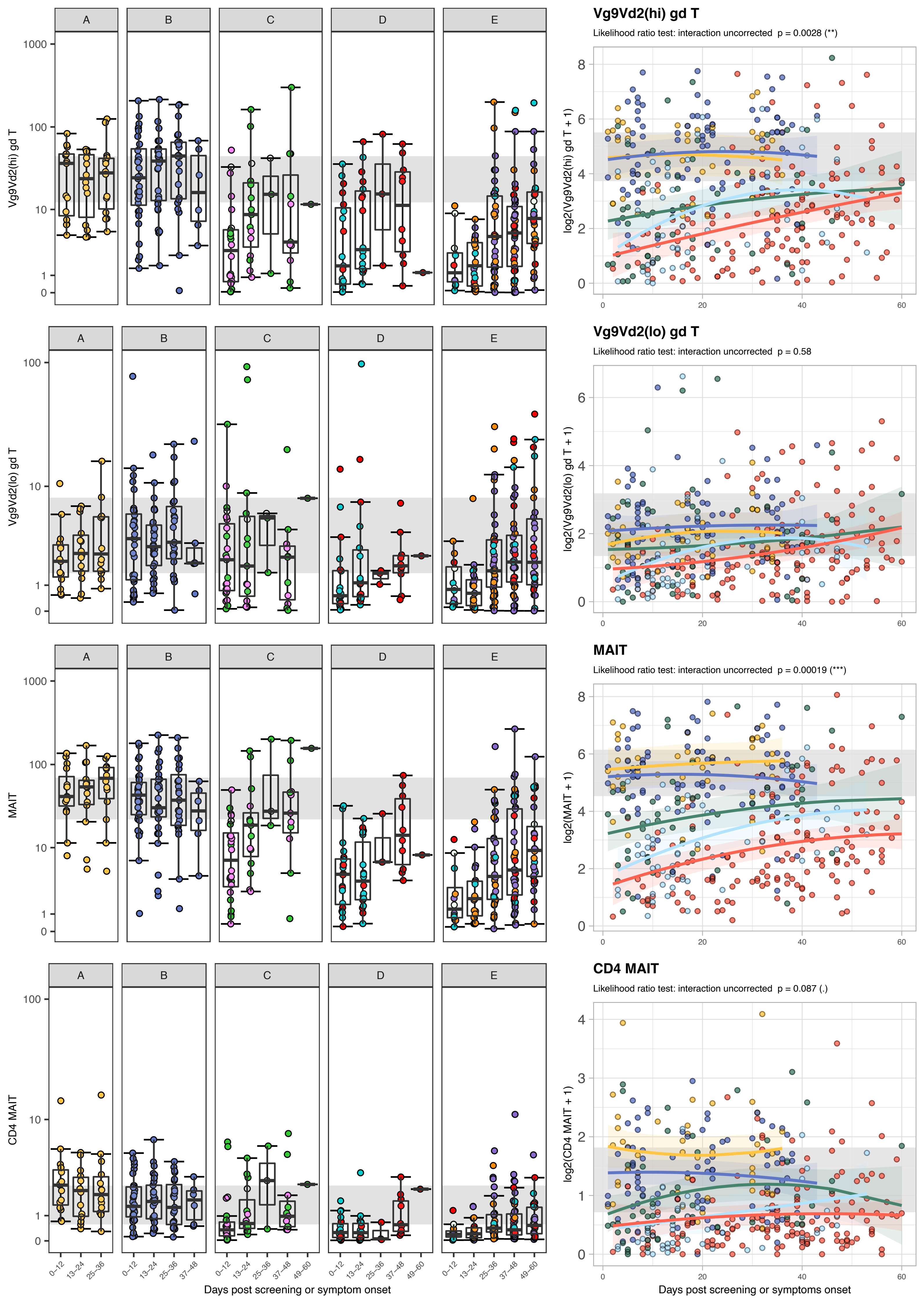

Vg9Vd2(hi) gd T
Likelihood ratio test: interaction uncorrected p = 0.0028 (**)
Vg9Vd2(lo) gd T
Likelihood ratio test: interaction uncorrected p = 0.58
MAIT
Likelihood ratio test: interaction uncorrected p = 0.00019 (***)
CD4 MAIT
Likelihood ratio test: interaction uncorrected p = 0.087 (.)
A
B
C
D
E
0–12
13–24
25–36
37–48
49–60
Vg9Vd2(hi) gd T
Vg9Vd2(lo) gd T
MAIT
CD4 MAIT
log2(Vg9Vd2(hi) gd T + 1)
log2(Vg9Vd2(lo) gd T + 1)
log2(MAIT + 1)
log2(CD4 MAIT + 1)
Days post screening or symptom onset
Days post screening or symptoms onset

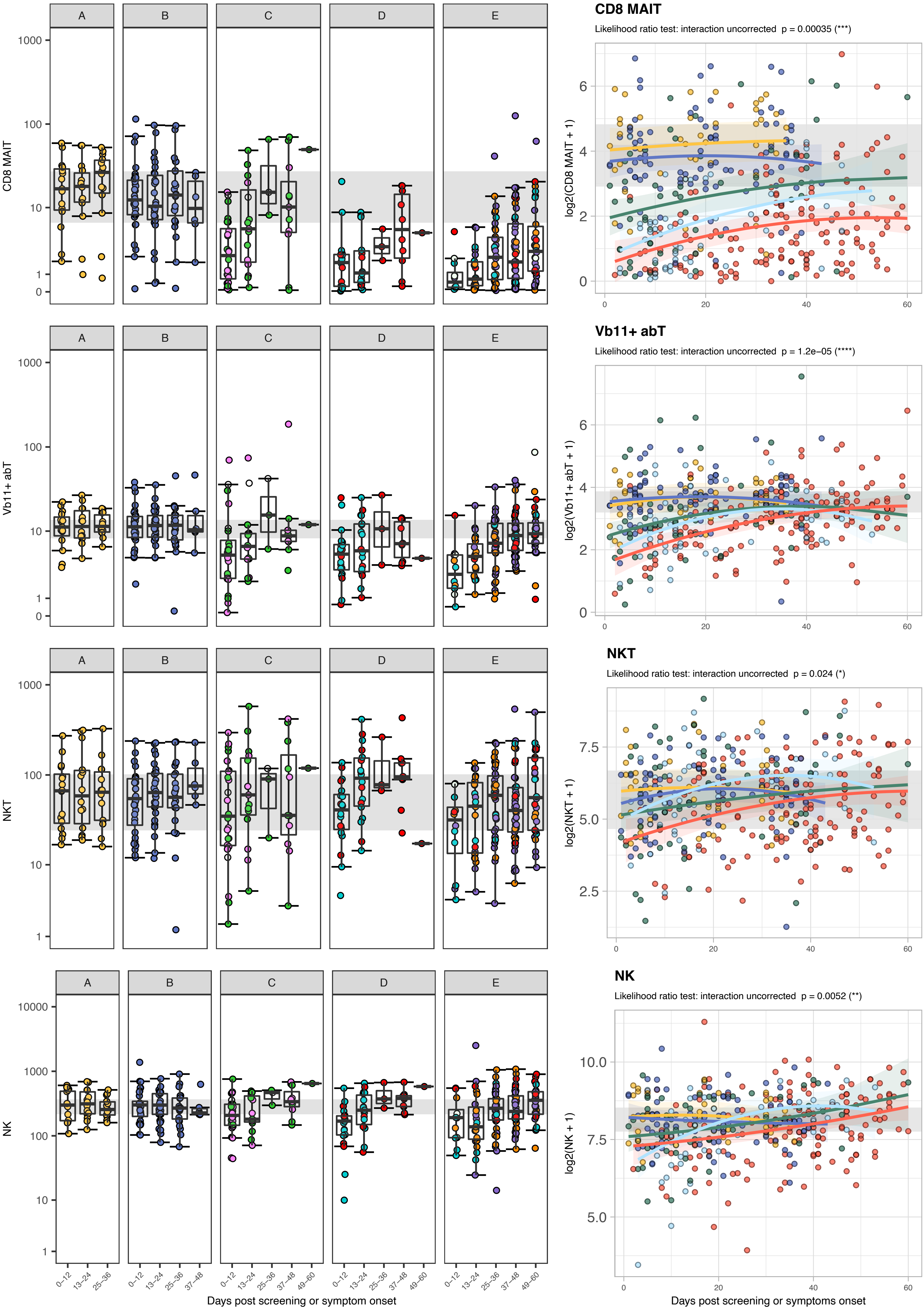
CD8 MAIT
Likelihood ratio test: interaction uncorrected p = 0.00035 (***)
log2(CD8 MAIT + 1)
Vb11+ abT
Likelihood ratio test: interaction uncorrected p = 1.2e−05 (****)
log2(Vb11+ abT + 1)
NKT
Likelihood ratio test: interaction uncorrected p = 0.024 (*)
log2(NKT + 1)
NK
Likelihood ratio test: interaction uncorrected p = 0.0052 (**)
log2(NK + 1)
A
B
C
D
E
0–12
13–24
25–36
37–48
49–60
Days post screening or symptom onset
Days post screening or symptoms onset

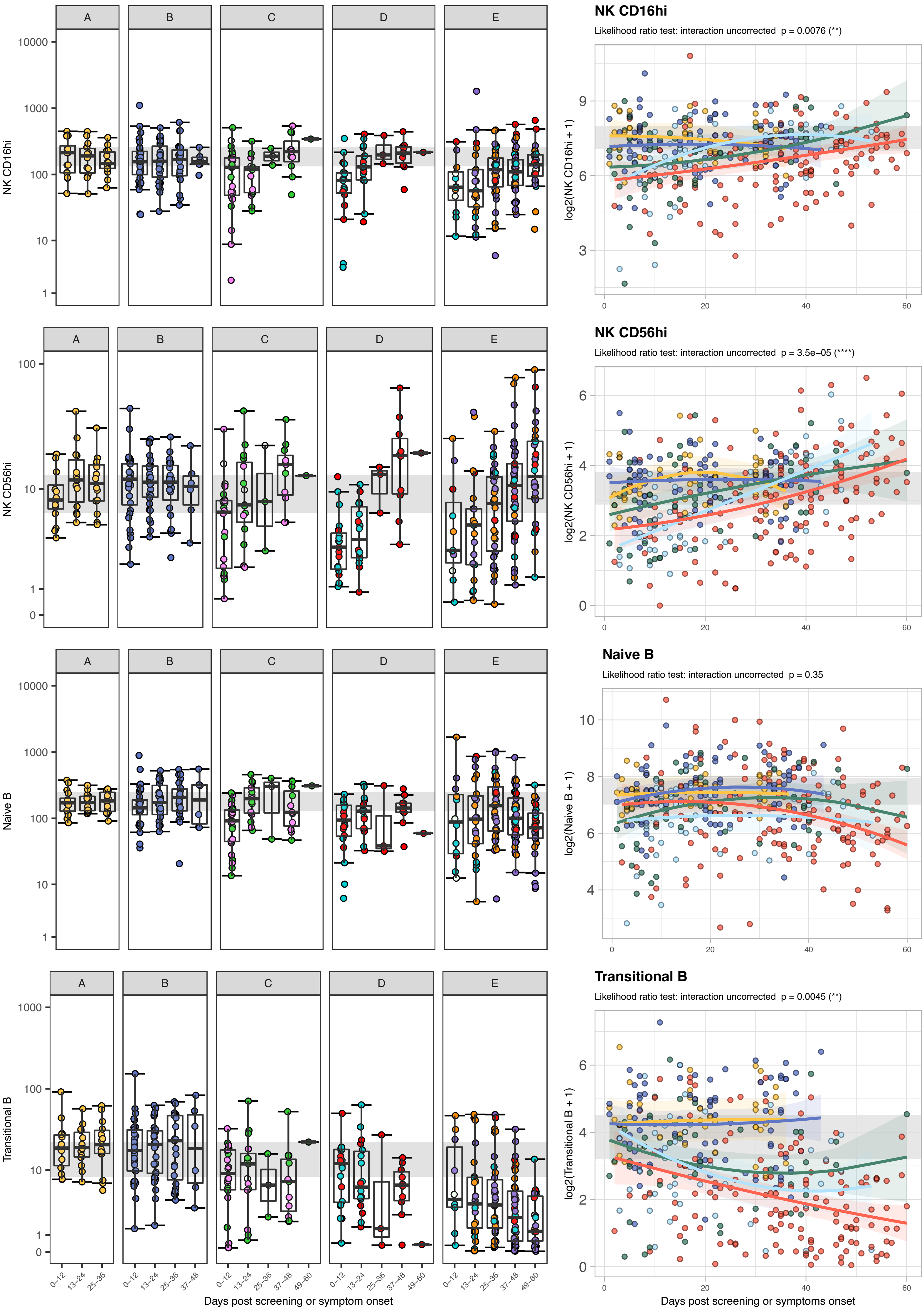
A
B
C
D
E
NK CD16hi
Likelihood ratio test: interaction uncorrected p = 0.0076 (**)
log2(NK CD16hi + 1)
NK CD56hi
Likelihood ratio test: interaction uncorrected p = 3.5e−05 (****)
log2(NK CD56hi + 1)
Naive B
Likelihood ratio test: interaction uncorrected p = 0.35
log2(Naive B + 1)
Transitional B
Likelihood ratio test: interaction uncorrected p = 0.0045 (**)
log2(Transitional B + 1)
0–12
13–24
25–36
37–48
49–60
Days post screening or symptom onset
Days post screening or symptoms onset

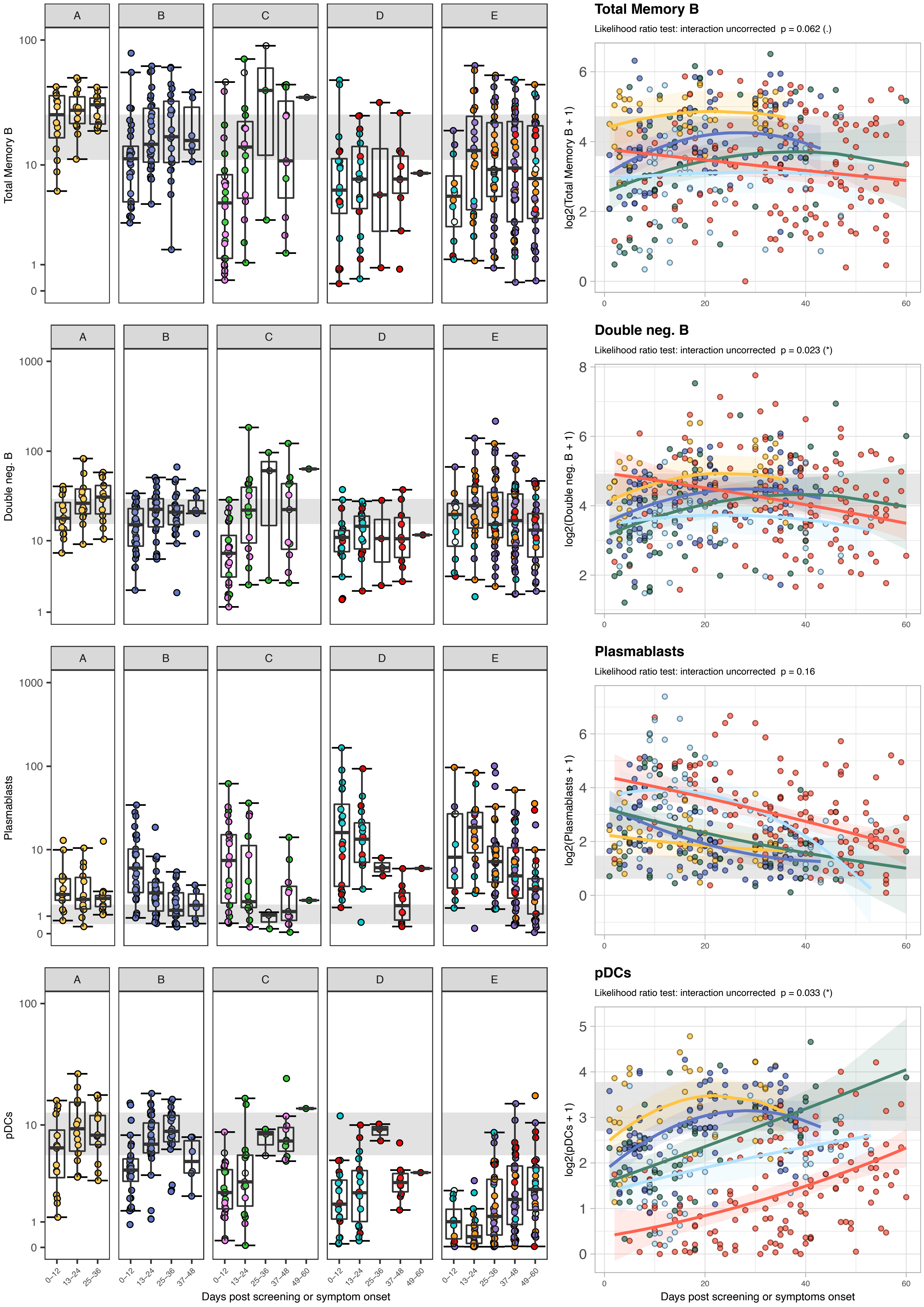

Total Memory B
Likelihood ratio test: interaction uncorrected p = 0.062 (.)
Double neg. B
Likelihood ratio test: interaction uncorrected p = 0.023 (*)
Plasmablasts
Likelihood ratio test: interaction uncorrected p = 0.16
pDCs
Likelihood ratio test: interaction uncorrected p = 0.033 (*)
A
B
C
D
E
log2(Total Memory B + 1)
log2(Double neg. B + 1)
log2(Plasmablasts + 1)
log2(pDCs + 1)
0–12
13–24
25–36
37–48
49–60
Days post screening or symptom onset
Days post screening or symptoms onset

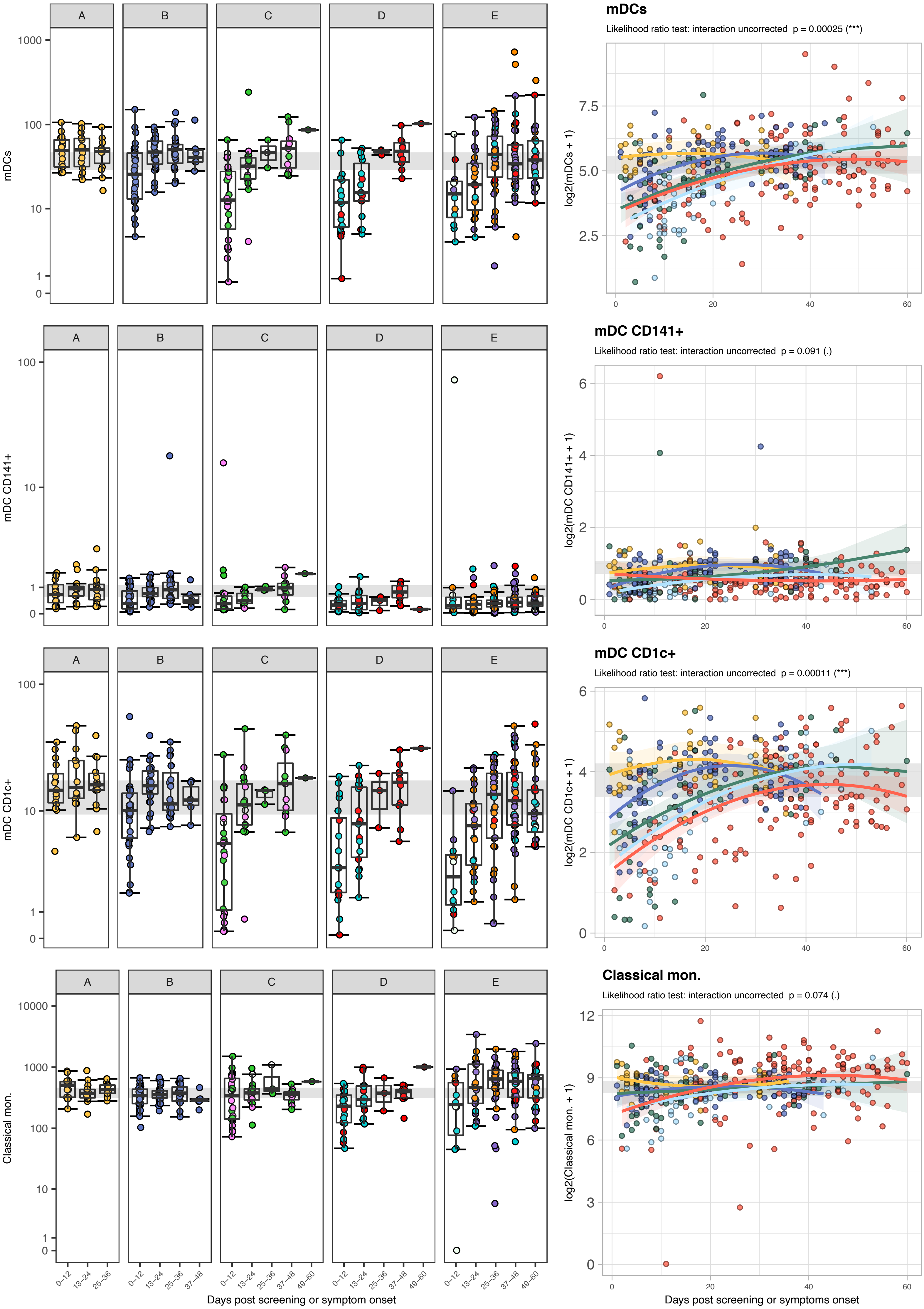

A
B
C
D
E
mDCs
Likelihood ratio test: interaction uncorrected p = 0.00025 (***)
log2(mDCs + 1)
mDC CD141+
Likelihood ratio test: interaction uncorrected p = 0.091 (.)
log2(mDC CD141+ + 1)
mDC CD1c+
Likelihood ratio test: interaction uncorrected p = 0.00011 (***)
log2(mDC CD1c+ + 1)
Classical mon.
Likelihood ratio test: interaction uncorrected p = 0.074 (.)
log2(Classical mon. + 1)
0–12
13–24
25–36
37–48
49–60
Days post screening or symptom onset
Days post screening or symptoms onset

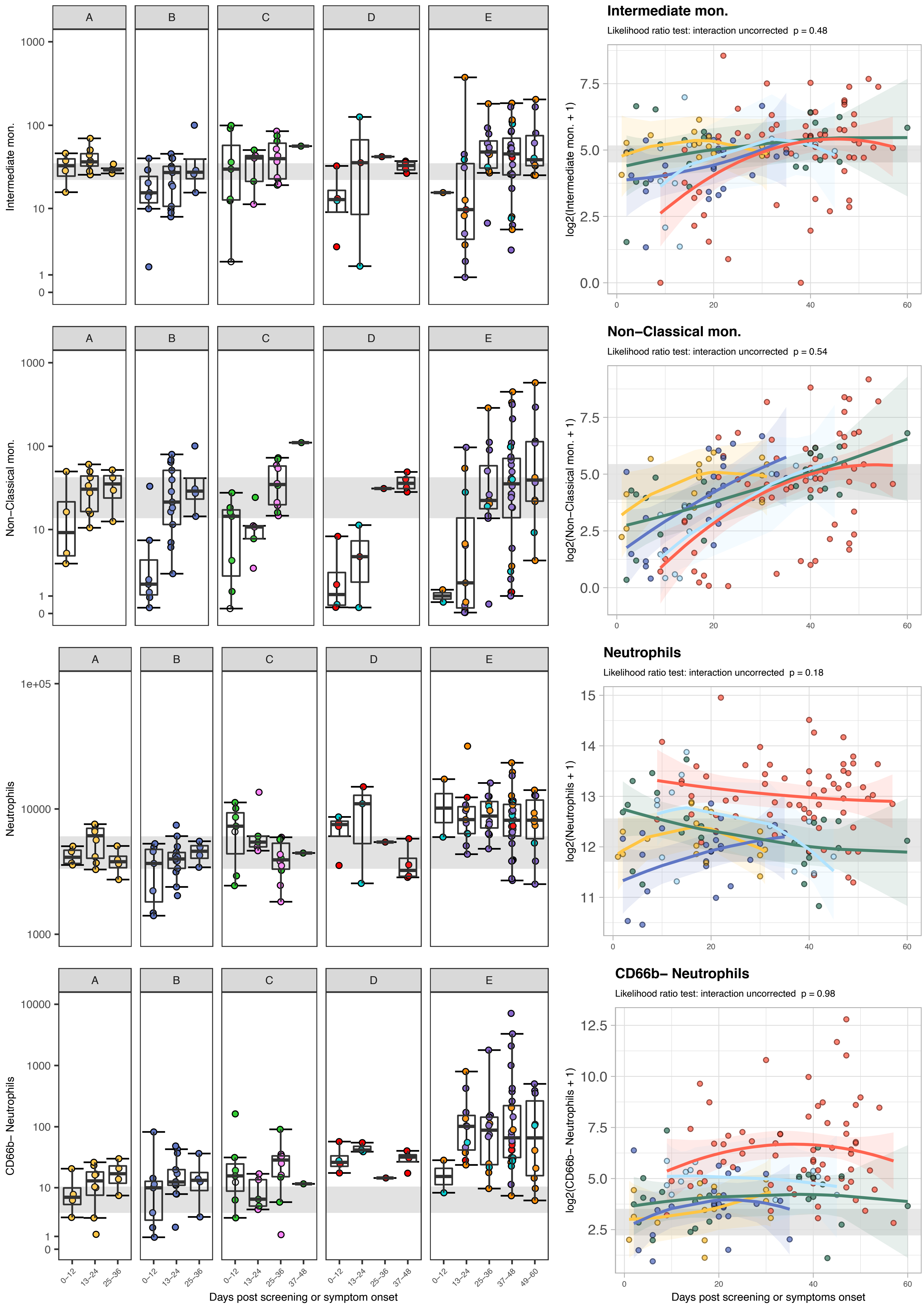

Intermediate mon.
Likelihood ratio test: interaction uncorrected p = 0.48
Non−Classical mon.
Likelihood ratio test: interaction uncorrected p = 0.54
Neutrophils
Likelihood ratio test: interaction uncorrected p = 0.18
CD66b− Neutrophils
Likelihood ratio test: interaction uncorrected p = 0.98
A
B
C
D
E
Intermediate mon.
Non−Classical mon.
Neutrophils
CD66b− Neutrophils
log2(Intermediate mon. + 1)
log2(Non−Classical mon. + 1)
log2(Neutrophils + 1)
log2(CD66b− Neutrophils + 1)
0−12
13−24
25−36
37−48
49−60
Days post screening or symptom onset
Days post screening or symptoms onset

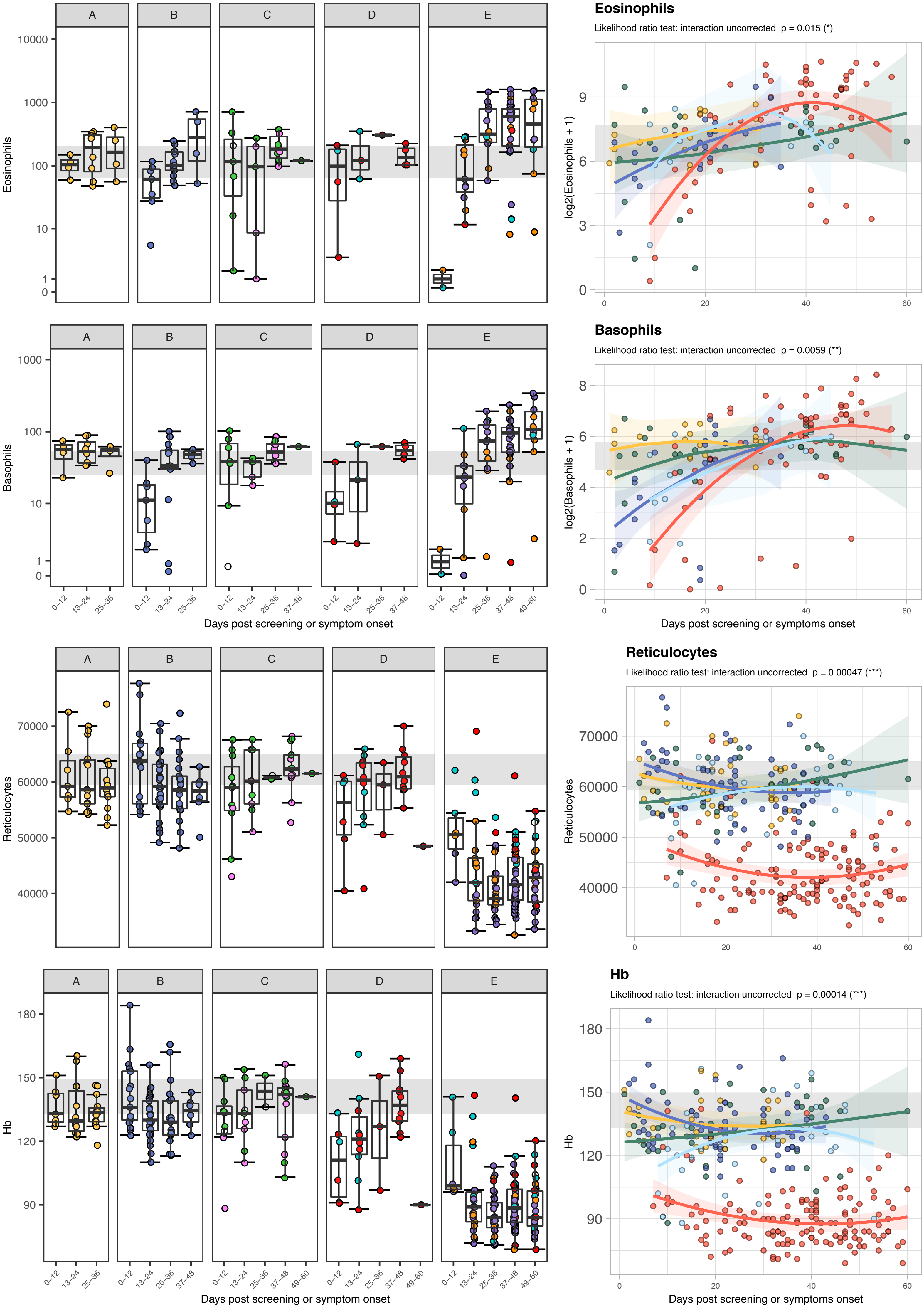

A
B
C
D
E
Eosinophils
Likelihood ratio test: interaction uncorrected p = 0.015 (*)
log2(Eosinophils + 1)
Basophils
Likelihood ratio test: interaction uncorrected p = 0.0059 (**)
log2(Basophils + 1)
Reticulocytes
Likelihood ratio test: interaction uncorrected p = 0.00047 (***)
Hb
Likelihood ratio test: interaction uncorrected p = 0.00014 (***)
0–12
13–24
25–36
37–48
49–60
Days post screening or symptom onset
Days post screening or symptoms onset

Data S3: Differences in serum cytokines, complement components and immunophenotyping between patients in cluster 1 and cluster 2 as defined in Figure 4A

A) Mixed-effect model with quadratic time trend showing the longitudinal trajectories of inflammatory markers, and boxplot at time of sampling (<=10 days post symptom onset), for individuals in clusters 1 and 2. **B)** Mixed-effect model with quadratic time trend showing the longitudinal trajectories for all cell populations over time, for COVID-19 cases in clusters 1 and 2. Grey bands in **A)** and **B)** indicate the interquartile range of the corresponding measurements in HCs. Nominal and adjusted p-values for the time x cluster interaction term are reported.

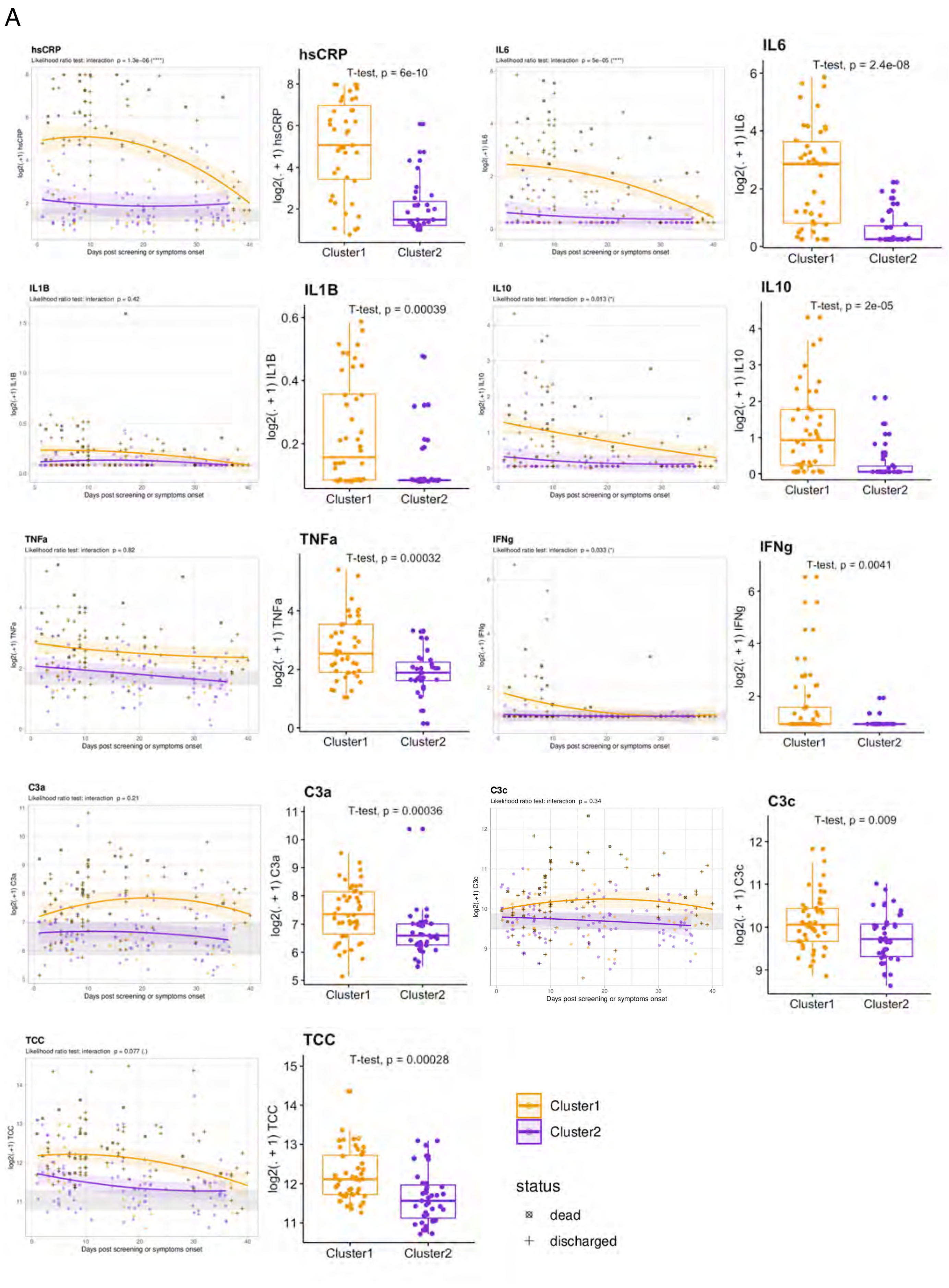
A
hsCRP
Likelihood ratio test: interaction p = 1.3e−06 (****)
T-test, p = 6e-10
log2(. + 1) hsCRP
IL6
Likelihood ratio test: interaction p = 5e−05 (****)
T-test, p = 2.4e-08
log2(. + 1) IL6
IL1B
Likelihood ratio test: interaction p = 0.42
T-test, p = 0.00039
log2(. + 1) IL1B
IL10
Likelihood ratio test: interaction p = 0.013 (*)
T-test, p = 2e-05
log2(. + 1) IL10
TNFa
Likelihood ratio test: interaction p = 0.82
T-test, p = 0.00032
log2(. + 1) TNFa
IFNg
Likelihood ratio test: interaction p = 0.033 (*)
T-test, p = 0.0041
log2(. + 1) IFNg
C3a
Likelihood ratio test: interaction p = 0.21
T-test, p = 0.00036
log2(. + 1) C3a
C3c
Likelihood ratio test: interaction p = 0.34
T-test, p = 0.009
log2(. + 1) C3c
TCC
Likelihood ratio test: interaction p = 0.077 (.)
T-test, p = 0.00028
log2(. + 1) TCC
Days post screening or symptoms onset
Cluster1
Cluster2
status
dead
discharged

B

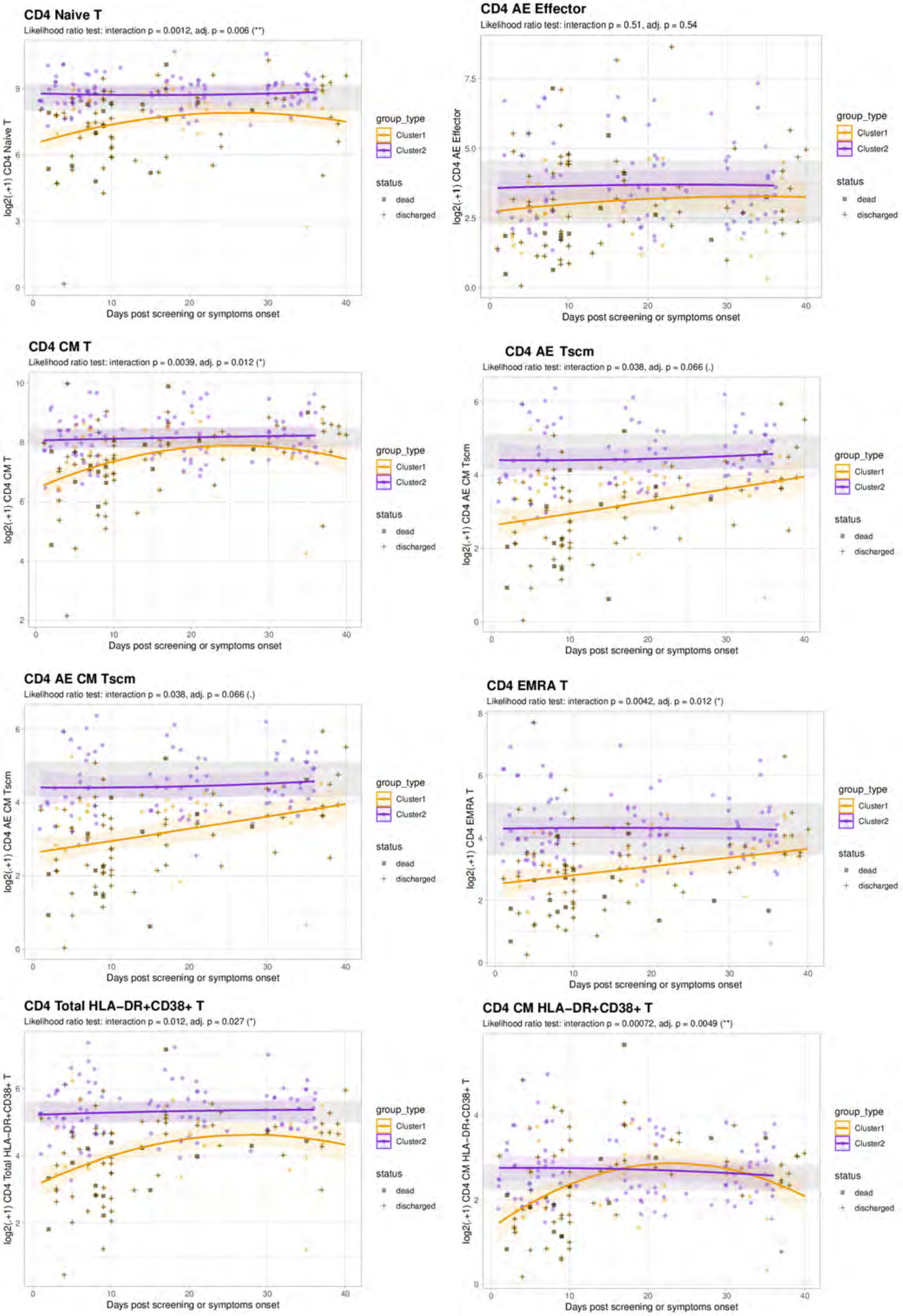
CD4 Naive T
Likelihood ratio test: interaction p = 0.0012, adj. p = 0.006 (**)
log2(.+1) CD4 Naive T
Days post screening or symptoms onset
CD4 AE Effector
Likelihood ratio test: interaction p = 0.51, adj. p = 0.54
log2(.+1) CD4 AE Effector
Days post screening or symptoms onset
CD4 CM T
Likelihood ratio test: interaction p = 0.0039, adj. p = 0.012 (*)
log2(.+1) CD4 CM T
Days post screening or symptoms onset
CD4 AE Tscm
Likelihood ratio test: interaction p = 0.038, adj. p = 0.066 (.)
log2(.+1) CD4 AE CM Tscm
Days post screening or symptoms onset
CD4 AE CM Tscm
Likelihood ratio test: interaction p = 0.038, adj. p = 0.066 (.)
log2(.+1) CD4 AE CM Tscm
Days post screening or symptoms onset
CD4 EMRA T
Likelihood ratio test: interaction p = 0.0042, adj. p = 0.012 (*)
log2(.+1) CD4 EMRA T
Days post screening or symptoms onset
CD4 Total HLA−DR+CD38+ T
Likelihood ratio test: interaction p = 0.012, adj. p = 0.027 (*)
log2(.+1) CD4 Total HLA−DR+CD38+ T
Days post screening or symptoms onset
CD4 CM HLA−DR+CD38+ T
Likelihood ratio test: interaction p = 0.00072, adj. p = 0.0049 (**)
log2(.+1) CD4 CM HLA−DR+CD38+ T
Days post screening or symptoms onset
group_type
Cluster1
Cluster2
status
dead
discharged

CD4 EM HLA−DR+CD38+ T

Likelihood ratio test: interaction p = 0.0017, adj. p = 0.0076 (**)

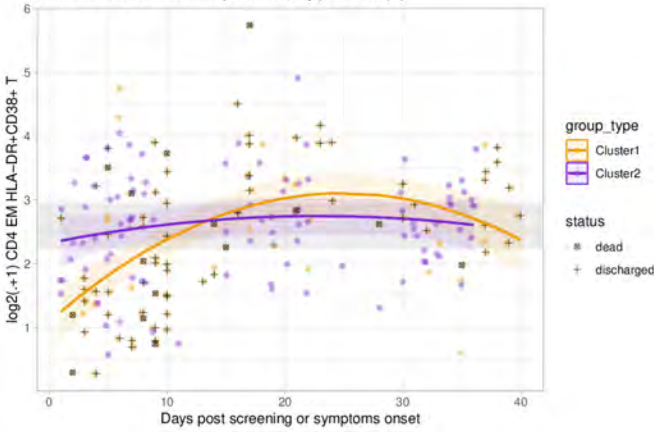

CD4 EMRA HLA−DR+CD38+ T

Likelihood ratio test: interaction p = 0.18, adj. p = 0.23

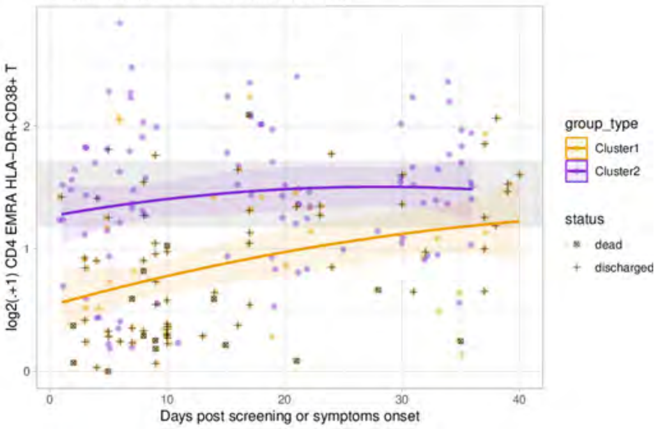

CD4 Non−naive HLA−DR+CD38+ T

Likelihood ratio test: interaction p = 0.00038, adj. p = 0.0038 (**)

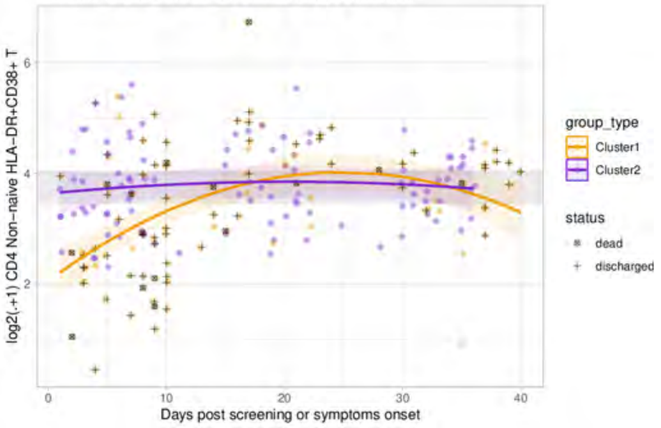

CD4 Tfh−like

Likelihood ratio test: interaction p = 0.018, adj. p = 0.035 (*)

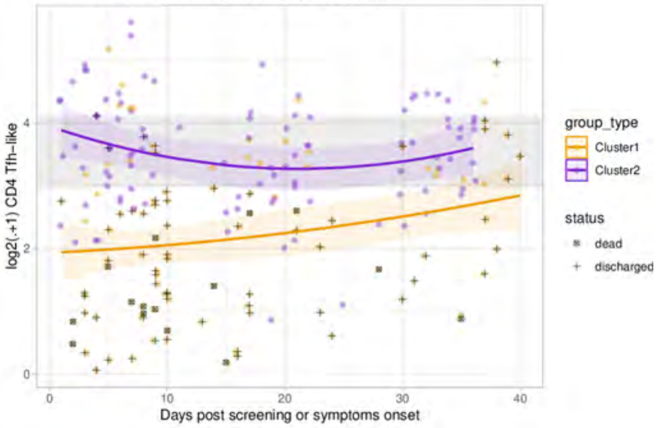

Treg

Likelihood ratio test: interaction p = 0.00025, adj. p = 0.0037 (**)

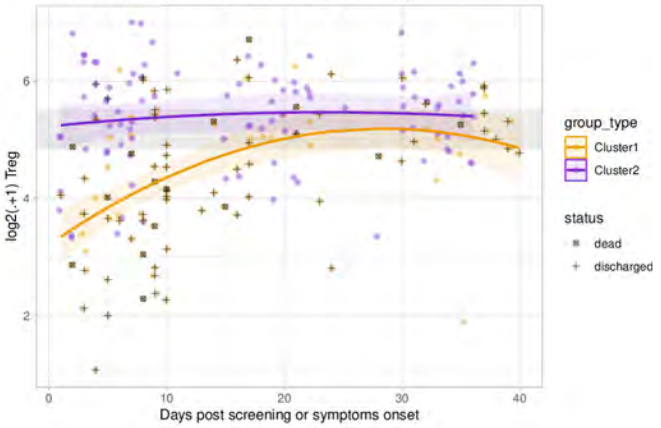

Treg HLA−DR+

Likelihood ratio test: interaction p = 0.002, adj. p = 0.0076 (**)

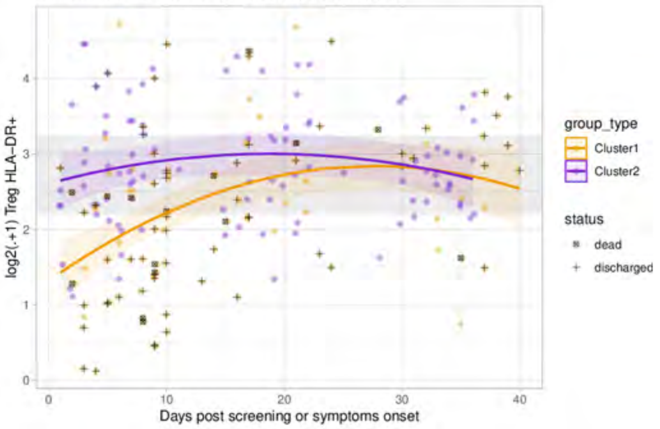

Treg Helios+

Likelihood ratio test: interaction p = 0.0018, adj. p = 0.0076 (**)

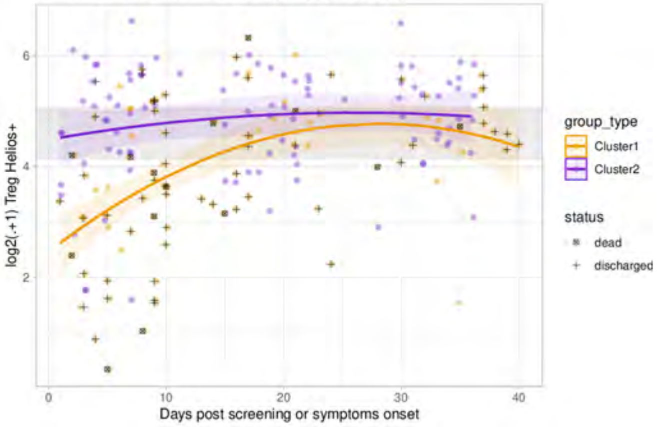

CD4 CCR6+

Likelihood ratio test: interaction p = 0.0062, adj. p = 0.016 (*)

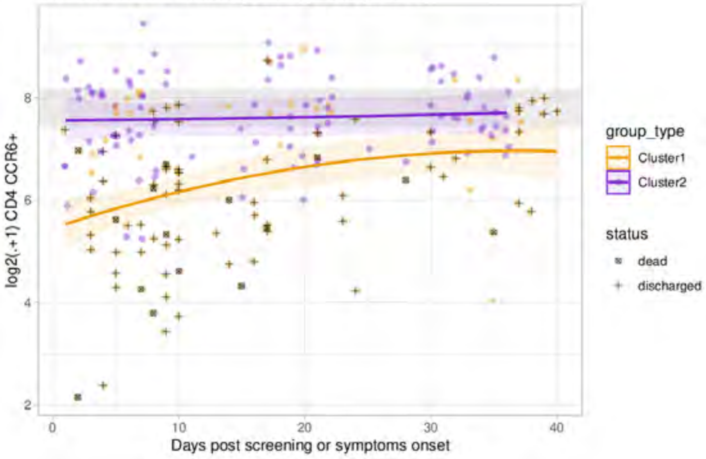

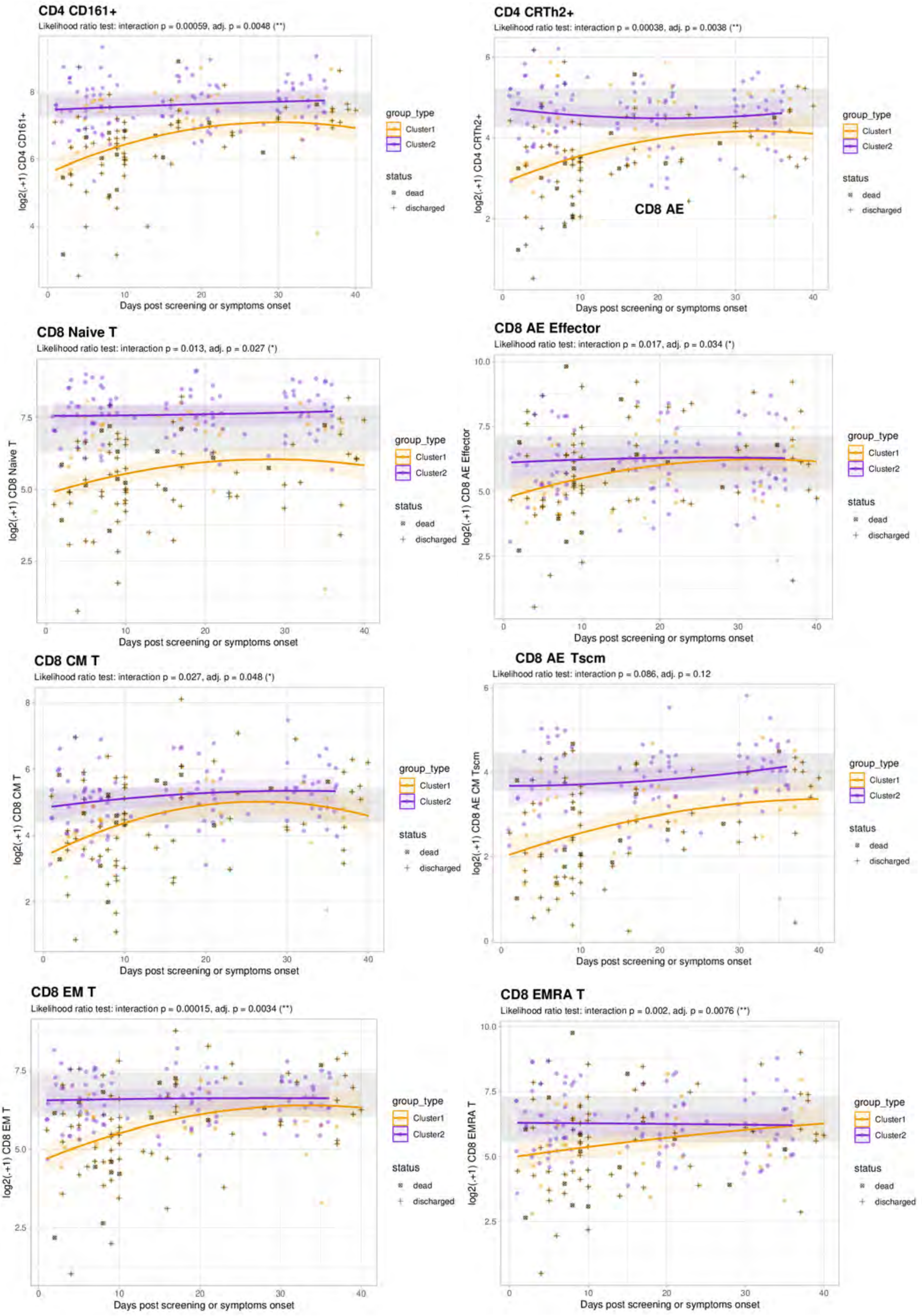
CD4 CD161+
Likelihood ratio test: interaction p = 0.00059, adj. p = 0.0048 (**)
log2(.+1) CD4 CD161+
Days post screening or symptoms onset
CD4 CRTh2+
Likelihood ratio test: interaction p = 0.00038, adj. p = 0.0038 (**)
log2(.+1) CD4 CRTh2+
CD8 AE
Days post screening or symptoms onset
CD8 Naive T
Likelihood ratio test: interaction p = 0.013, adj. p = 0.027 (*)
log2(.+1) CD8 Naive T
Days post screening or symptoms onset
CD8 AE Effector
Likelihood ratio test: interaction p = 0.017, adj. p = 0.034 (*)
log2(.+1) CD8 AE Effector
Days post screening or symptoms onset
CD8 CM T
Likelihood ratio test: interaction p = 0.027, adj. p = 0.048 (*)
log2(.+1) CD8 CM T
Days post screening or symptoms onset
CD8 AE Tscm
Likelihood ratio test: interaction p = 0.086, adj. p = 0.12
log2(.+1) CD8 AE CM Tscm
Days post screening or symptoms onset
CD8 EM T
Likelihood ratio test: interaction p = 0.00015, adj. p = 0.0034 (**)
log2(.+1) CD8 EM T
Days post screening or symptoms onset
CD8 EMRA T
Likelihood ratio test: interaction p = 0.002, adj. p = 0.0076 (**)
log2(.+1) CD8 EMRA T
Days post screening or symptoms onset
group_type
Cluster1
Cluster2
status
dead
discharged

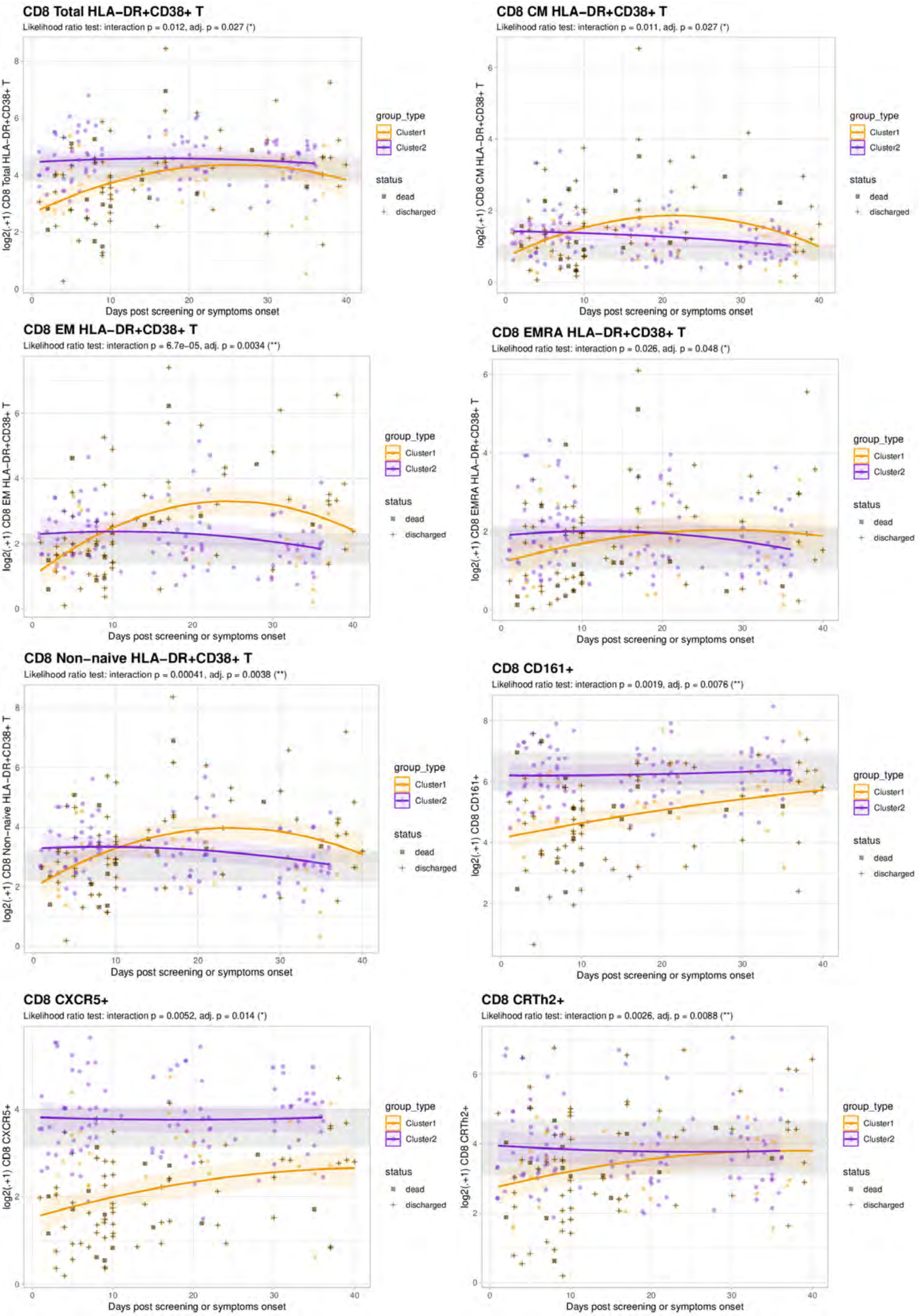
CD8 Total HLA−DR+CD38+ T
Likelihood ratio test: interaction p = 0.012, adj. p = 0.027 (*)
log2(.+1) CD8 Total HLA−DR+CD38+ T
Days post screening or symptoms onset
CD8 CM HLA−DR+CD38+ T
Likelihood ratio test: interaction p = 0.011, adj. p = 0.027 (*)
log2(.+1) CD8 CM HLA−DR+CD38+ T
Days post screening or symptoms onset
CD8 EM HLA−DR+CD38+ T
Likelihood ratio test: interaction p = 6.7e−05, adj. p = 0.0034 (**)
log2(.+1) CD8 EM HLA−DR+CD38+ T
Days post screening or symptoms onset
CD8 EMRA HLA−DR+CD38+ T
Likelihood ratio test: interaction p = 0.026, adj. p = 0.048 (*)
log2(.+1) CD8 EMRA HLA−DR+CD38+ T
Days post screening or symptoms onset
CD8 Non−naive HLA−DR+CD38+ T
Likelihood ratio test: interaction p = 0.00041, adj. p = 0.0038 (**)
log2(.+1) CD8 Non−naive HLA−DR+CD38+ T
Days post screening or symptoms onset
CD8 CD161+
Likelihood ratio test: interaction p = 0.0019, adj. p = 0.0076 (**)
log2(.+1) CD8 CD161+
Days post screening or symptoms onset
CD8 CXCR5+
Likelihood ratio test: interaction p = 0.0052, adj. p = 0.014 (*)
log2(.+1) CD8 CXCR5+
Days post screening or symptoms onset
CD8 CRTh2+
Likelihood ratio test: interaction p = 0.0026, adj. p = 0.0088 (**)
log2(.+1) CD8 CRTh2+
Days post screening or symptoms onset
group_type
Cluster1
Cluster2
status
dead
discharged

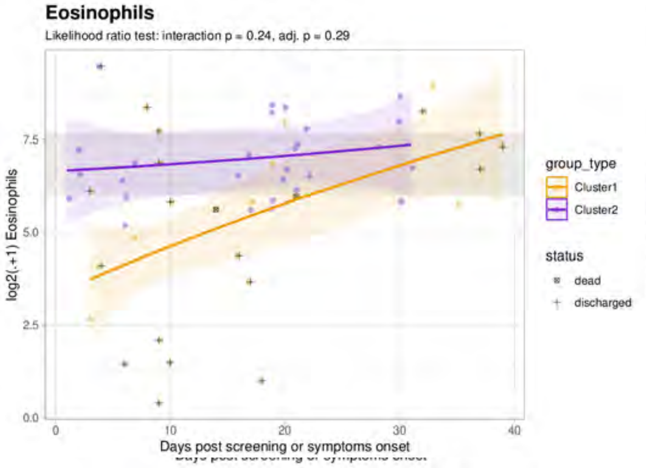
Eosinophils
Likelihood ratio test: interaction p = 0.24, adj. p = 0.29
log2(.+1) Eosinophils
Days post screening or symptoms onset
group_type
Cluster1
Cluster2
status
dead
discharged

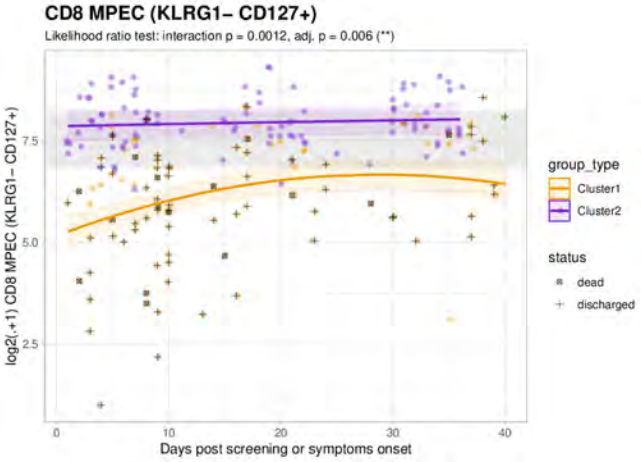
CD8 MPEC (KLRG1− CD127+)
Likelihood ratio test: interaction p = 0.0012, adj. p = 0.006 (**)
log2(.+1) CD8 MPEC (KLRG1− CD127+)
Days post screening or symptoms onset
group_type
Cluster1
Cluster2
status
dead
discharged

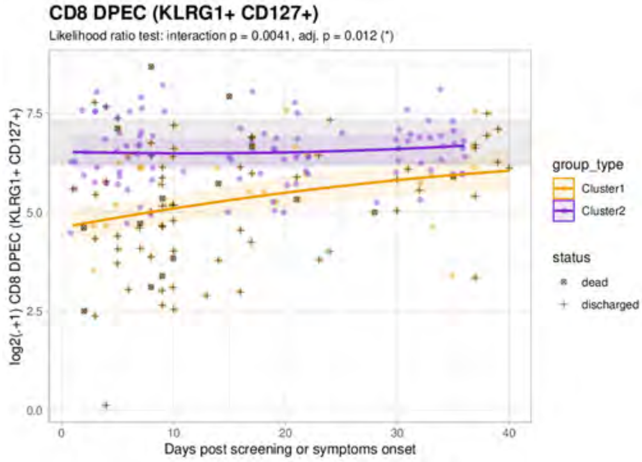
CD8 DPEC (KLRG1+ CD127+)
Likelihood ratio test: interaction p = 0.0041, adj. p = 0.012 (*)
log2(.+1) CD8 DPEC (KLRG1+ CD127+)
Days post screening or symptoms onset
group_type
Cluster1
Cluster2
status
dead
discharged

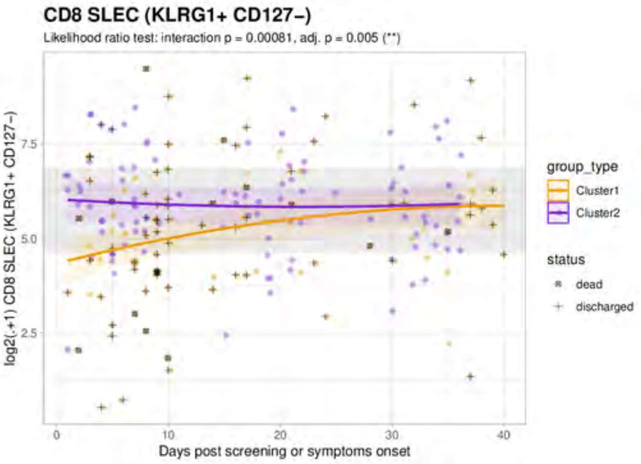
CD8 SLEC (KLRG1+ CD127−)
Likelihood ratio test: interaction p = 0.00081, adj. p = 0.005 (**)
log2(.+1) CD8 SLEC (KLRG1+ CD127−)
Days post screening or symptoms onset
group_type
Cluster1
Cluster2
status
dead
discharged

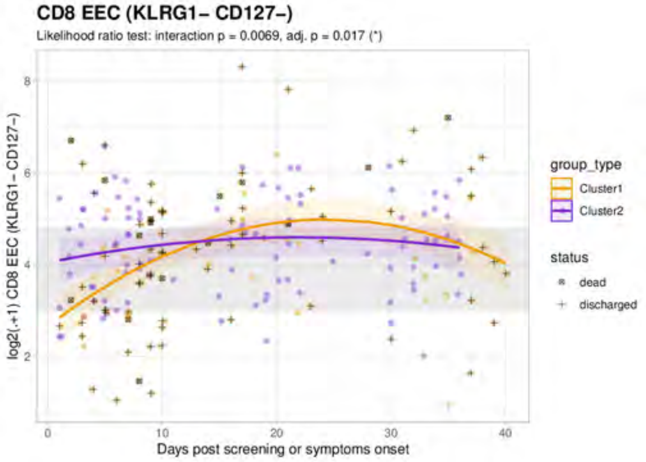
CD8 EEC (KLRG1− CD127−)
Likelihood ratio test: interaction p = 0.0069, adj. p = 0.017 (*)
log2(.+1) CD8 EEC (KLRG1− CD127−)
Days post screening or symptoms onset
group_type
Cluster1
Cluster2
status
dead
discharged

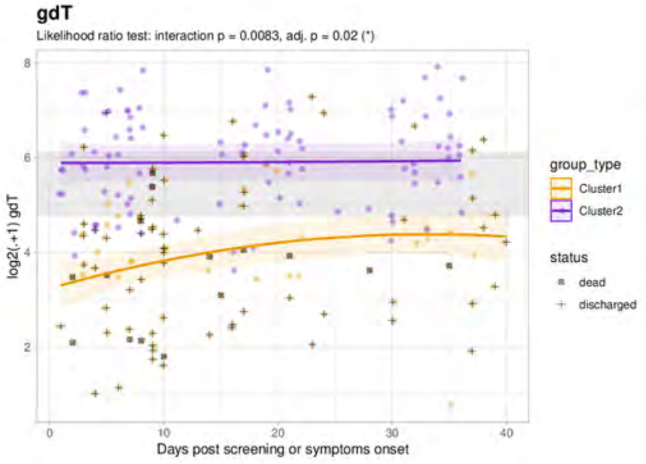
gdT
Likelihood ratio test: interaction p = 0.0083, adj. p = 0.02 (*)
log2(.+1) gdT
Days post screening or symptoms onset
group_type
Cluster1
Cluster2
status
dead
discharged

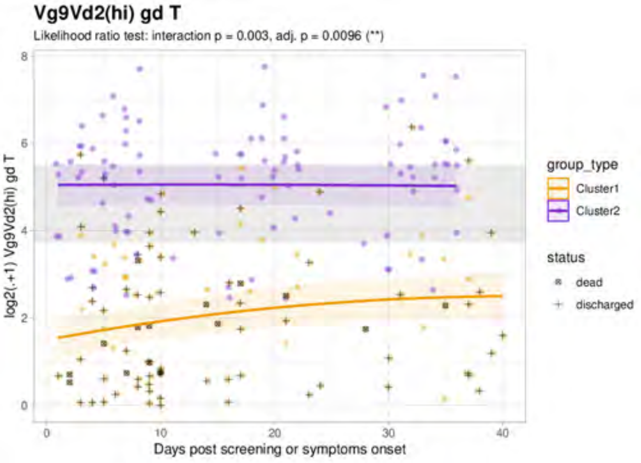
Vg9Vd2(hi) gd T
Likelihood ratio test: interaction p = 0.003, adj. p = 0.0096 (**)
log2(.+1) Vg9Vd2(hi) gd T
Days post screening or symptoms onset
group_type
Cluster1
Cluster2
status
dead
discharged

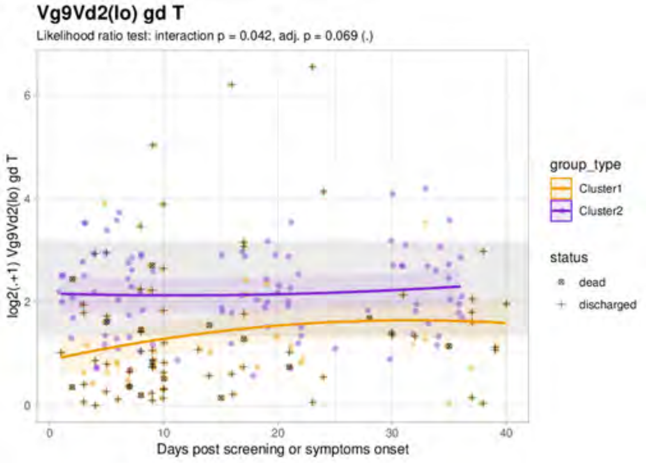
Vg9Vd2(lo) gd T
Likelihood ratio test: interaction p = 0.042, adj. p = 0.069 (.)
log2(.+1) Vg9Vd2(lo) gd T
Days post screening or symptoms onset
group_type
Cluster1
Cluster2
status
dead
discharged

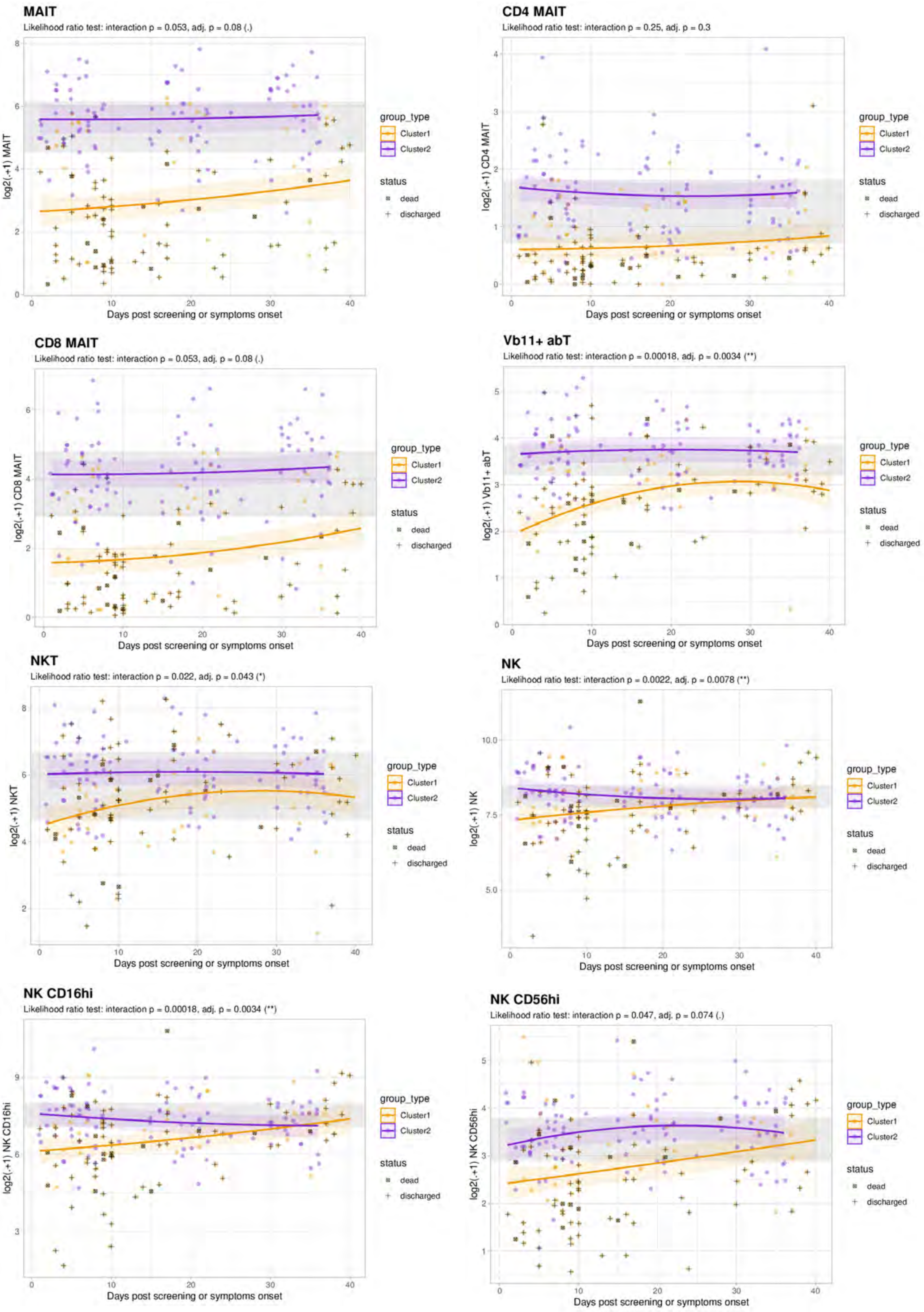
MAIT
Likelihood ratio test: interaction p = 0.053, adj. p = 0.08 (.)
log2(.+1) MAIT
Days post screening or symptoms onset
CD4 MAIT
Likelihood ratio test: interaction p = 0.25, adj. p = 0.3
log2(.+1) CD4 MAIT
Days post screening or symptoms onset
CD8 MAIT
Likelihood ratio test: interaction p = 0.053, adj. p = 0.08 (.)
log2(.+1) CD8 MAIT
Days post screening or symptoms onset
Vb11+ abT
Likelihood ratio test: interaction p = 0.00018, adj. p = 0.0034 (**)
log2(.+1) Vb11+ abT
Days post screening or symptoms onset
NKT
Likelihood ratio test: interaction p = 0.022, adj. p = 0.043 (*)
log2(.+1) NKT
Days post screening or symptoms onset
NK
Likelihood ratio test: interaction p = 0.0022, adj. p = 0.0078 (**)
log2(.+1) NK
Days post screening or symptoms onset
NK CD16hi
Likelihood ratio test: interaction p = 0.00018, adj. p = 0.0034 (**)
log2(.+1) NK CD16hi
Days post screening or symptoms onset
NK CD56hi
Likelihood ratio test: interaction p = 0.047, adj. p = 0.074 (.)
log2(.+1) NK CD56hi
Days post screening or symptoms onset
group_type
Cluster1
Cluster2
status
dead
discharged

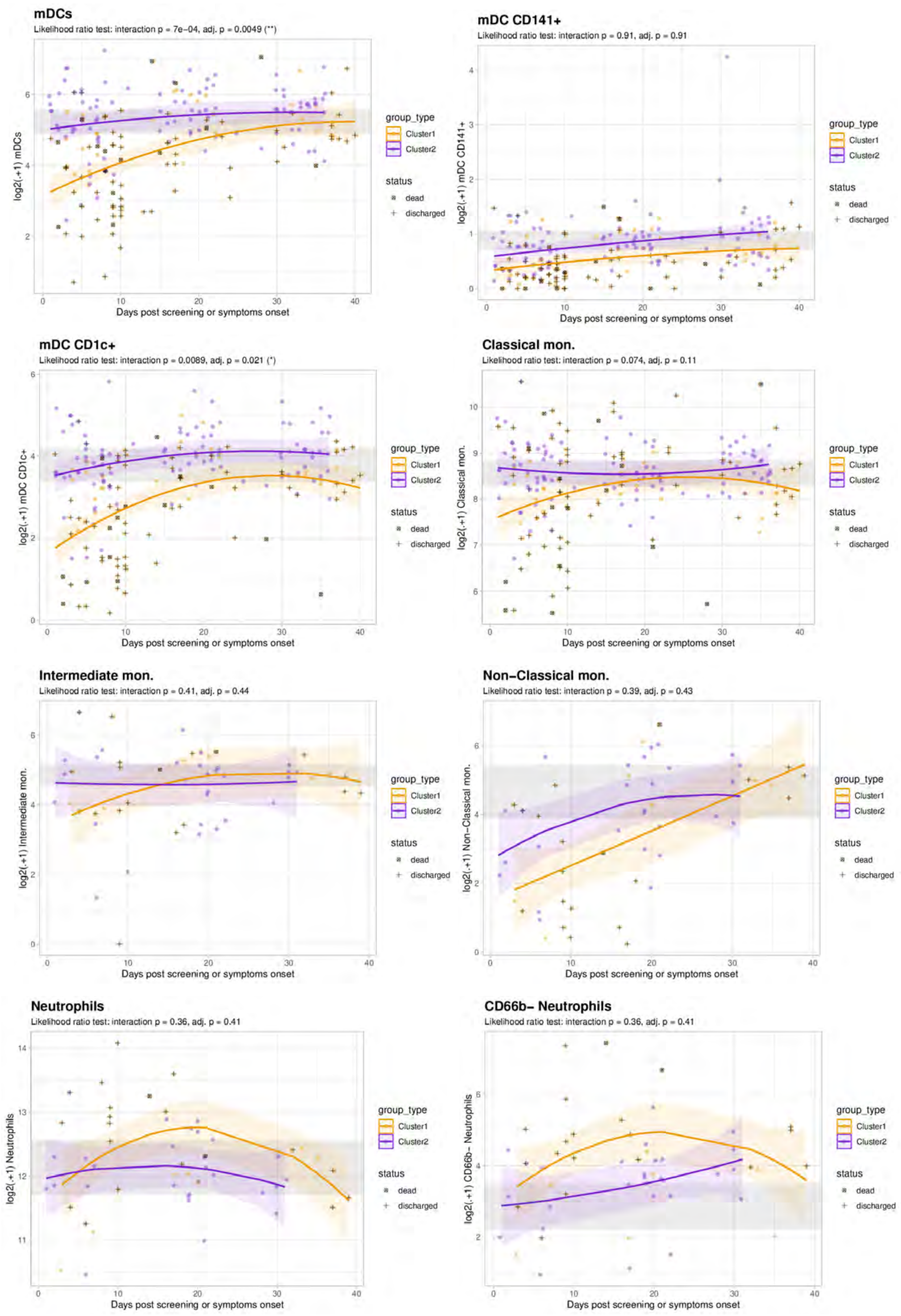
mDCs
Likelihood ratio test: interaction p = 7e−04, adj. p = 0.0049 (**)
log2(.+1) mDCs
Days post screening or symptoms onset
group_type
Cluster1
Cluster2
status
dead
discharged
mDC CD141+
Likelihood ratio test: interaction p = 0.91, adj. p = 0.91
log2(.+1) mDC CD141+
Days post screening or symptoms onset
mDC CD1c+
Likelihood ratio test: interaction p = 0.0089, adj. p = 0.021 (*)
log2(.+1) mDC CD1c+
Days post screening or symptoms onset
Classical mon.
Likelihood ratio test: interaction p = 0.074, adj. p = 0.11
log2(.+1) Classical mon.
Days post screening or symptoms onset
Intermediate mon.
Likelihood ratio test: interaction p = 0.41, adj. p = 0.44
log2(.+1) Intermediate mon.
Days post screening or symptoms onset
Non−Classical mon.
Likelihood ratio test: interaction p = 0.39, adj. p = 0.43
log2(.+1) Non−Classical mon.
Days post screening or symptoms onset
Neutrophils
Likelihood ratio test: interaction p = 0.36, adj. p = 0.41
log2(.+1) Neutrophils
Days post screening or symptoms onset
CD66b− Neutrophils
Likelihood ratio test: interaction p = 0.36, adj. p = 0.41
log2(.+1) CD66b− Neutrophils
Days post screening or symptoms onset

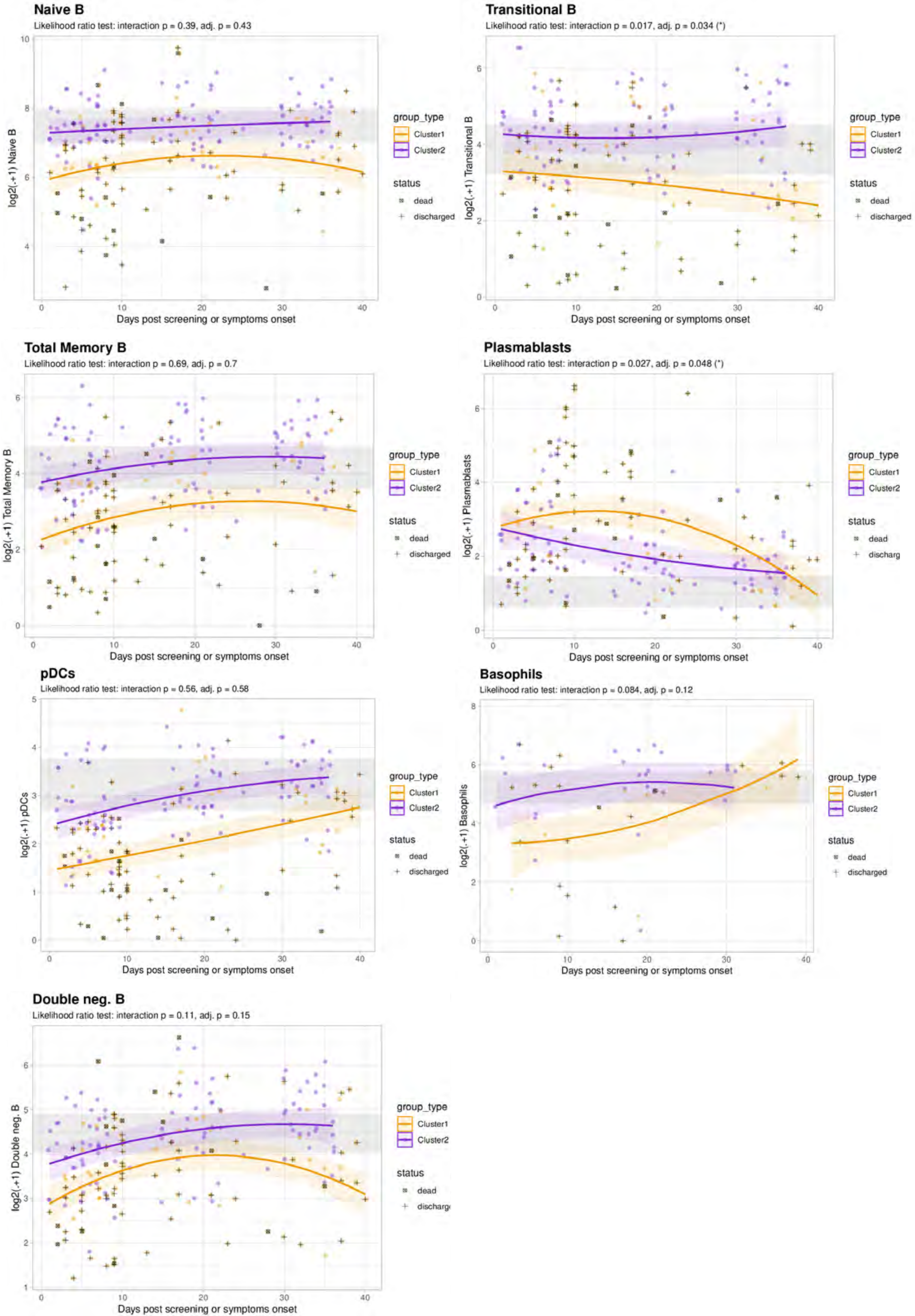
Naive B
Likelihood ratio test: interaction p = 0.39, adj. p = 0.43
log2(.+1) Naive B
Transitional B
Likelihood ratio test: interaction p = 0.017, adj. p = 0.034 (*)
log2(.+1) Transitional B
Total Memory B
Likelihood ratio test: interaction p = 0.69, adj. p = 0.7
log2(.+1) Total Memory B
Plasmablasts
Likelihood ratio test: interaction p = 0.027, adj. p = 0.048 (*)
log2(.+1) Plasmablasts
pDCs
Likelihood ratio test: interaction p = 0.56, adj. p = 0.58
log2(.+1) pDCs
Basophils
Likelihood ratio test: interaction p = 0.084, adj. p = 0.12
log2(.+1) Basophils
Double neg. B
Likelihood ratio test: interaction p = 0.11, adj. p = 0.15
log2(.+1) Double neg. B
Days post screening or symptoms onset
group_type
Cluster1
Cluster2
status
dead
discharged

Data S4: Flow cytometry gating strategy, related to STAR Methods

A) B cell, **B)** non-conventional T cell, **C)** DCs and monocyte, **D)** T regulatory cells, and **E)** conventional T cell panels.

A

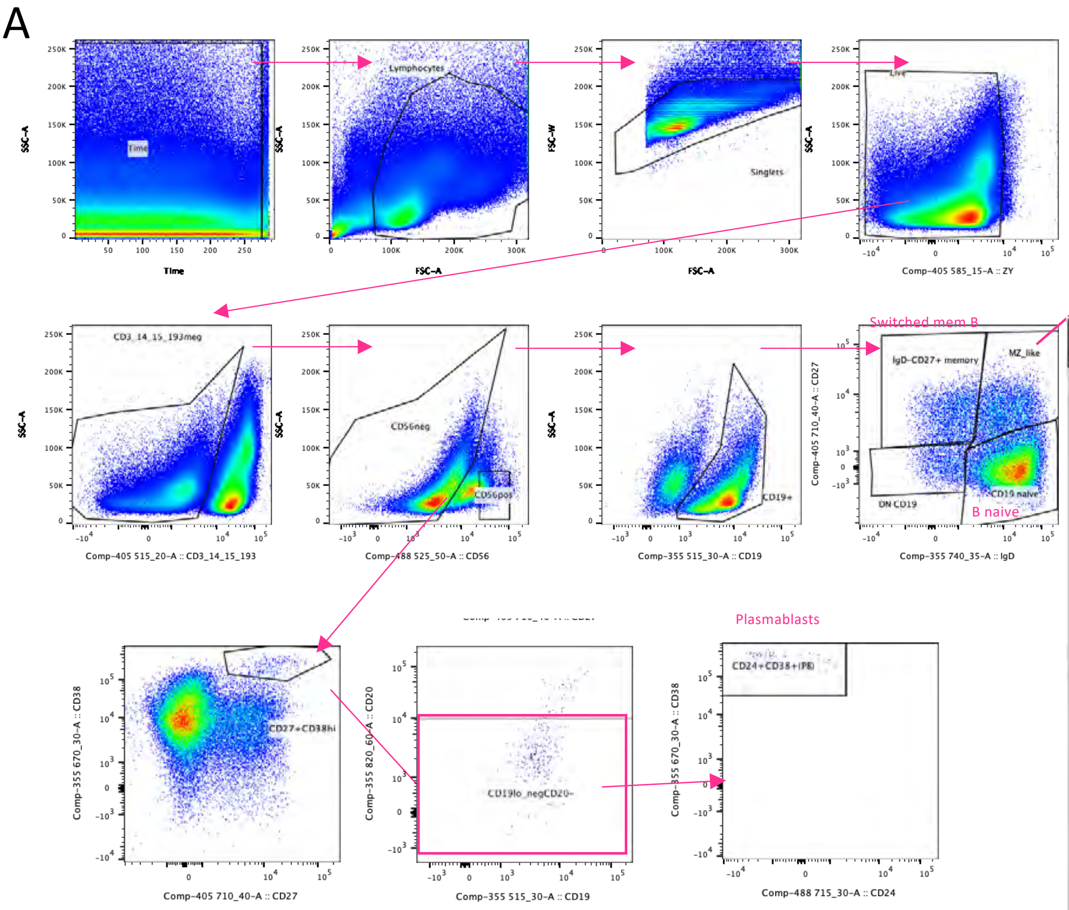

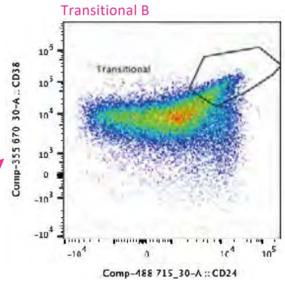

Panel	Laser	BPF	Antigen	Fluorophore
B cells	355	379/28		
		515/30	CD19	BUV496
		560/40		
		670/30	CD38	BUV661
		740/35		
		820/60	CD20	BUV805
	405	450/50	CD39	BV421
		515/20	CD3, CD14, CD15, CD193	BV510
		585/15	Zombie Yellow	
		605/40	HLADR	BV605
		670/30	CD71	BV650
		710/40	CD27	BV711
		741/40		
		780/60	CD73	BV785
	488	525/50	CD56	FITC
		610/20		
		685/35		
		715/30	CD24	BB700
		780/60		
	561	585/15		
		610/20		
		670/30	CD80	PECy5
		710/40		
		780/60	CD86	PECy7
	640	670/30	GLUT1	AF647
		730/45	CXCR5	APC-R700
		780/60		

B

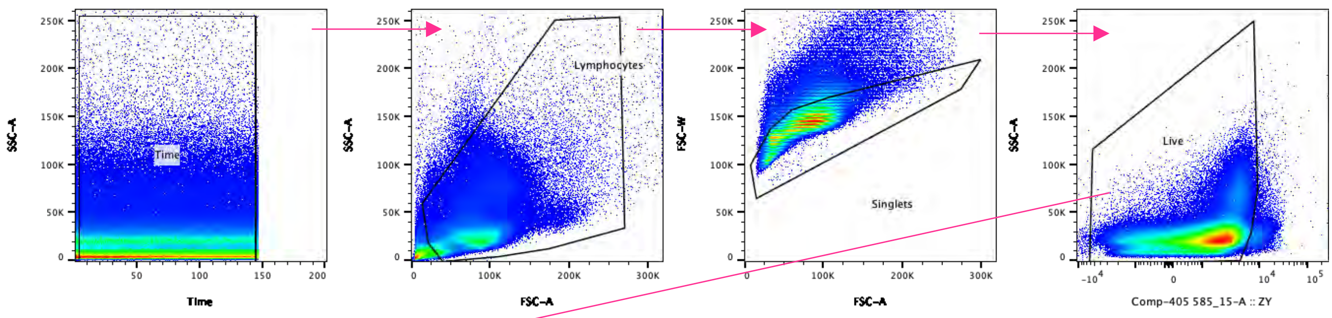

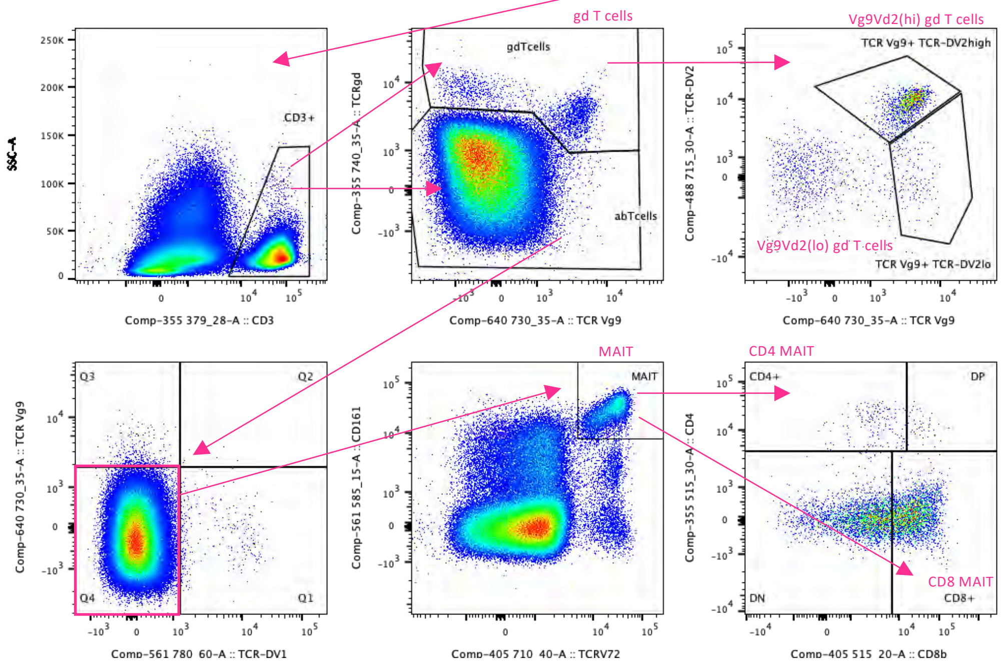

Panel	Laser	BPF	Antigen	Fluorophore
non-conventional T cells	355	379/28	CD3	BUV395
		515/30	CD4	BUV496
		560/40		
		670/30		
		740/35	TCRgd	BUV737
		820/60	CD28	BUV805
	405	450/50	PD-1	BV421
		515/20	CD8b	BV480
		585/15	Zombie Yellow	
		605/40	HLADR	BV605
		670/30	CD69	BV650
		710/40	TCRV7.2	BV711
		741/40		
		780/60		
	488	525/50	KLRG1	FITC
		610/20		
		685/35		
		715/30	TCR-DV2	PerCPCy5.5
		780/60		
	561	585/15	CD161	PE
		610/20		
		670/30	CD127	PECy5
		710/40		
		780/60	TCR-DV1	PECy7
	640	670/30	CCR5	AF647
		730/45	TCR Vγ9	AF700
		780/60	Vb11	APCVio770

C

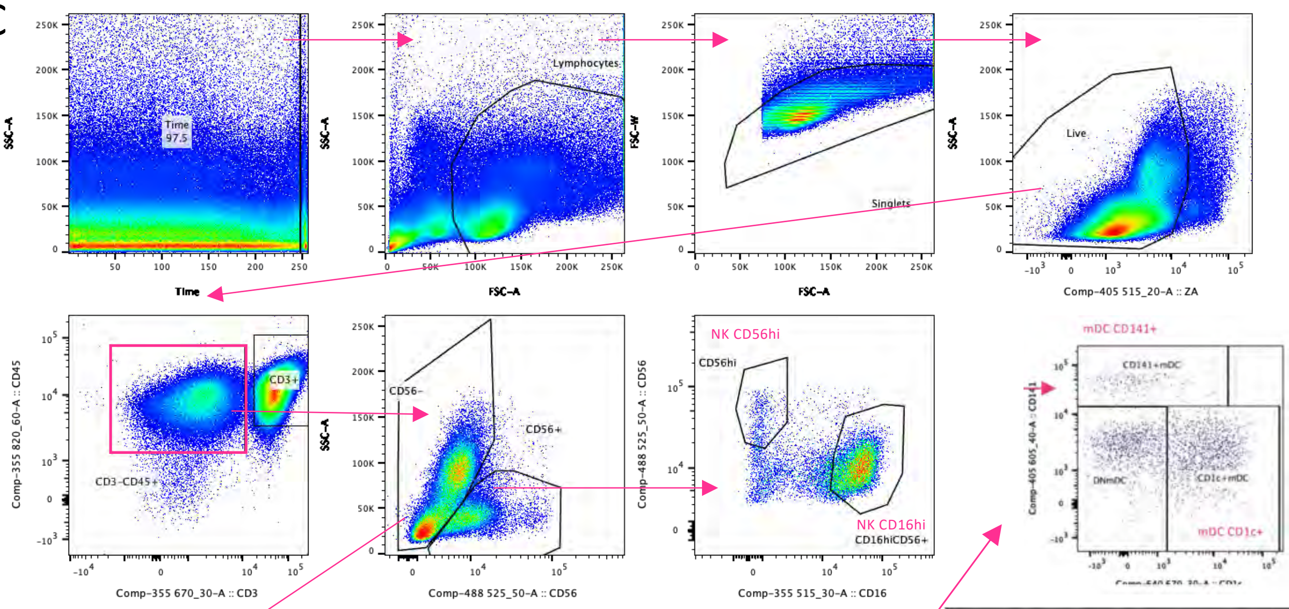

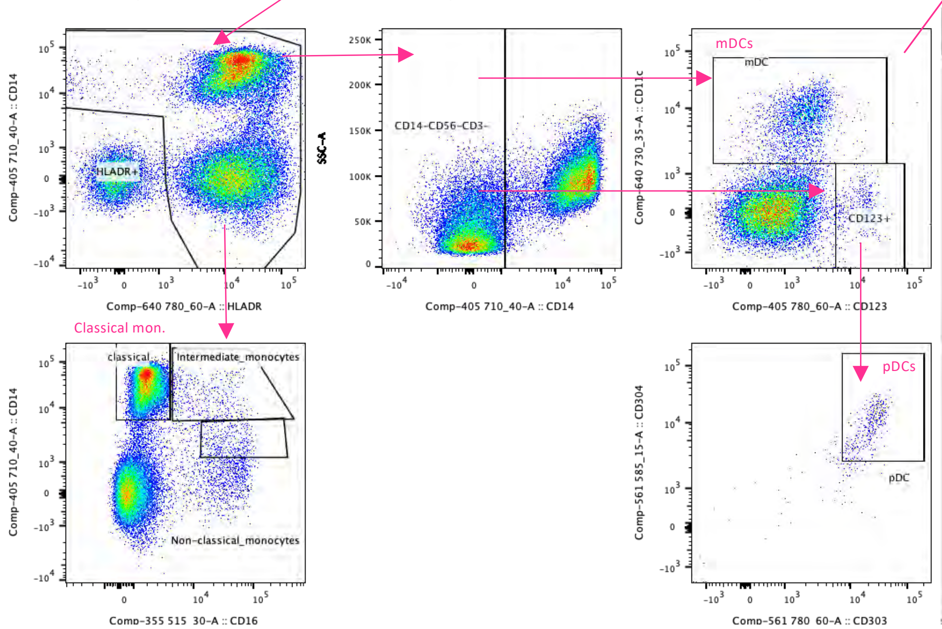

Panel	Laser	BPF	Antigen	Fluorophore
DC's and monocytes	355	379/28	CD40	BUV395
		515/30	CD16	BUV496
		560/40		
		670/30	CD3	BUV661
		740/35	CD86	BUV737
		820/60	CD45	BUV805
	405	450/50		
		515/20	Zombie Aqua	
		585/15	CD45RA	BV570
		605/40	CD141	BV605
		670/30	CCR7	BV650
		710/40	CD14	BV711
		741/40		
		780/60	CD123	BV786
	488	525/50	CD56	FITC
		610/20		
		685/35		
		715/30	CD32	BB700
		780/60		
	561	585/15	CD304	PE
		610/20	CD163	PE-CF594
		670/30	CD80	PECy5
		710/40		
		780/60	CD303	PE-Vio770
	640	670/30	CD1c	AF647
		730/45	CD11c	AF700
		780/60	HLA-DR	APC-H7

D

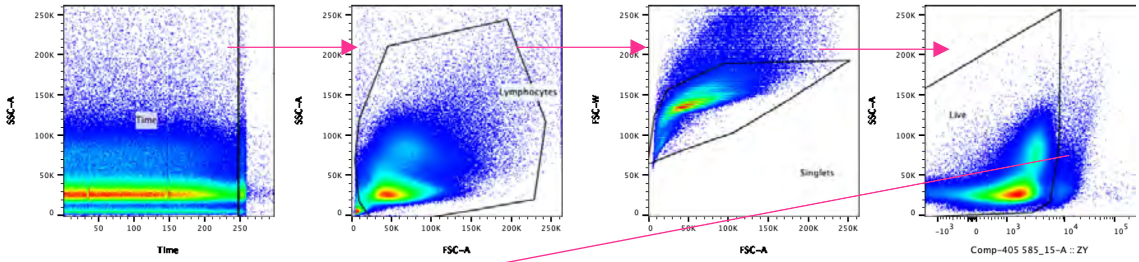

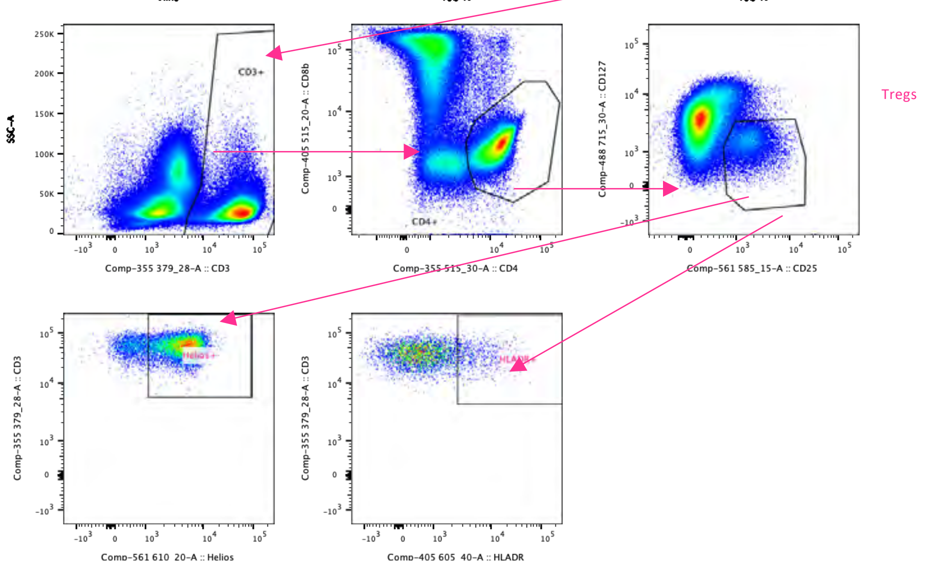

Panel	Laser	BPF	Antigen	Fluorophore
T regulatory cells	355	379/28	CD3	BUV395
		515/30	CD4	BUV496
		560/40		
		670/30		
		740/35		
		820/60	CD45RA	BUV805
	405	450/50	PD-1	BV421
		515/20	CD8b	BV480
		585/15	Zombie Yellow	
		605/40	HLADR	BV605
		670/30	CCR7	BV650
		710/40		
		741/40		
		780/60	CD73	Brilliant Violet 785™
	488	525/50		
		610/20		
		685/35		
		715/30	CD127	PerCP efluor710
		780/60		
	561	585/15	CD25	PE
		610/20	Helios	Pedazzle
		670/30		
		710/40		
		780/60	CCR4	PEVio770
	640	670/30	FoxP3	APC
		730/45	CXCR5	APC-R700
		780/60	CD39	APC-fire

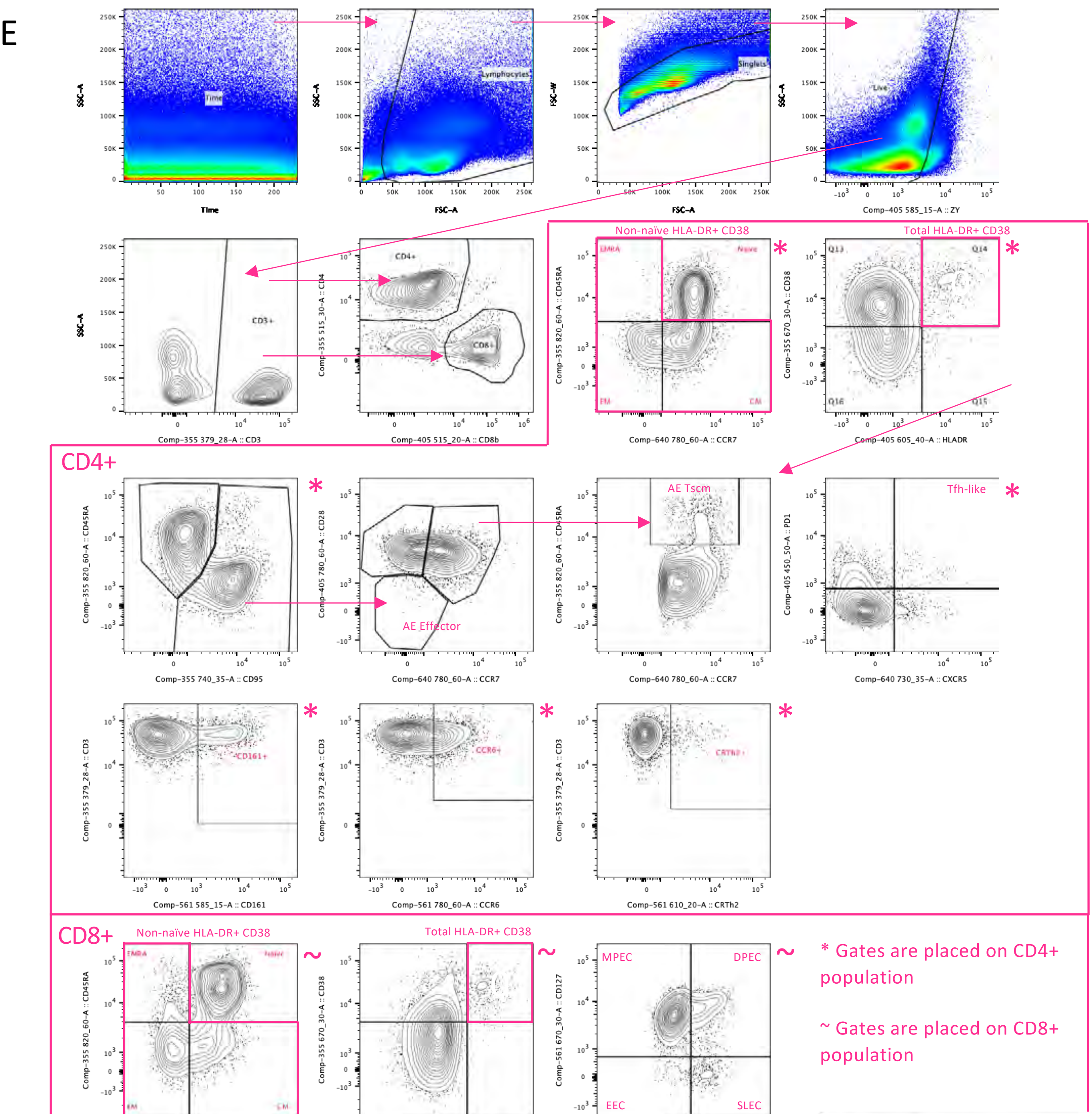

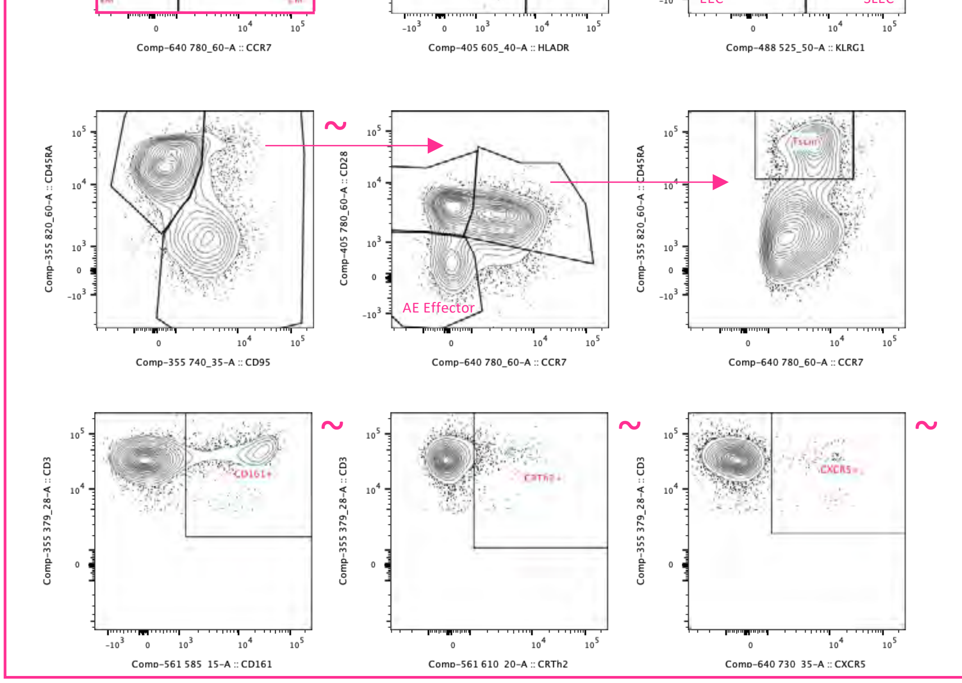

Panel	Laser	BPF	Antigen	Fluorophore
conventional T cells	355	379/28	CD3	BUV395
		515/30	CD4	BUV496
		560/40		
		670/30	CD38	BUV661
		740/35	CD95	BUV737
		820/60	CD45RA	BUV805
	405	450/50	PD-1	BV421
		515/20	CD8b	BV480
		585/15		Zombie Yellow
		605/40	HLADR	BV605
		670/30	CD69	BV650
		710/40	CD27	BV711
		741/40		
		780/60	CD28	BV785
	488	525/50	KLRG1	FITC
		610/20		
		685/35		
		715/30		
		780/60		
	561	585/15	CD161	PE
		610/20	CRTh2	PE-dazzle
		670/30	CD127	PECy5
		710/40		
		780/60	CCR6	PECy7
	640	670/30		
		730/45	CXCR5	APC-R700
		780/60	CCR7	APC-fire
