# Supplementary material for: Longitudinal analysis reveals that delayed bystander CD8+ T cell activation and early immune pathology distinguish severe COVID-19 from mild disease
Source: Immunity. 2021 Jun 8;54(6):1257–1275.e8. doi: 10.1016/j.immuni.2021.05.010 (PMC8125900; doi:10.1016/j.immuni.2021.05.010)
Supplement: Document S2. Article plus supplemental information [file mmc3.pdf]

Immunity

Article

Longitudinal analysis reveals that delayed bystander CD8+ T cell activation and early immune pathology distinguish severe COVID-19 from mild disease

Graphical Abstract

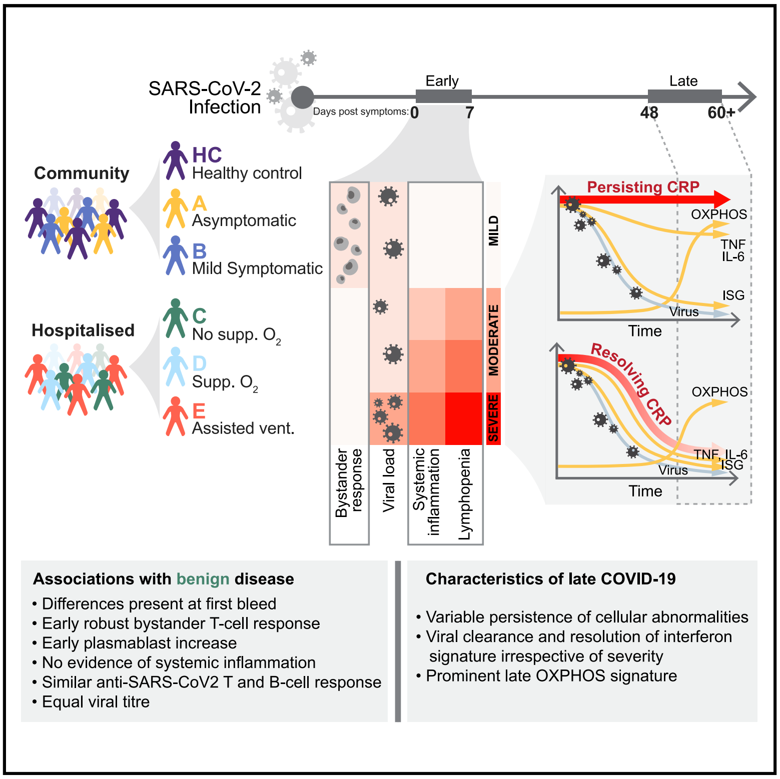

Authors

Laura Bergamaschi, Federica Mescia, Lorinda Turner, ..., John R. Bradley, Paul A. Lyons, Kenneth G.C. Smith

Correspondence

pal34@cam.ac.uk (P.A.L.),
kgcs2@cam.ac.uk (K.G.C.S.)

In brief

The immune changes that underlie COVID-19 severity have not been fully defined. By analyzing a longitudinal cohort of COVID-19 patients and integrating inflammatory factors, immunophenotyping, and transcriptome data, Bergamaschi et al. identify both early and persistent immune changes that distinguish mild and/or asymptomatic from more severe disease.

Highlights

- Longitudinal analysis of COVID-19 patients with a range of disease severity
- Early bystander CD8$^+$ T cell and plasmablast responses characterize mild disease
- Pronounced systemic inflammation evident at first presentation in more severe COVID-19
- Immune/inflammatory abnormalities persist in severe disease to 60 days post symptoms

Bergamaschi et al., 2021, Immunity *54*, 1257–1275
June 8, 2021 Crown Copyright © 2021 Published by Elsevier Inc.
https://doi.org/10.1016/j.immuni.2021.05.010

CellPress

Immunity

CellPress
OPEN ACCESS

Article

Longitudinal analysis reveals that delayed bystander CD8+ T cell activation and early immune pathology distinguish severe COVID-19 from mild disease

Laura Bergamaschi,[1,2,20] Federica Mescia,[1,2,20] Lorinda Turner,[1,2,20] Aimee L. Hanson,[1,2,20] Prasanti Kotagiri,[1,2,20] Benjamin J. Dunmore,[2] Hélène Ruffieux,[3] Aloka De Sa,[1,2] Oisín Huhn,[2] Michael D. Morgan,[4,5] Pehuén Pereyra Gerber,[1,2] Mark R. Wills,[1,2] Stephen Baker,[1,2] Fernando J. Calero-Nieto,[6] Rainer Doffinger,[7] Gordon Dougan,[1,2] Anne Elmer,[8] Ian G. Goodfellow,[9] Ravindra K. Gupta,[1,2] Myra Hosmillo,[9] Kelvin Hunter,[1,2] Nathalie Kingston,[10,11] Paul J. Lehner,[1,2] Nicholas J. Matheson,[1,2,12] Jeremy K. Nicholson,[13] Anna M. Petrunkina,[1,2] Sylvia Richardson,[3] Caroline Saunders,[8] James E.D. Thaventhiran,[1,2,14] Erik J.M. Toonen,[15] Michael P. Weekes,[1] Cambridge Institute of Therapeutic Immunology and Infectious Disease-National Institute of Health Research (CITIID-NIHR) COVID BioResource Collaboration, Berthold Göttgens,[6] Mark Toshner,[2,16,17] Christoph Hess,[1,2,18,19] John R. Bradley,[2,11] Paul A. Lyons,[1,2,*] and Kenneth G.C. Smith[1,2,21,*]

[1]Cambridge Institute of Therapeutic Immunology and Infectious Disease, Jeffrey Cheah Biomedical Centre, University of Cambridge, Cambridge CB2 0AW, UK
[2]Department of Medicine, University of Cambridge, Addenbrooke's Hospital, Cambridge CB2 0QQ, UK
[3]MRC Biostatistics Unit, University of Cambridge, Cambridge Biomedical Campus, Cambridge CB2 0SR, UK
[4]Cancer Research UK – Cambridge Institute, Robinson Way, Cambridge, CB2 0RE, UK
[5]European Molecular Biology Laboratory, European Bioinformatics Institute, Wellcome Genome Campus, Cambridge, UK
[6]Department of Haematology, Wellcome & MRC Cambridge Stem Cell Institute, University of Cambridge, Cambridge CB2 0AW, UK
[7]Department of Clinical Biochemistry and Immunology, Addenbrooke's Hospital, Cambridge CB2 0QQ, UK
[8]Cambridge Clinical Research Centre, NIHR Clinical Research Facility, Cambridge University Hospitals NHS Foundation Trust, Addenbrooke's Hospital, Cambridge CB2 0QQ, UK
[9]Division of Virology, Department of Pathology, University of Cambridge, Addenbrooke's Hospital, Cambridge CB2 0QQ, UK
[10]Department of Haematology, University of Cambridge, Cambridge Biomedical Campus, Cambridge CB2 0QQ, UK
[11]NIHR BioResource, Cambridge University Hospitals NHS Foundation, Cambridge Biomedical Campus, Cambridge CB2 0QQ, UK
[12]NHS Blood and Transplant, Cambridge, UK
[13]The Australian National Phenome Centre, Centre for Computational and Systems Medicine, Health Futures Institute, Murdoch University, Murdoch, Western Australia WA 6150, Australia
[14]MRC Toxicology Unit, School of Biological Sciences, University of Cambridge, Cambridge CB2 1QR, UK
[15]R&D Department, Hycult Biotech, 5405 PD Uden, the Netherlands
[16]Heart and Lung Research Institute, Cambridge Biomedical Campus, Cambridge CB2 0QQ, UK
[17]Royal Papworth Hospital NHS Foundation Trust, Cambridge Biomedical Campus, Cambridge CB2 0QQ, UK
[18]Department of Biomedicine, University and University Hospital Basel, 4031 Basel, Switzerland
[19]Botnar Research Centre for Child Health (BRCCH) University Basel & ETH Zurich, 4058 Basel, Switzerland
[20]These authors contributed equally
[21]Lead contact
*Correspondence: pal34@cam.ac.uk (P.A.L.), kgcs2@cam.ac.uk (K.G.C.S.)
https://doi.org/10.1016/j.immuni.2021.05.010

SUMMARY

The kinetics of the immune changes in COVID-19 across severity groups have not been rigorously assessed. Using immunophenotyping, RNA sequencing, and serum cytokine analysis, we analyzed serial samples from 207 SARS-CoV2-infected individuals with a range of disease severities over 12 weeks from symptom onset. An early robust bystander CD8$^+$ T cell immune response, without systemic inflammation, characterized asymptomatic or mild disease. Hospitalized individuals had delayed bystander responses and systemic inflammation that was already evident near symptom onset, indicating that immunopathology may be inevitable in some individuals. Viral load did not correlate with this early pathological response but did correlate with subsequent disease severity. Immune recovery is complex, with profound persistent cellular abnormalities in severe disease correlating with altered inflammatory responses, with signatures associated with increased oxidative phosphorylation replacing those driven by cytokines tumor necrosis factor (TNF) and interleukin (IL)-6. These late immunometabolic and immune defects may have clinical implications.

Crown Copyright © 2021 Published by Elsevier Inc.
This is an open access article under the CC BY license (http://creativecommons.org/licenses/by/4.0/).

INTRODUCTION

The immune pathology associated with COVID-19 is complex (Wang et al., 2020; Zhou et al., 2020). Most infected individuals mount a successful anti-viral response, resulting in few, if any, symptoms. In a minority of patients, there is evidence that ongoing cytokine production develops, associated with persistent systemic inflammation, end-organ damage, and often death (Del Valle et al., 2020; Lucas et al., 2020). The relationship between the initial immune response to SARS-CoV-2, viral clearance, and development of the ongoing inflammatory disease that drives severe COVID-19 is not clearly established, nor have the kinetics of the immune changes seen in COVID-19 been fully assessed as disease progresses. Defective immune recovery might drive ongoing disease and perhaps contribute to secondary immunodeficiency with an increased risk of subsequent infection.

Severe COVID-19 is associated with profound abnormalities in circulating immune cell subsets. There is a decrease in many peripheral blood subsets of both CD4^{+} and CD8^{+} T cells but an increase in activated and differentiated effector cells (Arunachalam et al., 2020; Hadjadj et al., 2020; Kuri-Cervantes et al., 2020; Laing et al., 2020; Mann et al., 2020; Mathew et al., 2020; Su et al., 2020). Cells expressing programmed cell death protein-1 (PD1) and other inhibitory molecules are increased, though whether these reflect genuine T cell exhaustion or changes accompanying T cell activation has not been firmly established. There is, nonetheless, evidence of functional impairment in both CD8^{+} and CD4^{+} T cells in a number of studies (Chen and John Wherry, 2020). Data on T helper (Th) cell subsets are variable, but there is evidence of increased Th17 cells and markedly reduced T follicular helper (Tfh) cells (Chen and John Wherry, 2020; Mathew et al., 2020; Su et al., 2020). There have been conflicting reports regarding B cell immunity, but increased circulating plasmablasts (Arunachalam et al., 2020; Hadjadj et al., 2020; Laing et al., 2020; Mathew et al., 2020) and reduced germinal center responses (Su et al., 2020) are consistently observed in severe COVID-19. Innate T cell subsets, including gamma-delta (gd) T cells and mucosal-associated invariant T (MAIT) cells, are also reduced, as are non-classical monocytes and both plasmacytoid and myeloid dendritic cells (pDCs and mDCs) (Kuri-Cervantes et al., 2020; Laing et al., 2020)

By analyzing transcriptome, serum cytokine, and immunophenotyping data of longitudinal samples from COVID-19 patients with a range of disease severities, for up to 3 months from symptom onset, we were able to address two important questions regarding the immune response to SARS-CoV-2. (1) How does the very early immune response in patients who cleared the virus and recovered from disease with few or no symptoms compare with those who progressed to severe inflammatory disease? This provided insight into the features of the initial immune response that correlate with severe inflammatory outcomes and whether systemic inflammation is an early or later development in those who progress to severe disease. (2) How rapidly do the profound immune defects that accompany severe COVID-19 recover, and do the kinetics of recovery relate to ongoing inflammation and clinical status?

We recruited 207 patients with COVID-19, ranging from asymptomatic healthcare workers in whom SARS-CoV-2 was detected on routine screening through to patients requiring assisted ventilation, and compared their results to 45 healthy controls. We performed detailed immune phenotyping at multiple time points up to 90 days from symptom onset, reporting absolute cell counts rather than proportions. All data have been made available at https://www.covid19cellatlas.org/patient/citiid/. We found that the immune response in patients with progressive COVID-19 was delayed compared to those with mild disease and was inflammatory in nature from the outset. Early immune cellular changes predicted severe disease course, and the variable recovery of these cells over 3 months is associated with marked changes in the nature of the systemic inflammation seen in severe COVID-19.

RESULTS

Patient cohorts

SARS-CoV-2 PCR-positive subjects were recruited for this study between 31st March and 20th July 2020. Those without symptoms, or with mild symptoms, were recruited from routine screening of healthcare workers (HCWs) (Rivett et al., 2020). COVID-19 patients were recruited at or soon after admission to Addenbrooke's or Royal Papworth hospitals.

Study participants were divided into five categories of clinical severity, used throughout this paper unless otherwise stated (Figures 1A; Data S1). These were: (A) asymptomatic HCWs; (B) HCWs who either were still working with mild symptoms insufficient to meet the criteria for self-isolation (Rivett et al., 2020) or were symptomatic and self-isolating; (C) patients who presented to hospital but never required oxygen supplementation; (D) patients who were admitted to hospital and whose maximal respiratory support was supplemental oxygen; and (E) patients who at some point required assisted ventilation. Three patients who died without admission to intensive care were also included in this severe group. In addition, 45 healthy controls (HCs), distributed across a range of age and gender, were recruited.

We analyzed the immune phenotype of 605 blood samples from 246 participants out to 90 days from the onset of symptoms (Figure 1A; Tables S1 and S2). As the clinical severity category increased, patients were more likely to be older and to be male (Figures 1B and 1C), as expected (Wang et al., 2020). A high-sensitivity C-reactive protein (CRP) assay demonstrated that classifying disease severity on the basis of maximal respiratory support is reflected in the CRP (Figure 1D). Patient courses are measured in time since symptom onset for groups B through to E. As they are asymptomatic, those in group A are measured from the date of their first positive swab; they are therefore likely to have been sampled later post-infection than patients in groups B–E, and are therefore not directly comparable to them in terms of time course. CRP (and, later, other variables) is compared to the interquartile range of 45 healthy controls. Nasopharyngeal swabs were assessed for SARS-CoV-2, allowing inclusion in this study, and were repeated in some patients. Initial viral titers, reflected by low PCR cycle threshold (CT) values, were higher in group E. With only occasional exceptions, patients in all severity groups had cleared the virus by 24 days after symptom onset (Figures 1D and S1A). Of the six patients with positive swabs after 30 days, four were overtly

CellPress
OPEN ACCESS

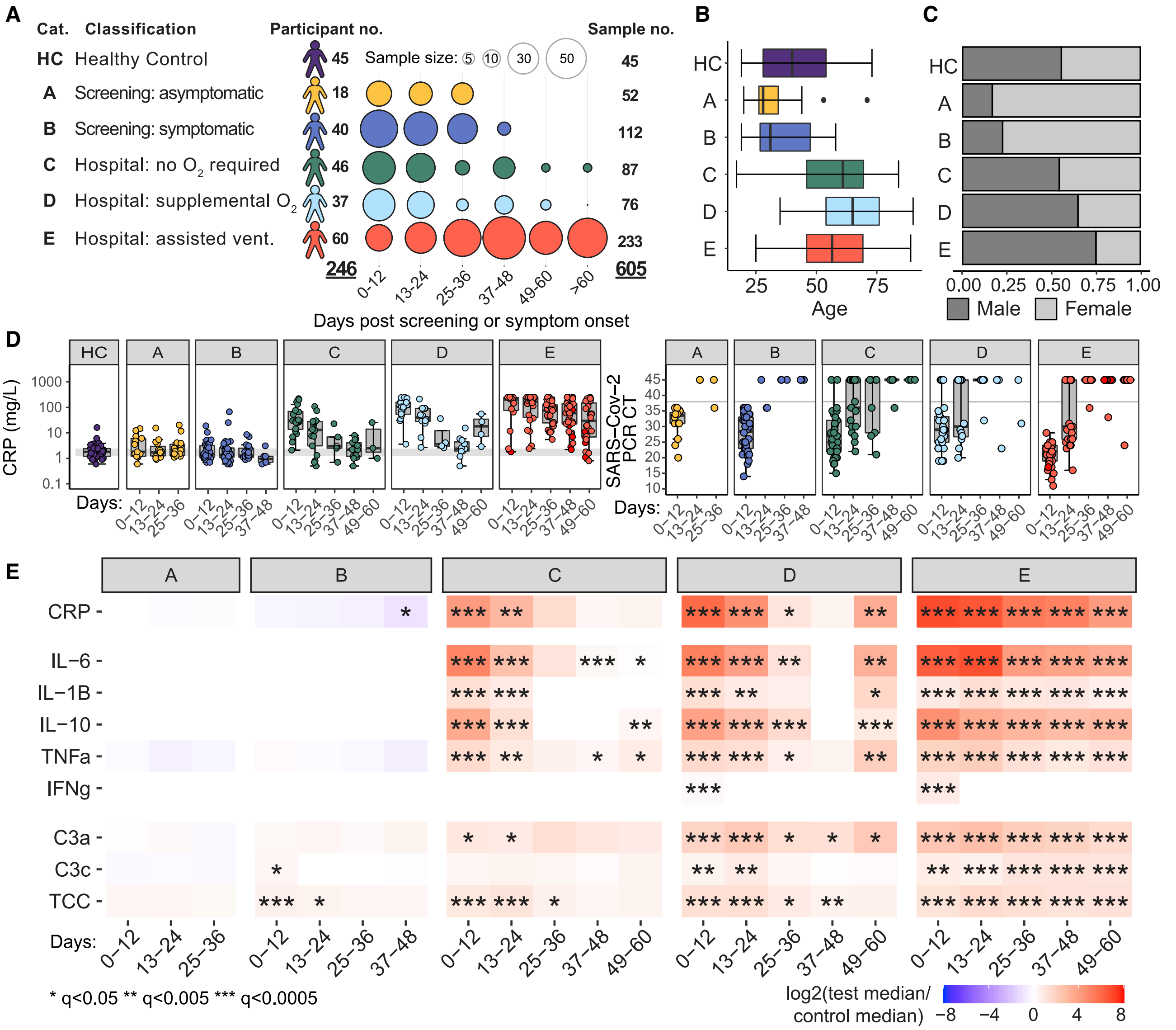

Figure 1. Cohort characteristics and changes in inflammatory markers over time

(A) Study participant and sample numbers split by severity categories and 12-day time bins post screening (group A) or symptom onset (groups B–E).

(B and C) Distribution of participant age (B) and gender (C) across severity categories.

(D) Boxplots showing CRP (mg/L) and SARS-CoV-2 PCR cycle threshold in 12-day time bins. Gray band, interquartile range of HCs or the SARS-CoV-2 swab cycle negative threshold (CT > 38).

(E) Heatmap showing $\log_2$ fold change in median CRP and serum cytokine and complement measures between COVID-19 cases and HCs, 12-day time bins. Wilcoxon test FDR adjusted p value: $^*p < 0.05$, $^{**}p < 0.005$, $^{***}p < 0.0005$.

See also Figure S1 and Table S1.

immunosuppressed (three solid organ transplants with recent induction/rejection treatment, one myeloma on B cell depletion therapy) and one was a peritoneal dialysis patient admitted with peritonitis.

We will outline the major datasets collected in this study before integrating them to study early and recovering disease.

Changes in cytokines and complement components with time and disease severity

Asymptomatic HCWs in group A had no evidence of cytokine or complement dysregulation, while those with mild symptoms (group B) showed an early, transient increase in C3c and the terminal complement complex (TCC), but not in CRP or cytokine concentrations (Figures 1E and S1B). Once patients developed symptoms severe enough to attend hospital (group C or above), a different picture was apparent. Both interleukin (IL)-6 and tumor necrosis factor (TNF)-α were raised in serum, along with other cytokines, as were all complement components measured. These abnormalities were maximal at the first bleed and largely persisted in group E. Abnormal IL-6 and TNF-α persisted in groups C and D despite clinical improvement (all had been discharged by days 49–60). Interferon-gamma (IFN-γ) was briefly

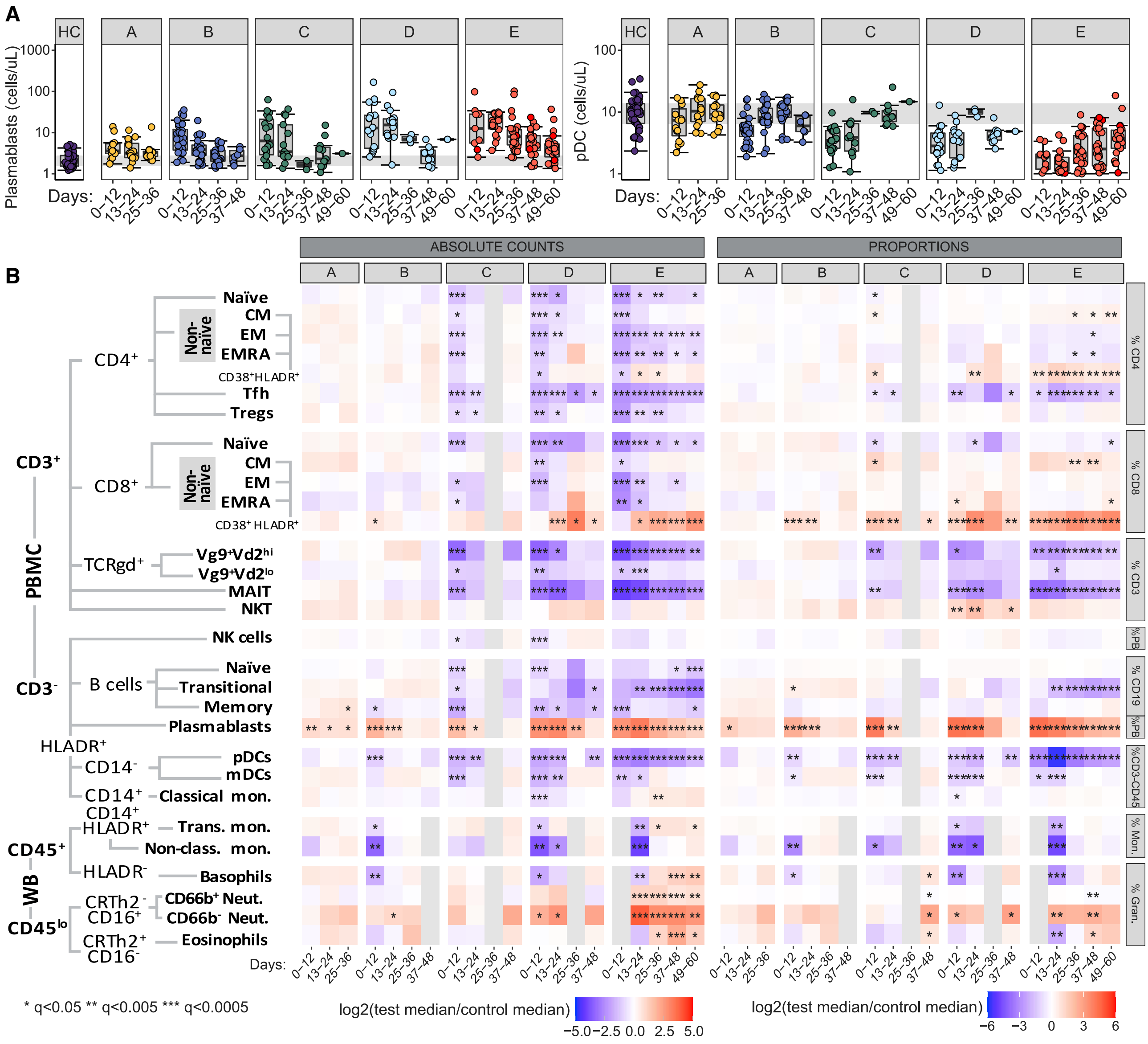

Figure 2. Profound immune abnormalities in moderate/severe COVID-19

(A) Boxplots showing absolute counts (cells/μL) for two representative cell populations, by severity groups and 12-day time bins post screening (group A) or symptom onset (groups B–E). Gray band; interquartile range of HCs.

(B) Heatmap showing the $\log_2$ fold change in median absolute cell count (left) or proportion of major subset (right) between COVID-19 cases (samples, n = 362) and HCs (n = 45), 12-day time bins.

Wilcoxon test FDR adjusted p value: *p < 0.05, **p < 0.005, ***p < 0.0005. Population hierarchy is shown to the left. Population proportions are calculated within parent populations listed to the right. PB or PBMC, peripheral blood mononuclear cells (flow cytometry); WB, whole blood (CyTOF).

See also Figure S3 and Data S2.

raised in only a subset of patients. Increased C3c was prominent in group B, while C3a became the dominant complement component elevated in more severe disease (groups C–E).

IL-6, TNF-α, IL-10, and IL-1β rose in those with more severe disease (groups C–E), but, in contrast, there was no increase in inflammatory cytokines in groups A and B, pointing to marked differences in very early inflammatory responses between resolving and progressive disease. In addition, the persistence of cytokine abnormalities even beyond 60 days from symptom onset could have implications for resolution of clinical disease.

Both onset and recovery of immune cellular abnormalities vary with disease severity

Using standardized flow cytometry panels, we explored the size of 64 cell populations over time across the five clinical strata. Trucount analysis enabled calculation of absolute cell numbers. Cellular changes with time were assessed (examples in Figure 2A), and outcomes for 30 cell types have been shown relative to the median for HCs (Figure 2B). CyTOF, which uses whole blood rather than peripheral blood mononuclear cells (PBMCs), was also used in a subset of patients to allow quantification of

CellPress
OPEN ACCESS

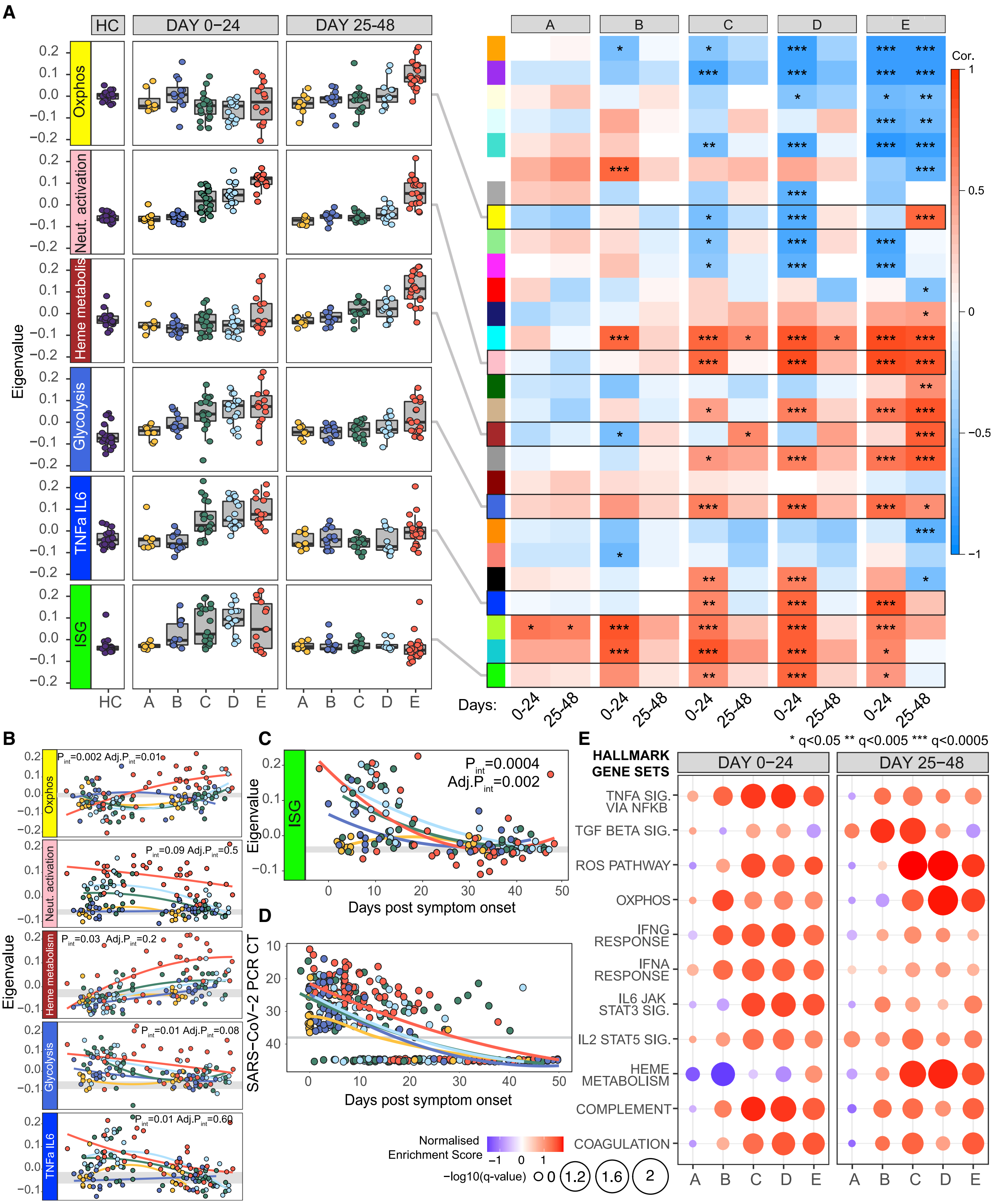

Figure 3. Whole-blood transcriptomic signatures over time (n = 183)

(A) Heatmap derived from WGCNA, correlating whole-blood co-expression gene modules (colored blocks, y axis) with COVID-19 severity groups (x axis) in 24-day time bins post screening (group A) or symptom onset (groups B–E). Boxes are colored by strength of correlation. For details of annotation (by Enrichr) and gene content of all modules, see Figure S4 and Table S3. Boxplots show eigenvalues within key transcriptomic modules, according to disease severity and time.

(legend continued on next page)

granulocytes and non-classical and intermediate monocytes (Figure S2; STAR Methods). See Data S2 for details of all cell types, including data beyond 48 days of symptom onset and time as a continuous variable.

Few changes in the immune phenotypes were seen in patients with asymptomatic (A) and very mild (B) disease, but once symptoms warranted presentation to hospital, the picture changed. Widespread immune abnormalities in groups C–E were most marked at the first bleed (Figure 2B), even when this was within 0–2 days of symptom onset (Data S2). Almost all $CD4^+$ T cells subsets were reduced, as were many $CD8^+$ T cell subsets and both naive and memory B cells; in contrast, plasmablast numbers rose in all groups. Innate lymphoid subsets, including MAIT cells, various gd T cell subsets, and natural killer (NK) cells, were also reduced, as were mDCs, and both non-classical and intermediate monocytes. These changes were correlated with, and were predictive of, severity, as discussed below.

We also calculated leucocyte number as a proportion of "parent" populations, either of total PBMCs (Figure S3A) or of major lymphoid compartments (Figures 2B and S3A), to allow comparison with most published literature (and all single-cell studies). Considering proportion underestimates COVID-19-associated pathology, for example, missing the early severity-correlated reduction in lymphocyte subsets, and the late persistence of low numbers of T cell subsets in severe disease. In agreement with this, analysis of CyTOF and CITE-seq data, which also examine cell proportions, did not identify most alterations in cell populations between severity groups that were observed by flow cytometry (Figures S3B and S3C).

Blood transcriptomic inflammation-related signatures vary with severity and time

Whole-blood RNA was isolated, and transcriptomes were generated by RNA sequencing (RNA-seq) and analyzed (in two time bins: 0 to 24 days and 25 to 48 days) using PLIER, which performs matrix factorization to identify interpretable latent factors. The contribution to each latent factor by immune cell subsets was then calculated (Figures S4A and S4B). These RNA-seq-derived latent factors were broadly aligned with the pattern observed in the cell count data (Figure 2B). An exception was the pronounced neutrophil signature seen early across groups C to E and persisting at days 25–48 in group E, demonstrating more pronounced neutrophil dysregulation across severity categories than suggested by increasing neutrophil number alone. An erythrocyte gene expression-driven latent factor was also prominent in group E at late time points, may be associated with heme metabolism, and is discussed later.

We then used unbiased weighted gene correlation network analysis (WGCNA) in the whole-blood transcriptome data to identify modules of co-regulated genes, where each could be summarized as an "eigengene." Prominent modules correlated with both disease severity and time (Figures 3A, 3B, S4C, and S4D; Table S3). The module enriched for TNF-α/IL-6 genes correlates well with the cytokine concentrations determined in Figure 1, rising early in groups C–E and then largely resolving by 25–48 days. A neutrophil activation module was also prominent early across groups C–E, as was one associated with glycolysis. Thus, there was clear transcriptional evidence of activation of broad inflammatory pathways at early time points, and these largely recovered in most patient groups (with the exception of group E, in which many patients had persistent disease). In contrast, an interferon-related module was upregulated in groups B–E at days 0 to 24 from symptom onset, before declining (Figures 3A). The relative contributions of type I, II, and III interferons to this interferon-stimulated gene (ISG)-associated module cannot be easily distinguished (Banchereau et al., 2017), but a more detailed analysis of its kinetics showed that, while expression peaked at different eigengene values in each severity group, it then declined in all of them by around 30 days (Figure 3C), coincident with viral clearance and occurring irrespective of clinical and inflammatory state (Figure 3D).

Finally, a supervised gene set enrichment analysis (GSEA) performed using Hallmark gene signatures (Figure 3E) (Liberzon et al., 2015) showed results largely consistent with the unbiased approaches just described, including demonstrating a late upregulation of genes associated with reactive oxygen species (ROS) and oxidative phosphorylation (OXPHOS) discussed below in the context of immune recovery.

Immune phenotype at presentation correlates with severity and may predict outcome

To determine whether the immune phenotype at presentation correlated with, or indeed could predict, subsequent disease course, we first performed a principal-component analysis using cell numbers across 24 primary immune cell populations from the 84 participants with blood draws taken between 0 and 10 days after the development of symptoms. Those in groups A and B clustered with HCs and away from those in groups C–E (Figure S5A). Hierarchical clustering of absolute cell counts identified two clusters (Figure 4A), one almost entirely comprised of HCWs from groups A and B (cluster 2) and the other containing all patients who progressed to ventilation and/or death and most who required supplementary oxygen support (cluster 1). Clustering using RNA-seq data obtained from 1–10 days after onset largely recapitulated that observed using cell number and was driven by ISG-, TNF-α-, and IL-6-associated gene pathways (Figure S5B). The severe cluster 1 was associated with increased age, CRP, TNF-α, and IL-6 (Figure 4B; Data S3A). Early differences between cell types drove this clustering, irrespective of their different

(B) Mixed-effects model with quadratic time trend showing the longitudinal expression of eigengenes over time by severity. Gray band, interquartile range of HCs. Nominal and adjusted p values for the time × severity group interaction term shown.
(C and D) Mixed-effects model showing longitudinal expression of eigengene capturing interferon-stimulated genes (ISG) (C) and equivalent mixed-model showing changes in SARS-CoV-2 PCR cycle threshold (viral load) by time and severity (D). y axis inverted in (D).
(E) GSEA enrichment for Hallmark genesets against HC in COVID-19 cases split by severity in 24-day time bins post screening (group A) or symptom onset (groups B–E).
FDR adjusted p value is shown by circle diameter, with color representing normalized enrichment score of the associated gene set. See also Figure S4 and Table S3.

CellPress
OPEN ACCESS

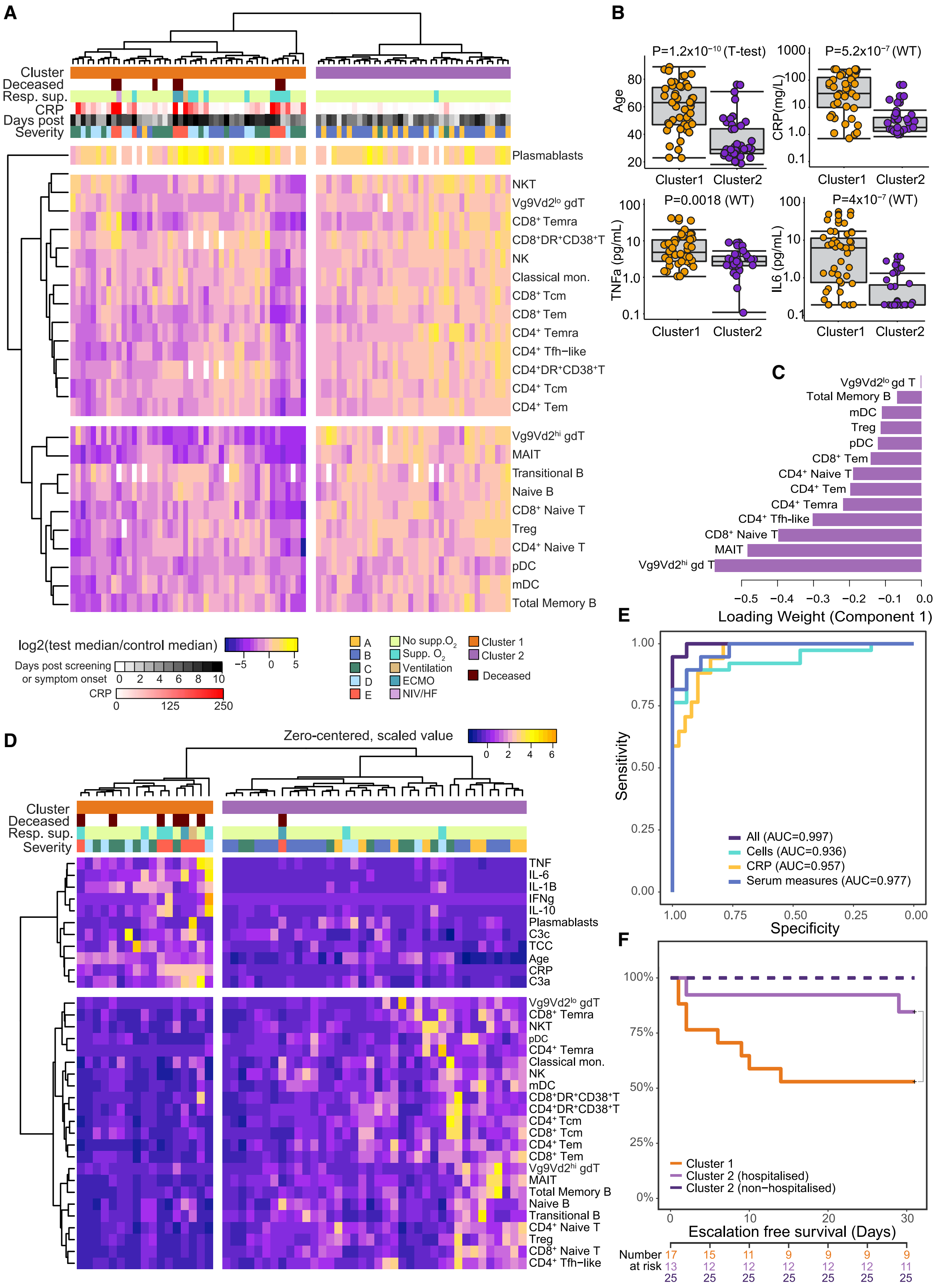

(legend on next page)

subsequent trajectories (Data S3B). We used a sparse partial least-squares discriminant analysis (sPLS-DA) to determine which cell subsets were most informative for cluster prediction: clusters 1 and 2 could be discriminated with a minimum classification error rate of 0.07 ± 0.02 (93% accuracy) based on 13 key cell populations selected by the model (Figures 4C and S5C–S5E). The area under the receiver operator characteristic (AUROC) curve for patient cluster classification based on these 13 cell types was 0.98 (98% chance of accurate cluster prediction) (Figure S5F). CRP alone was inferior to the cell types in classifying patients and did not improve their performance when added (Figure S5G). These cell types were often the most profoundly and persistently affected by severe COVID-19 (particularly MAIT, gd T, Tfh, and CD4$^+$ Temra cells).

The ability to cluster patients and separate those with mild or no symptoms (groups A and B) from those presenting to hospital (groups C–E) underlines the profound association of immune subset abnormalities with disease severity. It does not, however, predict outcome in a clinically useful way, as such predictions are only of practical value in those who present for medical attention. We therefore incorporated additional data related to inflammation, including cytokines, age, CRP, and complement components; re-clustering with this combined dataset led to a smaller severity-associated group (Figure 4D). Input from both immune cell subsets and inflammation-related markers was required for optimal classification (Figure 4E). When considering only the subset of patients recruited at hospital presentation (groups C–E) and bled within 10 days of symptom onset, this clustering predicted disease progression, as defined by the subsequent need for increased respiratory support, or death, after blood sampling (Figure 4F). This analysis requires validation with larger patient numbers but is comparable to similar observations made by others (e.g., Laing et al., 2020; Mathew et al., 2020) and indicates that a combination of immune phenotype data and inflammatory markers could provide potentially clinically useful prediction of disease progression.

Early immune responses correlate with COVID-19 severity

HCWs in groups A and B did not progress to severe COVID-19 disease and also clustered apart from those with more severe disease when using either early immune cell counts or transcriptome data (Figures 4A and S5B). Therefore, we compared the early features of immune responses in groups A and B to those in patients with more severe COVID-19, binned in 7-day intervals to provide finer definition, to seek early immune and inflammatory correlates of disease severity (Figure 5A).

A number of key features emerged. First, there was no evidence of systemic inflammation in groups A and B. CRP was normal (Figure 5A), cytokines were not raised (Figure 1E), and there was no RNA evidence of systemic inflammatory gene-related signatures (Figures 3A and 3B). Exceptions were transient increases in C3c and TCC. Most cell types that were profoundly reduced in groups C–E were normal in A and B (Figures 2 and S3), but some showed mild reductions in group B; pDCs and memory B cells have been shown as examples (Figures 5A–5C, S6A, and S6B). The fall in pDCs was modest and brief compared to severe disease and was consistent with tissue localization and local interferon production to support the anti-viral response (Cella et al., 1999).

In group B, cytotoxic CD8$^+$ T cells rose earlier than in groups C–E, apparent by day 7 and peaking up to 2 weeks after symptom onset, in contrast to the later and more sustained rise seen in severe COVID-19 (Figures 5A, 5B, and S6A). Early enrichment of a CD8$^+$ cytotoxic RNA signature was also seen in group B compared to C to E (Figure S6C). Consistent with these findings, spontaneous generation of IFN-γ by T cells was more pronounced in group B samples taken 2 weeks post symptom onset (Figure 5D).

Antigen-specific CD8$^+$ T cell responses were determined after stimulation with anti-SARS-CoV-2 peptides and subtraction of background spontaneous IFN-γ-producing T cells. They were similar in all severity groups early (Figures 5D and S6D) and so were not responsible for the increased effector CD8$^+$ T cells seen in mild disease, suggesting instead a role for "bystander" activation. Such bystander-activated cells are important in early anti-viral defense (Maurice et al., 2021) and characteristically express NKG2D, involved in the killing of infected cells, and both IL-7 receptor (IL7R) and CD8, which are downregulated in a T cell receptor (TCR)-dependent fashion (Kaech et al., 2003; Slifka and Whitton, 2000). CITE-seq protein expression analysis of activated CD8$^+$ T cells demonstrated an increase in surface NKG2D, IL7R, and CD8 in group B compared to more severe groups (Figure 5E), observations confirmed by RNA analysis (Figure 5E). Bystander CD8$^+$ T cells expressing CXCR3 rapidly home to sites of inflammation (Maurice et al., 2021), consistent

Figure 4. Multivariate analysis of immune-cell populations in early disease correlates with clinical outcome

(A) Unsupervised clustering of absolute cell counts across 24 cell populations (normalized to the median of HCs) for COVID-19 samples taken $\leq$10 days from screening (group A) or symptom onset (groups B–E). Cases group into two clusters (cluster 1, orange, n = 46; cluster 2, purple, n = 38) by Euclidean distance and Ward D hierarchical clustering.

(B) Boxplots comparing age and inflammatory characteristics of individuals in clusters 1 and 2 at the time of sampling.

(C) Thirteen cell types selected by sPLS-DA as most informative in predictive models discriminating patients in clusters 1 and 2. Bars indicate loading coefficient weights of selected features (ranked from most to least informative in cluster prediction, from bottom to top).

(D) Addition of age, CRP, serum cytokine, and complement measures to unsupervised clustering of cellular data in (A) results in tighter grouping of COVID-19 patients by severity (cluster 1, orange, n = 17. cluster 2, purple, n = 38).

(E) AUROC curve showing sensitivity and specificity of severity group prediction (derived from clustering in D), based on absolute counts of 24 key cell types, CRP, or serum measures alone compared to all available measures.

(F) Kaplan-Meier plot of escalation-free survival in individuals within severity cluster 1 or cluster 2 split by hospitalization status. Escalation defined as a step up in respiratory support or death.

p value for the chi-square test of the difference between cluster 1 (n = 17) and hospitalized patients in cluster 2 (n = 13) is shown; numbers denote non-escalated patients in each group from days 0 to 30 post symptom onset.

See also Figure S5 and Data S3.

CellPress
OPEN ACCESS

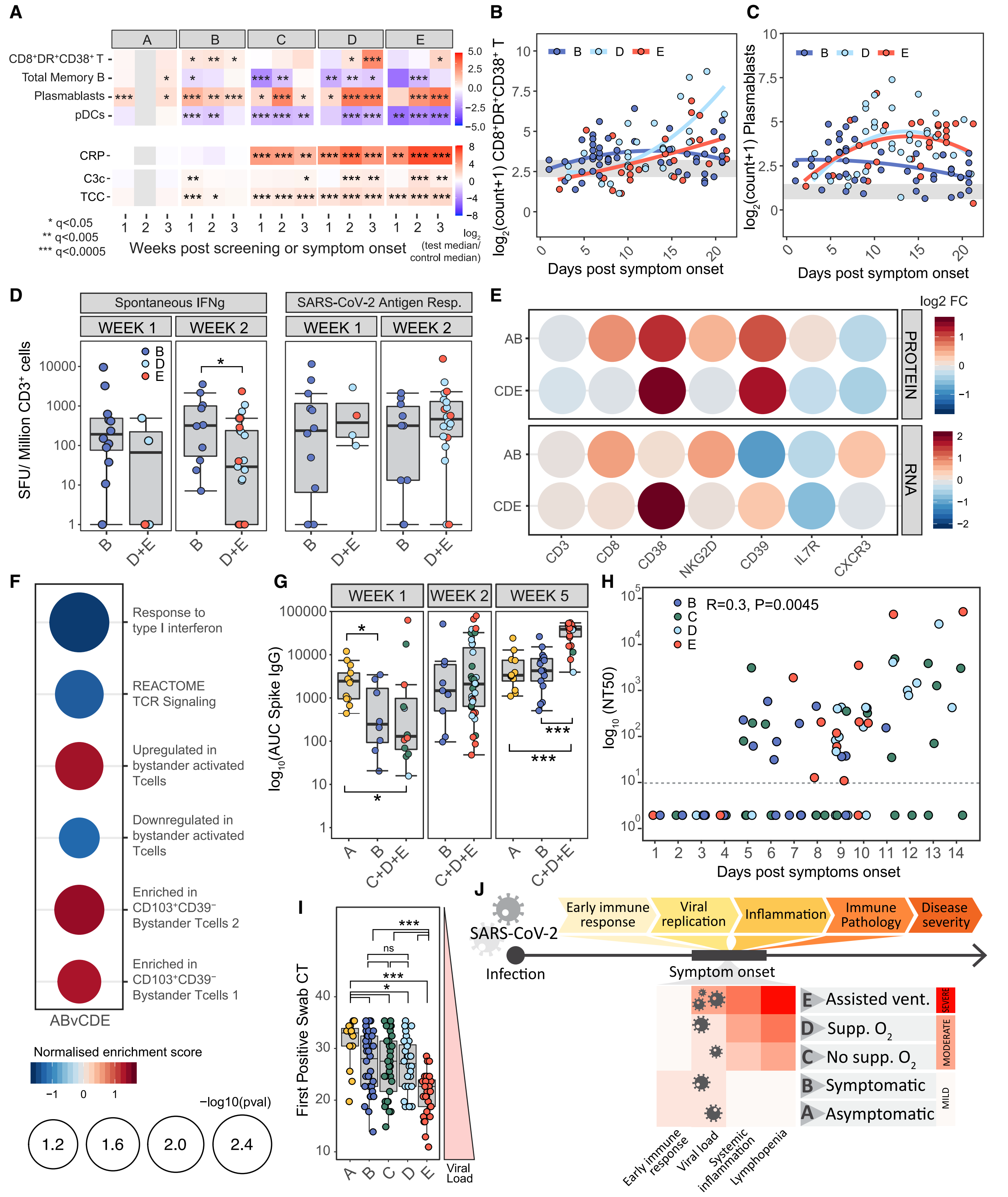

Figure 5. Early immune changes associated with mild or severe disease outcome

(A) Heatmap showing the $\log_2$ fold change in median absolute cell counts, CRP or complement measures between COVID-19 cases and HCs by severity and in 7-day time bins post screening (group A) or symptom onset (groups B–E). Wilcoxon test FDR adjusted p value: *p < 0.05, **p < 0.005, ***p < 0.0005.

(B and C) Mixed-effect model with quadratic time trend showing cellular trajectories over time in sample groups B, D, and E in non-naive HLA-DR+CD38+ CD8 T cells (B) or plasmablasts (C) (cells/μL) from weeks 1–3 post symptom onset (samples, n = 207).

(legend continued on next page)

with enrichment for CXCR3 RNA, but not surface protein (Figure 5E). Transcriptional signature-derived bystander-activated CD8$^+$ T cells were enriched in patients with mild disease, while those from TCR-activated cells were associated with severe COVID-19 (Figure 5F), again consistent with widespread early bystander activation in the CD8$^+$ T cell population in patients destined to have good disease outcomes.

An early increase in plasmablasts was seen in groups A and B, occurring up to a week before a larger rise in more severe disease (Figures 5C and S6B). We therefore measured total immunoglobulin concentrations and anti-spike IgG and anti-SARS-CoV-2 neutralising antibody titers (Figures 5G, 5H, S6E, and S6F). Group B patients maintained their serum IgM concentrations, which fell rapidly in those with more severe disease (Figure S6G), and their titers of anti-spike IgG and early neutralisation responses were comparable to patients progressing to more severe COVID-19 (Figures 5G and 5H). This suggests that the B cell difference between patients with mild and more severe disease might lie in more robust non-antigen-specific B cell activation, perhaps impacting via "natural" antibody and/or non-antibody-dependent mechanisms.

Virus at first swab, as assessed by PCR CT value, was comparable in groups B, C, and D (and the lower titer in group A samples was not comparable as they will have been taken later after infection, as described above). Initial viral titer was therefore not associated with an increased risk of hospital admission (being similar in groups B, C, and D) but was higher in group E (Figure 5I). These viral titers were reflected in interferon-related transcription signatures, which are prominent in groups B–E (Figures 3A, 3C, and S6H). Despite the fact that high IFN signatures correlated with severe disease, they were not necessarily driving that severity. The fact that the subgroup with the highest IFN *within* group E do best (Figure S6I) suggests that the inflammatory pathways that create severe disease are distinct from IFN (which is also consistent with comparable kinetics of reduction of IFN signature regardless of severity; Figure 3C) and that a robust IFN response may be beneficial in this context, though this needs confirmation in a larger dataset.

Taken together, these data suggest that an early adaptive immune response is prominent in individuals who are asymptomatic or have mild disease, characterized by a rapid production of activated bystander CD8$^+$ T cells, plasmablasts, and likely pDC tissue localization before antigen-specific responses become apparent. This appeared a more important correlate of severity than viral titer, which only became relevant in those progressing to ventilation or death (Figure 5J).

In those with more severe disease (groups C–E), evidence of systemic inflammation was present from the first blood test (Figure 5). If we focused on the 16 patients in groups C–E sampled between 2 days before and 4 days after symptom onset, 15 had a CRP >10 mg/L and/or neutrophil activation eigengene >0. All five patients sampled between 2 days before and 2 days after symptom onset met these criteria. In contrast, this was not seen in group A or B despite the fact that they mounted a more prominent early cellular response. It was not clear whether inflammation seen in C–E was causally related to the poor early cellular response seen in these patients. Such inflammation clearly did not, however, develop later from the progression of a non-inflammatory immune response or as a result of failure to clear virus and suggested that the inflammatory die is cast by the time symptoms appear; thus, strategies to prevent it and reduce its clinical impact would need to be established very early (Figure 5J).

Distinct patterns of immune recovery in COVID-19

In contrast to groups A and B, cellular changes in groups C–E were profound and usually most prominent at the first bleed (Figure 2), so determination of change in immune cell subsets over time was likely to be most informative in these groups. Therefore, we explored cell kinetics in groups C–E, assigning patients to two categories based on whether their CRP concentrations remained elevated above 10 mg/L ("persisting CRP") or fell below 10 mg/L ("resolving CRP") by their final bleed within 3 months post symptom onset (Figure 6A). The latter group included both individuals with early high CRP that then fell, together with those for which CRP remained low (10 mg/L) throughout. Changes in CRP over time differed between these two groups when assessed using a mixed-effects model, with time modeled as a continuous variable (Figure 6B).

To compare cellular changes over time between persisting and resolving CRP patient groups, a "rate of change" for each cell population was calculated over 60 days post symptom onset. In brief, this rate captured both the initial deviation in cell counts from normal within a window of 0–12 days and the time taken for cells counts to stabilize within a normal range if cellular recovery did occur (see STAR Methods). Five predominant trajectories were observed; populations that did not deviate from heathy numbers over the duration of study (e.g., NKT cells), those that increased progressively from normal over time (e.g., effector CD8$^+$ T cells), those that fell progressively from normal over time (e.g., transitional B cells), those that trended toward recovery after an initial rise in numbers (plasmablasts), and those

(D) Number of CD3$^+$ T cells secreting IFN-γ spontaneously or following SARS-CoV-2 antigen stimulation in samples from groups B (n = 22) and D and E combined (n = 25), 1 or 2 weeks post symptom onset. Kruskal-Wallis test p values: *p < 0.05.

(E) Log$_2$ fold change (FC) in expression of CD8$^+$ T cell transcripts reflecting T cell activation and surface protein expression (detected by antibody staining) in CITE-seq data from non-naive CD8$^+$ T cells from patients in groups A and B (n = 5) and C, D, and E combined (n = 13), relative to HCs (n = 11).

(F) Normalized gene set enrichment score for gene sets associated with TCR-dependent and bystander T cell activation in single-cell transcriptomic data from non-naive CD8$^+$ T cells from patients in groups A and B versus C, D, and E. FDR adjusted p value shown by circle diameter.

(G) Area under the curve for SARS-CoV-2 spike-specific IgG titers at 1, 2, and 5 weeks post screening (group A) or symptom onset (groups B–E). Groups C, D, and E are combined to increase statistical power, Kruskal-Wallis test p values annotated as in (A).

(H) SARS-CoV-2 antibody titers achieving 50% neutralization (NT50) in patients from groups B–E in the first 2 weeks post symptom onset (n = 102). Samples with no detectable neutralizing activity at the lowest dilution (dotted line) are plotted at an arbitrary NT50 of 1. p value and Pearson's correlation shown.

(I) Boxplots showing SARS-Cov-2 viral load, taken as first positive swab PCR CT, in severity groups. Wilcoxon test p values annotated as in (A).

(J) Schematic summarizing variation in immune features of SARS-CoV-2 infection across cases of varying disease severity.

See also Figure S6.

CellPress
OPEN ACCESS

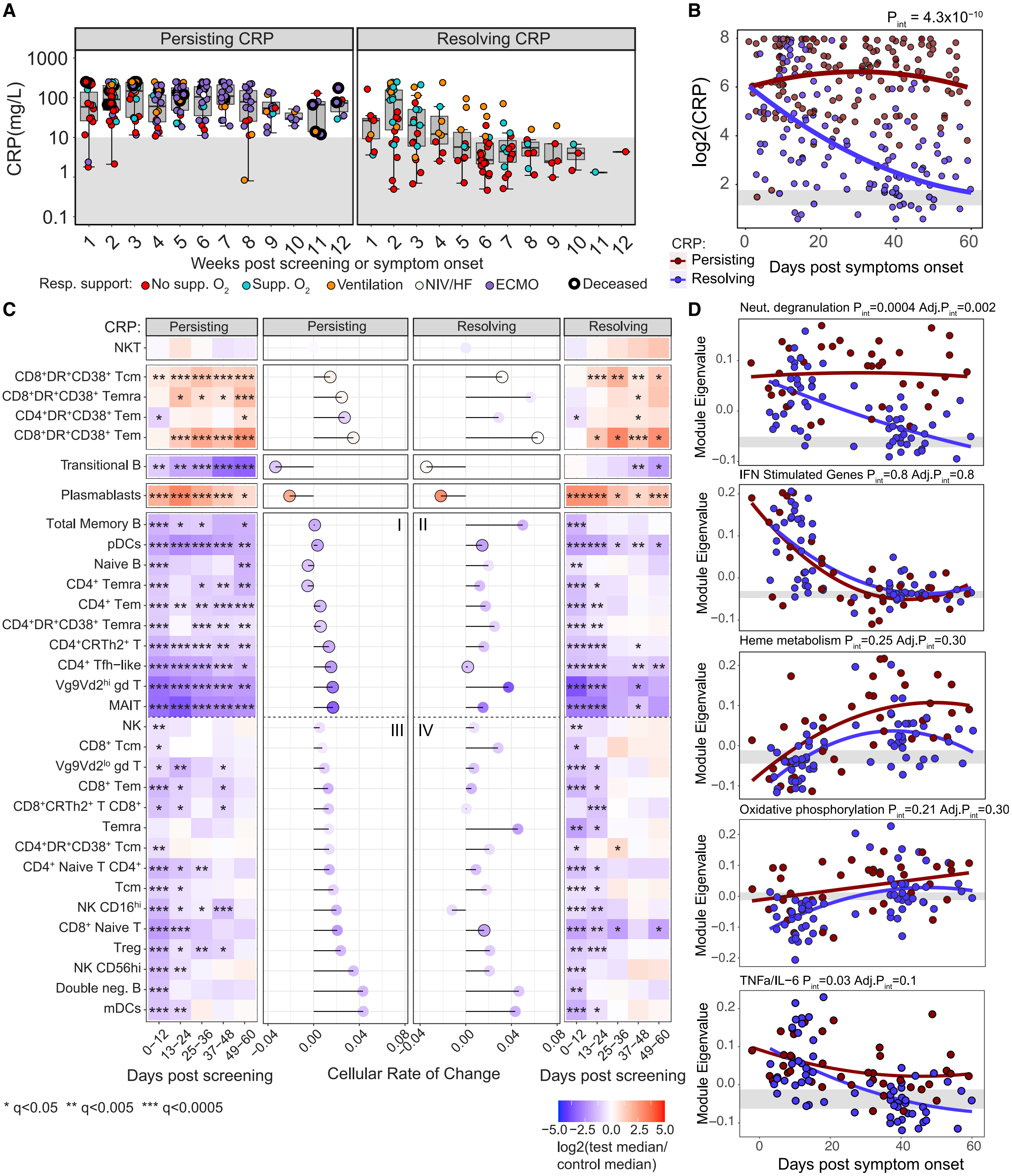

Figure 6. Cellular and transcriptional trajectories in persisting and resolving disease (n = 263)

(A) CRP (mg/L) from groups C, D, and E grouped by persisting and resolving CRP.

(B) Mixed-effect model with quadratic time trend showing log_2(CRP) trajectories in both patient groups, and the likelihood-ratio test p value for the time × group interaction term. Gray band, interquartile range in HCs.

(C) Heatmap showing the log_2 fold change in median absolute cell count between COVID-19 cases in groups C, D, and E, split according to persisting or resolving CRP, and HCs. 12-day time bins. Wilcoxon test FDR adjusted p value: *p < 0.05, **p < 0.005, ***p < 0.0005. Rate of cell number changes shown by lollipop plot;

(legend continued on next page)

that tended toward recovery after an initial drop in numbers (e.g., naive CD4$^+$ T cells) (Figure 6C).

The absolute number of most cell populations fell precipitously early and then showed variable recovery. For descriptive purposes, these were arranged into a group of cell subtypes that failed to recover, or recovered, in the persisting CRP group (Figure 6C, quadrants I and III, respectively) and their equivalents in the resolving CRP group (Figure 6C, quadrants II and IV, respectively). Notably, a few populations remained abnormal in both persisting and resolving CRP groups out to 60 days post symptom onset, including pDCs, Tfh, MAIT, and Vg9Vd2 (hi) gd T cells. All other populations showing an early drop in counts (with the exception of naive CD8$^+$ T cells) recovered in those with resolving CRP (II and IV) and at rates more rapid than seen in those with persisting high CRP. In the persisting CRP group, a number of cell types remained markedly abnormal (including memory B cells and various CD4$^+$ T cell subsets: quadrant I), whereas a second group of cell types recovered despite persisting inflammation (including NK cells and some CD8$^+$ T cell subsets: quadrant III).

We then explored the relationship between cell recovery and the inflammatory response. It might be expected that where CRP remained persistently elevated, immune defects might persist, if these defects were secondary to the inflammatory state. Consistent with this, the cohort with persistently raised CRP also has raised TNF-α and IL-6 protein (Figure 1D). Likewise, transcriptional signatures of TNF-α/IL-6 and neutrophil activation were increased in severe disease (Figure 3), particularly in the persistent CRP group (Figure 6D). This ongoing inflammation may contribute to the sustained reduction in cell numbers at late times seen in quadrant I, together with persistently raised HLA-DR$^+$/CD38$^+$ effector T cells and plasmablasts (Figure 6C). Therefore, it was also not unexpected that most cell types reduced in acute disease recover over a few weeks as the CRP falls, as was seen for most cells in quadrants II and IV (Figure 6C).

More intriguing were the cells that recovered rapidly in the face of ongoing inflammation (quadrant III). While the reasons for this are likely to differ between cell types and to be multifactorial, these cell reductions might be driven in part by the viral infection per se and/or virus-induced interferon. It was notable that, after initially rising, IFN-γ (Figures 1E and S1B) and ISG signatures fell to normal values independent of both disease severity group (Figure 3C) and CRP (Figure 6D) but correlated with declining virus titer (Figures 3D and S4E). Thus, cell types known to leave the circulation due to interferon stimulation (Kamphuis et al., 2006), such as T and NK cells (Hirsch and Johnson, 1986; Zafranskaya et al., 2007), may recover as viral infection is controlled and interferon-dependent inflammation falls, independent of ongoing CRP-associated inflammation.

Finally, a small number of cell types remained statistically abnormal after 60 days, even in the resolving CRP group. Thus, effector CD4$^+$ and CD8$^+$ T cells and plasmablasts remained elevated, and pDCs, Tfh, MAIT, and Vg9Vd2-expressing gd T cells remained reduced (Figure 6C) and were among those cells most predictive of poor prognosis (Figure 4C). These abnormalities persist despite resolution not only of CRP but of neutrophil degranulation, TNF-α/IL-6, and glycolysis-related signatures (e.g., Figures 1D and 6D). Possible mechanisms behind these sustained abnormalities are discussed below.

The late appearance of OXPHOS and ROS pathways correlates with differential immune recovery

In late, severe COVID-19, whole-blood transcriptome analysis showed prominent inflammation-related signatures that were distinct from those seen early in disease. These signatures were related to OXPHOS, ROS, and heme metabolism. These were demonstrated in an unbiased fashion using WGCNA, where modules characterized by OXPHOS and heme metabolism were prominent at days 25 to 48 post symptoms, with OXPHOS most prominent in group E and heme metabolism in C-E (Figures 3B and 3C). Enrichment of Hallmark signatures confirmed the association of OXPHOS and heme metabolism in groups C–E and also found association of a ROS signature (Figures 3E and 7A). Consistent with this, the expression of the genes driving the enrichment of each signature was upregulated in the three most severe clinical groups (Figure 7B; Table S3). The late rise in these three correlated signatures occurred irrespective of persisting or resolving CRP-associated inflammation (Figure 6D) and appeared independent of specific cell population recovery (Figure S7A).

We then correlated cellular and transcript signature changes in COVID-19. In the first 24 days after symptom onset, there was a strong association between TNF-α/IL-6, neutrophil degranulation, and interferon signatures with most cell subsets reduced in severe disease (Figure S7B). Later, between 24 and 48 days after symptom onset, these associations changed (Figures 7C and S7C). While TNF-α/IL-6 and neutrophil degranulation signatures were still associated with many cell subsets that continue to be reduced, the interferon signature was no longer a significant player. The persistent increase seen in activated effector CD4$^+$ and CD8$^+$ T cells and plasmablasts was now particularly associated with the OXPHOS signature, which, having become more prominent later in disease (Figure 3B), had a much more restricted and specific association with immune dysfunction than other inflammatory signatures.

It was thus clear that, for some cell types, the association between their number and the inflammatory milieu changed over time but, for others, was more consistent. It was the inflammatory signatures that appeared late in disease, in particular OXPHOS, that were specifically associated with persistent derangement of cell types of potential pathological importance, such as increased HLA-DR$^+$CD38$^+$ T cells and plasmablasts and reduced pDCs (Figure 7D).

DISCUSSION

In asymptomatic or mildly symptomatic, non-progressive SARS-CoV-2 infection (groups A and B), there was evidence of an early

faster rate of recovery, or deviation from normal, is indicated by increasing stem length. Points are colored by log_2 fold change in median absolute cell counts from HCs at 0–12 days; black outline indicates failure to recover to HC numbers within 60 days (defined in STAR Methods).

(D) Mixed-effect models showing longitudinal trajectories of gene module eigenvalues capturing neutrophil degranulation, interferon-stimulated genes, heme metabolism, and oxidative phosphorylation in CRP groups, p values reported as (B).

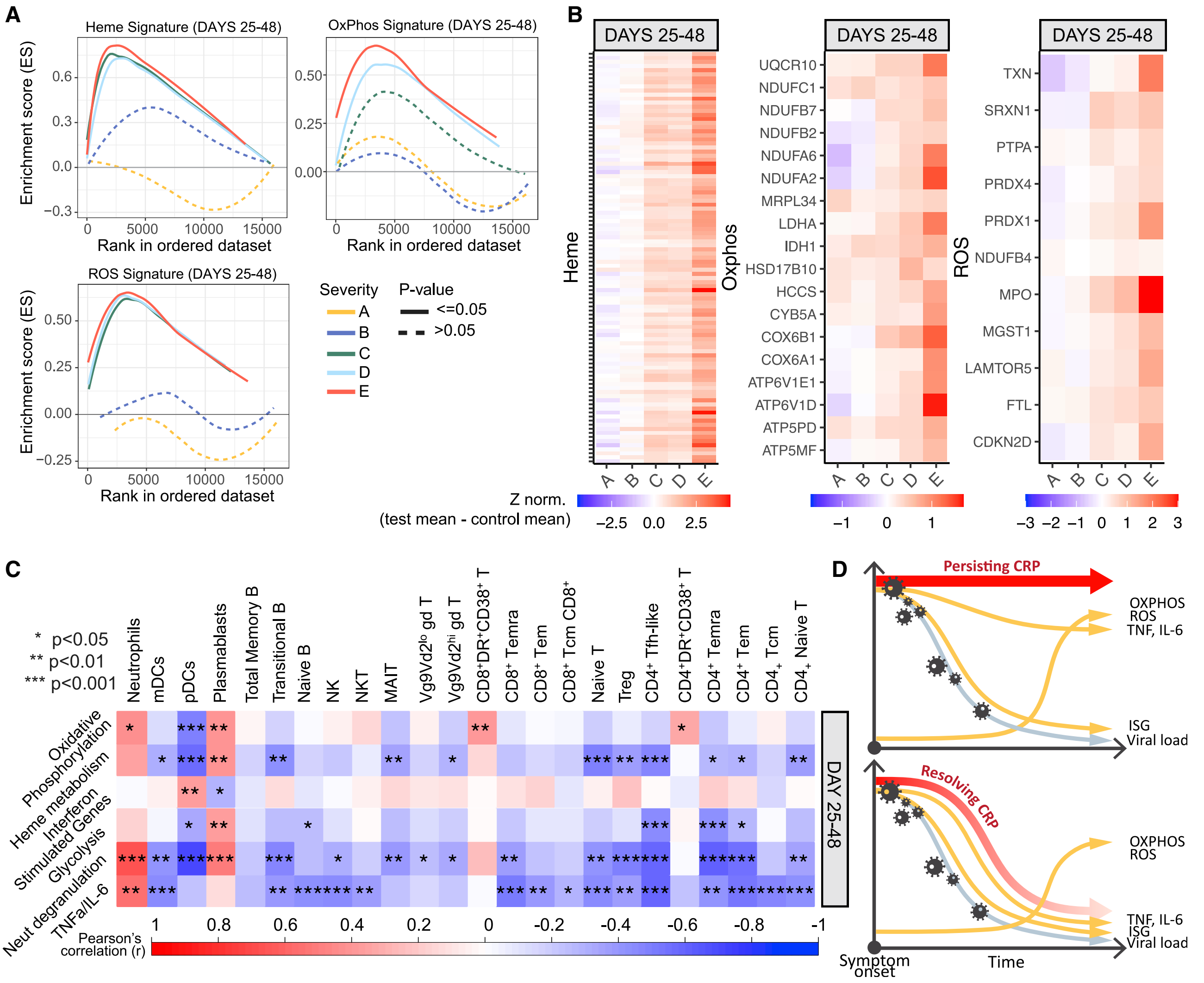

Figure 7. Altered transcriptional changes in prolonged disease (n = 183)

(A) Enrichment score for Hallmark genesets capturing heme metabolism, OXPHOS- and ROS-related genes (by GSEA) in groups A–E in samples taken 25–48 days post screening (group A) or symptom onset (groups B–E).

(B) Heatmap showing relative expression of the intersection of GSEA leading edge genes from groups C, D, and E, across severity groups in samples taken 25–48 days post screening (group A) or symptom onset (groups B–E).

(C) Heatmap showing correlation between transcriptional eigengenes and absolute cell counts, at 25–48 days post symptom onset. Boxes are colored by strength of correlation, Pearson correlation p values: *p < 0.05, **p < 0.01, ***p < 0.001.

(D) Schematic representation of the trajectory of immunological changes in SARS-CoV-2 infection over time in patients with persisting or resolving systemic inflammation.

See also Figure S7.

robust adaptive immune response. Circulating plasmablasts and $CD8^+HLA\text{-}DR^+CD38^+$ activated T cells expanded earlier and in higher numbers than in more severe COVID-19 groups, most notably in the first week after symptom onset. Both of these cell populations then contracted in A and B, as they continued to rise in groups C–E. Despite this, and a prominent early interferon transcription signature, both the antibody and T cell SARS-CoV-2-specific immune responses were comparable between early mild and severe disease. This suggested that the increased plasmablasts and effector $CD8^+$ T cells could reflect an enhanced bystander response in mild disease, something then supported by single-cell CITE-seq analysis.

Bystander $CD8^+$ T cell activation was first described in the context of lymphocytic choriomeningitis virus (LCMV) infection in mice (Tough et al., 1996). It involves activation of memory $CD8^+$ T cells independent of TCR stimulation (whether by the initiating or cross-reactive antigen) (Maurice et al., 2021). It is driven by type I IFN in many inflammatory contexts—in particular, viral infection. Bystander $CD8^+$ T cells are activated well before the antigen-specific response is seen, being detectable within a day of viral infection and often peaking within a week (Berg et al., 2003). Bystander cells can migrate to infected organs using CXCR3 and kill virally infected cells through NKG2D- and granzyme-B-dependent mechanisms (Maurice

et al., 2019). They have been shown to be critically important in the early defense against viral infections, in part by direct antiviral effects and in part by producing IFN-γ and other cytokines that then activate antigen-presenting cells (Soudja et al., 2014) and control the memory/effector balance of subsequent antigen-specific responses (Krummel et al., 2018). Similar, though less well defined, bystander phenomena occur in CD4$^+$, MAIT, and gd T cell responses (Holzapfel et al., 2014; Ribeiro et al., 2015; Ussher et al., 2018). The early and pronounced production of bystander-activated CD8$^+$ T cells in patients groups A and B could be directly related to disease resolution and prevention of progression to more severe disease, a process likely to begin before symptom onset. Understanding the factors that impede this early bystander response could be important for developing strategies to prevent severe disease.

In contrast, persistent bystander CD8$^+$ T cell activation has been associated with inflammatory pathology in the context of both chronic infection and autoimmunity. NKG2D-dependent killing of non-virally infected hepatocytes in hepatitis A exacerbates liver damage (Kim et al., 2018) and may also play a role in inflammatory lung disease (Borchers et al., 2009), and there is evidence that NKG2D ligands are expressed in the lungs in COVID-19 (Wauters et al., 2021), so it could be that a late and persistent expansion of bystander-activated effector T cells in severe COVID-19 could be a driver of lung pathology. The well-documented links between bystander CD8$^+$ T cell activation and autoimmunity (Groh et al., 2003; Meresse et al., 2004) also raise the possibility that they may play a role in the autoimmune manifestations observed in COVID-19.

The early increase in circulating plasmablasts seen in mild COVID-19 is likely to be comprised of SARS-CoV-2-specific cells, perhaps to an extent not yet fully reflected in serum antibodies, together with non-SARS-CoV-2-specific bystander-activated cells. Bystander B cell activation is known to involve memory (Horns et al., 2020) as well as innate-like B cells (including B1 cells in the mouse and marginal zone cells in both mouse and human). Their function is not fully defined but includes an increase in production of "natural antibodies" (Antin et al., 1986) alongside non-antibody-associated functions, which may include antigen transport to secondary lymphoid organs, antigen presentation to T cells, cytokine production, and immune regulation. These bystander B cell functions are known to play a role in early defense against bacterial infection (Boes et al., 1998; Haas et al., 2005; Ochsenbein et al., 1999) and may be involved in autoimmunity (Espéli et al., 2019; Sanderson et al., 2017). Their role in viral infection is less clear, but it may be that early bystander B cell activation helps determine the outcome of COVID-19 in a manner analogous to the role proposed for bystander CD8$^+$ T cells. A closer investigation of the early B cell response in COVID-19 will be required to confirm this.

At the same time as this pronounced early immune response is seen in groups A and B, there is no evidence of systemic inflammation, apart from early, transient complement activation. CRP, circulating TNF-α and IL-6, and transcriptional signatures of a number of inflammatory pathways are not raised in groups A and B but are already prominent in groups C–E. The severe and widespread leucocyte subset depletion seen at the initial bleed in the more severe COVID-19 patient groups C–E, and observed by many others (Carissimo et al., 2020; Laing et al., 2020; Mathew et al., 2020; Su et al., 2020), is not apparent in those with asymptomatic or mildly symptomatic disease, suggesting that this, too, is a feature of a pathological immune response. Immune cell subset numbers correlated strongly with severe and progressive disease. Coupled with evidence of early systemic inflammation in severity groups C–E, our findings suggest that the immune pathology associated with severe COVID-19 either is established immediately post infection or, if there is a transition point from an effective to a pathological response, is likely to occur before the time of symptom onset. This finding may have major implications as to how disease needs to be managed, because intervention to prevent immune pathology would need to be targeted very early in the disease course and perhaps pre-emptively in high-risk groups screened and diagnosed before symptoms develop.

The reason for the failure to mount a robust early B and T cell response in the context of severe COVID-19 was likely to be multifactorial. There was no evidence for a relationship with viral load and progression to inflammatory disease, as initial viral titers were comparable between groups B, C, and D. Once inflammatory disease is established, however, viral titer may be associated with subsequent outcome, as increased viral titer was seen in group E, consistent with reports that high initial viral titer might be associated with mortality (Pujadas et al., 2020). Genetic association studies in severe COVID-19 point to genes that are implicated in driving antiviral responses. The most prominent are associations with genes involved in the type I interferon pathway (Pairo-Castineira et al., 2020; Zhang et al., 2020), known to be the key driver of bystander T cell activation (Maurice et al., 2021). Increasing age and comorbidity such as diabetes and chronic inflammatory disease are known to suppress early CD8$^+$ T and B cell responses (Shen-Orr et al., 2016; Weiskopf et al., 2009). An in-depth understanding of these risk factors may instruct strategies to assess risk of progression before inflammatory responses become self-sustained.

While clear distinctions in immune responsiveness were apparent between groups A and B versus groups C, D, and E, differences between the more severe groups themselves were less obvious. Those with symptomatic disease warranting admission to hospital clustered together using the size of just 13 key cell populations: these clusters correlated strongly with clinical severity, and immune cell subset numbers together with inflammatory mediator concentrations provided prediction of subsequent progression, as well as COVID-19-associated death. Similar observations have been made by others (Chen and John Wherry, 2020). Predicting progression after presentation to hospital with moderate/severe disease could be of limited clinical use, and it would be of more benefit to predict progression in cases with milder COVID-19, but this may not be possible in practice, given that inflammatory immunopathology is likely to be present at symptom onset. A study to address this issue would need to be conducted in particularly high-risk patient groups to ensure an adequate event rate and would require diagnosis through asymptomatic screening to detect changes before symptoms develop.

We can find no equivalent studies in other severe infections that explore the kinetics of the cellular immune response across this range of time and clinical severity. Nonetheless, many of the changes seen in response to SARS-CoV-2 have been reported in

CellPress
OPEN ACCESS

the context of other viral infections. Both an early reduction in blood pDC and T cells has been shown in respiratory syncytial virus (RSV) (Russell et al., 2017) and influenza A (Fox et al., 2012; Lichtner et al., 2011), for example. NK cells in the blood also fall in influenza, probably as a result of migration to the lungs. Finally, plasmablast expansion has also been seen, particularly in dengue fever (Wrammert et al., 2012). The nature of these studies makes a direct comparison with the situation in SARS-CoV-2 difficult, but it would seem likely that many immunological changes seen in COVID-19 mirror those seen in responses to other infections, though, in general, the cellular changes we observe appear to be more profound, peak earlier in disease course, and are more persistent in COVID-19 than in studies performed in other infectious contexts.

The recovery of the profound immune dysregulation seen in severe COVID-19 is potentially of major clinical relevance, as such recovery may be required for the resolution of inflammatory disease or to prevent secondary infection or SARS-CoV-2 reinfection. We found that immune cell abnormalities often persist for weeks to months after SARS-CoV-2 infection, and different cell populations exhibited quite different patterns of resolution. Some recovered as systemic inflammation resolved, others did not, and a third group resolved even in the face of persistent systemic inflammation. Understanding the inflammatory drivers or associations of this differential recovery could provide insight into the immune pathology of COVID-19 and potentially of other infections. To begin to explore this, we correlated immune changes with measurements of systemic inflammation throughout the disease course. Patients with severe COVID are characterized by high CRP, and this correlates with evidence of TNF-α- and IL-6-driven processes at both the protein and transcriptome expression levels, as well as with both neutrophil activation and glycolytic metabolism. The fact that many cellular abnormalities persisted while these biologic processes were apparent, while others appeared to resolve alongside them, suggests that the nature of systemic inflammation is important in driving different aspects of immune pathology.

Finally, some cell populations remained markedly abnormal or showed a limited recovery, even once CRP-associated inflammation had resolved and indeed after patients had been discharged from hospital. These persistent changes may reflect a slow intrinsic regenerative capacity of the cell type concerned, but in other situations, such as the continued elevation of effector T and B cells, it is tempting to speculate that there is ongoing abnormal signaling driving such changes. For that reason, we explored late changes that are seen in the inflammatory response in COVID-19. Transcriptional signatures associated with OXPHOS-, ROS-, and heme-related metabolic pathways arose late in those with severe COVID-19 but were not prominent in early disease. Activation of immune cells results in metabolic reprogramming that supports cell growth, proliferation, and differentiation. Disruption of metabolic pathways can, through many machanisms, result in immune dysfunction (Bantug et al., 2018). It is unlikely that the metabolic signatures observed here simply reflect heightened bioenergetic requirements of activated immune cells, as one would expect similar requirements to be also present early in disease. OXPHOS can drive inflammation (Mills et al., 2017), and we note that COVID-19 patients treated with metformin, which inhibits complex I of the respiratory chain, had lower amounts of circulating inflammatory cytokines (Cheng et al., 2020). The ROS transcriptional signature may reflect more abundant production of ROS, inevitably accompanying increased OXPHOS. Alternatively, it may reflect specific mitochondrial pathology and thus contribute to immune cell dysfunction (Nathan and Cunningham-Bussel, 2013). Mitochondria are also critically involved in heme biosynthesis. Heme serves as a prosthetic group for haemoglobin as well as many other proteins, including several that constitute the respiratory chain of mitochondria. While free heme can act as a damage-associated molecular pattern and promote ROS formation, the role of heme biosynthesis versus catabolism in balancing cellular sensitivity to oxidants is complex and context dependent (Prestes et al., 2020). Here, given correlated regulation of heme and OXPHOS pathways in the clinical groups C–E, activity of these modules may be interrelated and possibly together reflect dysfunctional mitochondria. How heme and OXPHOS transcriptional programmes are linked on a molecular level cannot be inferred from our data. Erythroid cell activation has recently been detected in severe COVID-19 (Bernardes et al., 2020) and could also contribute to a heme transcriptional signature. However, the increase in heme metabolism in our cohort correlates strongly with falling haemoglobin, and reticulocytes in patients in groups C, D, and E are low, suggesting suppression rather than activation of erythropoiesis. Understanding the mechanism linking metabolic dysregulation to persistent immune pathology in COVID-19 will require further study over longer disease courses.

Limitation of study

Many of the abnormalities we have observed in COVID-19 might also be features of other severe viral infections. To identify which are COVID specific will require a comparison with an appropriate disease control group. Sample size is critical when studying heterogeneous disease, and severe COVID-19 falls into this category. While the cohorts described here are comparable in size with other large detailed immunophenotyping studies, larger ones would nonetheless increase our power. In particular, validation of the prognosis prediction models described here is required in large independent cohorts. Very early features of the immune response are associated with disease outcome; exploring these during the period between infection and symptom onset will be important for understanding disease progression but presents major practical difficulties. Continued follow-up of patients will be needed to determine the persistence of abnormalities still observed at late time points. Finally, because our patients were recruited during the first pandemic wave, a follow-up study examining the immune response to new SARS-CoV-2 strains with different virulence could be informative.

STAR★METHODS

Detailed methods are provided in the online version of this paper and include the following:

- KEY RESOURCES TABLE
- RESOURCE AVAILABILITY
  - ○ Lead contact
  - ○ Materials availability
  - ○ Data and code availability

- EXPERIMENTAL MODEL AND SUBJECT DETAILS
  - Human subjects
  - Cell lines
  - Virus
- METHOD DETAILS
  - Clinical data collection
  - Peripheral blood mononuclear cell preparation and flow immunophenotyping
  - CyTOF
  - Reticulocyte counts
  - Complement
  - CRP
  - Cytokines
  - IFN-μ FLUOROSPOT assays
  - SARS-CoV-2 serology
  - SARS-CoV-2 neutralisation assay
  - Whole blood bulk RNA-Seq
  - Single cell RNA-seq
- QUANTIFICATION AND STATISTICAL ANALYSIS

SUPPLEMENTAL INFORMATION

Supplemental information can be found online at https://doi.org/10.1016/j.immuni.2021.05.010.

CONSORTIA

The members of the Cambridge Institute of Therapeutic Immunology and Infectious Disease-National Institute of Health Research (CITIID-NIHR) COVID BioResource Collaboration are John Allison, Ali Ansaripour, Stephen Baker, Laura Bergamaschi, Ariana Betancourt, Sze-How Bong, Georgie Bower, John R. Bradley, Ashlea Bucke, Ben Bullman, Katherine Bunclark, Helen Butcher, Jo Calder, Laura Canna, Daniela Caputo, Debbie Clapham-Riley, Chiara Cossetti, Jerome D. Coudert, Eckart M.D.D. De Bie, Aloka De Sa, Eleanor Dewhurst, Giovanni di Stefano, Jason Domingo, Gordon Dougan, Benjamin J. Dunmore, Anne Elmer, Madeline Epping, Codie Fahey, Stuart Fawke, Stewart Fuller, Anita Furlong, Nick Gleadall, Ian G Goodfellow, Stefan Gräf, Barbara Graves, Jennifer Gray, Richard Grenfell, Ravindra K. Gupta, Julie Harris, Christoph Hess, Sarah Hewitt, Andrew Hinch, Josh Hodgson, Elaine Holmes, Christopher Huang, Oisín Huhn, Kelvin Hunter, Tasmin Ivers, Sarah Jackson, Isobel Jarvis, Emma Jones, Jane Kennet, Sherly Jose, Masa Josipovic, Mary Kasanicki, Nathalie Kingston,Jenny Kourampa, Elisa Laurenti, Ekaterina Legchenko, Paul J. Lehner, Emma Le Gresley, Daniel Lewis, Rachel Linger, Paul A. Lyons, Michael Mackay, John C. Marioni, Jimmy Marsden, Jennifer Martin, Cecilia Matara, Nicholas J. Matheson, Anne Meadows, Sarah Meloy, Nicole Mende, Federica Mescia, Alice Michael, Rachel Michel,Lucy Mwaura, Francesca Muldoon, Francesca Nice, Criona O'Brien, Ciara O'Donnell, Georgina Okecha, Ommar Omarjee, Nigel Ovington, Willem H. Owehand, Sofia Papadia, Caroline Patterson, Marianne Perera, Isabel Phelan, Linda Pointon, Petra Polgarova, Gary Polwarth, Nicole Pond, Jane Price, Cherry Publico, Rebecca Rastall, Carla Ribeiro, Nathan Richoz, Veronika Romashova, Sabrina Rossi, Jane Rowlands, Valentina Ruffolo, Caroline Saunders, Natalia Savinykh Yarkoni, Rahul Sharma, Joy Shih, Mayurun Selvan, Kenneth G.C. Smith, Sarah Spencer, Luca Stefanucci, Hannah Stark, Jonathan Stephens, Kathleen E Stirrups, Mateusz Strezlecki, Charlotte Summers, Rachel Sutcliffe, James E.D. Thaventhiran,Tobias Tilly, Zhen Tong, Hugo Tordesillas, Carmen Treacy, Mark Toshner, Paul Townsend, Lori Turner, Neil Walker, Jennifer Webster, Michael P. Weekes, Nicola K. Wilson, Jennifer Wood, Marta Wylot, and Cissy Yong.

ACKNOWLEDGMENTS

We thank all the patients and health care workers who consented to take part in this study. We are grateful to CVC Capital Partners, the Evelyn Trust (20/75), Addenbrooke's Charitable Trust, Cambridge University Hospitals (12/20A), the NIHR Cambridge Biomedical Research Centre, and the UKRI/NIHR through the UK Coronavirus Immunology Consortium (UK-CIC) for their financial support. Further support: K.G.C.S.: Wellcome Investigator Award (200871/Z/16/Z); M.P.W.: Wellcome Senior Clinical Research Fellowship (108070/Z/15/Z); C.H.: Wellcome COVID-19 Rapid Response DCF and the Fondation Botnar; N.M.: MRC (CSF MR/P008801/1), NHSBT (WPA15-02), and Addenbrooke's Charitable Trust, (grant ref. to 900239 NJM); I.G.G.: Wellcome Senior Fellowship and Wellcome grant (Ref: 207498/Z/17/Z); P.J.L.: Wellcome Trust Principal Research Fellowship (084957/Z/08/Z) and MRC (MR/V011561/1); J.K.N.: The Spinnaker Healthcare Research Trust and the McCusker Foundation as part of Australian National Phenome Centre. P.K. is the recipient of a Jacquot Research Entry Scholarship of the Royal Australasian College of Physicians Foundation. We would like to thank the NIHR Cambridge Clinical Research Facility outreach team for enrollment of patients; the NIHR Cambridge Biomedical Research Centre Cell Phenotyping Hub and the CRUK Cambridge Institute flow cytometry core facility for flow and mass cytometry; and the Cambridge NIHR BRC Stratified Medicine Core Laboratory NGS Hub (supported by an MRC Clinical Infrastructure Award) for RNA sequencing.

AUTHOR CONTRIBUTIONS

Conceptualization, K.G.C.S., J.R.B., P.A.L., and C.H.; Methodology, L.B., L.T., M.R.W., I.G.G., and R.D.; Software, K.H.; Validation, L.T.; Formal analysis, L.B., F.M., L.T., A.H., P.K., and H.R.; Data curation, F.M., A.H., P.K., and L.T.; Investigation, L.B., F.M., L.T., P.K., A.D.S., O.H., M.D.M., P.P.G., M.R.W., I.G.G., M.H., F.J.C., R.D., J.K.N., and E.J.M.T.; Visualization, A.H. and P.K.; Project administration, L.B., F.M., L.T., B.J.D., C.S., and A.E.; Funding acquisition, P.J.L., N.K., J.R.B., C.H., G.D., M.P.W., P.A.L., and K.G.C.S.; Supervision, J.R.B., P.A.L., and K.G.C.S.; Resources, A.M.P., M.T., M.P.W., and N.J.M.; Writing – original draft, K.G.C.S., P.A.L., P.J.L., and C.H.; Writing – review & editing, L.B., F.M., A.H., L.T., P.K., H.R., B.J.D., A.D.S., O.H., M.D.M., P.P.G., F.J.C., R.D., K.H., S.B., R.K.G., J.K.N., C.S., A.E., A.M.P., E.J.M.T., N.K., P.J.L., N.J.M., S.R., J.E.D.T., M.P.W., B.G., M.T., C.H., J.R.B., G.D., M.R.W., M.H., I.G.G., P.A.L., and K.G.C.S.

DECLARATION OF INTERESTS

The authors declare they have no competing interests. E.J.M. Toonen is an employee of Hycult Biotechnology b.v.

Received: December 21, 2020
Revised: March 13, 2021
Accepted: May 11, 2021
Published: May 16, 2021

REFERENCES

Akbari, P., Vuckovic, D., Jiang, T., Kundu, K., Kreuzhuber, R., Bao, E.L., Mayer, L., Collins, J.H., Downes, K., Georges, M., et al. (2020). Genetic Analyses of Blood Cell Structure for Biological and Pharmacological Inference. bioRxiv. https://doi.org/10.1101/2020.01.30.927483.

Antin, J.H., Emerson, S.G., Martin, P., Gadol, N., and Ault, K.A. (1986). Leu-1+ (CD5+) B cells. A major lymphoid subpopulation in human fetal spleen: phenotypic and functional studies. J. Immunol. *136*, 505–510.

Arunachalam, P.S., Wimmers, F., Mok, C.K.P., Perera, R.A.P.M., Scott, M., Hagan, T., Sigal, N., Feng, Y., Bristow, L., Tak-Yin Tsang, O., et al. (2020). Systems biological assessment of immunity to mild versus severe COVID-19 infection in humans. Science *369*, 1210–1220.

Banchereau, R., Cepika, A.M., Banchereau, J., and Pascual, V. (2017). Understanding Human Autoimmunity and Autoinflammation Through Transcriptomics. Annu. Rev. Immunol. *35*, 337–370.

Bantug, G.R., Galluzzi, L., Kroemer, G., and Hess, C. (2018). The spectrum of T cell metabolism in health and disease. Nat. Rev. Immunol. *18*, 19–34.

Berg, R.E., Crossley, E., Murray, S., and Forman, J. (2003). Memory CD8+ T cells provide innate immune protection against Listeria monocytogenes in the absence of cognate antigen. J. Exp. Med. *198*, 1583–1593.

CellPress
OPEN ACCESS

Bernardes, J.P., Mishra, N., Tran, F., Bahmer, T., Best, L., Blase, J.I., Bordoni, D., Franzenburg, J., Geisen, U., Josephs-Spaulding, J., et al. (2020). Longitudinal Multi-omics Analyses Identify Responses of Megakaryocytes, Erythroid Cells, and Plasmablasts as Hallmarks of Severe COVID-19. Immunity *53*, 1296–1314.

Boes, M., Prodeus, A.P., Schmidt, T., Carroll, M.C., and Chen, J. (1998). A critical role of natural immunoglobulin M in immediate defense against systemic bacterial infection. J. Exp. Med. *188*, 2381–2386.

Borchers, M.T., Wesselkamper, S.C., Curull, V., Ramirez-Sarmiento, A., Sánchez-Font, A., Garcia-Aymerich, J., Coronell, C., Lloreta, J., Agusti, A.G., Gea, J., et al. (2009). Sustained CTL activation by murine pulmonary epithelial cells promotes the development of COPD-like disease. J. Clin. Invest. *119*, 636–649.

Carissimo, G., Xu, W., Kwok, I., Abdad, M.Y., Chan, Y.H., Fong, S.W., Puan, K.J., Lee, C.Y., Yeo, N.K., Amrun, S.N., et al. (2020). Whole blood immunophenotyping uncovers immature neutrophil-to-VD2 T-cell ratio as an early marker for severe COVID-19. Nat. Commun. *11*, 5243.

Cella, M., Jarrossay, D., Facchetti, F., Alebardi, O., Nakajima, H., Lanzavecchia, A., and Colonna, M. (1999). Plasmacytoid monocytes migrate to inflamed lymph nodes and produce large amounts of type I interferon. Nat. Med. *5*, 919–923.

Chen, Z., and John Wherry, E. (2020). T cell responses in patients with COVID-19. Nat. Rev. Immunol. *20*, 529–536.

Chen, E.Y., Tan, C.M., Kou, Y., Duan, Q., Wang, Z., Meirelles, G.V., Clark, N.R., and Ma'ayan, A. (2013). Enrichr: interactive and collaborative HTML5 gene list enrichment analysis tool. BMC Bioinformatics *14*, 128.

Cheng, X., Liu, Y.-M., Li, H., Zhang, X., Lei, F., Qin, J.-J., Chen, Z., Deng, K.-Q., Lin, L., Chen, M.-M., et al. (2020). Metformin Is Associated with Higher Incidence of Acidosis, but Not Mortality, in Individuals with COVID-19 and Pre-existing Type 2 Diabetes. Cell Metab. *32*, 537–547.

Collier, D.A., Assennato, S.M., Warne, B., Sithole, N., Sharrocks, K., Ritchie, A., Ravji, P., Routledge, M., Sparkes, D., Skittrall, J., et al.; CITIID-NIHR COVID BioResource Collaboration (2020). Point of Care Nucleic Acid Testing for SARS-CoV-2 in Hospitalized Patients: A Clinical Validation Trial and Implementation Study. Cell Rep. Med. *1*, 100062.

Daly, J.L., Simonetti, B., Klein, K., Chen, K.E., Williamson, M.K., Antón-Plágaro, C., Shoemark, D.K., Simón-Gracia, L., Bauer, M., Hollandi, R., et al. (2020). Neuropilin-1 is a host factor for SARS-CoV-2 infection. Science *370*, 861–865.

Del Valle, D.M., Kim-Schulze, S., Huang, H.H., Beckmann, N.D., Nirenberg, S., Wang, B., Lavin, Y., Swartz, T.H., Madduri, D., Stock, A., et al. (2020). An inflammatory cytokine signature predicts COVID-19 severity and survival. Nat. Med. *26*, 1636–1643.

Dobin, A., Davis, C.A., Schlesinger, F., Drenkow, J., Zaleski, C., Jha, S., Batut, P., Chaisson, M., and Gingeras, T.R. (2013). STAR: ultrafast universal RNA-seq aligner. Bioinformatics *29*, 15–21.

Espéli, M., Bashford-Rogers, R., Sowerby, J.M., Alouche, N., Wong, L., Denton, A.E., Linterman, M.A., and Smith, K.G.C. (2019). FcγRIIb differentially regulates pre-immune and germinal center B cell tolerance in mouse and human. Nat. Commun. *10*, 1970.

Fox, A., Le, N.M., Horby, P., van Doorn, H.R., Nguyen, V.T., Nguyen, H.H., Nguyen, T.C., Vu, D.P., Nguyen, M.H., Diep, N.T., et al. (2012). Severe pandemic H1N1 2009 infection is associated with transient NK and T deficiency and aberrant CD8 responses. PLoS ONE *7*, e31535.

Gerber, P.P., Duncan, L.M., Greenwood, E.J.D., Marelli, S., Naamati, A., Teixeira-Silva, A., Crozier, T.W.M., Gabaev, I., Zhan, J.R., Protasio, A.V., et al. (2021). Protease-activatable biosensors of SARS-CoV-2 infection for cell-based drug, neutralisation and virological assays. bioRxiv. https://doi.org/10.1101/2021.03.22.435957.

Groh, V., Bruhl, A., El-Gabalawy, H., Nelson, J.L., and Spies, T. (2003). Stimulation of T cell autoreactivity by anomalous expression of NKG2D and its MIC ligands in rheumatoid arthritis. Proc. Natl. Acad. Sci. USA *100*, 9452–9457.

Gu, Z., Eils, R., and Schlesner, M. (2016). Complex heatmaps reveal patterns and correlations in multidimensional genomic data. Bioinformatics *32*, 2847–2849.

Haas, K.M., Poe, J.C., Steeber, D.A., and Tedder, T.F. (2005). B-1a and B-1b cells exhibit distinct developmental requirements and have unique functional roles in innate and adaptive immunity to S. pneumoniae. Immunity *23*, 7–18.

Hadjadj, J., Yatim, N., Barnabei, L., Corneau, A., Boussier, J., Smith, N., Péré, H., Charbit, B., Bondet, V., Chenevier-Gobeaux, C., et al. (2020). Impaired type I interferon activity and inflammatory responses in severe COVID-19 patients. Science *369*, 718–724.

Hirsch, R.L., and Johnson, K.P. (1986). The effects of long-term administration of recombinant alpha-2 interferon on lymphocyte subsets, proliferation, and suppressor cell function in multiple sclerosis. J. Interferon Res. *6*, 171–177.

Holzapfel, K.L., Tyznik, A.J., Kronenberg, M., and Hogquist, K.A. (2014). Antigen-dependent versus -independent activation of invariant NKT cells during infection. J. Immunol. *192*, 5490–5498.

Horns, F., Dekker, C.L., and Quake, S.R. (2020). Memory B Cell Activation, Broad Anti-influenza Antibodies, and Bystander Activation Revealed by Single-Cell Transcriptomics. Cell Rep. *30*, 905–913.

Kaech, S.M., Tan, J.T., Wherry, E.J., Konieczny, B.T., Surh, C.D., and Ahmed, R. (2003). Selective expression of the interleukin 7 receptor identifies effector CD8 T cells that give rise to long-lived memory cells. Nat. Immunol. *4*, 1191–1198.

Kamphuis, E., Junt, T., Waibler, Z., Forster, R., and Kalinke, U. (2006). Type I interferons directly regulate lymphocyte recirculation and cause transient blood lymphopenia. Blood *108*, 3253–3261.

Kim, J., Chang, D.Y., Lee, H.W., Lee, H., Kim, J.H., Sung, P.S., Kim, K.H., Hong, S.H., Kang, W., Lee, J., et al. (2018). Innate-like Cytotoxic Function of Bystander-Activated CD8(+) T Cells Is Associated with Liver Injury in Acute Hepatitis A. Immunity *48*, 161–173.

Kim, D., Paggi, J.M., Park, C., Bennett, C., and Salzberg, S.L. (2019). Graph-based genome alignment and genotyping with HISAT2 and HISAT-genotype. Nat. Biotechnol. *37*, 907–915.

Krummel, M.F., Mahale, J.N., Uhl, L.F.K., Hardison, E.A., Mujal, A.M., Mazet, J.M., Weber, R.J., Gartner, Z.J., and Gérard, A. (2018). Paracrine costimulation of IFN-γ signaling by integrins modulates CD8 T cell differentiation. Proc. Natl. Acad. Sci. USA *115*, 11585–11590.

Kuri-Cervantes, L., Pampena, M.B., Meng, W., Rosenfeld, A.M., Ittner, C.A.G., Weisman, A.R., Agyekum, R.S., Mathew, D., Baxter, A.E., Vella, L.A., et al. (2020). Comprehensive mapping of immune perturbations associated with severe COVID-19. Sci. Immunol. *5*.

Laing, A.G., Lorenc, A., Del Molino Del Barrio, I., Das, A., Fish, M., Monin, L., Muñoz-Ruiz, M., McKenzie, D.R., Hayday, T.S., Francos-Quijorna, I., et al. (2020). A dynamic COVID-19 immune signature includes associations with poor prognosis. Nat. Med. *26*, 1623–1635.

Langfelder, P., and Horvath, S. (2008). WGCNA: an R package for weighted correlation network analysis. BMC Bioinformatics *9*, 559.

Law, C.W., Chen, Y., Shi, W., and Smyth, G.K. (2014). voom: Precision weights unlock linear model analysis tools for RNA-seq read counts. Genome Biol. *15*, R29.

Liao, M., Liu, Y., Yuan, J., Wen, Y., Xu, G., Zhao, J., Cheng, L., Li, J., Wang, X., Wang, F., et al. (2020). Single-cell landscape of bronchoalveolar immune cells in patients with COVID-19. Nat. Med. *26*, 842–844.

Liberzon, A., Birger, C., Thorvaldsdóttir, H., Ghandi, M., Mesirov, J.P., and Tamayo, P. (2015). The Molecular Signatures Database (MSigDB) hallmark gene set collection. Cell Syst. *1*, 417–425.

Lichtner, M., Mastroianni, C.M., Rossi, R., Russo, G., Belvisi, V., Marocco, R., Mascia, C., Del Borgo, C., Mengoni, F., Sauzullo, I., et al. (2011). Severe and persistent depletion of circulating plasmacytoid dendritic cells in patients with 2009 pandemic H1N1 infection. PLoS ONE *6*, e19872.

Lucas, C., Wong, P., Klein, J., Castro, T.B.R., Silva, J., Sundaram, M., Ellingson, M.K., Mao, T., Oh, J.E., Israelow, B., et al.; Yale IMPACT Team (2020). Longitudinal analyses reveal immunological misfiring in severe COVID-19. Nature *584*, 463–469.

Mann, E.R., Menon, M., Knight, S.B., Konkel, J.E., Jagger, C., Shaw, T.N., Krishnan, S., Rattray, M., Ustianowski, A., Bakerly, N.D., et al.; NIHR Respiratory TRC; CIRCO (2020). Longitudinal immune profiling reveals key myeloid signatures associated with COVID-19. Sci. Immunol. *5*.

Mathew, D., Giles, J.R., Baxter, A.E., Oldridge, D.A., Greenplate, A.R., Wu, J.E., Alanio, C., Kuri-Cervantes, L., Pampena, M.B., D'Andrea, K., et al.; UPenn COVID Processing Unit (2020). Deep immune profiling of COVID-19 patients reveals distinct immunotypes with therapeutic implications. Science *369*.

Maurice, N.J., McElrath, M.J., Andersen-Nissen, E., Frahm, N., and Prlic, M. (2019). CXCR3 enables recruitment and site-specific bystander activation of memory CD8^{+} T cells. Nat. Commun. *10*, 4987.

Maurice, N.J., Taber, A.K., and Prlic, M. (2021). The Ugly Duckling Turned to Swan: A Change in Perception of Bystander-Activated Memory CD8 T Cells. J. Immunol. *206*, 455–462.

Menzies, S.A., Volkmar, N., van den Boomen, D.J., Timms, R.T., Dickson, A.S., Nathan, J.A., and Lehner, P.J. (2018). The sterol-responsive RNF145 E3 ubiquitin ligase mediates the degradation of HMG-CoA reductase together with gp78 and Hrd1. eLife *7*.

Meresse, B., Chen, Z., Ciszewski, C., Tretiakova, M., Bhagat, G., Krausz, T.N., Raulet, D.H., Lanier, L.L., Groh, V., Spies, T., et al. (2004). Coordinated induction by IL15 of a TCR-independent NKG2D signaling pathway converts CTL into lymphokine-activated killer cells in celiac disease. Immunity *21*, 357–366.

Miles, A.L., Burr, S.P., Grice, G.L., and Nathan, J.A. (2017). The vacuolar-ATPase complex and assembly factors, TMEM199 and CCDC115, control HIF1α prolyl hydroxylation by regulating cellular iron levels. eLife *6*.

Mills, E.L., Kelly, B., and O'Neill, L.A.J. (2017). Mitochondria are the powerhouses of immunity. Nat. Immunol. *18*, 488–498.

Mlcochova, P., Collier, D., Ritchie, A., Assennato, S.M., Hosmillo, M., Goel, N., Meng, B., Chatterjee, K., Mendoza, V., Temperton, N., et al. (2020). Combined point of care nucleic acid and antibody testing for SARS-CoV-2 following emergence of D614G Spike Variant. Cell Rep. Med. *1*, 100099.

Nathan, C., and Cunningham-Bussel, A. (2013). Beyond oxidative stress: an immunologist's guide to reactive oxygen species. Nat. Rev. Immunol. *13*, 349–361.

Ochsenbein, A.F., Fehr, T., Lutz, C., Suter, M., Brombacher, F., Hengartner, H., and Zinkernagel, R.M. (1999). Control of early viral and bacterial distribution and disease by natural antibodies. Science *286*, 2156–2159.

Pairo-Castineira, E., Clohisey, S., Klaric, L., Bretherick, A.D., Rawlik, K., Pasko, D., Walker, S., Parkinson, N., Head Fourman, M., Russell, C.D., et al. (2020). Genetic mechanisms of critical illness in COVID-19. Nature *591*, 92–98.

Patterson, E.I., Prince, T., Anderson, E.R., Casas-Sanchez, A., Smith, S.L., Cansado-Utrilla, C., Solomon, T., Griffiths, M.J., Acosta-Serrano, Á., Turtle, L., and Hughes, G.L. (2020). Methods of Inactivation of SARS-CoV-2 for Downstream Biological Assays. J. Infect. Dis. *222*, 1462–1467.

Pinheiro, J., Bates, D., DebRoy, S., Sarkar, D., Heisterkamp, S., Van Willigen, B., and Ranke, J.EISPACKR-core (2021). Linear and Nonlinear Mixed Effects Models (R Foundation for Statistical Computing).

Prestes, E.B., Alves, L.S., Rodrigues, D.A.S., Dutra, F.F., Fernandez, P.L., Paiva, C.N., Kagan, J.C., and Bozza, M.T. (2020). Mitochondrial Reactive Oxygen Species Participate in Signaling Triggered by Heme in Macrophages and upon Hemolysis. J. Immunol. *205*, 2795–2805.

Pujadas, E., Chaudhry, F., McBride, R., Richter, F., Zhao, S., Wajnberg, A., Nadkarni, G., Glicksberg, B.S., Houldsworth, J., and Cordon-Cardo, C. (2020). SARS-CoV-2 viral load predicts COVID-19 mortality. Lancet Respir. Med. *8*, e70.

R Core Team (2015). R: A language and environment for statistical computing (Vienna, Austria: R Foundation for Statistical Computing).

Ribeiro, S.T., Ribot, J.C., and Silva-Santos, B. (2015). Five Layers of Receptor Signaling in $\gamma\delta$ T-Cell Differentiation and Activation. Front. Immunol. *6*, 15.

Rivett, L., Sridhar, S., Sparkes, D., Routledge, M., Jones, N.K., Forrest, S., Young, J., Pereira-Dias, J., Hamilton, W.L., Ferris, M., et al.; CITIID-NIHR COVID-19 BioResource Collaboration (2020). Screening of healthcare workers for SARS-CoV-2 highlights the role of asymptomatic carriage in COVID-19 transmission. eLife *9*.

Robinson, M.D., McCarthy, D.J., and Smyth, G.K. (2010). edgeR: a Bioconductor package for differential expression analysis of digital gene expression data. Bioinformatics *26*, 139–140.

Rohart, F., Gautier, B., Singh, A., and Lê Cao, K.A. (2017). mixOmics: An R package for 'omics feature selection and multiple data integration. PLoS Comput. Biol. *13*, e1005752.

Russell, C.D., Unger, S.A., Walton, M., and Schwarze, J. (2017). The Human Immune Response to Respiratory Syncytial Virus Infection. Clin. Microbiol. Rev. *30*, 481–502.

Sanderson, N.S., Zimmermann, M., Eilinger, L., Gubser, C., Schaeren-Wiemers, N., Lindberg, R.L., Dougan, S.K., Ploegh, H.L., Kappos, L., and Derfuss, T. (2017). Cocapture of cognate and bystander antigens can activate autoreactive B cells. Proc. Natl. Acad. Sci. USA *114*, 734–739.

Shen-Orr, S.S., Furman, D., Kidd, B.A., Hadad, F., Lovelace, P., Huang, Y.W., Rosenberg-Hasson, Y., Mackey, S., Grisar, F.A., Pickman, Y., et al. (2016). Defective Signaling in the JAK-STAT Pathway Tracks with Chronic Inflammation and Cardiovascular Risk in Aging Humans. Cell Syst. *3*, 374–384.

Slifka, M.K., and Whitton, J.L. (2000). Activated and memory CD8+ T cells can be distinguished by their cytokine profiles and phenotypic markers. J. Immunol. *164*, 208–216.

Soudja, S.M., Chandrabos, C., Yakob, E., Veenstra, M., Palliser, D., and Lauvau, G. (2014). Memory-T-cell-derived interferon-γ instructs potent innate cell activation for protective immunity. Immunity *40*, 974–988.

Stephenson, E., Reynolds, G., Botting, R.A., Calero-Nieto, F.J., Morgan, M., Tuong, Z.K., Bach, K., Sungnak, W., Worlock, K.B., Yoshida, M., et al. (2021). The cellular immune response to COVID-19 deciphered by single cell multi-omics across three UK centres. medRxiv. https://doi.org/10.1101/2021.01.13.21249725.

Su, Y., Chen, D., Yuan, D., Lausted, C., Choi, J., Dai, C.L., Voillet, V., Duvvuri, V.R., Scherler, K., Troisch, P., et al. (2020). Multi-omics resolves a sharp disease-state shift between mild and moderate COVID-19. Cell *183*, 1479–1495.

Subramanian, A., Tamayo, P., Mootha, V.K., Mukherjee, S., Ebert, B.L., Gillette, M.A., Paulovich, A., Pomeroy, S.L., Golub, T.R., Lander, E.S., and Mesirov, J.P. (2005). Gene set enrichment analysis: a knowledge-based approach for interpreting genome-wide expression profiles. Proc. Natl. Acad. Sci. USA *102*, 15545–15550.

Tough, D.F., Borrow, P., and Sprent, J. (1996). Induction of bystander T cell proliferation by viruses and type I interferon in vivo. Science *272*, 1947–1950.

Ussher, J.E., Willberg, C.B., and Klenerman, P. (2018). MAIT cells and viruses. Immunol. Cell Biol. *96*, 630–641.

Wang, D., Hu, B., Hu, C., Zhu, F., Liu, X., Zhang, J., Wang, B., Xiang, H., Cheng, Z., Xiong, Y., et al. (2020). Clinical Characteristics of 138 Hospitalized Patients With 2019 Novel Coronavirus-Infected Pneumonia in Wuhan, China. JAMA *323*, 1061–1069.

Warnes, G.R., Bolker, B., Bonebakker, L., Gentleman, R., Huber, W., Liaw, A., Lumley, T., Maechler, M., Magnusson, A., Moeller, S., et al. (2020). gplots: Various R Programming Tools for Plotting Data. https://github.com/talgalili/gplots.

Wauters, E., Van Mol, P., Garg, A.D., Jansen, S., Van Herck, Y., Vanderbeke, L., Bassez, A., Boeckx, B., Malengier-Devlies, B., Timmerman, A., et al.; CONTAGIOUS collaborators (2021). Discriminating mild from critical COVID-19 by innate and adaptive immune single-cell profiling of bronchoalveolar lavages. Cell Res. *31*, 272–290.

Weiskopf, D., Weinberger, B., and Grubeck-Loebenstein, B. (2009). The aging of the immune system. Transpl. Int. *22*, 1041–1050.

Wrammert, J., Onlamoon, N., Akondy, R.S., Perng, G.C., Polsrila, K., Chandele, A., Kwissa, M., Pulendran, B., Wilson, P.C., Wittawatmongkol, O., et al. (2012). Rapid and massive virus-specific plasmablast responses during acute dengue virus infection in humans. J. Virol. *86*, 2911–2918.

Xiong, X., Qu, K., Ciazynska, K.A., Hosmillo, M., Carter, A.P., Ebrahimi, S., Ke, Z., Scheres, S.H.W., Bergamaschi, L., Grice, G.L., et al.; CITIID-NIHR COVID-19 BioResource Collaboration (2020). A thermostable, closed SARS-CoV-2 spike protein trimer. Nat. Struct. Mol. Biol. *27*, 934–941.

Zafranskaya, M., Oschmann, P., Engel, R., Weishaupt, A., van Noort, J.M., Jomaa, H., and Eberl, M. (2007). Interferon-beta therapy reduces CD4+ and CD8+ T-cell reactivity in multiple sclerosis. Immunology *121*, 29–39.

Zhang, Q., Bastard, P., Liu, Z., Le Pen, J., Moncada-Velez, M., Chen, J., Ogishi, M., Sabli, I.K.D., Hodeib, S., Korol, C., et al. (2020). Inborn errors of type I IFN immunity in patients with life-threatening COVID-19. Science *370*, eabd4570.

Zhou, F., Yu, T., Du, R., Fan, G., Liu, Y., Liu, Z., Xiang, J., Wang, Y., Song, B., Gu, X., et al. (2020). Clinical course and risk factors for mortality of adult inpatients with COVID-19 in Wuhan, China: a retrospective cohort study. Lancet *395*, 1054–1062.

STAR★METHODS

KEY RESOURCES TABLE

REAGENT or RESOURCE	SOURCE	IDENTIFIER
Antibodies		
Anti-human CCR4 PEVio770 (REA279)	Miltenyi	RRID:AB_2655909
Anti-human CCR5 AF647 (HEK/1/85a)	Biolegend	RRID:AB_528760
Anti-human CCR6 PECy7 (G034E3)	BioLegend	RRID:AB_10916518
Anti-human CCR7 BV650 (G043H7)	BioLegend	RRID:AB_2563867
Anti-human CCR7 APC-fire (G043H7)	BioLegend	RRID:AB_2750147
Anti-human CD11c AF700 (B-ly6)	BD	RRID:AB_10612006
Anti-human CD123 BV786 (7G3)	BD	RRID:AB_2738662
Anti-human CD127 PECy5 (eBioRDR5)	Thermo	RRID:AB_2043801
Anti-human CD127 PerCP efluor710 (eBioRDR5)	Thermo	RRID:AB_2762464
Anti-human CD14 BV711 (MjP9)	BD	RRID:AB_2744290
Anti-human CD14 BV510 (63D3)	BioLegend	RRID:AB_2716229
Anti-human CD141 BV605 (1A4)	BD	RRID:AB_2740151
Anti-human CD15 BV510 (W6D3)	BioLegend	RRID:AB_2563400
Anti-human CD16 BUV496 (3G8)	BD	RRID:AB_2870224
Anti-human CD161 PE (HP-3G10)	BioLegend	RRID:AB_1501083
Anti-human CD163 PE-CF594 (GHI/61)	BD	RRID:AB_2737711
Anti-human CD19 BUV496 (SJ25C1)	BD	RRID:AB_2870221
Anti-human CD193 BV510 (5E8)	BioLegend	RRID:AB_2571977
Anti-human CD1c AF647 (F10/21A3)	BD	RRID:AB_2744318
Anti-human CD20 BUV805 (2H7)	BD	RRID:AB_2870192
Anti-human CD24 BB700 (ML5)	BD	RRID:AB_2744333
Anti-human CD25 PE (BC96)	Thermo	RRID:AB_1659682
Anti-human CD27 BV711 (O323)	BioLegend	RRID:AB_2563809
Anti-human CD28 BV785 (CD28.2)	BioLegend	RRID:AB_2632607
Anti-human CD28 BUV805 (L293)	BD	RRID:AB_2872889
Anti-human CD3 BUV395 (SK7)	BD	RRID:AB_2744382
Anti-human CD3 BUV661 (UCHT1)	BD	RRID:AB_2870239
Anti-human CD3 BV510 (UCHT1)	BioLegend	RRID:AB_2563468
Anti-human CD303 PE-Vio770 (REA693)	Miltenyi	RRID:AB_2657198
Anti-human CD304 PE (U21-1283)	BD	RRID:AB_2744361
Anti-human CD32 BB700 (FLI8.26)	BD	RRID:AB_2871430
Anti-human CD38 BUV661 (HIT2)	BD	RRID:AB_2870242
Anti-human CD39 BV421 (A1)	BioLegend	RRID:AB_2564575
Anti-human CD39 APC-fire (A1)	BioLegend	RRID:AB_2650839
Anti-human CD4 BUV496 (SK3)	BD	RRID:AB_2870220
Anti-human CD40 BUV395 (5C3)	BD	RRID:AB_2739110
Anti-human CD45 BUV805 (HI30)	BD	RRID:AB_2870179
Anti-human CD45RA BV570 (HI100)	BioLegend	RRID:AB_2563813
Anti-human CD45RA BUV805 (HI100)	BD	RRID:AB_2871317
Anti-human CD56 FITC (MEM188)	Thermo	RRID:AB_10372519
Anti-human CD69 BV650 (FN50)	BioLegend	RRID:AB_2563158
Anti-human CD71 BV650 (CY1G4)	BioLegend	RRID:AB_2687103
Anti-human CD73 Brilliant Violet 785™ (AD2)	BioLegend	RRID:AB_2687234
Anti-human CD73 BV785 (AD2)	BioLegend	RRID:AB_2687234

(Continued on next page)

Continued

REAGENT or RESOURCE	SOURCE	IDENTIFIER
Anti-human CD80 PECy5 (L307.4)	BD	RRID:AB_397239
Anti-human CD86 BUV737 (FUN-1)	BD	RRID:AB_2814790
Anti-human CD86 PECy7 (BU63)	BioLegend	RRID:AB_2728392
Anti-human CD8b BV480 (2ST8.5H7)	BD	RRID:AB_2743598
Anti-human CD95 BUV737 (DX2)	BD	RRID:AB_2870117
Anti-human CRTh2 PE-dazzle (BM16)	BioLegend	RRID:AB_2572053
Anti-human CXCR5 APC-R700 (RF8B2)	BD	RRID:AB_2739103
Anti-human FoxP3 APC (236A/E7)	Thermo	RRID:AB_10804651
Anti-human GLUT1 AF647 (202915)	BD	RRID:AB_2869787
Anti-human Helios Pedazzle (22F6)	BioLegend	RRID:AB_2565797
Anti-human HLA-DR APC-H7 (G46-6)	BD	RRID:AB_10611876
Anti-human HLADR BV605 (L243)	BioLegend	RRID:AB_2561913
Anti-human KLRG1 FITC (REA261)	Miltenyi	RRID:AB_2652570
Anti-human PD-1 BV421 (EH12.2H7)	BioLegend	RRID:AB_10960742
Anti-human TCR Vg9 AF700 (B3)	Biolegend	RRID:AB_2814207
Anti-human TCR-DV1 PECy7 (TS8.2)	Thermo	RRID:AB_2762454
Anti-human TCR-DV2 PerCPCy5.5 (B6)	BioLegend	RRID:AB_2687330
Anti-human TCRgd BUV737 (11F2)	BD	RRID:AB_2872944
Anti-human TCRV7.2 BV711 (3C10)	BioLegend	RRID:AB_2629680
Anti-human Vb11 APCVio770 (REA559)	Miltenyi	RRID:AB_2653747
Zombie Aqua	BioLegend	Cat#423101
Zombie Yellow	BioLegend	Cat#423103
Anti-human CD3 FITC (UCHT1)	BioLegend	RRID:AB_2562046
Anti-human CD4 PE (RPA-T4)	BioLegend	RRID:AB_2562053
Anti-human CD8a PerCP-Cy5.5 (RPA-8a)	BioLegend	RRID:AB_1575074
Fixable Far Red Dead Cell Stain Kit	Thermo	Cat#423109
Chemicals, peptides, and recombinant proteins		
Spike SARS-CoV-2 peptide prot-S	Miltenyi Biotec	Cat#130-126-701
Spike SARS-CoV-2 peptide Prot-S1	Miltenyi Biotec	Cat#130-127-048
Nucleocapsid SARS-CoV-2 peptide	Miltenyi Biotec	Cat#130-126-699
Membrane SARS-CoV-2 peptide	Miltenyi Biotec	Cat#130-126-703
Staphylococcus Enterotoxin B (SEB)	Sigma Aldrich	Cat#S4881-1MG
Phytohaemagglutinin (PHA)	Sigma Aldrich	Cat#L1668-5MG
anti-CD3	Mabtech AB	Cat#3605-1-1000
Spike SARS-CoV-2 protein	Xiong et al., 2020	N/A
SARS-CoV-2 neutralisation assay	Gerber, et al., 2021	N/A
Critical commercial assays		
6-color TBNK Reagent with BD Trucount™ Tubes	BD	RRID:AB_2868707
Maxpar® Direct™ Immune Profiling Assay™	Fluidigm	Cat#201325
Human IFNg FLUOROSPOT	Mabtech AB	Cat#X-01A-10
hIFN-g HS LxPA MAG	R&D systems/ Biotechne	Cat#LHSCM285B
Human IL-1b Mag Bead Set	R&D systems/ Biotechne	Cat#LHSCM201
Human IL-6 Mag Bead Set	R&D systems/ Biotechne	Cat#LHSCM206
Human IL-10 Mag Bead Set	R&D systems/ Biotechne	Cat#LHSCM217
Human TNF-a Mag Bead Set	R&D systems/ Biotechne	Cat#LHSCM210
Base Kit, HS Cytokine A, Mag	R&D systems/ Biotechne	Cat#LHSCM000
Fluorocell™ RET	Sysmex Corporation	Cat#BN-337-547
C3a, Human, ELISA kit	Hycult Biotech	Cat#HK354
C3c, Human, ELISA kit	Hycult Biotech	Cat#HK368

(*Continued on next page*)

Continued		
REAGENT or RESOURCE	SOURCE	IDENTIFIER
TCC, Human, ELISA kit	Hycult Biotech	Cat#HK328
SMARTer® Stranded Total RNA-Seq v2 - Pico Input Mammalian kit	Takara	Cat#634414
Deposited data		
CITE-seq data	Stephenson et al., 2021, Cambridge cohort	Array Express:E-MTAB-10026
Absolute cell count, Whole blood RNA-seq, cytokine and complement measurements, SARS-CoV-2 specific antibody titers	This paper	https://www.covid19cellatlas.org/patient/citiid/
Flow cytometry data: B cell and Treg panels	This paper	Flow Repository:FR-FCM-Z3XQ
Flow cytometry data: Monocyte panel	This paper	Flow Repository:FR-FCM-Z3SR
Flow cytometry data: Conventional T cell panel	This paper	Flow Repository:FR-FCM-Z3ST;
Flow cytometry data: non-conventional T cell panel	This paper	Flow Repository:FR-FCM-Z3SS
Whole blood RNA-seq data	This paper	EGA:EGAS00001005332
Software and algorithms		
Maxpar® Pathsetter™ software v2.0.45	Verity Software House, Topsham, ME	N/A
FlowJo v10.2	FlowJo LLC	N/A
CyTOF Software v6.7.1016	Fluidigm	N/A
AID EliSpot v7 software	Autoimmun Diagnostika GmbH, Strasberg, Germany	N/A
FastQC v.0.11.8	Babraham Bioinformatics, UK	https://www.bioinformatics.babraham.ac.uk/projects/fastqc/
Trim_galore v.0.6.4	Babraham Bioinformatics, UK	https://www.bioinformatics.babraham.ac.uk/projects/trim_galore/
BBMap v.38.67	BBMap - Bushnell B.	https://sourceforge.net/projects/bbmap/
HISAT2 v.2.1.0	Kim et al., 2019	http://daehwankimlab.github.io/hisat2/
R	R Core Team, 2015	N/A
pathway-level information extractor	PLIER	http://gobie.csb.pitt.edu/PLIER
Gene set enrichment analysis (GSEA)	Broad Institute	https://www.gsea-msigdb.org/gsea/index.jsp
Other		
XN-1000 hematology analyzer	Sysmex	N/A
Symphony X-50	BD	N/A
Helios mass cytometer	Fluidigm	N/A
AID iSpot reader	Oxford Biosystems	N/A
Luminex	Bio-Plex, Bio-Rad, UK	N/A

RESOURCE AVAILABILITY

Lead contact

Further information and requests for resources and reagents should be directed to and will be fulfilled by the lead contact Prof Kenneth Smith (kgcs2@cam.ac.uk).

Materials availability

This study did not generate new unique reagents.

Data and code availability

The datasets generated during this study are available at NIHR CITIID COVID-19 Cohort (https://www.covid19cellatlas.org/patient/citiid/). In addition, whole blood RNA-seq data are available at European Genome-phenome Archive (EGA, ID:EGAS00001005332), and flow cytometry data are available at FLOW Repository (IDs: FR-FCM-Z3XQ, FR-FCM-Z3SR, FR-FCM-Z3ST, FR-FCM-Z3SS).

CITE-seq processed data are available to download from Array Express using accession number E-MTAB-10026.

EXPERIMENTAL MODEL AND SUBJECT DETAILS

Human subjects

Study participants were recruited between 31/3/2020 and 20/7/2020 from patients attending Addenbrooke's Hospital with a suspected or nucleic acid amplification test (NAAT) confirmed diagnosis of COVID-19 (including point of care testing (Collier et al., 2020; Mlcochova et al., 2020)), patients admitted to Royal Papworth Hospital NHS Foundation Trust or Cambridge and Peterborough Foundation Trust with a confirmed diagnosis of COVID-19, together with Health Care Workers identified through staff screening as PCR positive for SARS-CoV-2 (Rivett et al., 2020). Detailed information on the patients (age, gender, and clinical features) can be found in Table S1. Controls were recruited among hospital staff attending Addenbrooke's serology screening program, and selected to cover the whole age spectrum of COVID-19 positive study participants, across both genders. Only controls with negative serology results (45 out of 47) were subsequently included in the study. Recruitment of inpatients at Addenbrooke's Hospital and Health Care Workers was undertaken by the NIHR Cambridge Clinical Research Facility outreach team and the NIHR BioResource research nurse team. Ethical approval was obtained from the East of England – Cambridge Central Research Ethics Committee (''NIHR BioResource'' REC ref 17/EE/0025, and ''Genetic variation AND Altered Leucocyte Function in health and disease - GANDALF'' REC ref 08/H0308/176). All participants provided informed consent.

Inpatients were sampled at study entry, and then at regular intervals as long as they remained admitted to hospital (approximately weekly up to 4 weeks, and then every 2 weeks up to 12 weeks). Discharged patients were invited to provide a follow-up sample 4-8 weeks after study enrolment. Health care workers were sampled at study entry, and subsequently after approximately 2 and 4 weeks. At each time-point, blood samples were drawn in EDTA, sodium citrate, serum and PAXgene Blood RNA tubes (BD Biosciences) and processed by the CITIID-NIHR COVID BioResource Collaboration group (Document S1).

Cell lines

HEK293 T cells (a female cell line) were a kind gift from Paul Lehner, authenticated by STR profiling (Menzies et al., 2018; Miles et al., 2017). They were cultured in DMEM supplemented with 10% fetal calf serum (FCS), 100 units/mL penicillin, and 0.1 mg/ml streptomycin at 37 C in 5% CO2.

Virus

The virus used in this study was the clinical isolate SARS-CoV428 2/human/Liverpool/REMRQ0001/2020, a kind gift from Ian Goodfellow (University of Cambridge), isolated by Lance Turtle (University of Liverpool), and David Matthews and Andrew Davidson (University of Bristol) (Daly et al., 2020; Patterson et al., 2020).

METHOD DETAILS

Clinical data collection

Clinical data were retrospectively collected by review of medical charts and entered into spreadsheets or Castor EDC, a cloud-based clinical data management system. Available laboratory test results and administered in-patient medications were extracted from Epic electronic health records (Addenbrooke's Hospital) and from MetaVision ICU (RPH ITU). Data were merged from the various data sources using R version 3.6 and the R packages readr (1.3.1), openxlsx (4.1.4), dplyr (0.8.3), tidyr (1.0.2) and lubridate (1.7.4).

Health care workers were classified in 2 groups (A and B) according to whether they were asymptomatic (group A) or had possible COVID-19 symptoms (group B) at the time of PCR testing. For this purpose, new-onset fever (> 37.8 C), cough, loss of sense of smell, hoarseness, nasal discharge or congestion, shortness of breath, wheeze, headache, muscle aches, nausea, vomiting and diarrhea were considered as possible COVID-19 symptoms.

Participants in group A were further sub-grouped according to whether they were completely asymptomatic ($n = 8$), or had had any of the above COVID-19 symptoms before PCR testing ($n = 10$, median time from symptoms to COVID-19 PCR test 26 days, range 9-42 days).

Group B participants included both staff who were self-isolating because of COVID-19 symptoms ($n = 30$), and staff members who were reporting fit for duty, but had some symptoms that did not reach the threshold for self-isolation at that time ($n = 10$).

Hospital patients were assigned to 3 severity groups, mainly reflecting the maximum intensity of respiratory support for COVID-19 received during their hospital stay:

- group C: did not receive any supplemental oxygen. Five patients were discharged soon after initial diagnosis and assessment but followed up as part of the study.
- group D: received supplemental oxygen using low flow nasal prongs, simple face mask, Venturi mask or non-rebreather face mask.
- group E: received any of non-invasive ventilation (NIV), mechanical ventilation or ECMO. Patients who received supplemental oxygen (but no ventilation) and deceased in hospital were also assigned to group E.

In patients who were already established on home NIV for chronic respiratory failure, NIV delivered as per the home prescription (e.g., nocturnal) was not considered for the purpose of classification. Moreover, oxygen requirements that were clearly not related to

COVID-19 were also not considered for classification purposes. In particular, 2 cases who received low flow supplemental oxygen for non-COVID-19 indications (ascitic splinting in decompensated cirrhosis in one case, and recovery from anesthesia after orthopedic surgery in the other) were assigned to group C. Cases in group C were further sub-classified according to chest radiology results (X-ray and, if available, CT scan), as:

- abnormal radiology: chest X-ray/ CT scan showed changes compatible with COVID-19
- normal radiology: chest X-ray/ CT scan did not show any abnormality compatible with COVID-19 (reported as normal or showing lung changes diagnostic of conditions other than COVID-19).

Immunological parameters were analyzed according to time since onset of symptoms, or otherwise time since positive SARS-CoV-2 NAAT (in group A and in 4 asymptomatic patients in group C). Seven cases admitted to hospital for COVID-19 had no date of onset of symptoms documented in the medical records. In these cases, the date of onset of symptoms was estimated as follows: hospital admission date - median time from symptoms to hospital admission in patients admitted for COVID-19.

Following clinician review, 6 cases were considered not classifiable, due to complex concomitant pathologies that coexisted with COVID-19 and dominated the clinical picture, confounding the interpretation of clinical outcome. These cases were not included in any analyses; more details are reported in Table S2**.** Data S1 summarizes the timing of research samples and clinical trajectories for volunteers in severity groups C, D and E included in the analysis.

Peripheral blood mononuclear cell preparation and flow immunophenotyping

Each participant provided 27 mL of peripheral venous blood collected into 9 mL sodium citrate tube. Peripheral blood mononuclear cells (PBMCs) were isolated using Leucosep tubes (Greiner Bio-One) with Histopaque 1077 (Sigma) by centrifugation at 800x g for 15 min at room temperature. PBMCs at the interface were collected, rinsed twice with autoMACS running buffer (Miltenyi Biotech) and cryopreserved in FBS with 10% DMSO. All samples were processed within 4 h of collection.

Five distinct antibody cocktails (Data S4) were used to label approximately 10^6 PBMCs using standard methods. T regulatory cells were fixed and permeabilized following surface staining prior to the addition of intracellular antibodies. Samples were stored at 4°C and acquired within 4 h using a 5-laser BD Symphony X-50 flow cytometer. Single color compensation tubes (BD CompBeads) or cells were prepared for each of the fluorophores used and acquired at the start of each flow cytometer run.

For direct enumeration of T, B and NK cells, an aliquot of whole blood (50 µl) was added to BD TruCount tubes with 20µl- BD Multitest 6-color TBNK reagent (BD Biosciences) and processed as per the manufacturer's instructions.

Samples were gated in FlowJo v10.2 according to the schema set out in Data S4. The number of cells falling within each gate was recorded. For analysis, these were expressed as an absolute concentration of cells per ml, calculated using the proportions of daughter populations present within the parent population determined using the BD TruCountsystem.

CyTOF

The protocol used to isolate PBMCs led to an impaired recovery of the different monocyte populations, specifically intermediate and non-classical monocytes (Figure S2). To extend our analysis to these and other granulocyte populations we performed mass cytometric analysis on a subgroup of patients and healthy controls (249 samples). Briefly, whole blood samples (270µl) were stained using the Fluidigm Maxpar® Direct Immune Profiling Assay according to the manufacturer's instructions. Samples were cryopreserved at −80°C following staining and thawed for analysis within 4 weeks. Samples were acquired using a Fluidigm Helios mass cytometer and normalized using the CyTOF Software v6.7.1016. FCS files generated were analyzed using the Maxpar® Pathsetter software v2.0.45 (Verity Software House, Topsham, ME). Standard settings were used to generate immune cell frequencies for 37 immune cell populations. Absolute cell numbers were calculated using the proportions of these immune cell populations within the parent populations determined by BD TruCount.

Reticulocyte counts

Reticulocyte numbers were measured using a Sysmex XN-1000 hematology analyzer according to manufacturer instruction and as previously described (Akbari et al., 2020). Briefly, Sysmex technology uses three signals to define the physiological and structural properties of cells and to distinguish reticulocytes from the other blood cells: forward scatter, side scatter and side fluorescent light. These measurements rely on the similar electromagnetic radiation and fluid dynamics concepts of a flow cytometer; reticulocyte specific fluorescent probes are covered by a patent deposited by Sysmex Corporation (i.e., Fluorocell RET, cat# BN-337-547).

Reticulocyte Ratio:

$$RET\% = \frac{\text{Particle count} \in \text{reticulocyte zone}}{(\text{Particle count} \in \text{mature RBC zone} + \text{Particle count} \in \text{reticulocyte zone})} \times 100$$

Reticulocyte Count:

$$\text{RET\#} = \frac{\text{RET\%} \times \text{RBC}}{100}$$

Complement

Complement activation was assessed by measuring C3 activation products (C3a and C3c) together with the terminal complement complex (TCC) as an end product of the complement cascade. Concentrations of these complement components were measured in EDTA plasma from patients using commercially available enzyme-linked immunosorbent assays (ELISA) kits (HK354 (C3a), HK368 (C3c), HK328 (TCC), Hycult Biotech, Uden, the Netherlands) according to the manufacturer's protocols.

CRP

High sensitivity CRP was measured using the standard assay by the Core Biochemical Assay Laboratory (CBAL) at Cambridge University Hospitals NHS Foundation Trust.

Cytokines

IL-6, IL-10, IL-1β, TNF-α and IFN-γ were measured in serum from patients and HCs by high sensitivity Base Kit HS Cytokine A Mag (cat# LHSCM000, R&D Systems / Biotechne) on a Luminex analyzer (Bio-Plex, Bio-Rad, UK) as standard clinical assay performed by the Clinical Immunology Laboratory at the Department of Biochemistry and Immunology, Addenbrooke's Hospital Cambridge.

IFN-μ FLUOROSPOT assays

Frozen PBMCs were rapidly thawed, and the freezing medium was diluted into 10ml of TexMACS media (Miltenyi Biotech), centrifuged and resuspended in 10ml of fresh media with 10U /ml DNase (Benzonase, Merck-Millipore via Sigma-Aldrich), PBMCs were incubated at 37°C for 1 h, followed by centrifugation and resuspension in fresh media supplemented with 5% Human AB serum (Sigma Aldrich) before being counted. PBMCs were stained with 2μl of each antibody: anti-CD3- fluorescein isothiocyanate (FITC), clone UCHT1; anti-CD4- phycoerythrin (PE), clone RPA-T4; anti-CD8a- peridinin-chlorophyll protein - cyanine 5.5 (PerCP Cy5.5), clone RPA-8a (all BioLegend, London, UK), LIVE/DEAD Fixable Far Red Dead Cell Stain Kit (Thermo Fisher Scientific). PBMC phenotyping was performed on the BD Accuri C6 flow cytometer. Data were analyzed with FlowJo v10 (Becton Dickinson, Wokingham, UK). 1.5 to 2.5 × 10^5 PBMCs were incubated in pre-coated Fluorospot plates (Human IFN-γ FLUOROSPOT (Mabtech AB, Nacka Strand, Sweden)) in triplicate with peptide mixes specific for Spike, Nucleocapsid and Membrane proteins of SARS-CoV-2 (final peptide concentration 1μg/ml/peptide, Miltenyi Biotech) and an unstimulated and positive control mix (containing anti-CD3 (Mabtech AB), Staphylococcus Enterotoxin B (SEB), Phytohaemagglutinin (PHA) (all Sigma Aldrich)) at 37°C in a humidified CO_2 atmosphere for 48 h. The cells and medium were decanted from the plate and the assay developed following the manufacturer's instructions. Developed plates were read using an AID iSpot reader (Oxford Biosystems, Oxford, UK) and counted using AID EliSpot v7 software (Autoimmun Diagnostika GmbH, Strasberg, Germany). All data were then corrected for background cytokine production and expressed as SFU/Million $CD3^+$T cells.

SARS-CoV-2 serology

Quantification of Spike SARS-CoV-2 specific antibodies was performed by ELISA as described by Xiong X et al. (Xiong et al., 2020). Briefly, serum samples collected at time of enrolment in the study and at the 4-8 week follow-up visit were first screened for positivity and then antibody titers were determined by an end-point analysis. AUC values were calculated in R (3.6.3) using the flux (0.3-0) package. Kruskal–Wallis test was used to calculate p values among the different disease severities.

SARS-CoV-2 neutralisation assay

The virus used in this study was the clinical isolate SARS-CoV-2/human/Liverpool/REMRQ0001/2020, a kind gift from Ian Goodfellow (University of Cambridge), isolated by Lance Turtle (University of Liverpool) and David Matthews and Andrew Davidson (University of Bristol) (Daly et al., 2020; Patterson et al., 2020).

Sera were heat-inactivated at 56°C for 30 min, then frozen in aliquots at −80°C. Neutralising antibody titers at 50% inhibition (NT50s) were measured as previously described (Gerber et al., 2021).

In brief, HEK293T reporter cells expressing Renilla luciferase (Rluc) and SARS-CoV-2 Papain-like protease-activatable circularly permuted firefly luciferase (FFluc) were seeded in flat-bottomed 96-well plates. The next day, SARS-CoV-2 viral stock (MOI = 1) was pre-incubated with a 3-fold dilution series of each serum for 2 h at 37°C, then added to the cells. After 24 h, cells were lysed in Dual-Glo Luciferase Buffer (Promega) diluted 1:1 with PBS and 1% NP-40. Lysates were transferred to white half-area 96-well plates, and infectious virus quantitated as the ratio of FFluc/Rluc activity measured using the Dual-Glo kit (Promega) according to the manufacturer's instructions.

Experiments were conducted in duplicate. To obtain NT50s, FFluc/Rluc ratios were analyzed using the Sigmoidal, 4PL, X is log(concentration) function in GraphPad Prism.

Whole blood bulk RNA-Seq

Whole blood RNA was extracted from PAXgene Blood RNA tubes (BD Biosciences) of 188 COVID-19 patients, at up to 2 time points, and 42 healthy volunteers. RNA-Sequencing libraries were generated using the SMARTer® Stranded Total RNA-Seq v2 - Pico Input Mammalian kit (Takara) using 10ng RNA as input following the manufacturer's protocol. Libraries were pooled together (n = 96) and sequenced using 75bp paired-end chemistry across 4 lanes of a Hiseq4000 instrument (Illumina) to achieve 10 million reads per sample. Read quality was assessed using FastQC v.0.11.8 (Babraham Bioinformatics, UK), and SMARTer adaptors trimmed and residual

rRNA reads depleted *in silico* using Trim_galore v.0.6.4 (Babraham Bioinformatics, UK) and BBSplit (BBMap v.38.67(BBMap - Bushnell B. - https://sourceforge.net/projects/bbmap/)), respectively. Alignment was performed using HISAT2 v.2.1.0 (Kim et al., 2019) against the GRCh38 genome, achieving a greater than 95% alignment rate. Count matrices were generated using featureCounts (Rsubreads package - (Liao et al., 2020) and stored as a DGEList object (EdgeR package (Robinson et al., 2010) for further analysis.

All downstream data handling was performed in R (R Core Team, 2015). Counts were filtered using filterByExpr (EdgeR) with a gene count threshold of 10 CPM and the minimum number of samples set at the size of the smallest disease group. Library counts were normalized using calcNormFactors (EdgeR) using the method 'weighted trimmed mean of M-values'. The function 'voom' (Law et al., 2014) was applied to the data to estimate the mean-variance relationship, allowing adjustment for heteroscedasticity.

Single cell RNA-seq

CITE-seq data were generated from frozen PBMCs as described by Stephenson et al. (Stephenson et al., 2021). Briefly, after thawing, pools of 4 samples were generated by combined 500,000 viable cells per individual (total of 2 million cells per pool). TotalSeq-C antibody cocktail (BioLegend 99813) was used to perform cell surface marker staining on 500,000 cells per pool. 50,000 live cells (up to a maximum of 60,000 total cells) for each pool were processed using Single Cell V(D)J 5¢ version 1.1 (1000020) together with Single Cell 5¢ Feature Barcode library kit (1000080), Single Cell V(D)J Enrichment Kit, Human B Cells (1000016) and Single Cell V(D)J Enrichment Kit, Human T Cells (1000005) (10xGenomics) according to the manufacturer's protocols. Samples were sequenced on NovaSeq 6000 (Illumina) using S1 flowcells. Droplet libraries were processed using Cellranger v4.0. Reads were aligned to the GRCh38 human genome concatenated to the SARS-Cov-2 genome (NCBI SARS-CoV-2 isolate Wuhan-Hu-1) using STAR (Dobin et al., 2013) and unique molecular identifiers (UMIs) deduplicated. CITE-seq UMIs were counted for GEX and ADT libraries simultaneously to generate feature X droplet UMI count matrices.

QUANTIFICATION AND STATISTICAL ANALYSIS

All statistical analyses were conducted using custom scripts in R (R Core Team, 2015). Absolute cell counts (cells/uL) were offset by +1 to allow subsequent log2 transformation of zero counts. Where shown, time measures represent time from symptom onset (for severity groups B, C, D and E) or first positive COVID-19 swab (group A). Unless otherwise specified, longitudinally collected data was grouped by bins of 7 or 12 days. Pairwise statistical comparison of absolute cell counts, CRP or serum measures between individuals in a given severity group at a given time bin and HCs, or between severity groups, was conducted by Wilcoxon test unless otherwise specified. For analyses involving repeated-measures, false discovery rate corrected (Benjamini & Hochberg) p value were reported. For individuals sampled more than once within a given time bin, data from the earliest blood collection was used.

Cell subset deconvolution of the whole blood RNA-Seq dataset was performed using pathway-level information extractor (PLIER) (http://gobie.csb.pitt.edu/PLIER). Latent factors were generated by leveraging off pre-existing knowledge of cell specific pathways. To better understand the relationship between gene expression and clinical severity, weighted gene co-expression network analysis was carried out using the WGCNA package (Langfelder and Horvath, 2008) in R. Briefly, a signed adjacency matrix was generated and a soft thresholding power was chosen to impose approximate scale-free topology. Modules were identified from the resulting topological overlap matrix with a specified minimum module size of n = 30. Modules were summarized using singular value decomposition, and the resulting module eigengene correlated with clinical traits. Significance of the correlation between a given clinical trait and a modular eigengene was assessed using linear regression with Bonferroni adjustment to correct for multiple testing. Modules were annotated using Enrichr (Chen et al., 2013). Longitudinal mixed modeling of gene module changes over time (y_{ij}) was conducted using the *nlme* package in R (Pinheiro et al., 2021), including time (t_{ij}) with a quadratic trend and disease severity category or unsupervised cluster ids (X_j) as fixed effects, and sampled individuals as random effects (u_j):

$$y_{ij} = \beta_{0j} + \beta_{1j} t_{ij} + \beta_{2j} t_{ij}^2 + \varepsilon_{ij}, \quad \varepsilon_{ij} \sim N\left(0, \sigma^2\right),$$

$$\beta_{0j} = \gamma_{00} + \gamma_{01} X_j + u_j, \quad \beta_{1j} = \gamma_{10} + \gamma_{11} X_j, \quad \beta_{2j} = \gamma_{20} + \gamma_{21} X_j, \quad u_j \sim N\left(0, \tau^2\right),$$

i.e., using the *lme* formula:

$$\text{module\_eigenvalue} \sim (\text{time} + \text{I}(\text{time}\hat{2})) * \text{category}, \text{random} = \sim 1|\text{subject}$$

Gene set enrichment analysis (GSEA) (Subramanian et al., 2005) was used to identify biological pathways enriched in COVID-19 severity groups relative to healthy controls. Briefly, a list of ranked genes, determined by Signal-To-Noise ratio, was generated. An enrichment score was calculated, determined by how often genes from the geneset of interest appeared at the top or the bottom of the pre-ranked set of genes with the enrichment score representing the maximum deviation from zero. To assess statistical significance, an empirical phenotype- based permutation test was run, where a collection of enrichment scores was generated from the random assignment of phenotype to samples and used to generate a null distribution. To account for multiple testing, an FDR rate q < 0.20 was deemed significant. A leading edge analysis was performed to determine the genes contributing the most to the enrichment of a given pathway and was subsequently illustrated in a heatmap. Hallmark gene sets from the Molecular Signatures Database (https://www.broadinstitute.org/gsea/msigdb) were used in analysis.

Principal component analysis (PCA) of centered and scaled absolute counts for 24 major cell types was conducted using the *pca()* function from the package *mixOmics* (Rohart et al., 2017). Unsupervised clustering of log2 transformed absolute cell counts, normalized to the median of the corresponding control population, was conducted using the *heatmap.2()* function from the package *gplots* (Warnes et al., 2020), with a Euclidean distance function applied to both rows and columns of the data matrix and hierarchical clustering computed using the *ward.D* method. Partial least-squares discriminant analysis (PLS-DA) was conducted using the *plsda()* function from the package *mixOmics* (Rohart et al., 2017), a supervised method of sample discrimination whereby sample clustering is informed by group membership (here patient clusters 1 and 2). The classification performance of the PLS-DA model was determined using the *perf()* function via 10 iterations of 5-fold cross-validation, with two components deemed sufficient to minimize the balanced error rate of prediction. Variable selection on components 1 and 2 was conducted using the *tune()* function, with 13 cell types selected as those most strongly contributing to discrimination of patient clusters. An AUROC curve showing the performance of a predictive model based on these 13 cell types was generated using the *auroc()* function. To assess whether clinical severity was reflected on a transcriptional level in an unsupervised fashion, K-means clustering was utilized on normalized whole blood RNASeq gene expression counts. Heatmaps were created using the ComplexHeatmap package (Gu et al., 2016), with data scaled and centered prior to visualization.

Cellular recovery rates over 60 days were calculated for each cell type in patients from groups C, D and E, split into those with persistently elevated (> 10mg/L) or resolving CRP (falling below 10mg/L by final bleed), over 60 and 40 days respectively. Using a 12 day sliding window with single day increments, the 'window of recovery' for each given cell population was defined as the window in which absolute cell counts for COVID-19 samples no longer differed from controls when assessed by Wilcoxon test, and remained as such for the subsequent 7 windows, and 80% of all windows remaining. Recovery rate was taken as the log2 normalized ratio of test and control absolute counts for patient samples collected within the first time window (0-12 days), subtracted from the equivalent value calculated within the window of recovery, divided by the upper day boundary of the recovery window.

The relationships between immunophenotyping and transcriptional data in the form of gene expression modules were assessed using Pearson's correlation (Hmisc package) and visualized with corrplot.

Immunity, Volume *54*

Supplemental information

Longitudinal analysis reveals that delayed bystander CD8+ T cell activation and early immune pathology distinguish severe COVID-19 from mild disease

Laura Bergamaschi, Federica Mescia, Lorinda Turner, Aimee L. Hanson, Prasanti Kotagiri, Benjamin J. Dunmore, Hélène Ruffieux, Aloka De Sa, Oisín Huhn, Michael D. Morgan, Pehuén Pereyra Gerber, Mark R. Wills, Stephen Baker, Fernando J. Calero-Nieto, Rainer Doffinger, Gordon Dougan, Anne Elmer, Ian G. Goodfellow, Ravindra K. Gupta, Myra Hosmillo, Kelvin Hunter, Nathalie Kingston, Paul J. Lehner, Nicholas J. Matheson, Jeremy K. Nicholson, Anna M. Petrunkina, Sylvia Richardson, Caroline Saunders, James E.D. Thaventhiran, Erik J.M. Toonen, Michael P. Weekes, Cambridge Institute of Therapeutic Immunology and Infectious Disease-National Institute of Health Research (CITIID-NIHR) COVID BioResource Collaboration, Berthold Göttgens, Mark Toshner, Christoph Hess, John R. Bradley, Paul A. Lyons, and Kenneth G.C. Smith

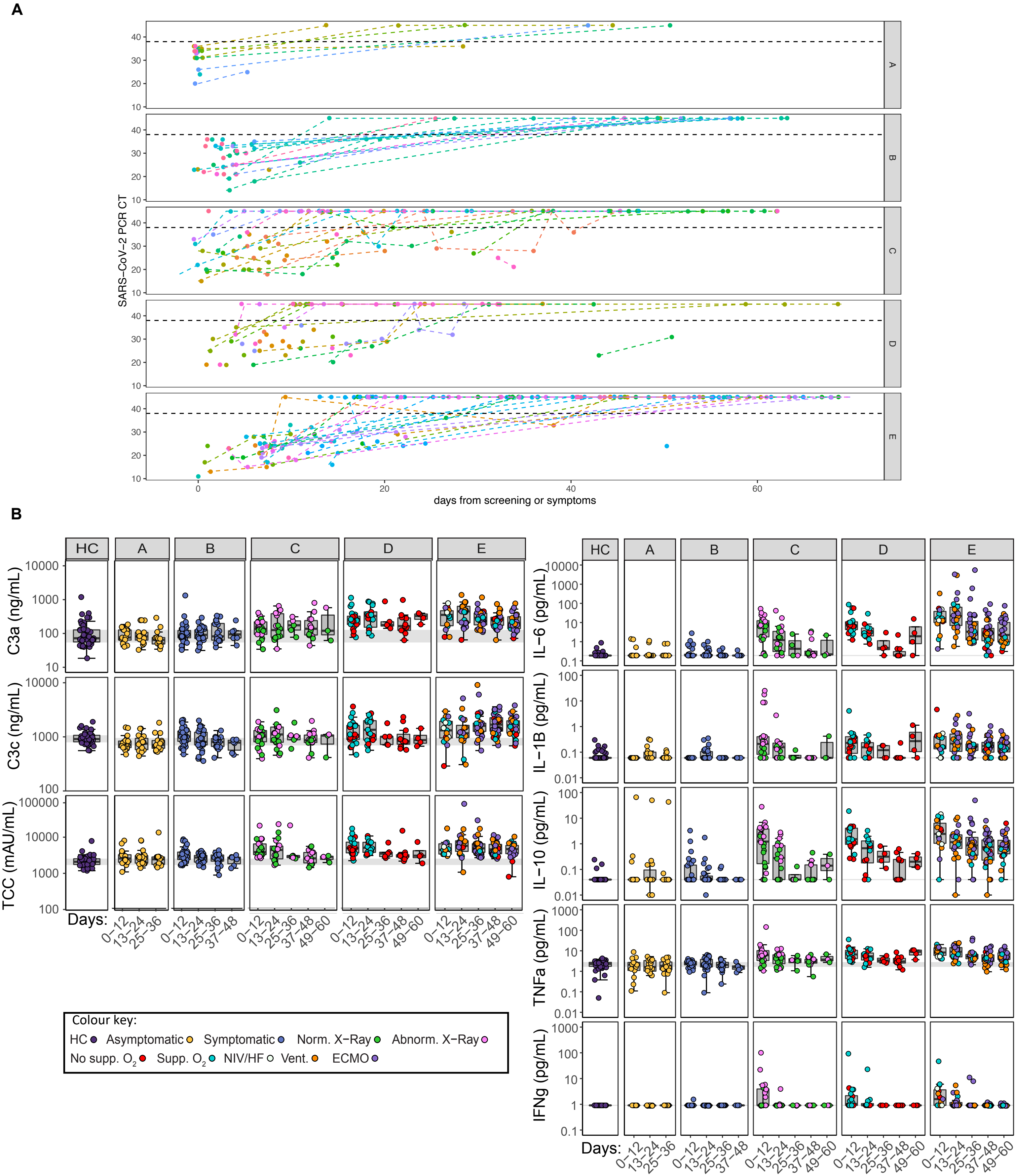
A
SARS−CoV−2 PCR CT
days from screening or symptoms
B
HC A B C D E
C3a (ng/mL)
C3c (ng/mL)
TCC (mAU/mL)
IL−6 (pg/mL)
IL−1B (pg/mL)
IL−10 (pg/mL)
TNFa (pg/mL)
IFNg (pg/mL)
Days: 0−12 13−24 25−36 37−48 49−60
Colour key:
HC Asymptomatic Symptomatic Norm. X−Ray Abnorm. X−Ray
No supp. O2 Supp. O2 NIV/HF Vent. ECMO

Figure S1: Inflammation and viral load changes over time in COVID-19 patients, related to Figure 1

A) SARS-CoV-2 PCR CT values over time across patient severity groups. CT values ≤ 38 (dashed horizontal line) were reported as a positive result. A discretional CT number of 45 was assigned to samples with no detectable SARS-CoV-2 RNA. Each colour corresponds to a different subject. Repeat measures for a single participant are linked by dashed line. **B)** Boxplots showing complement components and cytokine concentrations for samples collected within 12-day time bins. Grey band indicates the interquartile range of the corresponding measure in HCs. Points are coloured based on asymptomatic or symptomatic classification for categories A and B respectively, normal or abnormal chest radiology (group C), and type of respiratory support at sampling (group D and E), as colour key provided. NIV/HF, non-invasive ventilation/high-flow oxygen; vent, mechanical ventilation; ECMO, extracorporeal membrane oxygenation.

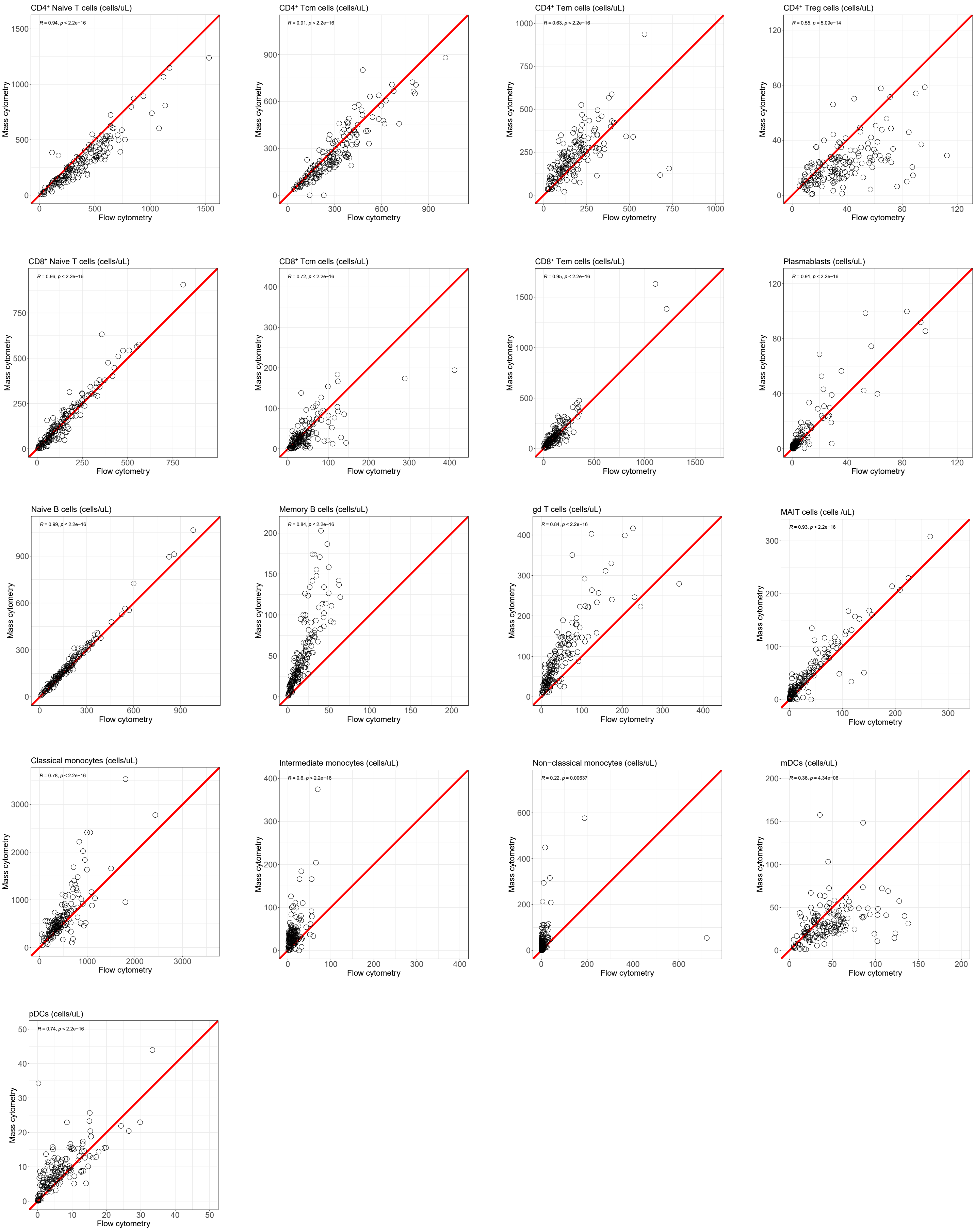

CD4⁺ Naive T cells (cells/uL)
R = 0.94, p < 2.2e−16
CD4⁺ Tcm cells (cells/uL)
R = 0.91, p < 2.2e−16
CD4⁺ Tem cells (cells/uL)
R = 0.63, p < 2.2e−16
CD4⁺ Treg cells (cells/uL)
R = 0.55, p = 5.09e−14
CD8⁺ Naive T cells (cells/uL)
R = 0.96, p < 2.2e−16
CD8⁺ Tcm cells (cells/uL)
R = 0.72, p < 2.2e−16
CD8⁺ Tem cells (cells/uL)
R = 0.95, p < 2.2e−16
Plasmablasts (cells/uL)
R = 0.91, p < 2.2e−16
Naive B cells (cells/uL)
R = 0.99, p < 2.2e−16
Memory B cells (cells/uL)
R = 0.84, p < 2.2e−16
gd T cells (cells/uL)
R = 0.84, p < 2.2e−16
MAIT cells (cells /uL)
R = 0.93, p < 2.2e−16
Classical monocytes (cells/uL)
R = 0.78, p < 2.2e−16
Intermediate monocytes (cells/uL)
R = 0.6, p < 2.2e−16
Non−classical monocytes (cells/uL)
R = 0.22, p = 0.00637
mDCs (cells/uL)
R = 0.36, p = 4.34e−06
pDCs (cells/uL)
R = 0.74, p < 2.2e−16
Mass cytometry
Flow cytometry

Figure S2: Comparison of absolute cell counts derived by flow and mass cytometry, related to STAR methods. Scatter plots showing the correlation between cell populations quantified by both flow and mass cytometry (cells/uL). Pearson correlation R value and p-values of correlation test are reported for each comparison.

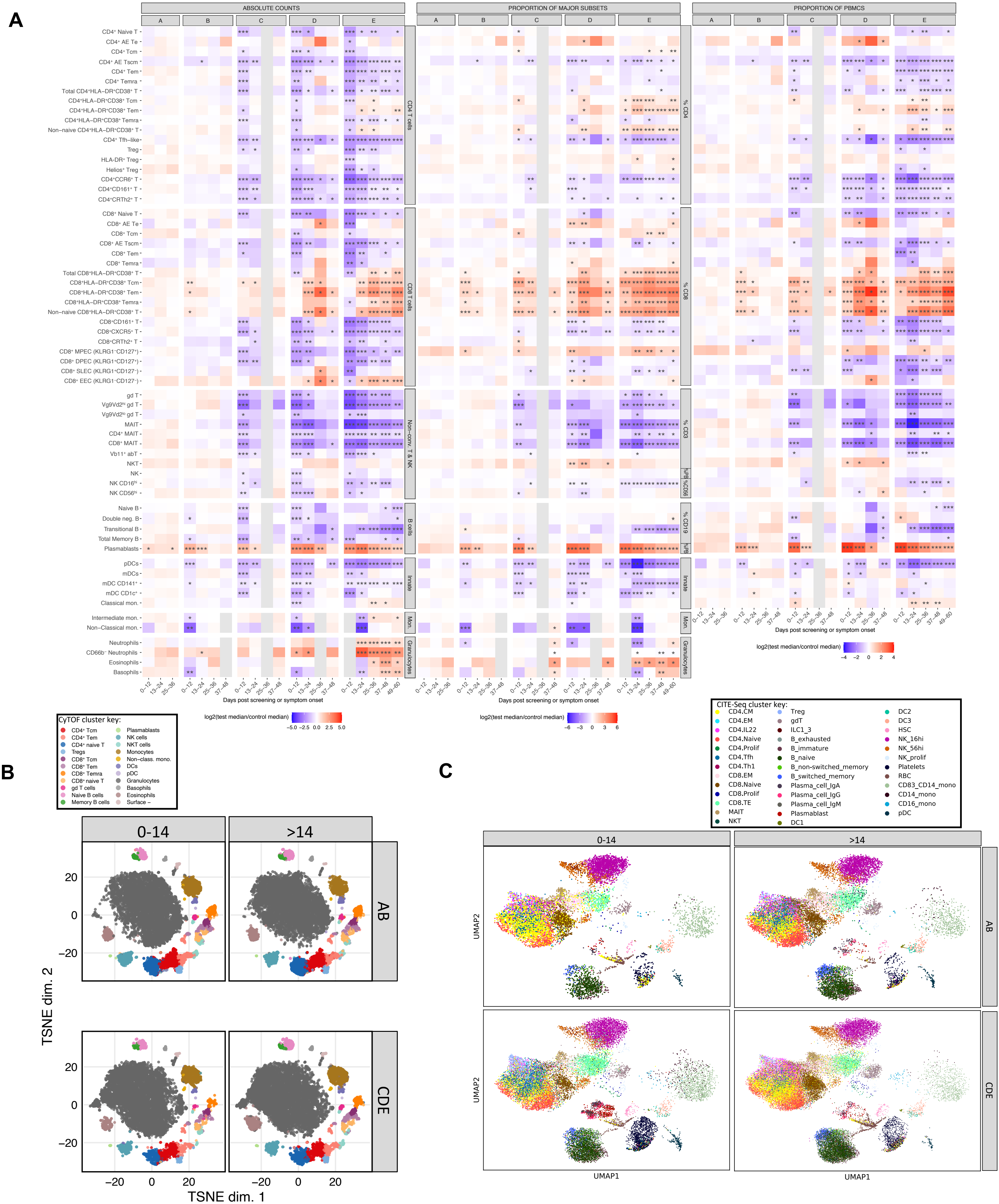

A
ABSOLUTE COUNTS
PROPORTION OF MAJOR SUBSETS
PROPORTION OF PBMCS
A
B
C
D
E
CD4⁺ Naive T
CD4⁺ AE Te
CD4⁺ Tcm
CD4⁺ AE Tscm
CD4⁺ Tem
CD4⁺ Temra
Total CD4⁺HLA−DR⁺CD38⁺ T
CD4⁺HLA−DR⁺CD38⁺ Tcm
CD4⁺HLA−DR⁺CD38⁺ Tem
CD4⁺HLA−DR⁺CD38⁺ Temra
Non−naive CD4⁺HLA−DR⁺CD38⁺ T
CD4⁺ Tfh−like
Treg
HLA-DR⁺ Treg
Helios⁺ Treg
CD4⁺CCR6⁺ T
CD4⁺CD161⁺ T
CD4⁺CRTh2⁺ T
CD8⁺ Naive T
CD8⁺ AE Te
CD8⁺ Tcm
CD8⁺ AE Tscm
CD8⁺ Tem
CD8⁺ Temra
Total CD8⁺HLA−DR⁺CD38⁺ T
CD8⁺HLA−DR⁺CD38⁺ Tcm
CD8⁺HLA−DR⁺CD38⁺ Tem
CD8⁺HLA−DR⁺CD38⁺ Temra
Non−naive CD8⁺HLA−DR⁺CD38⁺ T
CD8⁺CD161⁺ T
CD8⁺CXCR5⁺ T
CD8⁺CRTh2⁺ T
CD8⁺ MPEC (KLRG1⁻CD127⁺)
CD8⁺ DPEC (KLRG1⁺CD127⁺)
CD8⁺ SLEC (KLRG1⁺CD127⁻)
CD8⁺ EEC (KLRG1⁻CD127⁻)
gd T
Vg9Vd2ʰⁱ gd T
Vg9Vd2ˡᵒ gd T
MAIT
CD4⁺ MAIT
CD8⁺ MAIT
Vb11⁺ abT
NKT
NK
NK CD16ʰⁱ
NK CD56ʰⁱ
Naive B
Double neg. B
Transitional B
Total Memory B
Plasmablasts
pDCs
mDCs
mDC CD141⁺
mDC CD1c⁺
Classical mon.
Intermediate mon.
Non−Classical mon.
Neutrophils
CD66b⁻ Neutrophils
Eosinophils
Basophils
CD4 T cells
CD8 T cells
Non−conv. T & NK
B cells
Innate
Mon.
Granulocytes
% CD4
% CD8
% CD3
%PB
%CD56
% CD19
0−12
13−24
25−36
37−48
49−60
Days post screening or symptom onset
log2(test median/control median)
−5.0 −2.5 0.0 2.5 5.0
−6 −3 0 3 6
−4 −2 0 2 4
B
CyTOF cluster key:
CD4⁺ Tcm
CD4⁺ Tem
CD4⁺ naive T
Tregs
CD8⁺ Tcm
CD8⁺ Tem
CD8⁺ Temra
CD8⁺ naive T
gd T cells
Naive B cells
Memory B cells
Plasmablasts
NK cells
NKT cells
Monocytes
Non−class. mono.
DCs
pDC
Granulocytes
Basophils
Eosinophils
Surface −
0-14
>14
AB
CDE
TSNE dim. 2
TSNE dim. 1
20
0
−20
C
CITE-Seq cluster key:
CD4.CM
CD4.EM
CD4.IL22
CD4.Naive
CD4.Prolif
CD4.Tfh
CD4.Th1
CD8.EM
CD8.Naive
CD8.Prolif
CD8.TE
MAIT
NKT
Treg
gdT
ILC1_3
B_exhausted
B_immature
B_naive
B_non-switched_memory
B_switched_memory
Plasma_cell_IgA
Plasma_cell_IgG
Plasma_cell_IgM
Plasmablast
DC1
DC2
DC3
HSC
NK_16hi
NK_56hi
NK_prolif
Platelets
RBC
CD83_CD14_mono
CD14_mono
CD16_mono
pDC
UMAP2
UMAP1

Figure S3: Cellular changes observed over time in COVID-19 patients including absolute cell counts and proportion, related to Figure 2

A) Heatmap showing the log2 fold change in median absolute cell count between COVID-19 patients and healthy controls, within severity categories and across 12-day time bins post screening (group A) or symptom onset (groups B-E). Missing data are shown in grey. Wilcoxon test FDR adjusted p-values: *<0.05, **<0.005, *** <0.0005. **B)** tSNE **and C)** UMAP plots comparing groups A and B to groups C, D and E within 14 or later (>14) days post screening (group A) or symptom onset (groups B-E) for **B)** CyTOF and **C)** CITE-Seq dataset. Cell clusters are coloured as key provide.

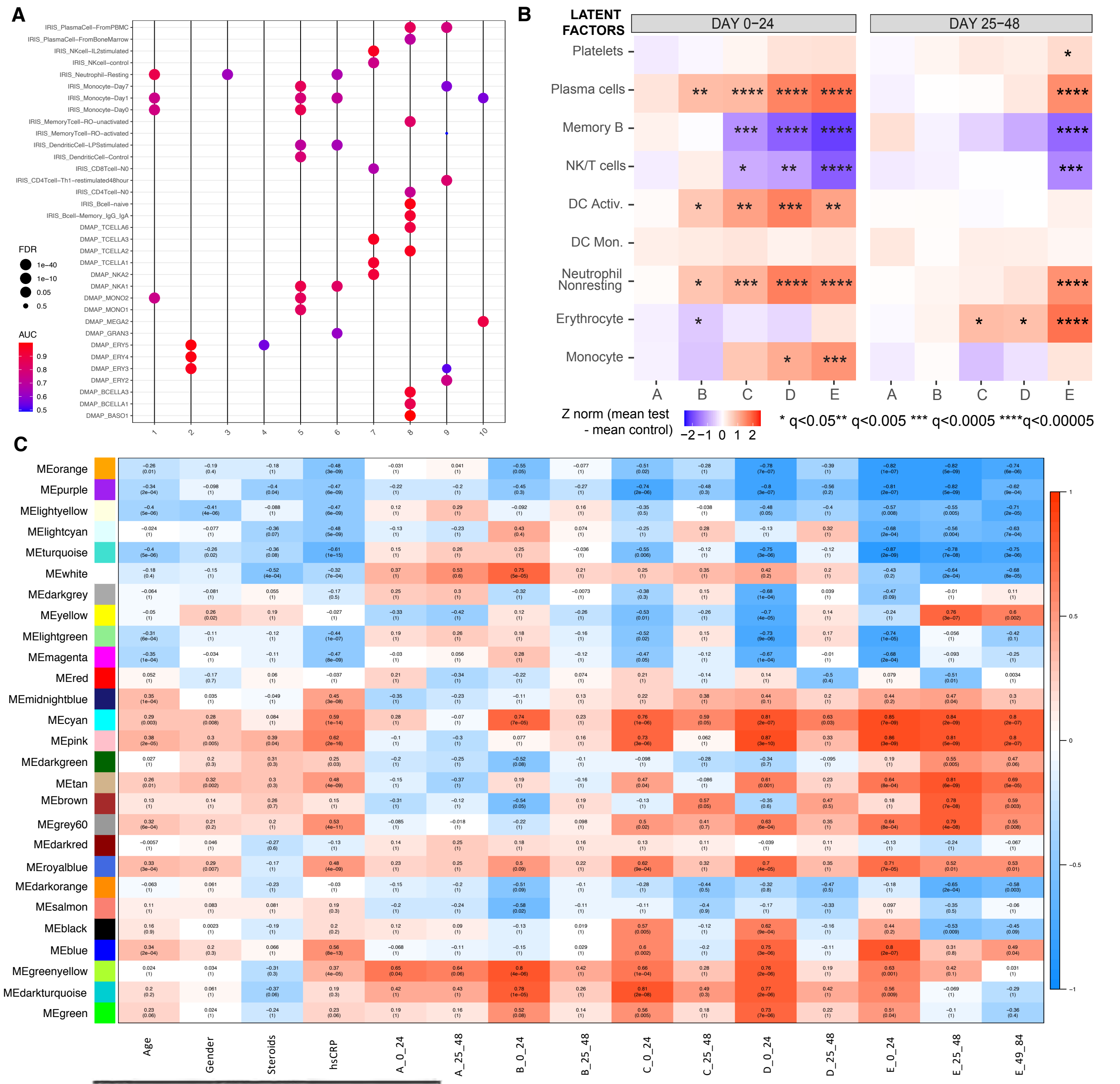

D

Module	Enrichr annotation
**Positively correlated in all severity groups during active disease**	
MEcyan	Histones
MEblue	TNFa/IL-6
MEpink	Complement/Coagulation/Neut degranulation
MEmidnightblue	Platelet activation
MEgrey60	Ferroptosis
MEroyalblue	Glycolysis
MEtan	No annotation
**Positively correlated at all time points except late severe**	
MEgreenyellow	Immunoglobulins
MEgreen	Interferon Stimulated Genes
MEwhite	No annotation
MEdarkturquise	No annotation
**Positively correlated in early mod/severe groups**	
MEblack	No annotation
**Positively correlated in late mod/severe groups**	
MEbrown	Heme metabolism
**Positively correlated in late severe group**	
MEyellow	Oxidative Phosphorylation
MEdarkgreen	No annotation
**Negatively correlated with disease especially in early mod/severe disease**	
MEdarkgrey	GPCR
MElightgreen	Ribosomal proteins
MEmagenta	MYC targets
MEorange	No annotation
MElightgreen	No annotation
**Negatively correlated with disease especially in late severe group**	
MEturquoise	Gene transcription
MEpurple	Splicesome
MElightyellow	BCR signalling
MElightcyan	IL-2/NK
MEred	No annotation
MEdarkorange	No annotation
**Negatively correlated with disease especially in early mild disease**	
MEsalmon	No annotation
**No correlate with disease**	
MEdarkred	No annotation

E

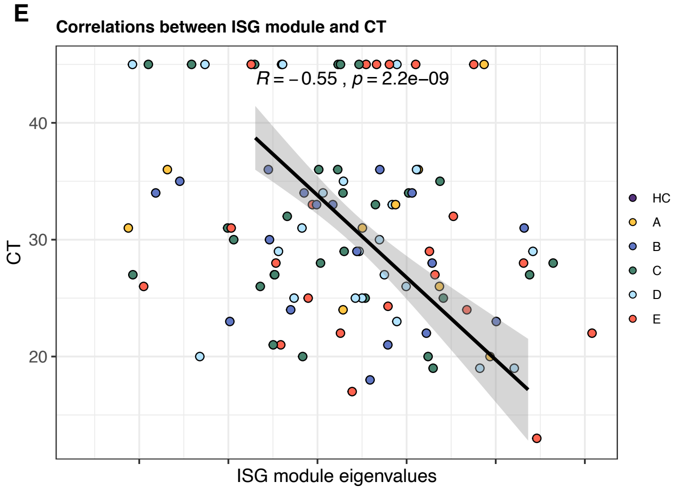

Figure S4: Cell subset deconvolution of whole blood transcriptome and transcriptional signatures observed over time in COVID-19, related to Figure 3. A) Annotation of Latent Factors used to perform the cell subset deconvolution shown in **B). B)** Cell subset deconvolution performed using PLIER, leveraging off prior knowledge of cell specific pathways. COVID-19 cases split by severity categories and 24-day time bins. Latent factor expression compared with HCs, FDR adjusted p-value: *<0.05, **<0.005, ***<0.0005. **C)** Heatmap illustrating the correlation among whole blood co-expression gene modules derived from WGCNA (coloured blocks, y axis) and age, gender, steroid treatment, CRP concentrations, and the comparison between HCs and COVID-19 cases split by severity in 24 days bins (x axis). Pearson correlation and FDR corrected p-values are shown for each comparison. The full list of genes in each module can be found in **Table S3**. **D)** Annotation by EnrichR of modules that correlate with disease and/or severity. **E)** Correlation between SARS-CoV-2 PCR CT values and Interferon Signature Genes (IGS) module eigenvalues (samples, n=248).

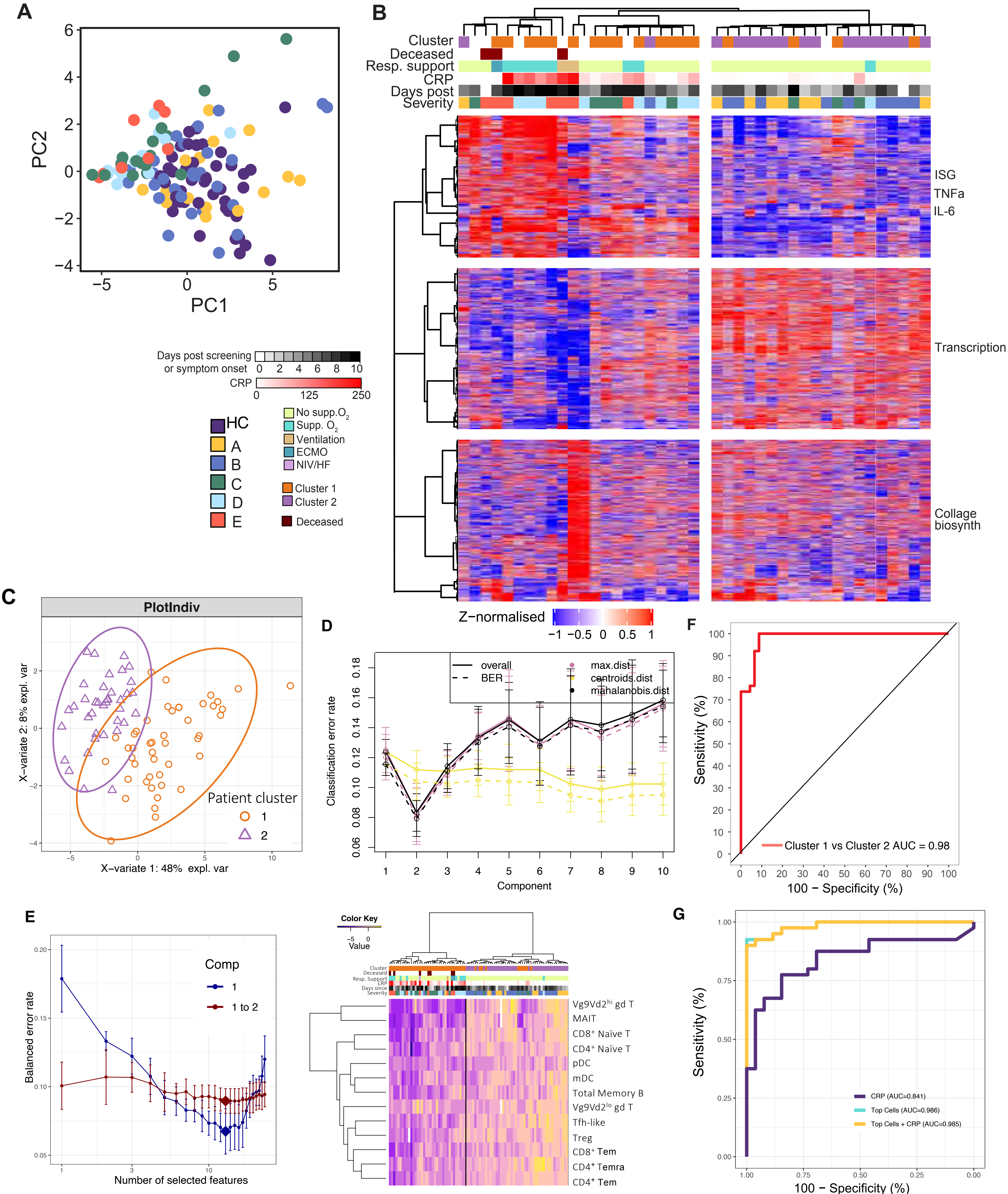

A
PC2
PC1
Days post screening or symptom onset
CRP
HC
A
B
C
D
E
No supp.O2
Supp. O2
Ventilation
ECMO
NIV/HF
Cluster 1
Cluster 2
Deceased
B
Cluster
Deceased
Resp. support
CRP
Days post
Severity
ISG
TNFa
IL-6
Transcription
Collage biosynth
Z−normalised
C
PlotIndiv
X−variate 2: 8% expl. var
X−variate 1: 48% expl. var
Patient cluster
D
overall
BER
max.dist
centroids.dist
mahalanobis.dist
Classification error rate
Component
F
Sensitivity (%)
100 − Specificity (%)
Cluster 1 vs Cluster 2 AUC = 0.98
E
Comp
1
1 to 2
Balanced error rate
Number of selected features
Color Key
Value
Vg9Vd2hi gd T
MAIT
CD8+ Naïve T
CD4+ Naïve T
pDC
mDC
Total Memory B
Vg9Vd2lo gd T
Tfh-like
Treg
CD8+ Tem
CD4+ Temra
CD4+ Tem
G
Sensitivity (%)
100 − Specificity (%)
CRP (AUC=0.841)
Top Cells (AUC=0.986)
Top Cells + CRP (AUC=0.985)

Figure S5: Multivariate analysis of immune-cell populations in early disease, related to Figure 4

A) Principal component analysis of peripheral blood absolute cell counts for 24 key cell subsets from HCs and COVID-19 cases, for samples taken ≤10 days from screening (group A) or symptom onset (groups B-E). Points are coloured according to severity category. **B)** K-means clustering of 18357 whole blood transcripts from COVID-19 samples taken ≤10 days from screening or symptom onset. Gene clusters are annotated for enriched signatures, samples are annotated according to corresponding cluster membership in **Figure 4A** where possible. **C)** Variable selection by sPLS-DA showing discrimination of patient clusters 1 (orange, n=46) and 2 (purple, n=38) derived in **Figure 4A**. **D)** Associated classification error rate of the predictive model across 10 iterations of 5-fold cross validation for components 1-10. **E)** Feature selection on components 1 and 2, determining 13 cell subsets as key contributors to cluster discrimination with minimal error. Unsupervised clustering of 13 selected cell types (normalised to the median of HCs), with original sample clusters and patient characteristics indicated. Error bars in **D)** and **E)** indicate standard deviations (SD). **F) and G)** AUROC curves showing sensitivity of cluster group prediction at varying specificity thresholds, based on **F)** absolute counts of 13 selected cell types, or **G)** CRP alone and combined with the absolute counts of 13 selected cell types.

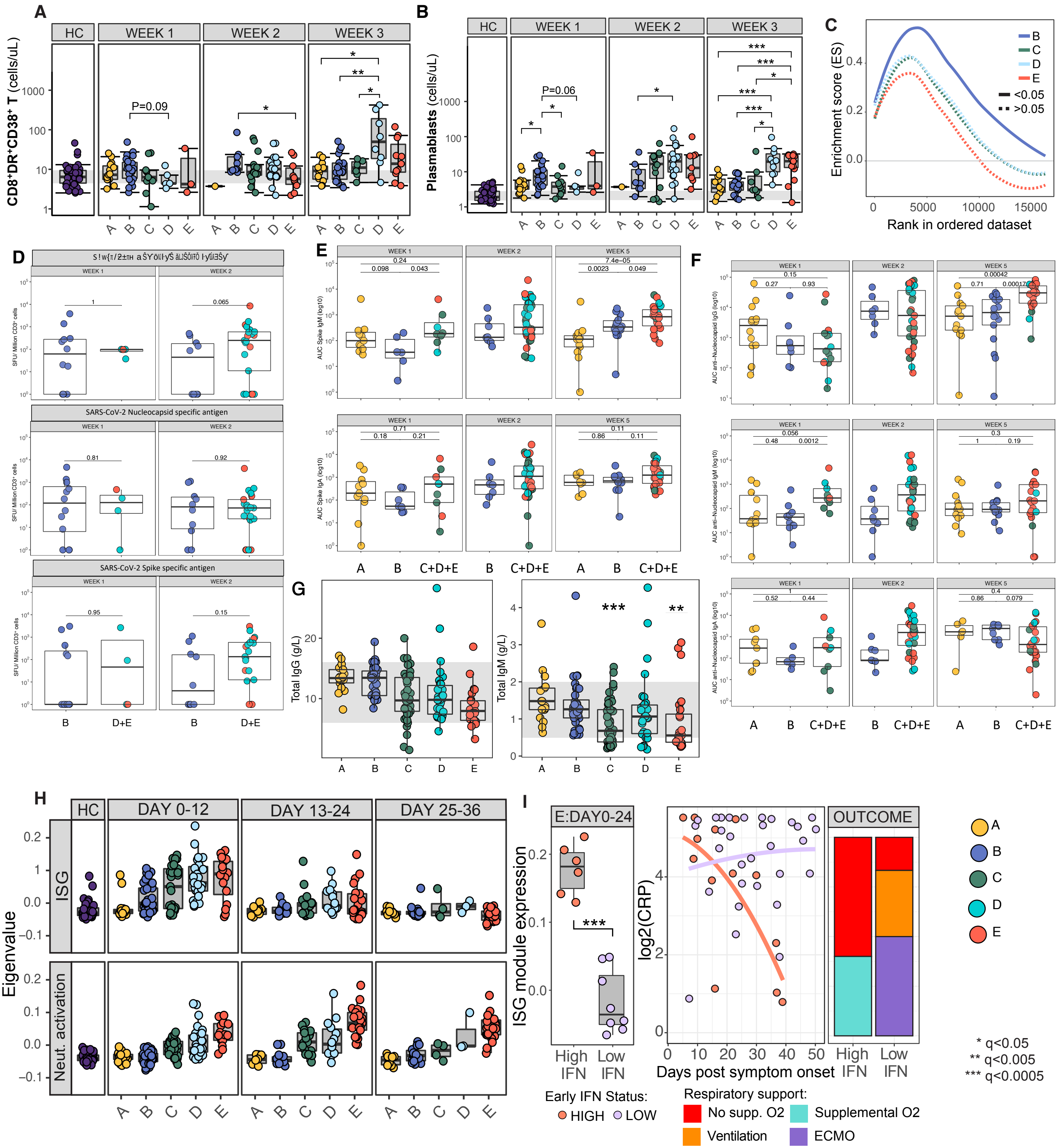
A
HC
WEEK 1
WEEK 2
WEEK 3
CD8+DR+CD38+ T (cells/uL)
P=0.09
B
Plasmablasts (cells/uL)
P=0.06
C
Enrichment score (ES)
Rank in ordered dataset
<0.05
>0.05
D
SARS-CoV-2 Nucleocapsid specific antigen
SARS-CoV-2 Spike specific antigen
SFU/ Million CD3+ cells
B
D+E
E
WEEK 5
AUC Spike IgM (log10)
AUC Spike IgA (log10)
A
C+D+E
F
AUC anti-Nucleocapsid IgG (log10)
AUC anti-Nucleocapsid IgM (log10)
AUC anti-Nucleocapsid IgA (log10)
G
Total IgG (g/L)
Total IgM (g/L)
H
DAY 0-12
DAY 13-24
DAY 25-36
ISG
Neut. activation
Eigenvalue
I
E:DAY0-24
ISG module expression
High IFN
Low IFN
log2(CRP)
Days post symptom onset
OUTCOME
Early IFN Status:
HIGH
LOW
Respiratory support:
No supp. O2
Supplemental O2
Ventilation
ECMO
* q<0.05
** q<0.005
*** q<0.0005

Figure S6: Early immune changes associated with mild disease and outcome extended data, related to Figure 5

A and B): Boxplots showing **A)** non-naive $CD8^{+}HLA\text{-}DR^{+}CD38^{+}$ T cell and **B)** plasmablast absolute cell counts (cells/uL) across severity categories at weeks 1-3 post screening (group A) or symptom onset (groups B-E). Wilcoxon test FDR adjusted p-value: *<0.05, **<0.005, ***<0.0005. Grey bar represents the interquartile range of the same cell populations in HCs. **C)** Enrichment score for $CD8^{+}$ T-cell activation signature as determined by GSEA in groups B-E for samples taken <24 days from symptom onset. **D)** Spot forming unit (SFU) numbers of $CD3^{+}$ T cells secreting IFN-γ in response to membrane, nucleocapsid and spike SARS-CoV-2 antigen stimulations, in patient samples from groups B (n=22), and D and E combined (n=25), one or two weeks post symptom onset. Kruskal-Wallis test p-values. **E)** Area under the curve for SARS-CoV-2 spike-specific IgM and IgA titres at 1, 2 and 5 weeks post screening (group A) or symptom onset (groups B-E). Groups C, D and E are combined for increased statistical power. Wilcoxon test p-values. **F)** Area under the curve for SARS-CoV-2 nucleocapsid-specific IgG, IgM and IgA titres. Groups, timepoints and p-values as in **E)**. **G)** Total IgG and IgM concentrations across severity groups, within 3 weeks post screening (group A) or symptom onset (groups B-E). Grey band corresponds to 5-95th centile ranges based on UK Caucasian population, as published in the Protein Reference Unit Handbook (9th Edition). P-values from comparisons with the reference range using Pearson's chi-square test, annotated as in **A)**. **H)** Boxplots capturing expression of interferon stimulated genes (ISG) and neutrophil activation-related transcriptomic eigenvalues across disease severity and time(samples, n=248). **I)** Stratification of group E samples taken <24 days post symptom onset into high and low expression of ISG, with persisting and resolving CRP concentrations and final respiratory status reported within 12 weeks shown by bar charts.

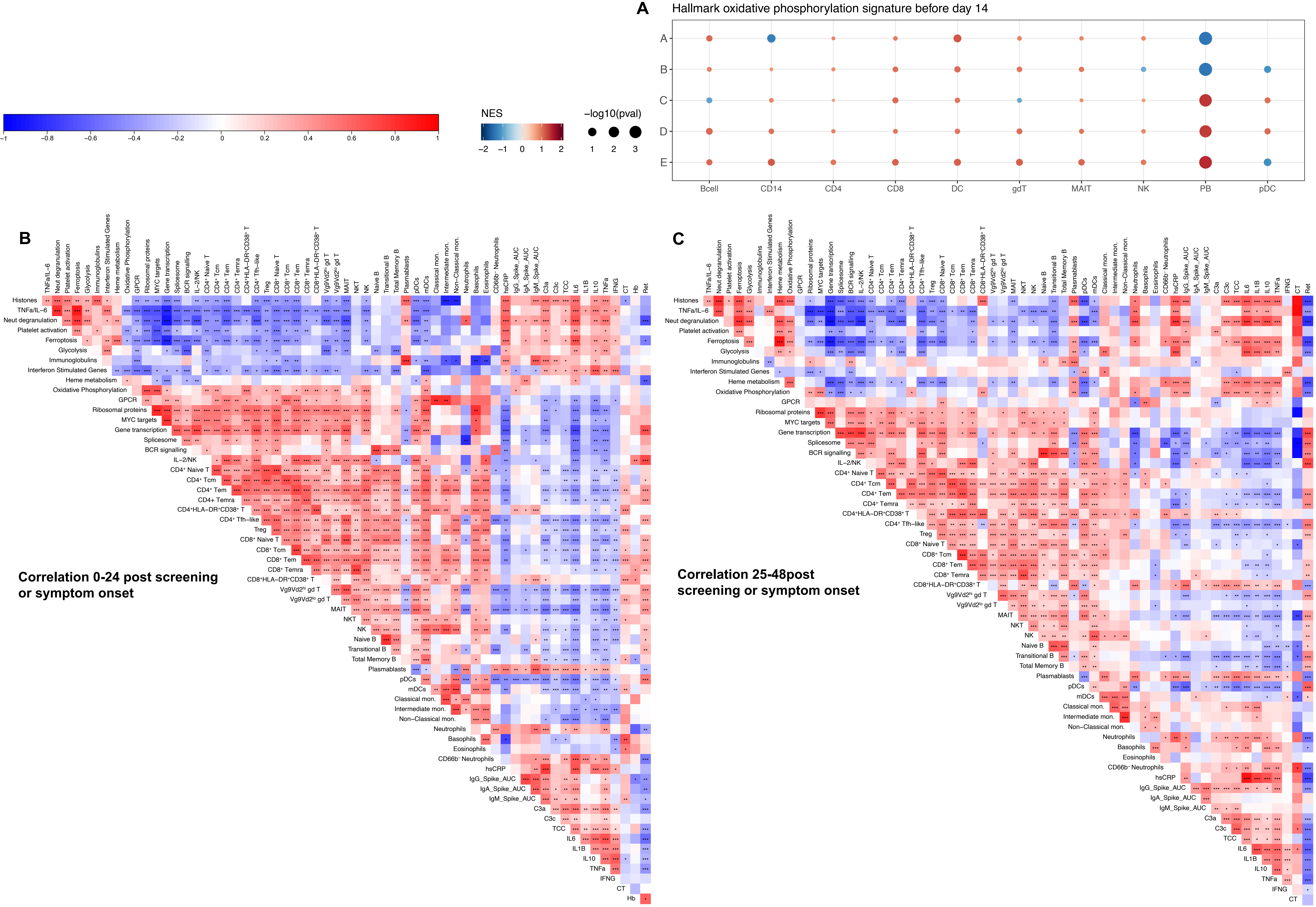

A
Hallmark oxidative phosphorylation signature before day 14
A
B
C
D
E
Bcell
CD14
CD4
CD8
DC
gdT
MAIT
NK
PB
pDC
NES
−2 −1 0 1 2
−log10(pval)
1 2 3
−1 −0.8 −0.6 −0.4 −0.2 0 0.2 0.4 0.6 0.8 1
B
C
Histones
TNFa/IL−6
Neut degranulation
Platelet activation
Ferroptosis
Glycolysis
Immunoglobulins
Interferon Stimulated Genes
Heme metabolism
Oxidative Phosphorylation
GPCR
Ribosomal proteins
MYC targets
Gene transcription
Splicesome
BCR signalling
IL−2/NK
CD4+ Naive T
CD4+ Tcm
CD4+ Tem
CD4+ Temra
CD4+HLA−DR+CD38+ T
CD4+ Tfh−like
Treg
CD8+ Naive T
CD8+ Tcm
CD8+ Tem
CD8+ Temra
CD8+HLA−DR+CD38+ T
Vg9Vd2hi gd T
Vg9Vd2lo gd T
MAIT
NKT
NK
Naive B
Transitional B
Total Memory B
Plasmablasts
pDCs
mDCs
Classical mon.
Intermediate mon.
Non−Classical mon.
Neutrophils
Basophils
Eosinophils
CD66b− Neutrophils
hsCRP
IgG_Spike_AUC
IgA_Spike_AUC
IgM_Spike_AUC
C3a
C3c
TCC
IL6
IL1B
IL10
TNFa
IFNG
CT
Hb
Ret
Correlation 0-24 post screening or symptom onset
Correlation 25-48post screening or symptom onset

Figure S7: Transcriptional changes in prolonged disease were independent of cell subset composition, related to Figures 7

A) GSEA assessing HALLMARK oxidative phosphorylation geneset in different cell type identify by CITE-Seq against HCs in COVID-19 patients collected within 14 days post screening (group A) or symptom onset (groups B-E). FDR adjusted p-value is shown by circle diameter, with colour representing normalised enrichment score. **B) and C):** Heatmap showing the correlation between gene expression eigenvalues derived from whole blood RNA-Seq, absolute cell counts and inflammatory characteristics in COVID-19 patients collected within **B)** the first 24 days, or **D)** between 25-48 days post screening (group A) or symptom onset (groups B-E). Pearson correlation p-values: *p<0.05, **p<0.01 and ***p<0.001.

Table S1: Clinical features of study participants, stratified by group A-E, related to Figure 1

	A	B	C	D	E
**n**	18	40	46	37	60
**Gender (% male)**	22.2%	22.5%	54.3%	64.9%	75.0%
**Age (years, mean (SD))**	32.9 (12.7)	36.0 (11.8)	58.0 (16.9)	64.4 (15.1)	57.0 (14.9)
**Days from COVID-19 symptoms to enrollment (days, mean (SD))**	NA	6.5 (2.9)	11.4 (6.7)	10.6 (8.1)	24.6 (14.3)
**COVID-19 chest radiology**	NA	NA	50.0%	89.2%	100%
**Non-COVID19 admissions**	NA	NA	30.2%	8.1%	6.7%
**Haemoglobin (g/L, mean (SD))**	NA	NA	124.8 (16.0)	121.6 (18.0)	95.2 (16.8)
**Serum creatinine (µmol/L, mean (SD))**	NA	NA	82.9 (40.1)	117.5 (154.7)	103.5 (129.3)
**Serum albumin (g/L, mean (SD))**	NA	NA	32.4 (7.1)	28.0 (6.3)	24.4 (7.2)
**LOS (days, median (IQR))**	NA	NA	4 (1.25-10)	10 (6-16)	44 (33.7-63.2)
**Admitted to ITU**	NA	NA	0%	13.5%	90.0%
**Deceased in hospital**	NA	NA	2.2%	0.0%	30.0%
**Hypertension**	NA	NA	47.8%	43.2%	48.3%
**CAD**	NA	NA	8.7%	24.3%	16.7%
**Other heart condition**	NA	NA	10.9%	18.9%	13.3%
**Diabetes mellitus**	NA	NA	26.1%	29.7%	43.3%
**CKD**	NA	NA	8.7%	16.2%	8.3%
**PVD**	NA	NA	6.5%	8.1%	8.3%
**CVA/TIA**	NA	NA	10.9%	2.7%	6.7%
**COPD**	NA	NA	6.5%	18.9%	5.0%
**Asthma**	NA	NA	21.7%	10.8%	10.0%
**Other lung disease**	NA	NA	10.9%	16.2%	10.0%
**Cancer**	NA	NA	4.4%	5.4%	1.7%
**Haematological cancer**	NA	NA	2.2%	5.4%	0.0%
**Corticosteroids**	NA	NA	19.6%	10.8%	10.0%
**Immunosuppressive treatment**	NA	NA	17.4%	16.2%	5.0%

SD is standard deviation, and IQR is interquartile range.
COVID-19 chest radiology: chest X-ray/ CT scan showed changes compatible with COVID-19, as opposed to normal findings or lung changes diagnostic of other conditions.
Non-COVID19 admissions: cases where COVID-19 was diagnosed during the hospital stay in patients initially admitted to hospital for reasons unrelated to COVID-19
Haemoglobin, serum albumin and serum creatinine: results from routine lab tests on the day of study enrollment, or closest result up to 2 days before. The included test results are available for at least for 75% of each severity group.
LOS: length of hospital stay (days from hospital admission to discharge, transfer or death in hospital)
Hypertension: history of hypertension, defined as blood pressure ≥140/80 on multiple occasions, or on treatment with any medication explicitly employed to reduce blood pressure
CAD: history of coronary artery disease, defined as myocardial infarction, angina, coronary artery stenting or coronary artery bypass grafting
Other heart condition: history of any other chronic cardiac disease (not CAD/hypertension), e.g. heart failure, congenital heart disease, cardiomyopathy, rheumatic heart disease
CKD: history of chronic kidney disease, defined as any of estimated glomerular filtration rate < 60 mL/min/1.73m^2, dialysis or kidney transplant
PVD: history of peripheral vascular disease, defined as intermittent claudication or past bypass for chronic arterial insufficiency, history of gangrene or acute arterial insufficiency, or thoracic/abdominal aneurysm (≥6 cm)
CVA/TIA: history of a cerebrovascular accident or transient ischemic attacks
COPD: history of chronic obstructive pulmonary disease
Other lung disease: history of other chronic pulmonary disease (non asthma/COPD), e.g. cystic fibrosis, bronchiectasis, interstitial lung disease
Cancer: current solid organ malignancy (active or in the last 5 years), except non-melanoma skin cancers
Corticosteroids: history of treatment with systemic corticosteroids in the 14 days prior to hospital admission/presentation
Immunosuppressive treatment: history of treatment with immunosuppressants (excluding corticosteroids) in the 14 days prior to hospital admission/presentation, or chemotherapy/biologic drugs in the previous 6 months

Table S2: patients excluded because of extreme confounding comorbidities, related to STAR Methods

Study ID	Patient profile
CV0266	Metastatic lung adenocarcinoma on immunotherapy and chemotherapy, presentation with new onset heart failure and pulmonary oedema, borderline positive COVID-19 PCR on nasopharyngeal swab, but no other clinical features of COVID-19
CV0258	Prolonged hospital admission for new diagnosis of acute myeloid leukemia with suspected leukemic lung infiltration and fungal chest infection, commenced on chemotherapy. COVID-19 PCR negative at hospital admission and subsequently positive.
CV0143	Mild COVID-19 symptoms, admitted for extensive necrotizing fasciitis/mediastinitis, treated with surgical debridement and complicated by massive haemorrhage. Enrolled in the study in the ITU after surgery.
CV0192	Emergency splenectomy following trauma. Fever and pneumonia in the post-op, with radiology in keeping with aspiration. Borderline positive COVID-19 PCR on nasopharyngeal swab.
CV0033	Lymphoma on palliative chemotherapy, initially admitted for interstitial pneumonia and neutropenia and treated as Pneumocystis pneumonia. COVID-19 PCR initially negative 4x and subsequently positive after 2 weeks.
CV0313	End-stage alcoholic cirrhosis with variceal bleeding and hepatic encephalopathy, admitted for transjugular intrahepatic portosystemic shunt procedure. Positive COVID-19 PCR on nasopharyngeal swab, initially with normal chest X-ray. Subsequently developed severe chest infection with bronchoalveolar lavage positive for Gram negative organisms and negative for COVID-19 PCR.

Data S1: Time course plots for hospitalized patients (groups C/D/E), related to STAR Methods

Swimmer plots for hospitalized cases in groups **A)** C, **B)** D, and **C)** E. Arrows indicate days since start of COVID-19 symptoms (or since positive SARS-CoV-2 PCR test, if asymptomatic). Black boxes indicate the duration of hospital stay whilst enrolled in the study. If patients were transferred from other hospitals (as indicated by the triangle symbol), information relative to the time before transfer to one of the study hospitals may be missing. Research blood collection timepoints are indicated by open circle, and time of death by red cross for deceased patients. Degree of respiratory support is indicated by colour gradient as per the key provided.

Legend

- no supplemental oxygen
- supplemental oxygen
- NIV/HF
- invasive ventilation
- ECMO
- ○ research bloods
- * asymptomatic
- ▽ admitted to other hospital
- → transferred to other hospital
- X deceased

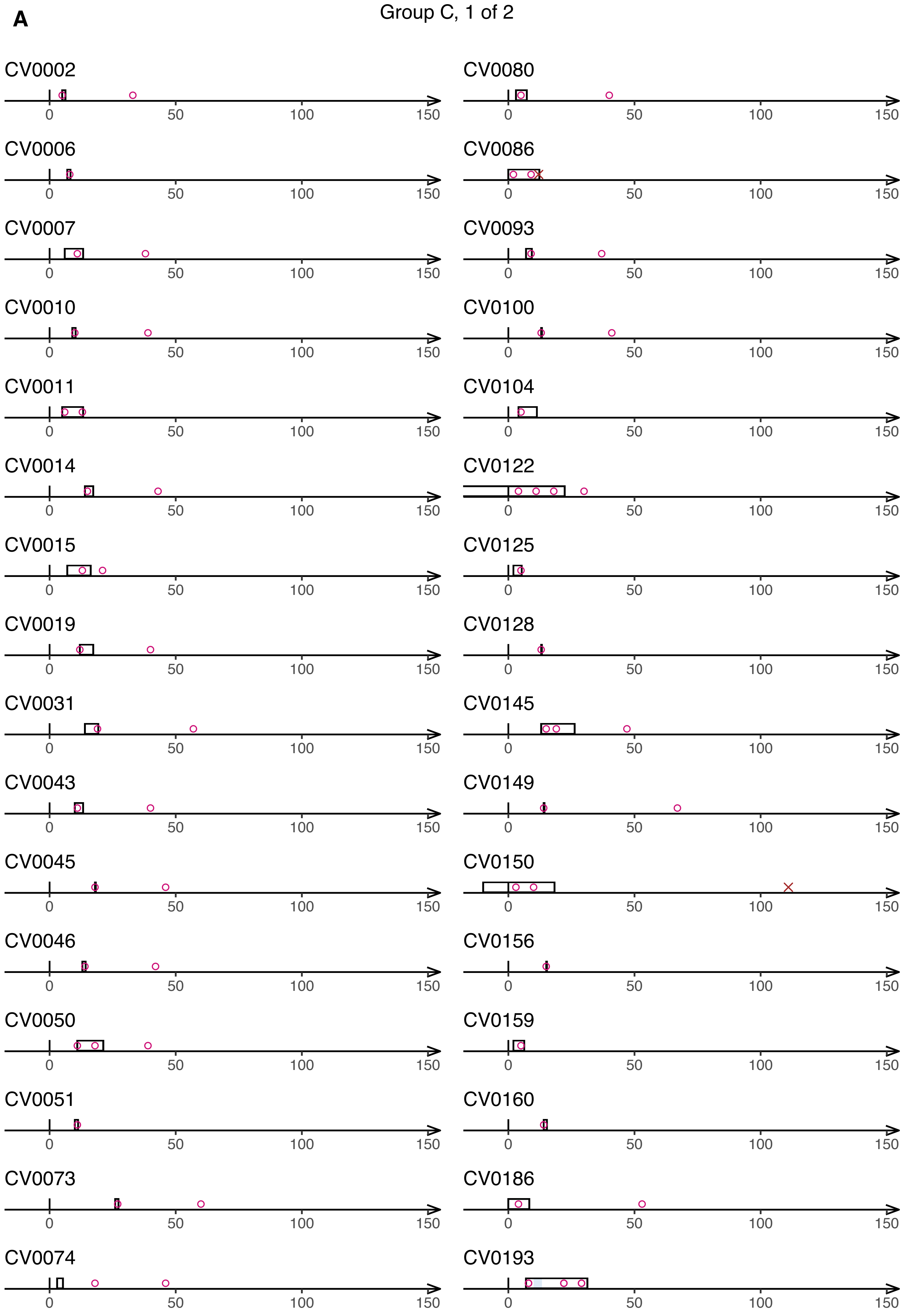
A
Group C, 1 of 2
CV0002
CV0006
CV0007
CV0010
CV0011
CV0014
CV0015
CV0019
CV0031
CV0043
CV0045
CV0046
CV0050
CV0051
CV0073
CV0074
CV0080
CV0086
CV0093
CV0100
CV0104
CV0122
CV0125
CV0128
CV0145
CV0149
CV0150
CV0156
CV0159
CV0160
CV0186
CV0193
0
50
100
150

Group C, 2 of 2

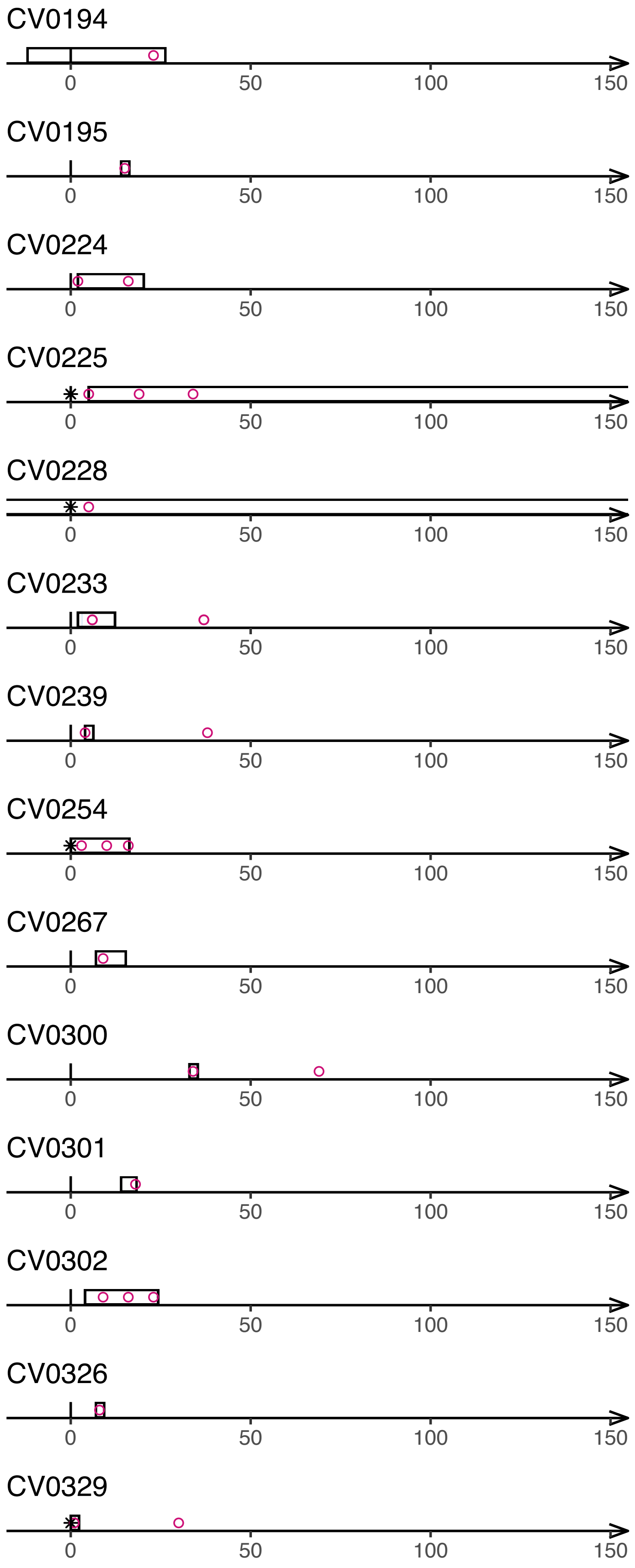
CV0194
CV0195
CV0224
CV0225
CV0228
CV0233
CV0239
CV0254
CV0267
CV0300
CV0301
CV0302
CV0326
CV0329
0
50
100
150

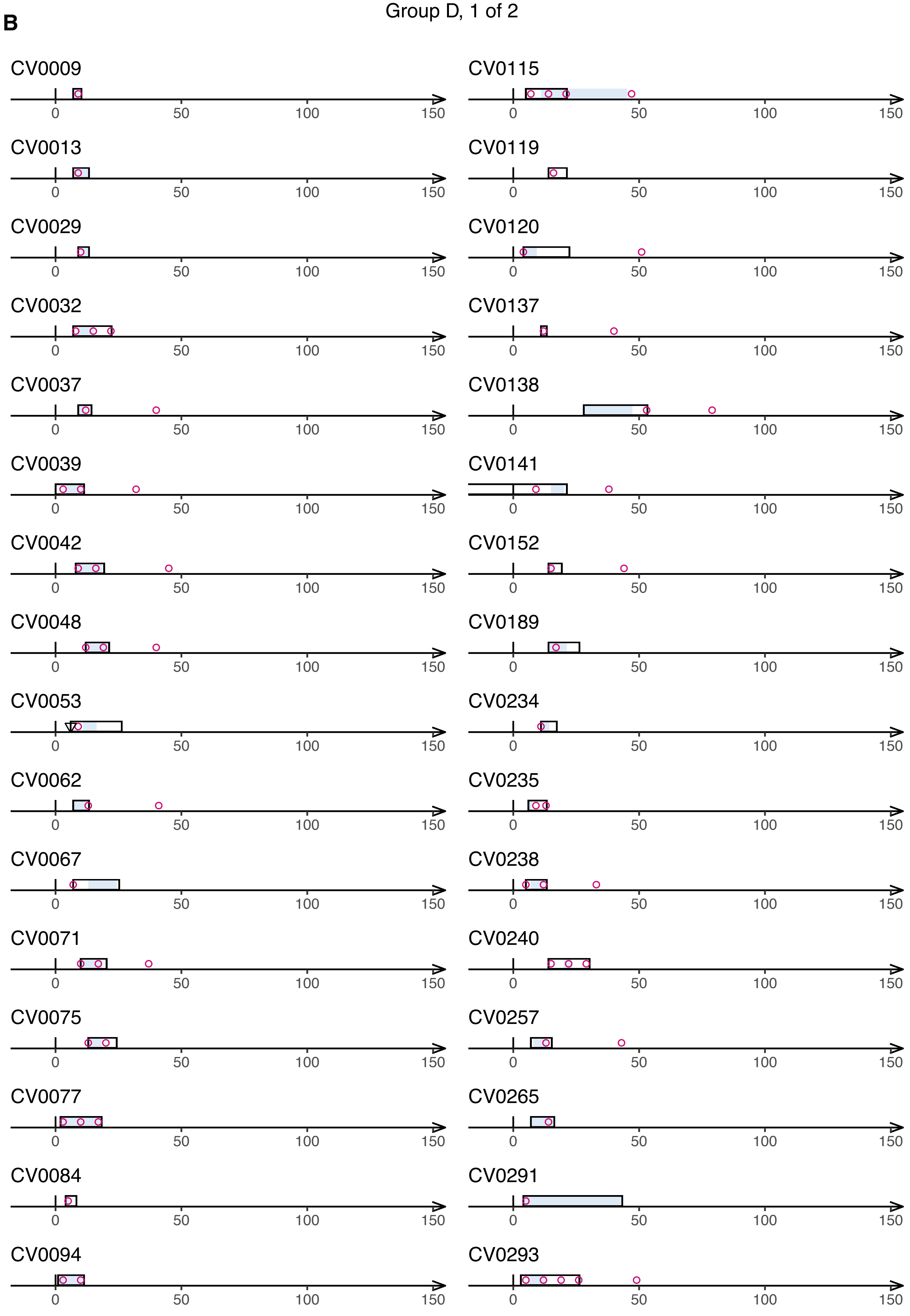
B
CV0009
CV0013
CV0029
CV0032
CV0037
CV0039
CV0042
CV0048
CV0053
CV0062
CV0067
CV0071
CV0075
CV0077
CV0084
CV0094
CV0115
CV0119
CV0120
CV0137
CV0138
CV0141
CV0152
CV0189
CV0234
CV0235
CV0238
CV0240
CV0257
CV0265
CV0291
CV0293
0
50
100
150

Group D, 2 of 2

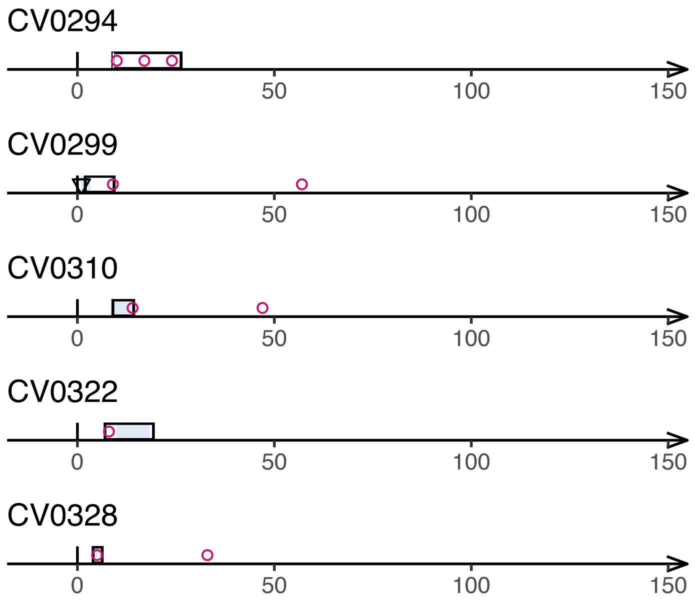

CV0294
0
50
100
150
CV0299
0
50
100
150
CV0310
0
50
100
150
CV0322
0
50
100
150
CV0328
0
50
100
150

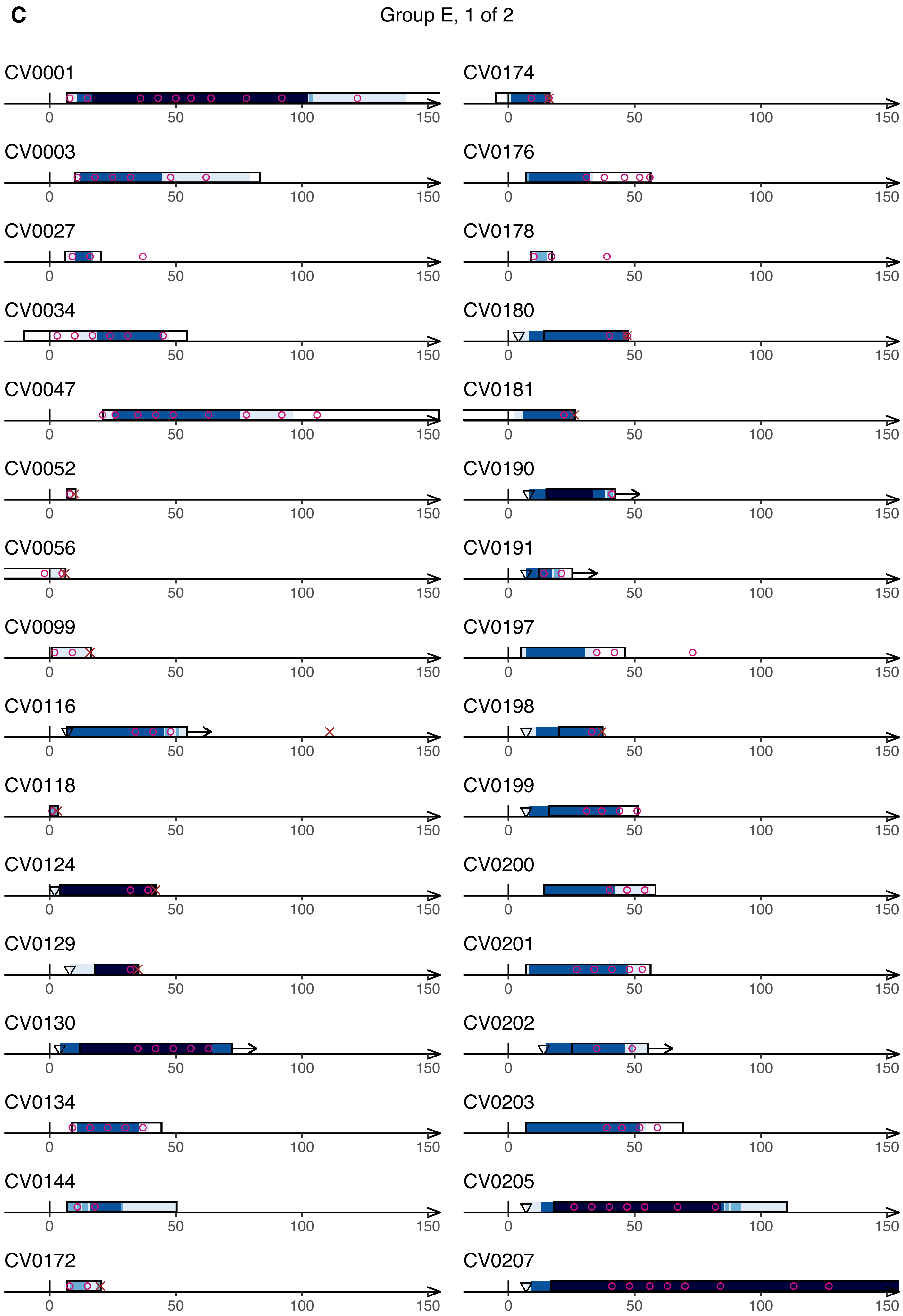
C
Group E, 1 of 2
CV0001
CV0003
CV0027
CV0034
CV0047
CV0052
CV0056
CV0099
CV0116
CV0118
CV0124
CV0129
CV0130
CV0134
CV0144
CV0172
CV0174
CV0176
CV0178
CV0180
CV0181
CV0190
CV0191
CV0197
CV0198
CV0199
CV0200
CV0201
CV0202
CV0203
CV0205
CV0207
0
50
100
150

Group E, 2 of 2

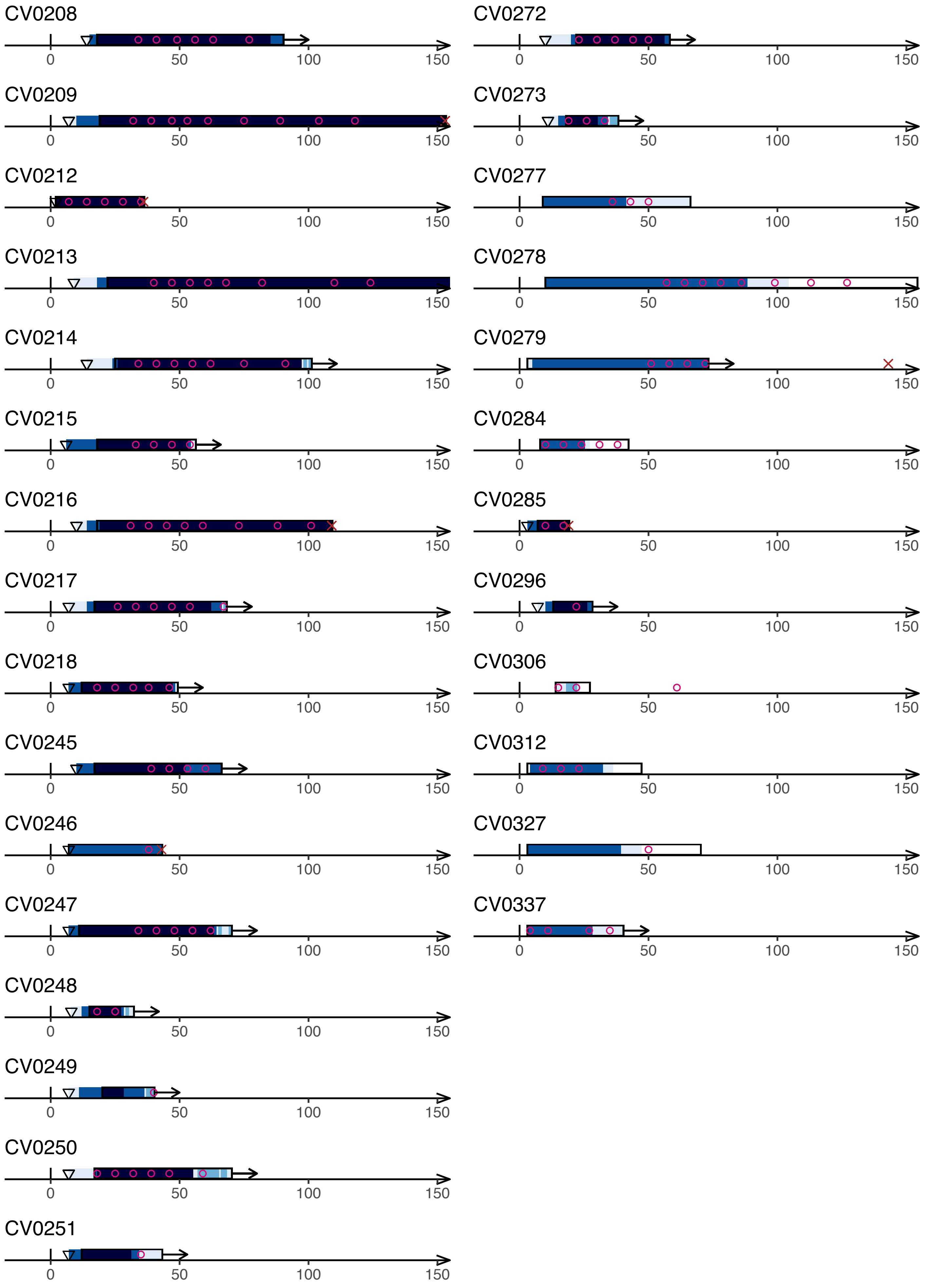
CV0208
CV0209
CV0212
CV0213
CV0214
CV0215
CV0216
CV0217
CV0218
CV0245
CV0246
CV0247
CV0248
CV0249
CV0250
CV0251
CV0272
CV0273
CV0277
CV0278
CV0279
CV0284
CV0285
CV0296
CV0306
CV0312
CV0327
CV0337
0
50
100
150

Data S2: Plots for the completed cell data, related to Figure 2

A) Boxplots showing absolute counts (cells/uL) for all the cell populations, split by severity categories and 12-day time bins post screening (group A) or symptom onset (groups B-E). Points are coloured based on asymptomatic or symptomatic classification for categories A and B respectively, normal or abnormal chest radiology (group C), and type of respiratory support at time of sampling (groups D and E), as per the key colour provided. **B)** Mixed-effects model with quadratic time trend showing the longitudinal trajectories of all the cell populations over time, grouped by severity. Nominal and adjusted p-values for the time x severity group interaction term are reported. Grey band in **A)** and **B)** indicates interquartile range of the corresponding population in healthy controls. NIV/HF, non-invasive ventilation/high-flow oxygen; vent, mechanical ventilation; ECMO, extracorporeal membrane oxygenation.

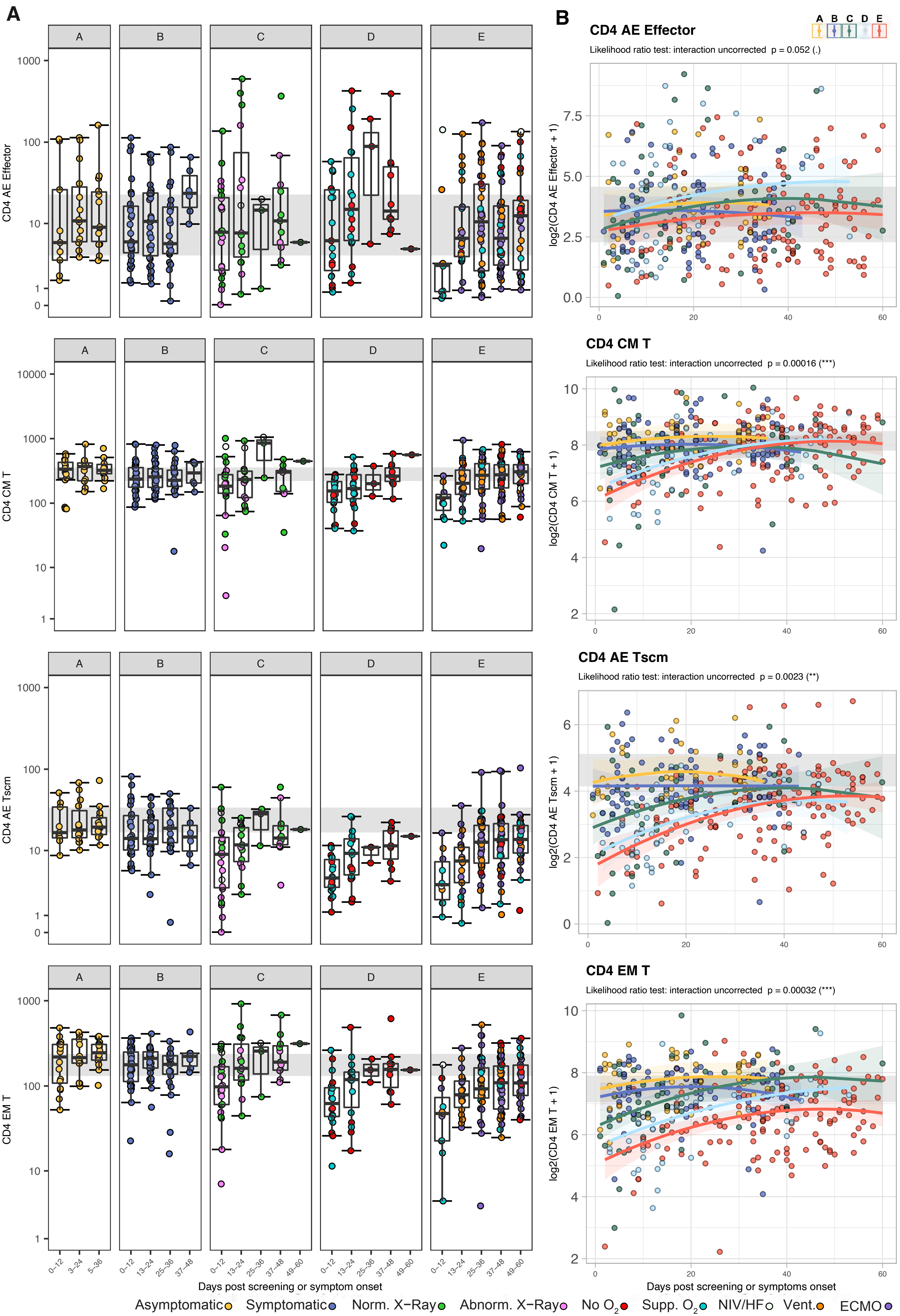

A
B
CD4 AE Effector
Likelihood ratio test: interaction uncorrected p = 0.052 (.)
log2(CD4 AE Effector + 1)
CD4 CM T
Likelihood ratio test: interaction uncorrected p = 0.00016 (***)
log2(CD4 CM T + 1)
CD4 AE Tscm
Likelihood ratio test: interaction uncorrected p = 0.0023 (**)
log2(CD4 AE Tscm + 1)
CD4 EM T
Likelihood ratio test: interaction uncorrected p = 0.00032 (***)
log2(CD4 EM T + 1)
CD4 AE Effector
CD4 CM T
CD4 AE Tscm
CD4 EM T
0–12 13–24 25–36 37–48 49–60
Days post screening or symptom onset
Days post screening or symptoms onset
Asymptomatic
Symptomatic
Norm. X−Ray
Abnorm. X−Ray
No O_2
Supp. O_2
NIV/HFO
Vent.
ECMO

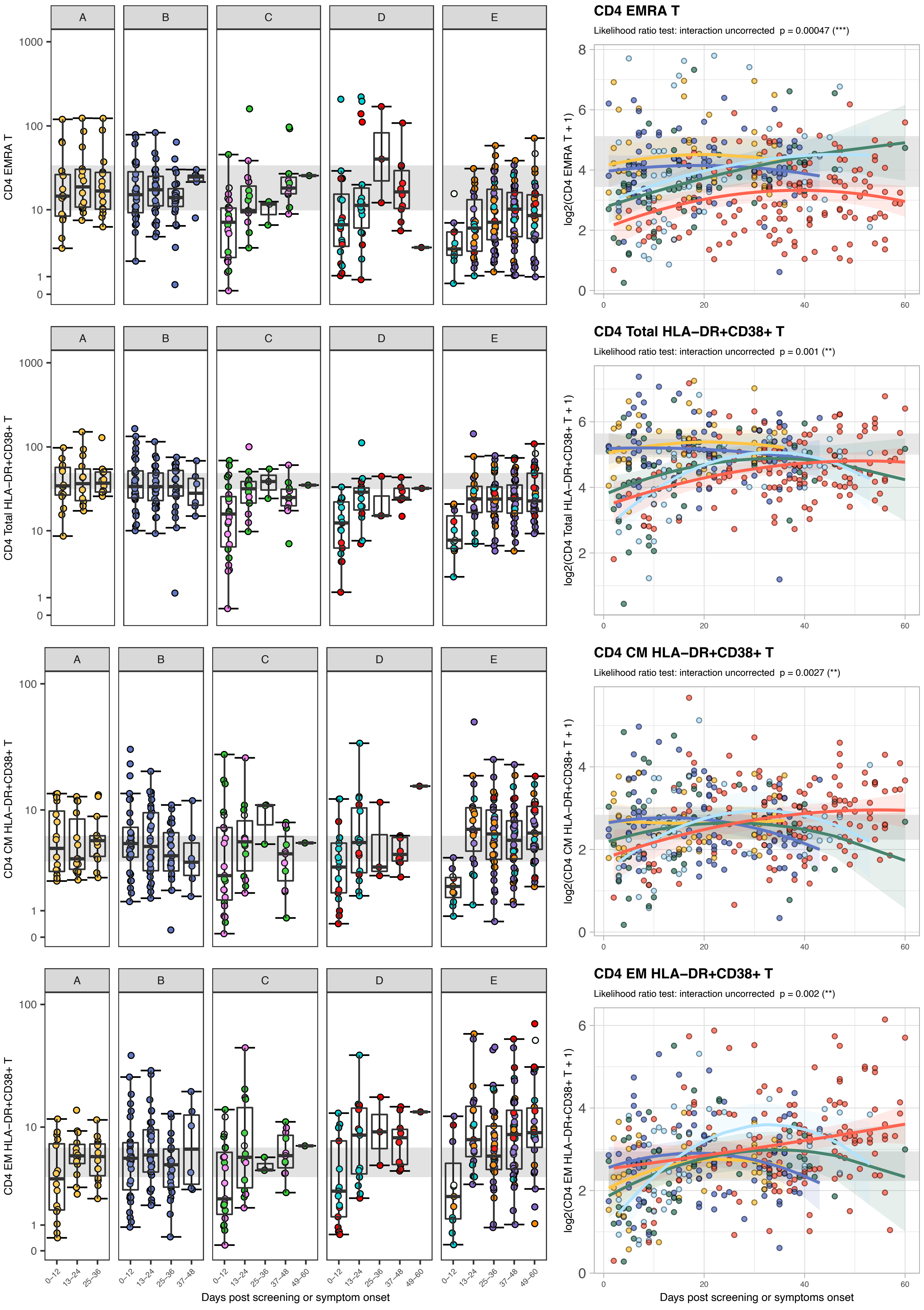

CD4 EMRA T
Likelihood ratio test: interaction uncorrected p = 0.00047 (***)
CD4 Total HLA−DR+CD38+ T
Likelihood ratio test: interaction uncorrected p = 0.001 (**)
CD4 CM HLA−DR+CD38+ T
Likelihood ratio test: interaction uncorrected p = 0.0027 (**)
CD4 EM HLA−DR+CD38+ T
Likelihood ratio test: interaction uncorrected p = 0.002 (**)
A
B
C
D
E
CD4 EMRA T
CD4 Total HLA−DR+CD38+ T
CD4 CM HLA−DR+CD38+ T
CD4 EM HLA−DR+CD38+ T
log2(CD4 EMRA T + 1)
log2(CD4 Total HLA−DR+CD38+ T + 1)
log2(CD4 CM HLA−DR+CD38+ T + 1)
log2(CD4 EM HLA−DR+CD38+ T + 1)
0–12
13–24
25–36
37–48
49–60
Days post screening or symptom onset
Days post screening or symptoms onset

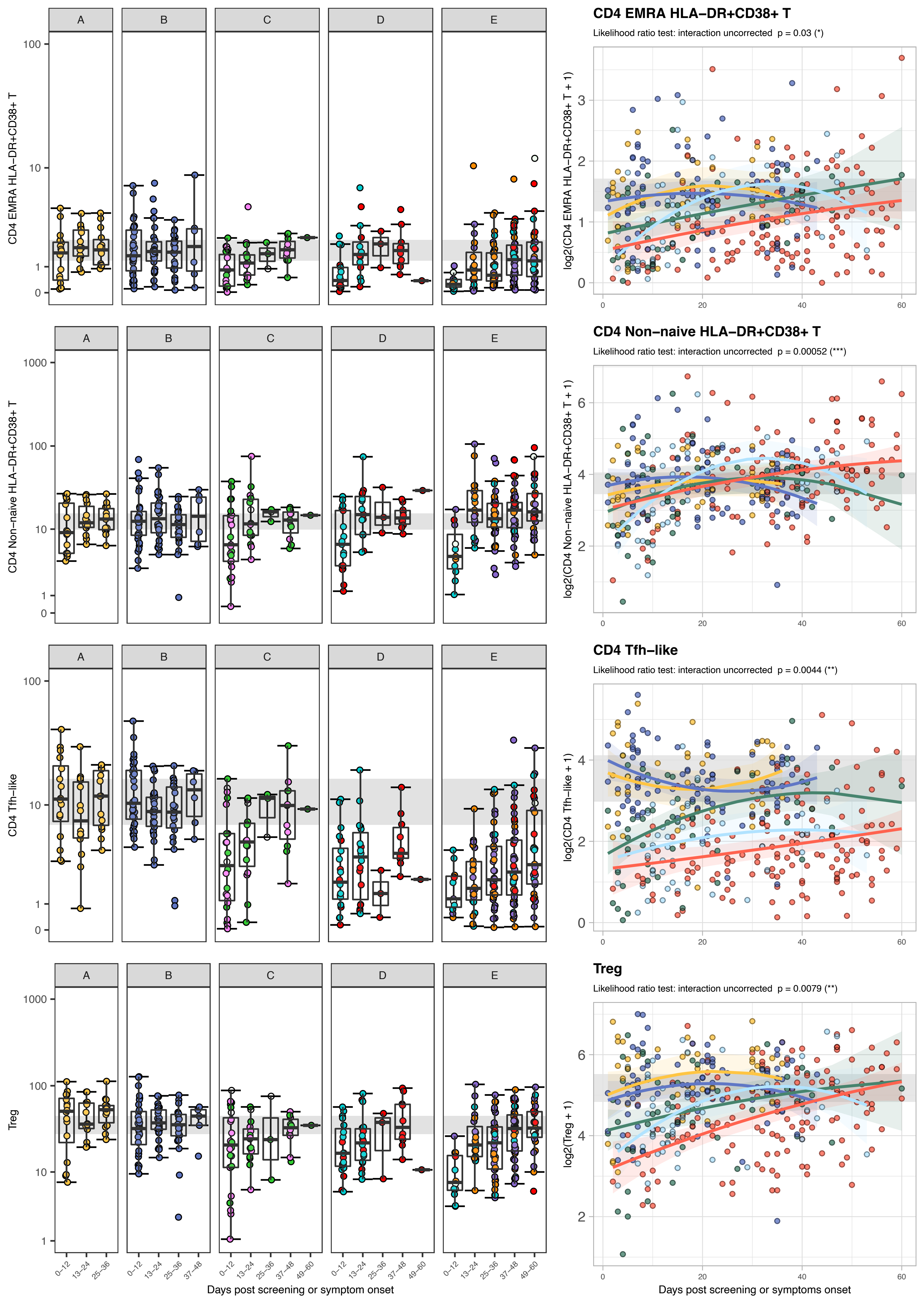

CD4 EMRA HLA−DR+CD38+ T
Likelihood ratio test: interaction uncorrected p = 0.03 (*)
log2(CD4 EMRA HLA−DR+CD38+ T + 1)
CD4 Non−naive HLA−DR+CD38+ T
Likelihood ratio test: interaction uncorrected p = 0.00052 (***)
log2(CD4 Non−naive HLA−DR+CD38+ T + 1)
CD4 Tfh−like
Likelihood ratio test: interaction uncorrected p = 0.0044 (**)
log2(CD4 Tfh−like + 1)
Treg
Likelihood ratio test: interaction uncorrected p = 0.0079 (**)
log2(Treg + 1)
A
B
C
D
E
0−12
13−24
25−36
37−48
49−60
Days post screening or symptom onset
Days post screening or symptoms onset

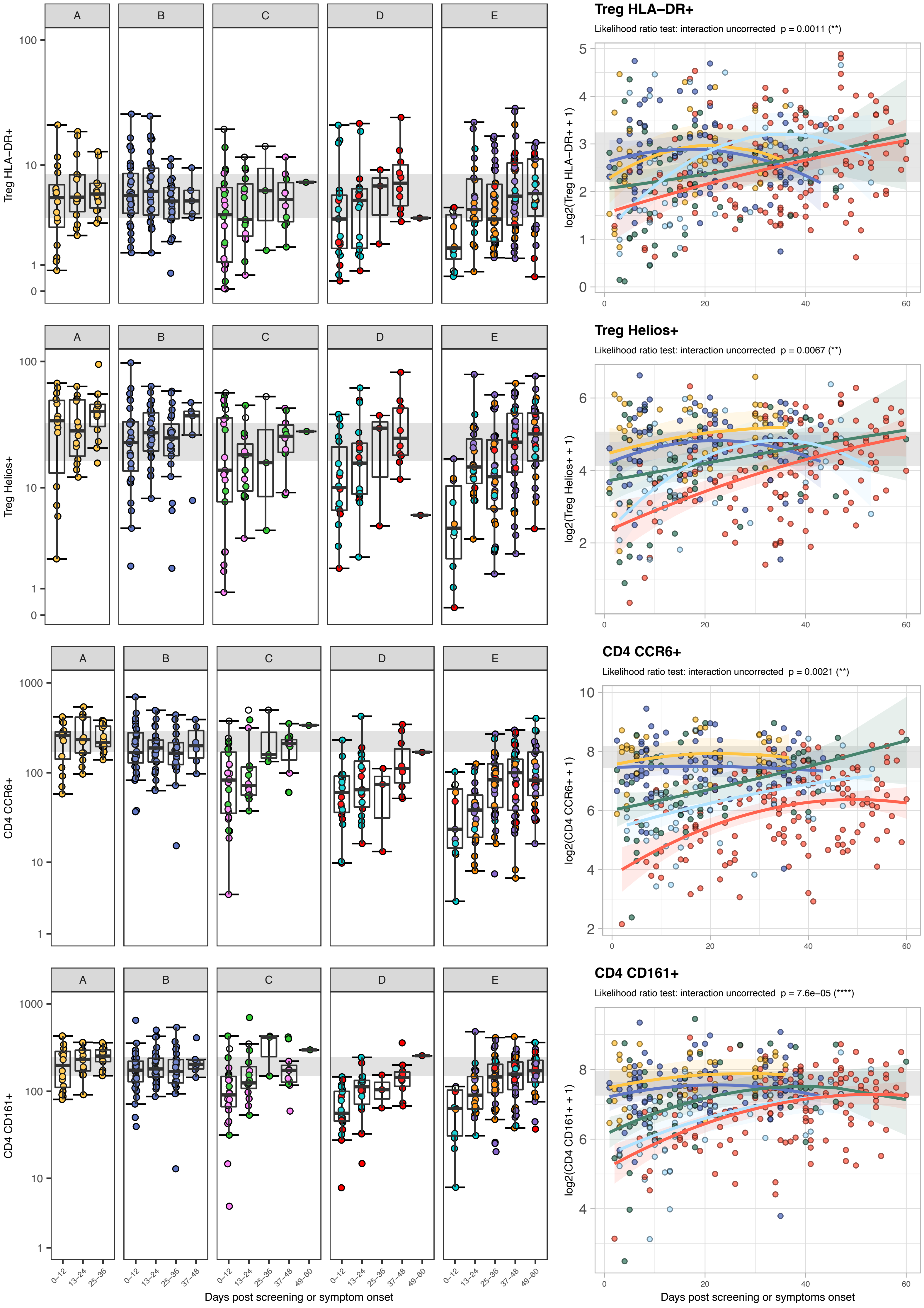

Treg HLA−DR+
Likelihood ratio test: interaction uncorrected p = 0.0011 (**)
Treg Helios+
Likelihood ratio test: interaction uncorrected p = 0.0067 (**)
CD4 CCR6+
Likelihood ratio test: interaction uncorrected p = 0.0021 (**)
CD4 CD161+
Likelihood ratio test: interaction uncorrected p = 7.6e−05 (****)
A
B
C
D
E
Treg HLA−DR+
Treg Helios+
CD4 CCR6+
CD4 CD161+
log2(Treg HLA−DR+ + 1)
log2(Treg Helios+ + 1)
log2(CD4 CCR6+ + 1)
log2(CD4 CD161+ + 1)
0−12
13−24
25−36
37−48
49−60
Days post screening or symptom onset
Days post screening or symptoms onset

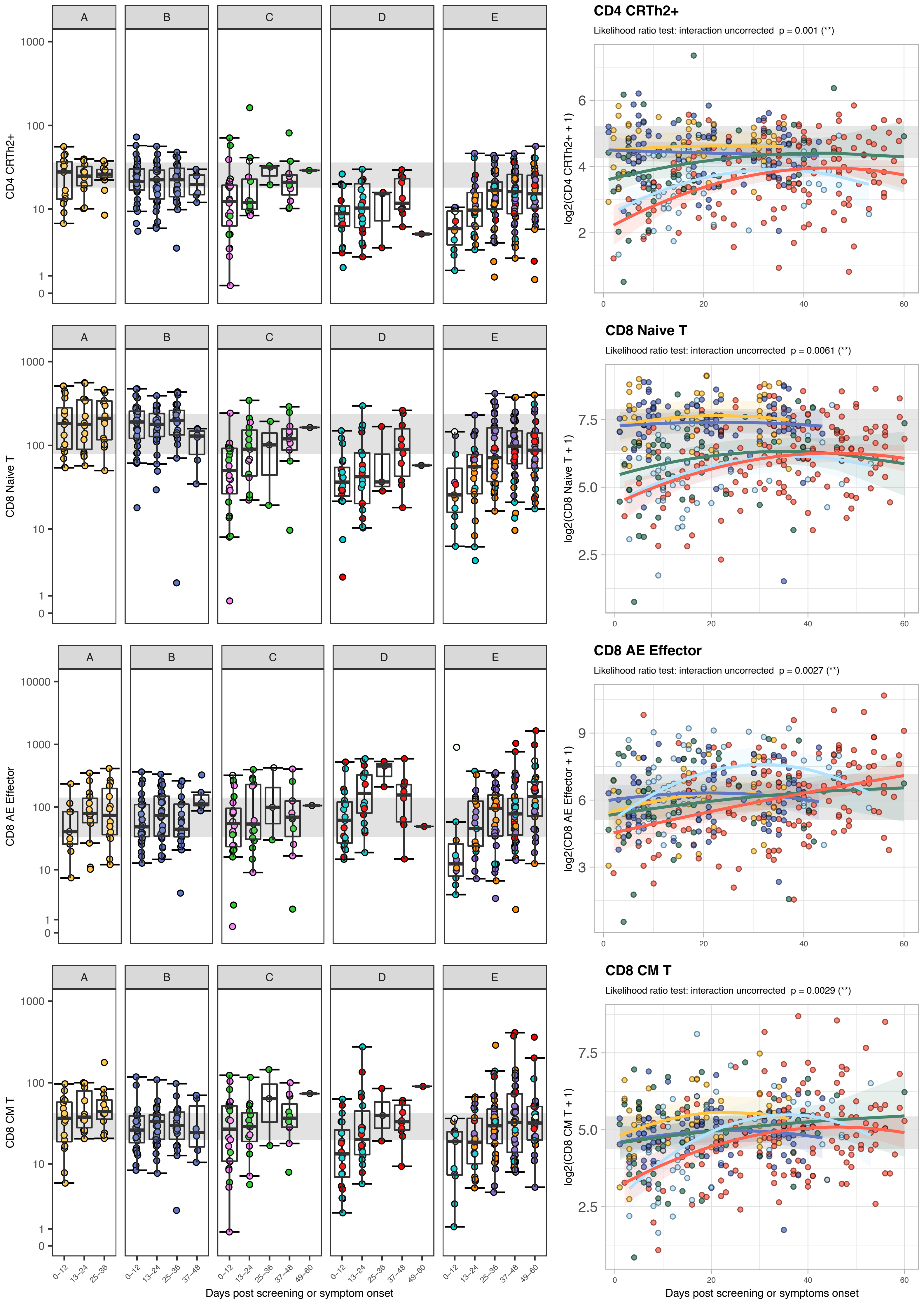
A
B
C
D
E
CD4 CRTh2+
CD4 CRTh2+
Likelihood ratio test: interaction uncorrected p = 0.001 (**)
log2(CD4 CRTh2+ + 1)
CD8 Naive T
CD8 Naive T
Likelihood ratio test: interaction uncorrected p = 0.0061 (**)
log2(CD8 Naive T + 1)
CD8 AE Effector
CD8 AE Effector
Likelihood ratio test: interaction uncorrected p = 0.0027 (**)
log2(CD8 AE Effector + 1)
CD8 CM T
CD8 CM T
Likelihood ratio test: interaction uncorrected p = 0.0029 (**)
log2(CD8 CM T + 1)
0–12
13–24
25–36
37–48
49–60
Days post screening or symptom onset
Days post screening or symptoms onset

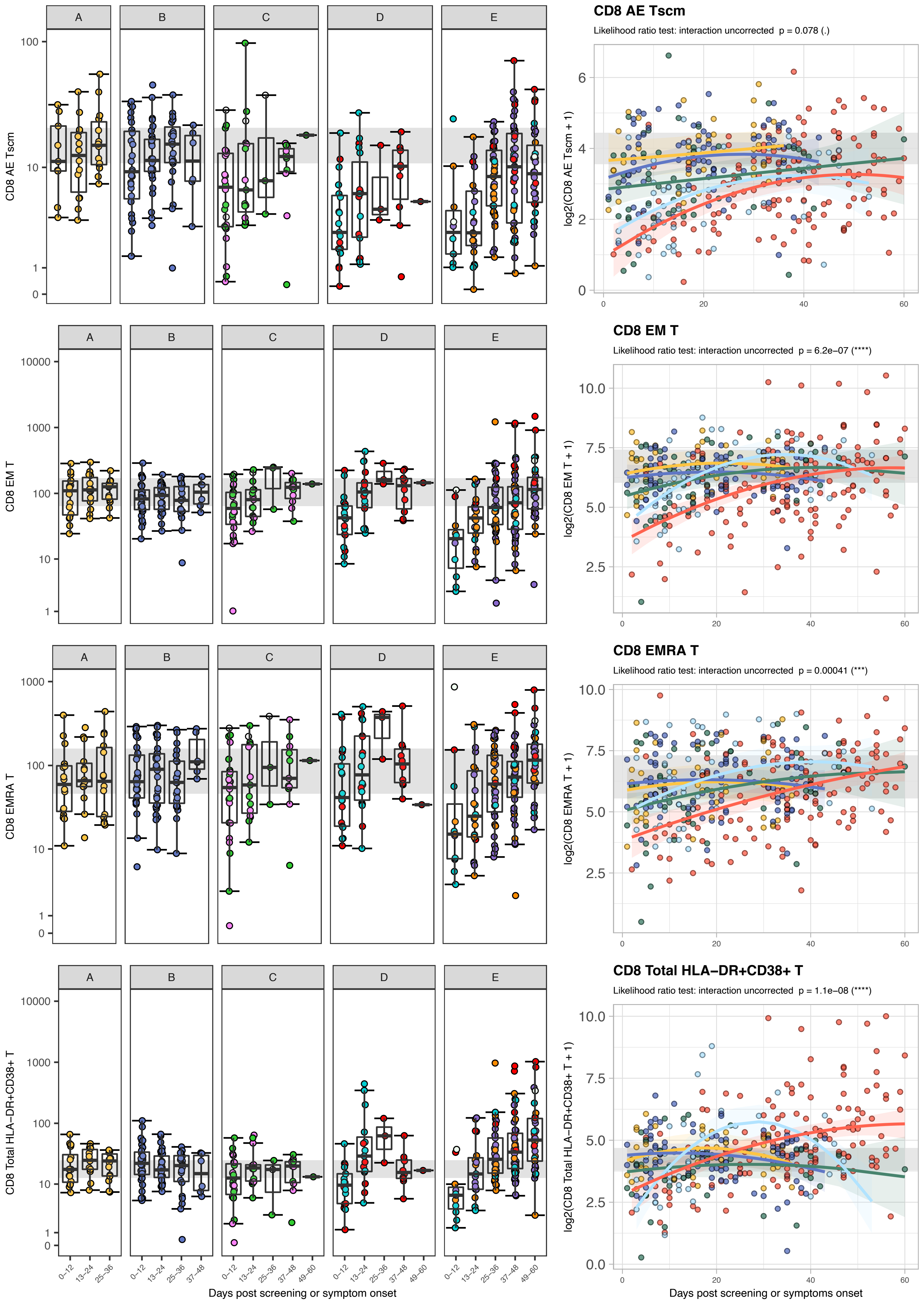

CD8 AE Tscm
Likelihood ratio test: interaction uncorrected p = 0.078 (.)
CD8 EM T
Likelihood ratio test: interaction uncorrected p = 6.2e−07 (****)
CD8 EMRA T
Likelihood ratio test: interaction uncorrected p = 0.00041 (***)
CD8 Total HLA−DR+CD38+ T
Likelihood ratio test: interaction uncorrected p = 1.1e−08 (****)
A
B
C
D
E
CD8 AE Tscm
CD8 EM T
CD8 EMRA T
CD8 Total HLA−DR+CD38+ T
log2(CD8 AE Tscm + 1)
log2(CD8 EM T + 1)
log2(CD8 EMRA T + 1)
log2(CD8 Total HLA−DR+CD38+ T + 1)
0–12
13–24
25–36
37–48
49–60
Days post screening or symptom onset
Days post screening or symptoms onset

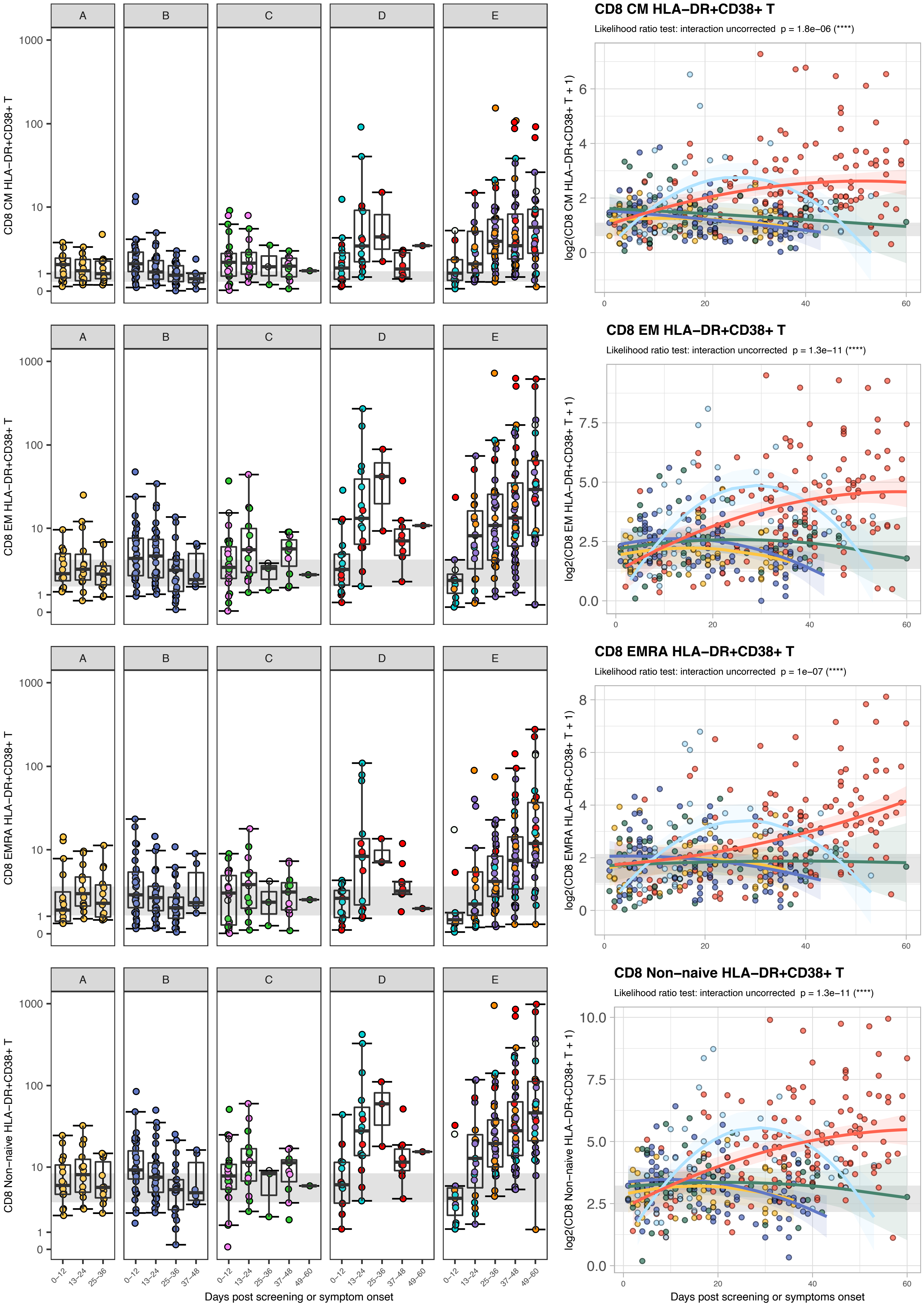

CD8 CM HLA−DR+CD38+ T
Likelihood ratio test: interaction uncorrected p = 1.8e−06 (****)
log2(CD8 CM HLA−DR+CD38+ T + 1)
CD8 EM HLA−DR+CD38+ T
Likelihood ratio test: interaction uncorrected p = 1.3e−11 (****)
log2(CD8 EM HLA−DR+CD38+ T + 1)
CD8 EMRA HLA−DR+CD38+ T
Likelihood ratio test: interaction uncorrected p = 1e−07 (****)
log2(CD8 EMRA HLA−DR+CD38+ T + 1)
CD8 Non−naive HLA−DR+CD38+ T
Likelihood ratio test: interaction uncorrected p = 1.3e−11 (****)
log2(CD8 Non−naive HLA−DR+CD38+ T + 1)
A
B
C
D
E
0–12
13–24
25–36
37–48
49–60
Days post screening or symptom onset
Days post screening or symptoms onset

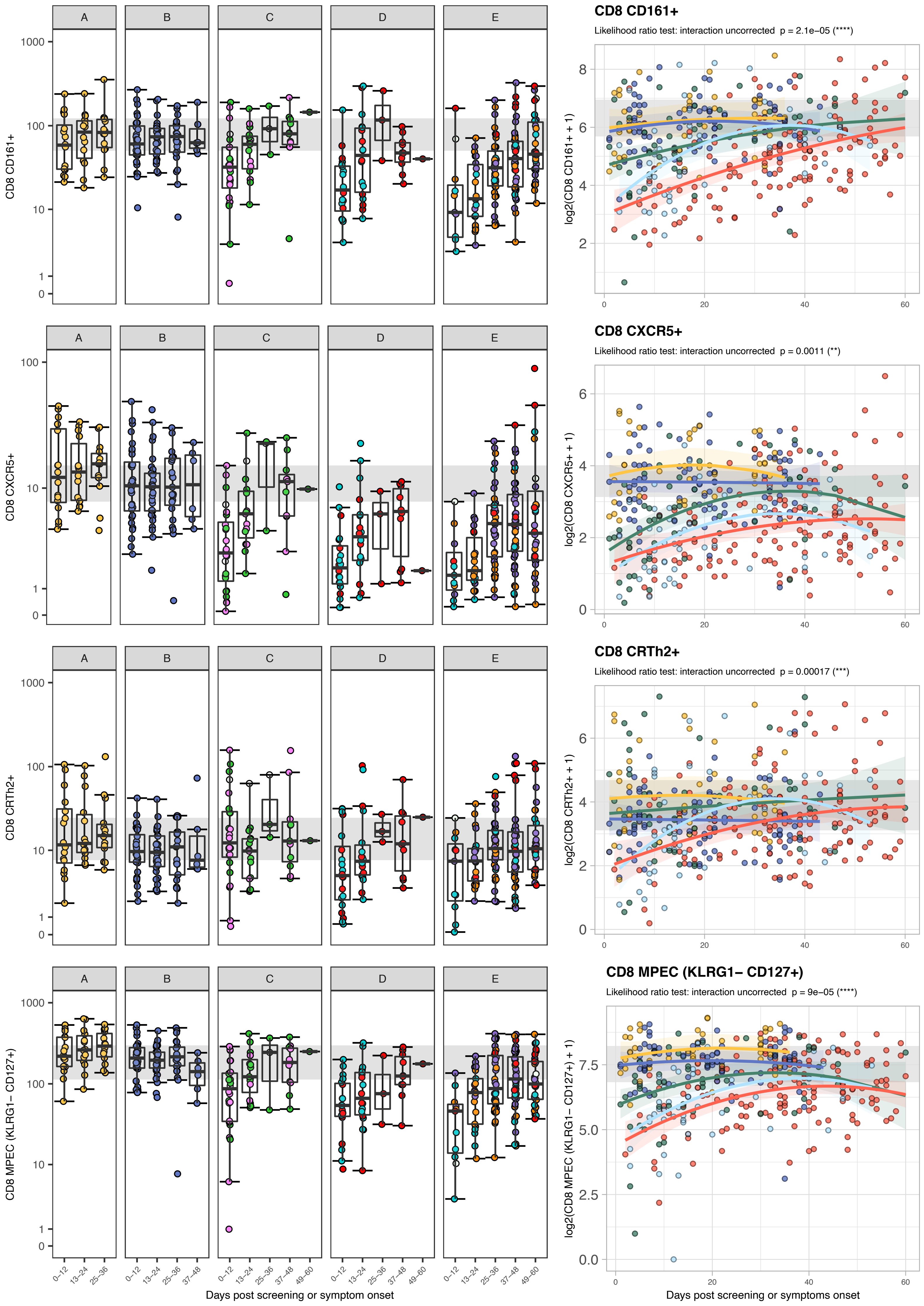
A
B
C
D
E
CD8 CD161+
Likelihood ratio test: interaction uncorrected p = 2.1e−05 (****)
log2(CD8 CD161+ + 1)
CD8 CXCR5+
Likelihood ratio test: interaction uncorrected p = 0.0011 (**)
log2(CD8 CXCR5+ + 1)
CD8 CRTh2+
Likelihood ratio test: interaction uncorrected p = 0.00017 (***)
log2(CD8 CRTh2+ + 1)
CD8 MPEC (KLRG1− CD127+)
Likelihood ratio test: interaction uncorrected p = 9e−05 (****)
log2(CD8 MPEC (KLRG1− CD127+) + 1)
0–12
13–24
25–36
37–48
49–60
Days post screening or symptom onset
Days post screening or symptoms onset

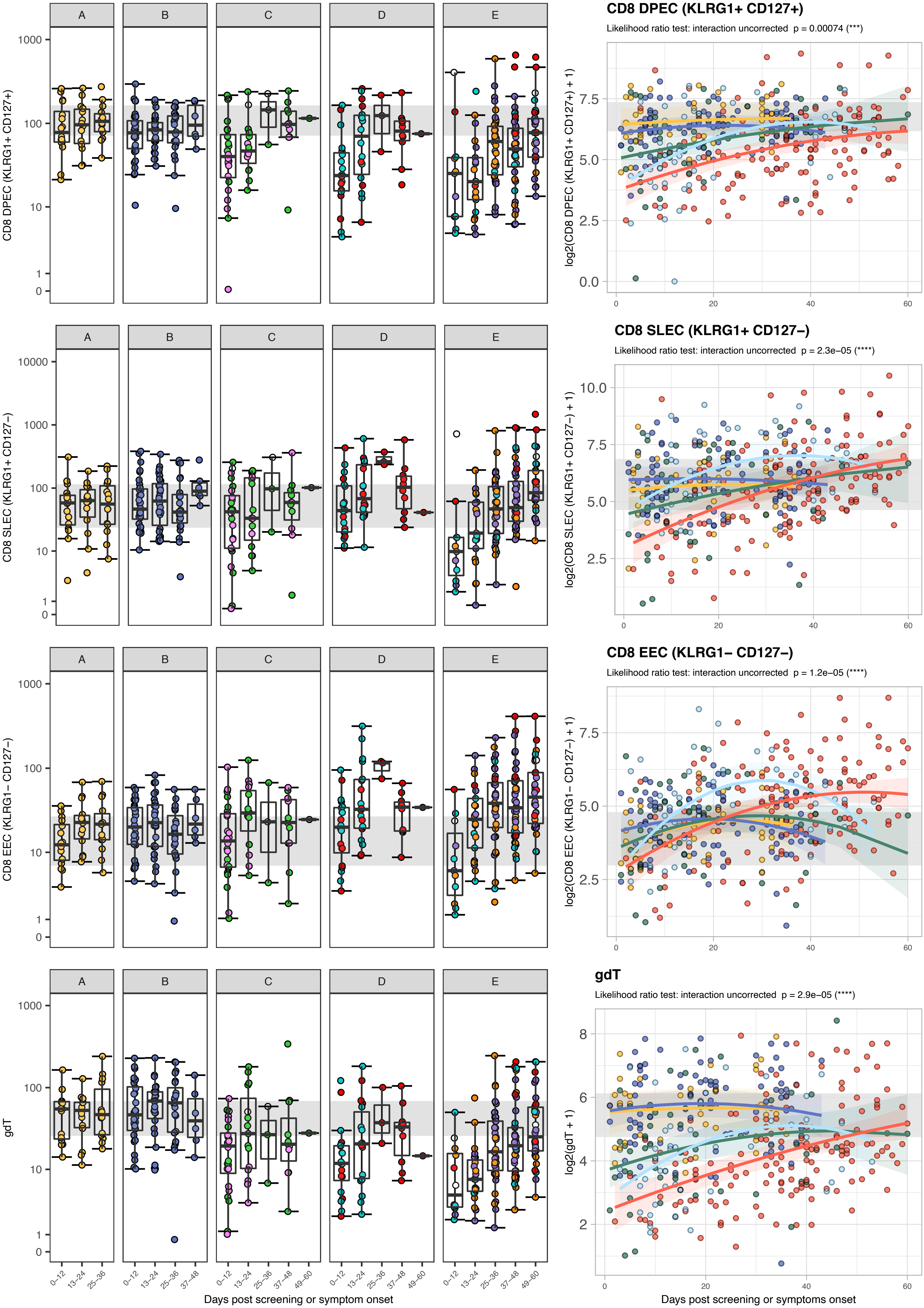
CD8 DPEC (KLRG1+ CD127+)
Likelihood ratio test: interaction uncorrected p = 0.00074 (***)
CD8 DPEC (KLRG1+ CD127+)
log2(CD8 DPEC (KLRG1+ CD127+) + 1)
CD8 SLEC (KLRG1+ CD127−)
Likelihood ratio test: interaction uncorrected p = 2.3e−05 (****)
CD8 SLEC (KLRG1+ CD127−)
log2(CD8 SLEC (KLRG1+ CD127−) + 1)
CD8 EEC (KLRG1− CD127−)
Likelihood ratio test: interaction uncorrected p = 1.2e−05 (****)
CD8 EEC (KLRG1− CD127−)
log2(CD8 EEC (KLRG1− CD127−) + 1)
gdT
Likelihood ratio test: interaction uncorrected p = 2.9e−05 (****)
gdT
log2(gdT + 1)
A
B
C
D
E
0–12
13–24
25–36
37–48
49–60
Days post screening or symptom onset
Days post screening or symptoms onset

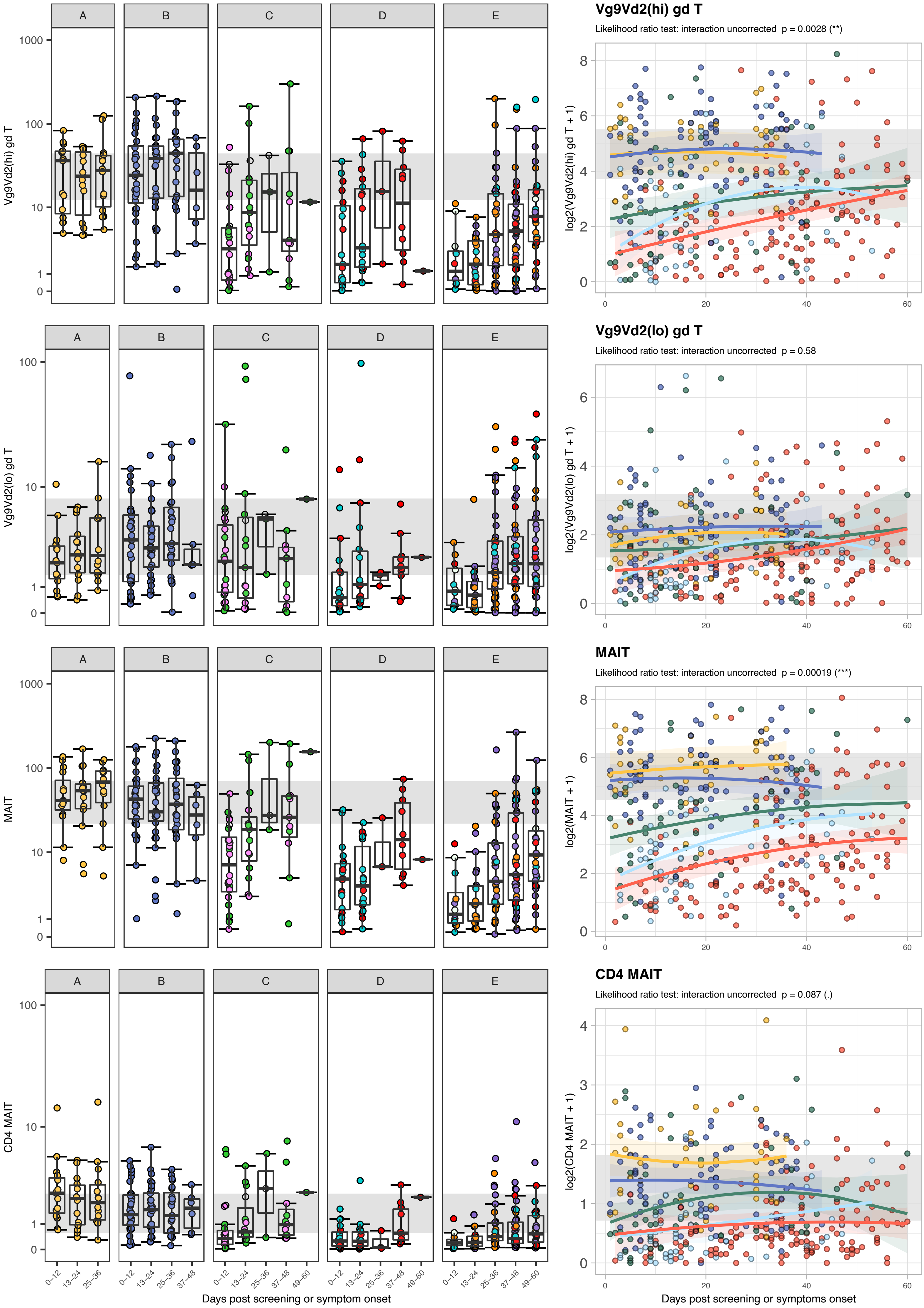
Vg9Vd2(hi) gd T
Likelihood ratio test: interaction uncorrected p = 0.0028 (**)
log2(Vg9Vd2(hi) gd T + 1)
Vg9Vd2(lo) gd T
Likelihood ratio test: interaction uncorrected p = 0.58
log2(Vg9Vd2(lo) gd T + 1)
MAIT
Likelihood ratio test: interaction uncorrected p = 0.00019 (***)
log2(MAIT + 1)
CD4 MAIT
Likelihood ratio test: interaction uncorrected p = 0.087 (.)
log2(CD4 MAIT + 1)
A
B
C
D
E
0–12
13–24
25–36
37–48
49–60
Days post screening or symptom onset
Days post screening or symptoms onset

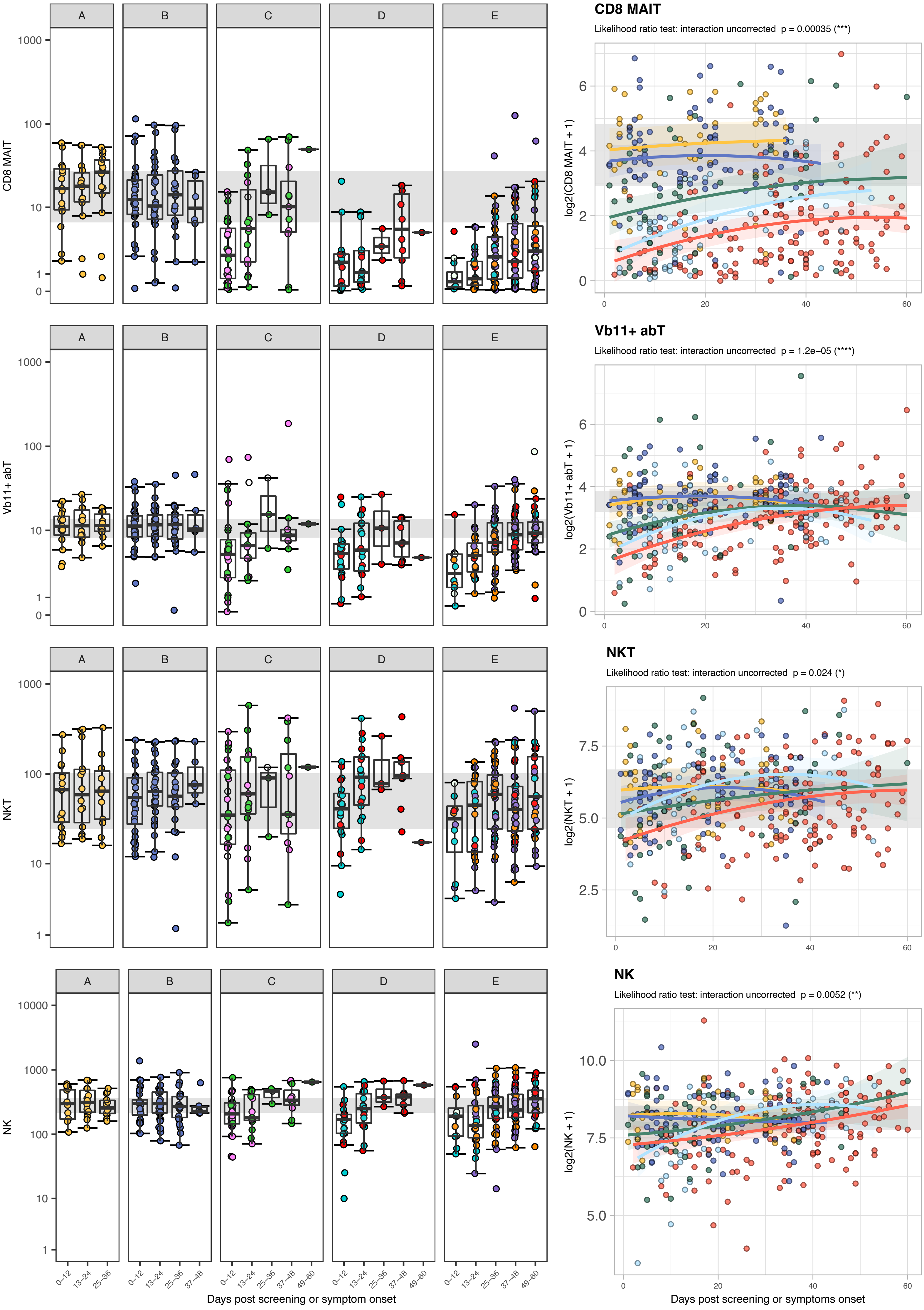

CD8 MAIT
Likelihood ratio test: interaction uncorrected p = 0.00035 (***)
log2(CD8 MAIT + 1)
Vb11+ abT
Likelihood ratio test: interaction uncorrected p = 1.2e−05 (****)
log2(Vb11+ abT + 1)
NKT
Likelihood ratio test: interaction uncorrected p = 0.024 (*)
log2(NKT + 1)
NK
Likelihood ratio test: interaction uncorrected p = 0.0052 (**)
log2(NK + 1)
A
B
C
D
E
0–12
13–24
25–36
37–48
49–60
Days post screening or symptom onset
Days post screening or symptoms onset

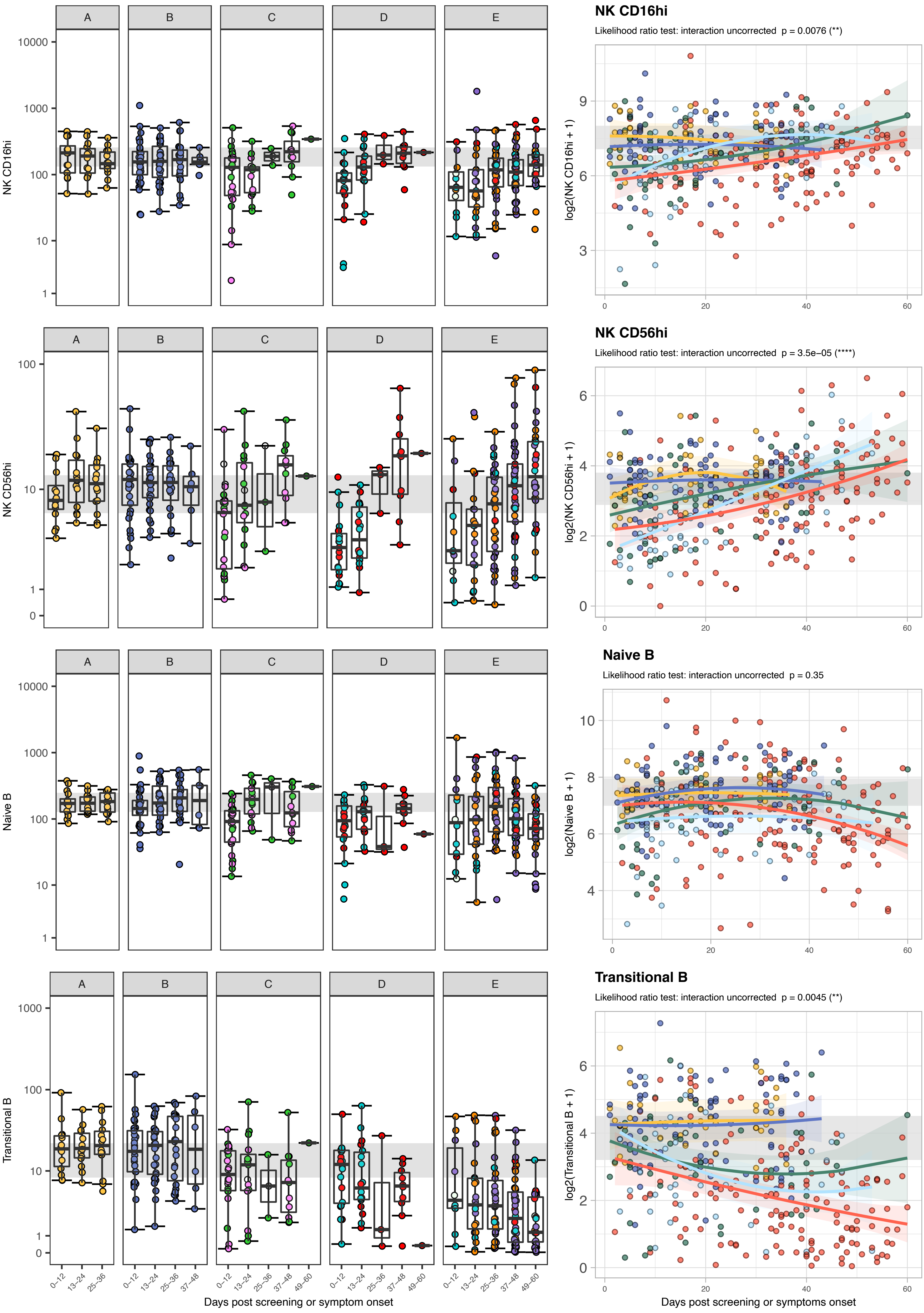

NK CD16hi
Likelihood ratio test: interaction uncorrected p = 0.0076 (**)
log2(NK CD16hi + 1)
NK CD56hi
Likelihood ratio test: interaction uncorrected p = 3.5e−05 (****)
log2(NK CD56hi + 1)
Naive B
Likelihood ratio test: interaction uncorrected p = 0.35
log2(Naive B + 1)
Transitional B
Likelihood ratio test: interaction uncorrected p = 0.0045 (**)
log2(Transitional B + 1)
A
B
C
D
E
Days post screening or symptom onset
Days post screening or symptoms onset
0–12
13–24
25–36
37–48
49–60

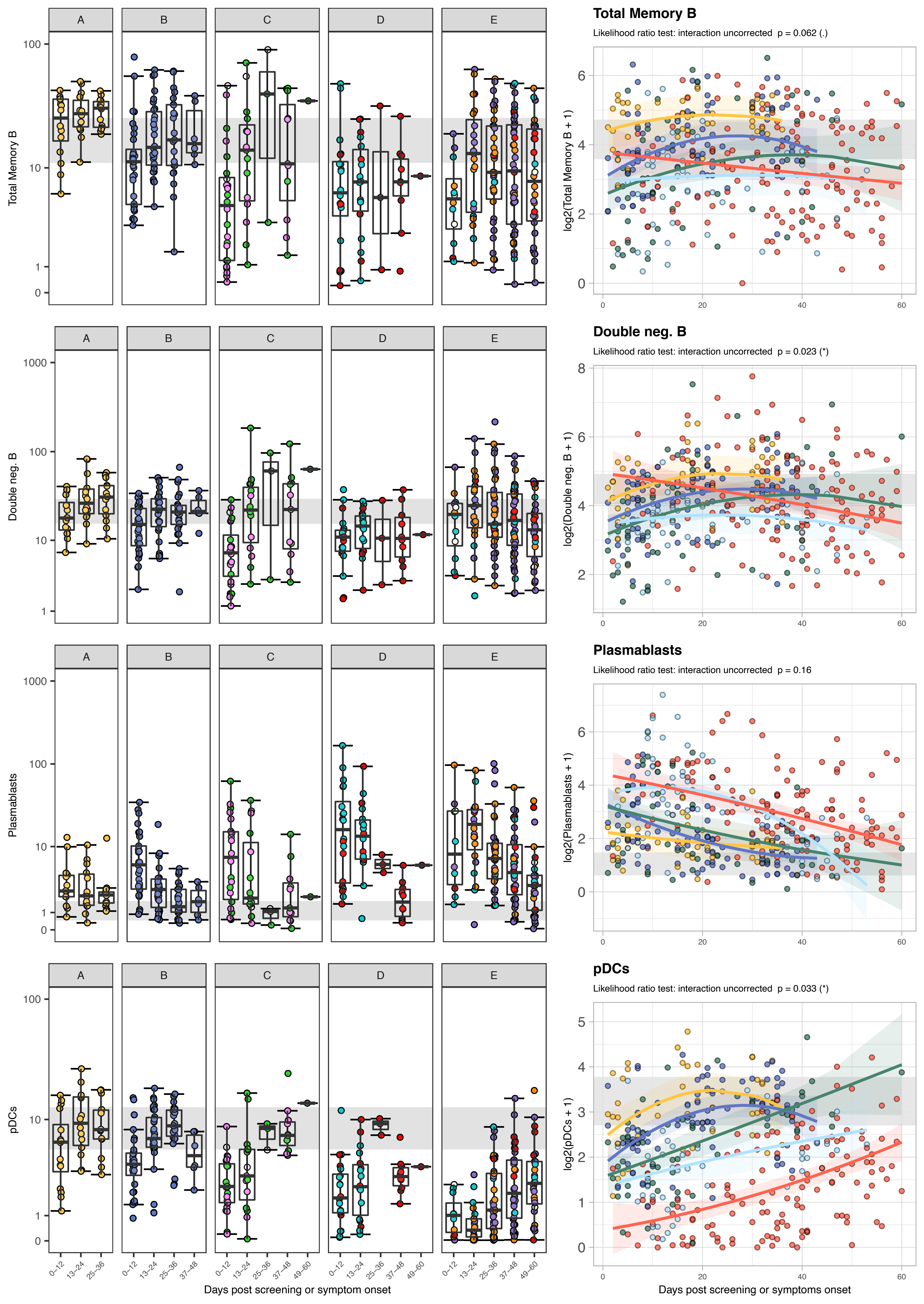
Total Memory B
Likelihood ratio test: interaction uncorrected p = 0.062 (.)
Double neg. B
Likelihood ratio test: interaction uncorrected p = 0.023 (*)
Plasmablasts
Likelihood ratio test: interaction uncorrected p = 0.16
pDCs
Likelihood ratio test: interaction uncorrected p = 0.033 (*)
A
B
C
D
E
Total Memory B
Double neg. B
Plasmablasts
pDCs
log2(Total Memory B + 1)
log2(Double neg. B + 1)
log2(Plasmablasts + 1)
log2(pDCs + 1)
0–12
13–24
25–36
37–48
49–60
Days post screening or symptom onset
Days post screening or symptoms onset

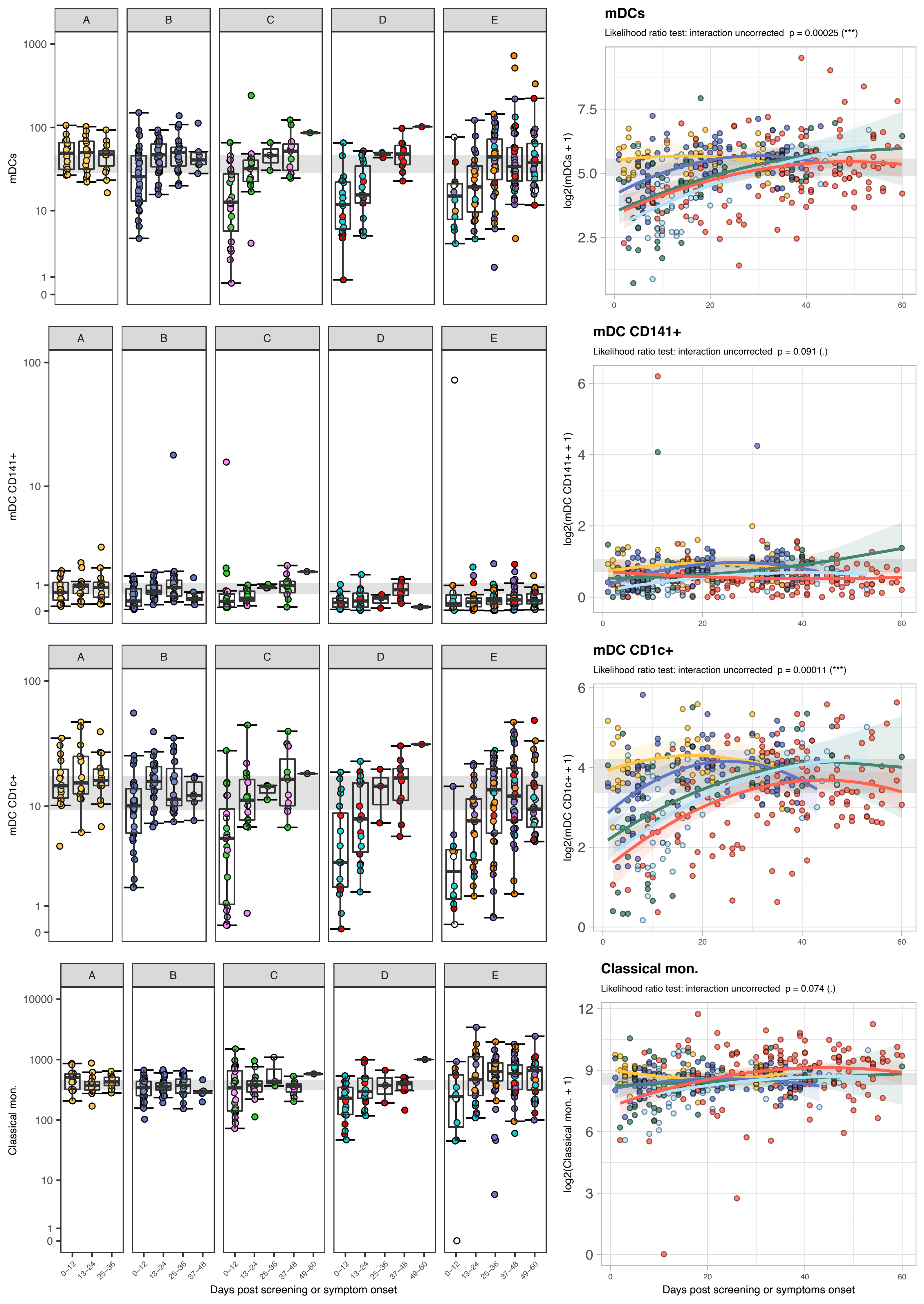
mDCs
Likelihood ratio test: interaction uncorrected p = 0.00025 (***)
log2(mDCs + 1)
mDC CD141+
Likelihood ratio test: interaction uncorrected p = 0.091 (.)
log2(mDC CD141+ + 1)
mDC CD1c+
Likelihood ratio test: interaction uncorrected p = 0.00011 (***)
log2(mDC CD1c+ + 1)
Classical mon.
Likelihood ratio test: interaction uncorrected p = 0.074 (.)
log2(Classical mon. + 1)
Days post screening or symptoms onset
A
B
C
D
E
Classical mon.
0–12
13–24
25–36
37–48
49–60
Days post screening or symptom onset

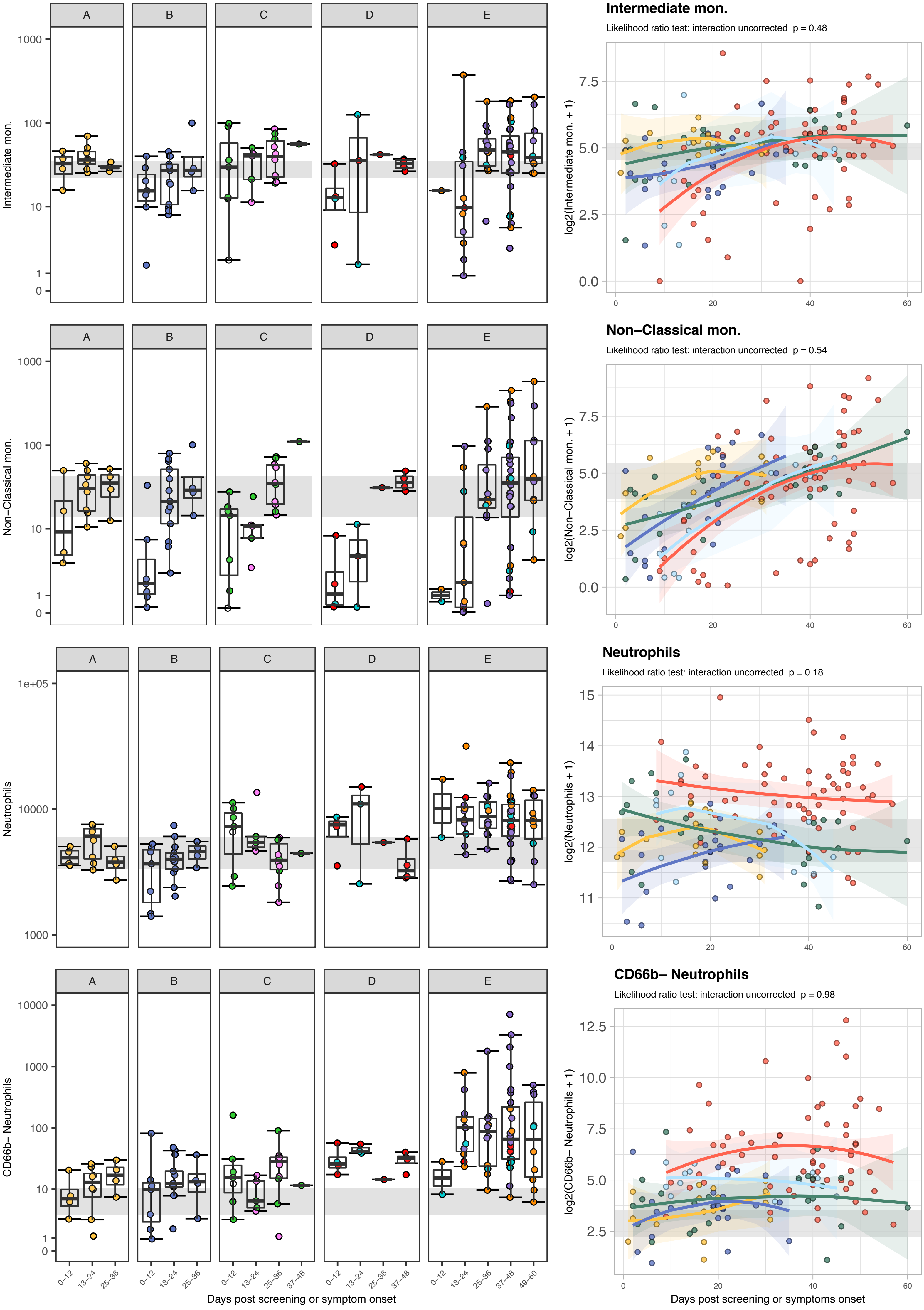

A
B
C
D
E
Intermediate mon.
Likelihood ratio test: interaction uncorrected p = 0.48
log2(Intermediate mon. + 1)
Non−Classical mon.
Likelihood ratio test: interaction uncorrected p = 0.54
log2(Non−Classical mon. + 1)
Neutrophils
Likelihood ratio test: interaction uncorrected p = 0.18
log2(Neutrophils + 1)
CD66b− Neutrophils
Likelihood ratio test: interaction uncorrected p = 0.98
log2(CD66b− Neutrophils + 1)
Days post screening or symptom onset
Days post screening or symptoms onset

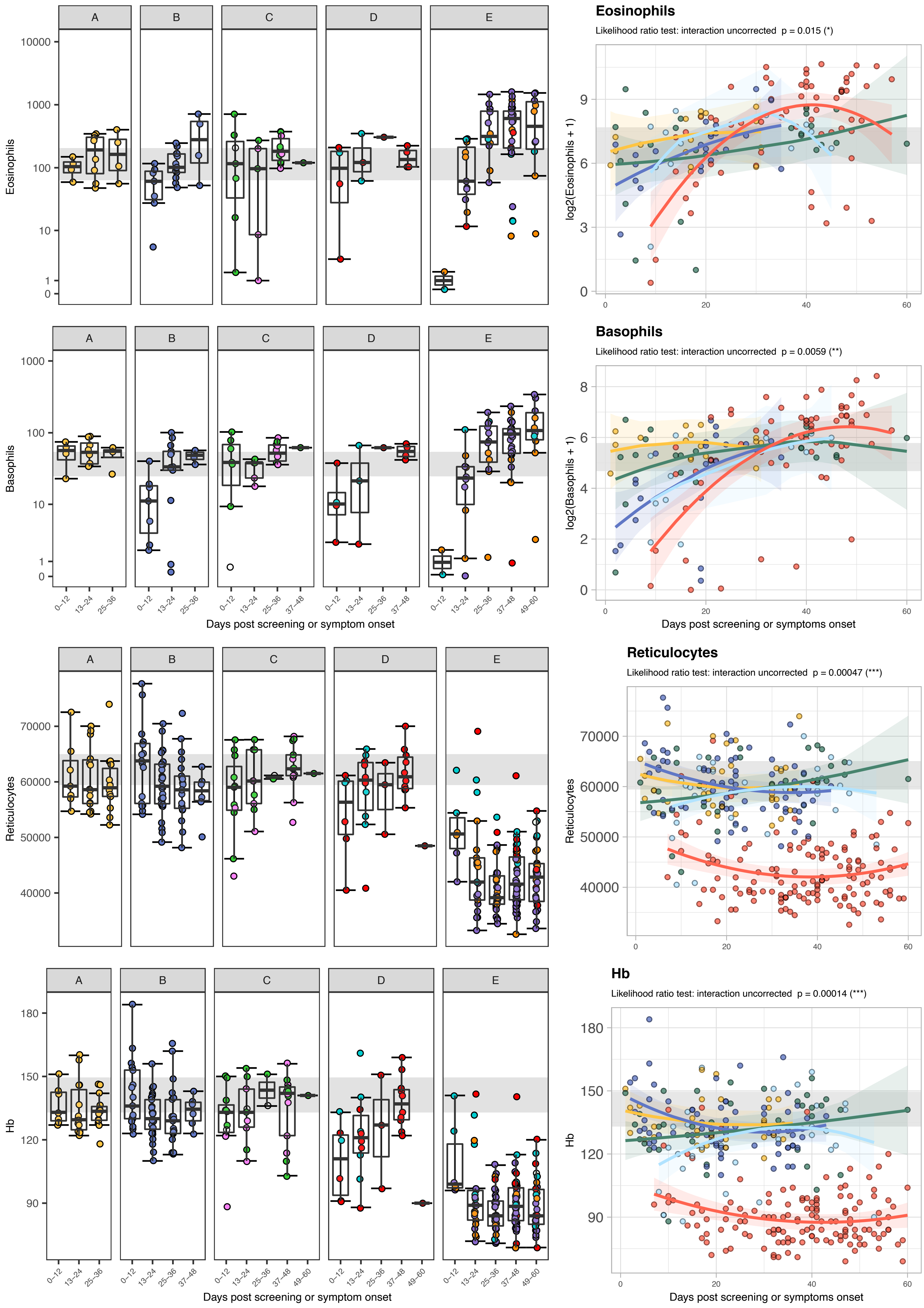

A
B
C
D
E
Eosinophils
10000
1000
100
10
1
0
Eosinophils
Likelihood ratio test: interaction uncorrected p = 0.015 (*)
log2(Eosinophils + 1)
Basophils
Likelihood ratio test: interaction uncorrected p = 0.0059 (**)
log2(Basophils + 1)
0–12
13–24
25–36
37–48
49–60
Days post screening or symptom onset
Days post screening or symptoms onset
Reticulocytes
Likelihood ratio test: interaction uncorrected p = 0.00047 (***)
70000
60000
50000
40000
Hb
Likelihood ratio test: interaction uncorrected p = 0.00014 (***)
180
150
120
90

Data S3: Differences in serum cytokines, complement components and immunophenotyping between patients in cluster 1 and cluster 2 as defined in Figure 4A

A) Mixed-effect model with quadratic time trend showing the longitudinal trajectories of inflammatory markers, and boxplot at time of sampling (<=10 days post symptom onset), for individuals in clusters 1 and 2. **B)** Mixed-effect model with quadratic time trend showing the longitudinal trajectories for all cell populations over time, for COVID-19 cases in clusters 1 and 2. Grey bands in **A)** and **B)** indicate the interquartile range of the corresponding measurements in HCs. Nominal and adjusted p-values for the time x cluster interaction term are reported.

A

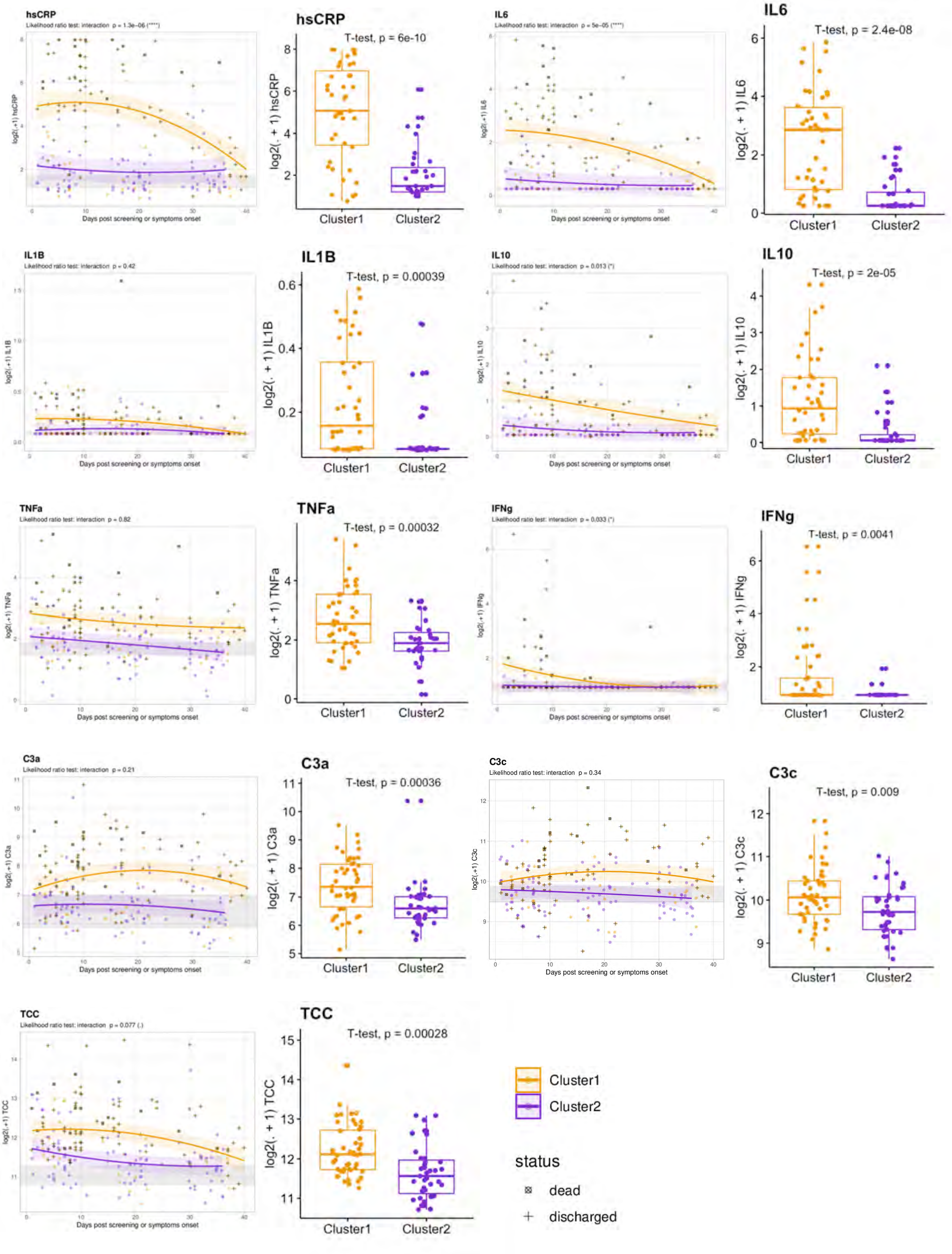
hsCRP
Likelihood ratio test: interaction p = 1.3e-06 (****)
log2(.+1) hsCRP
Days post screening or symptoms onset
hsCRP
T-test, p = 6e-10
log2(. + 1) hsCRP
Cluster1
Cluster2
IL6
Likelihood ratio test: interaction p = 5e-05 (****)
log2(.+1) IL6
Days post screening or symptoms onset
IL6
T-test, p = 2.4e-08
log2(. + 1) IL6
Cluster1
Cluster2
IL1B
Likelihood ratio test: interaction p = 0.42
log2(.+1) IL1B
Days post screening or symptoms onset
IL1B
T-test, p = 0.00039
log2(. + 1) IL1B
Cluster1
Cluster2
IL10
Likelihood ratio test: interaction p = 0.013 (*)
log2(.+1) IL10
Days post screening or symptoms onset
IL10
T-test, p = 2e-05
log2(. + 1) IL10
Cluster1
Cluster2
TNFa
Likelihood ratio test: interaction p = 0.82
log2(.+1) TNFa
Days post screening or symptoms onset
TNFa
T-test, p = 0.00032
log2(. + 1) TNFa
Cluster1
Cluster2
IFNg
Likelihood ratio test: interaction p = 0.033 (*)
log2(.+1) IFNg
Days post screening or symptoms onset
IFNg
T-test, p = 0.0041
log2(. + 1) IFNg
Cluster1
Cluster2
C3a
Likelihood ratio test: interaction p = 0.21
log2(.+1) C3a
Days post screening or symptoms onset
C3a
T-test, p = 0.00036
log2(. + 1) C3a
Cluster1
Cluster2
C3c
Likelihood ratio test: interaction p = 0.34
log2(.+1) C3c
Days post screening or symptoms onset
C3c
T-test, p = 0.009
log2(. + 1) C3c
Cluster1
Cluster2
TCC
Likelihood ratio test: interaction p = 0.077 (.)
log2(.+1) TCC
Days post screening or symptoms onset
TCC
T-test, p = 0.00028
log2(. + 1) TCC
Cluster1
Cluster2
Cluster1
Cluster2
status
dead
discharged

B

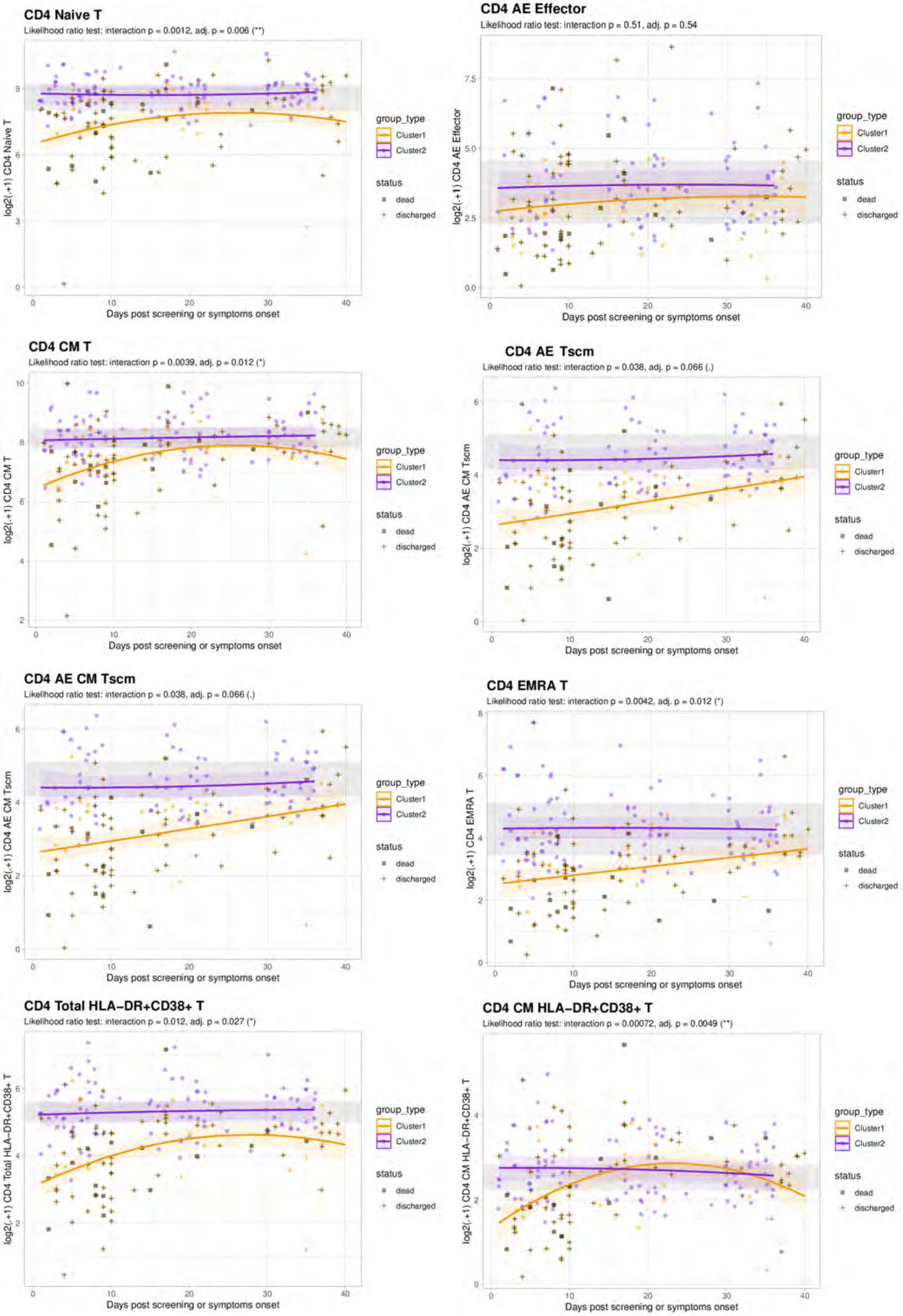
CD4 Naive T
Likelihood ratio test: interaction p = 0.0012, adj. p = 0.006 (**)
log2(.+1) CD4 Naive T
Days post screening or symptoms onset
CD4 AE Effector
Likelihood ratio test: interaction p = 0.51, adj. p = 0.54
log2(.+1) CD4 AE Effector
Days post screening or symptoms onset
CD4 CM T
Likelihood ratio test: interaction p = 0.0039, adj. p = 0.012 (*)
log2(.+1) CD4 CM T
Days post screening or symptoms onset
CD4 AE Tscm
Likelihood ratio test: interaction p = 0.038, adj. p = 0.066 (.)
log2(.+1) CD4 AE CM Tscm
Days post screening or symptoms onset
CD4 AE CM Tscm
Likelihood ratio test: interaction p = 0.038, adj. p = 0.066 (.)
log2(.+1) CD4 AE CM Tscm
Days post screening or symptoms onset
CD4 EMRA T
Likelihood ratio test: interaction p = 0.0042, adj. p = 0.012 (*)
log2(.+1) CD4 EMRA T
Days post screening or symptoms onset
CD4 Total HLA−DR+CD38+ T
Likelihood ratio test: interaction p = 0.012, adj. p = 0.027 (*)
log2(.+1) CD4 Total HLA−DR+CD38+ T
Days post screening or symptoms onset
CD4 CM HLA−DR+CD38+ T
Likelihood ratio test: interaction p = 0.00072, adj. p = 0.0049 (**)
log2(.+1) CD4 CM HLA−DR+CD38+ T
Days post screening or symptoms onset
group_type
Cluster1
Cluster2
status
dead
discharged

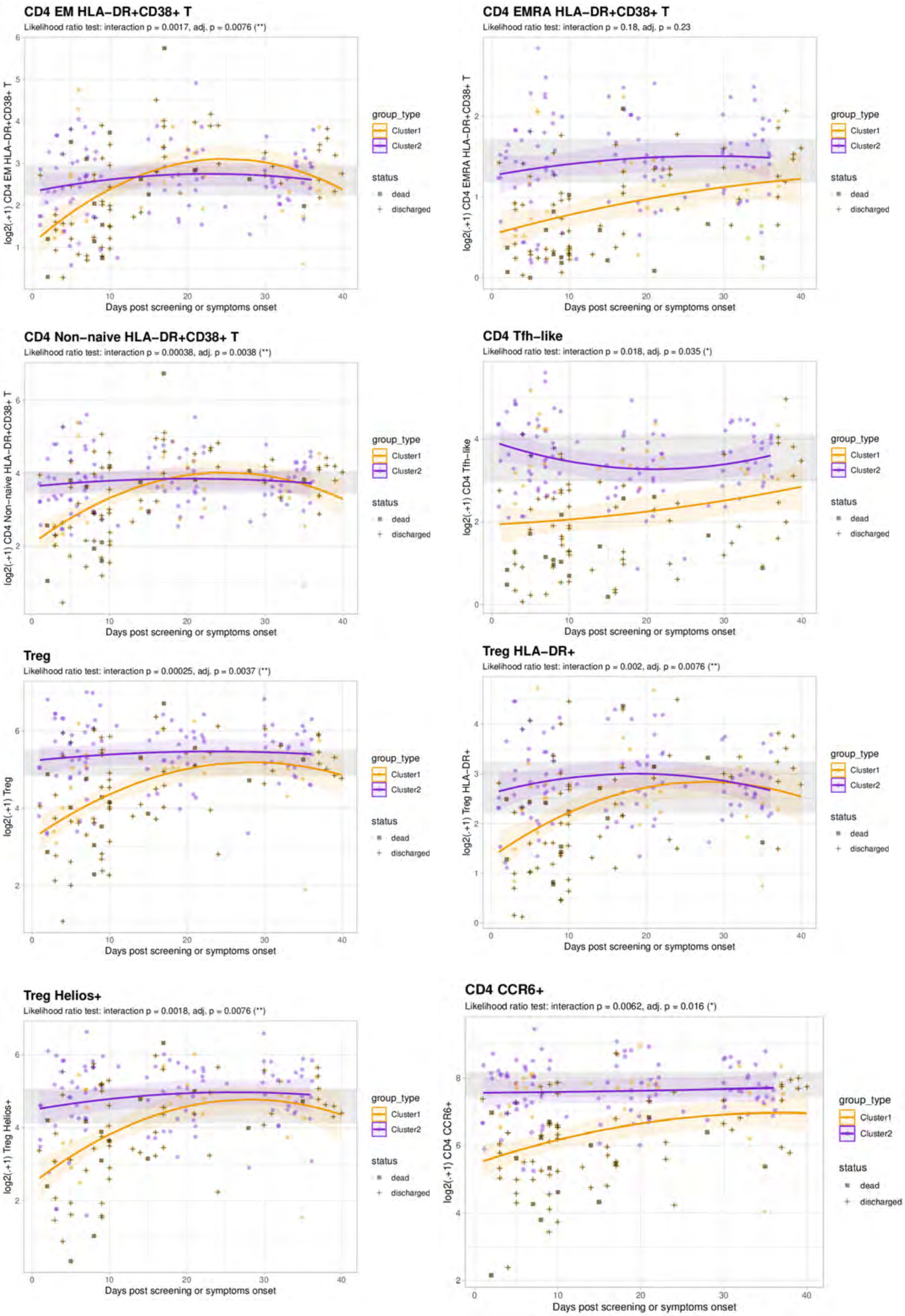
CD4 EM HLA−DR+CD38+ T
Likelihood ratio test: interaction p = 0.0017, adj. p = 0.0076 (**)
log2(.+1) CD4 EM HLA−DR+CD38+ T
Days post screening or symptoms onset
CD4 EMRA HLA−DR+CD38+ T
Likelihood ratio test: interaction p = 0.18, adj. p = 0.23
log2(.+1) CD4 EMRA HLA−DR+CD38+ T
Days post screening or symptoms onset
CD4 Non−naive HLA−DR+CD38+ T
Likelihood ratio test: interaction p = 0.00038, adj. p = 0.0038 (**)
log2(.+1) CD4 Non−naive HLA−DR+CD38+ T
Days post screening or symptoms onset
CD4 Tfh−like
Likelihood ratio test: interaction p = 0.018, adj. p = 0.035 (*)
log2(.+1) CD4 Tfh−like
Days post screening or symptoms onset
Treg
Likelihood ratio test: interaction p = 0.00025, adj. p = 0.0037 (**)
log2(.+1) Treg
Days post screening or symptoms onset
Treg HLA−DR+
Likelihood ratio test: interaction p = 0.002, adj. p = 0.0076 (**)
log2(.+1) Treg HLA−DR+
Days post screening or symptoms onset
Treg Helios+
Likelihood ratio test: interaction p = 0.0018, adj. p = 0.0076 (**)
log2(.+1) Treg Helios+
Days post screening or symptoms onset
CD4 CCR6+
Likelihood ratio test: interaction p = 0.0062, adj. p = 0.016 (*)
log2(.+1) CD4 CCR6+
Days post screening or symptoms onset
group_type
Cluster1
Cluster2
status
dead
discharged

CD4 CD161+

Likelihood ratio test: interaction p = 0.00059, adj. p = 0.0048 (**)

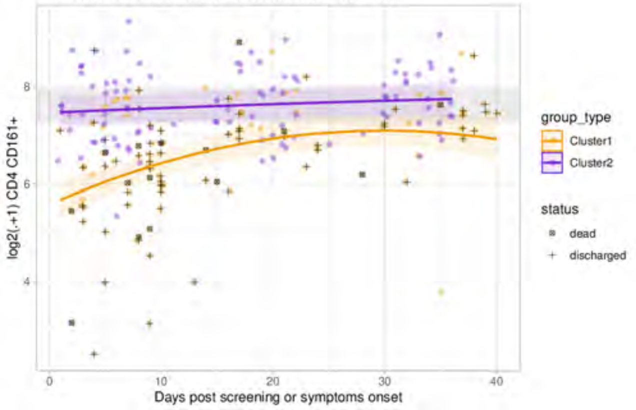

CD4 CRTh2+

Likelihood ratio test: interaction p = 0.00038, adj. p = 0.0038 (**)

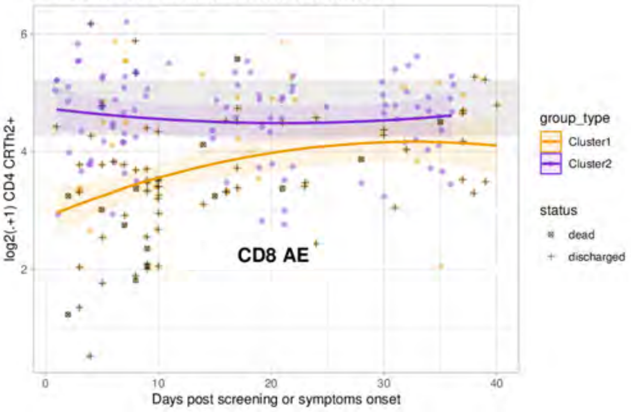

CD8 Naive T

Likelihood ratio test: interaction p = 0.013, adj. p = 0.027 (*)

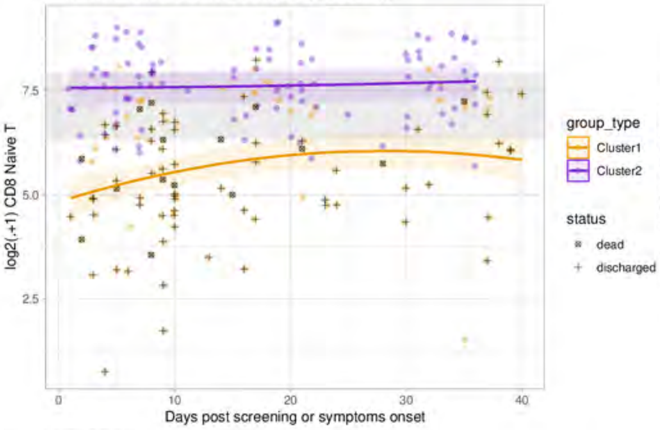

CD8 AE Effector

Likelihood ratio test: interaction p = 0.017, adj. p = 0.034 (*)

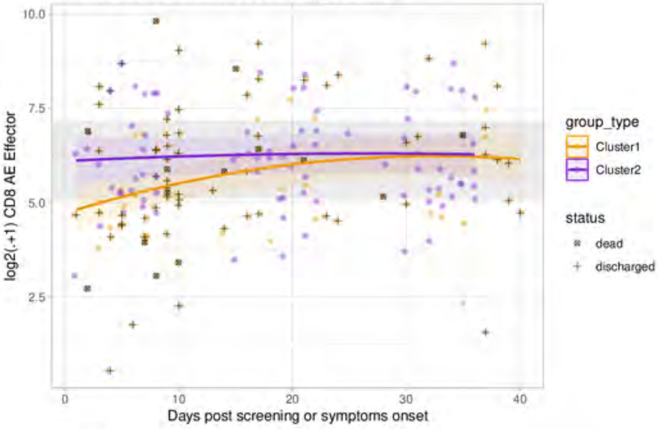

CD8 CM T

Likelihood ratio test: interaction p = 0.027, adj. p = 0.048 (*)

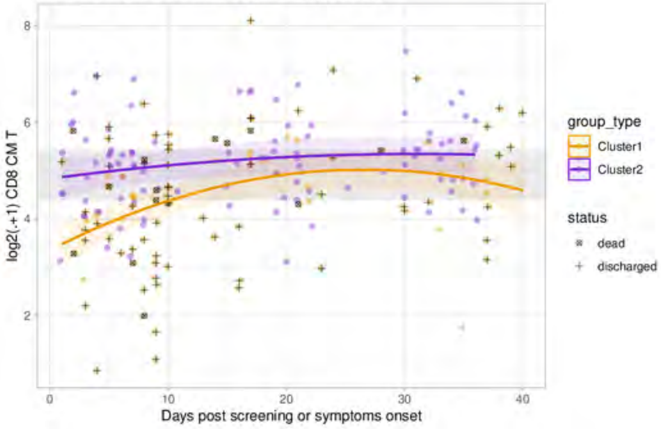

CD8 AE Tscm

Likelihood ratio test: interaction p = 0.086, adj. p = 0.12

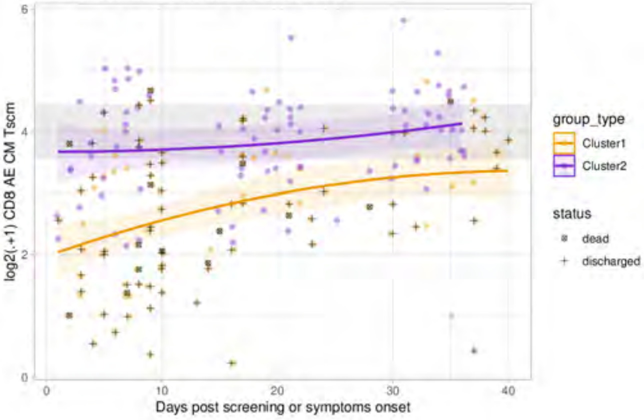

CD8 EM T

Likelihood ratio test: interaction p = 0.00015, adj. p = 0.0034 (**)

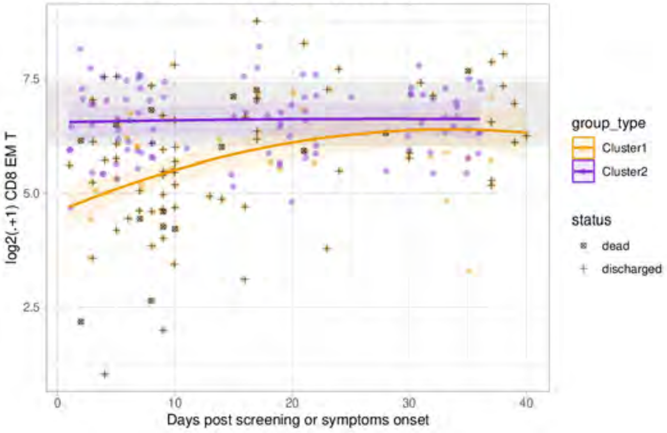

CD8 EMRA T

Likelihood ratio test: interaction p = 0.002, adj. p = 0.0076 (**)

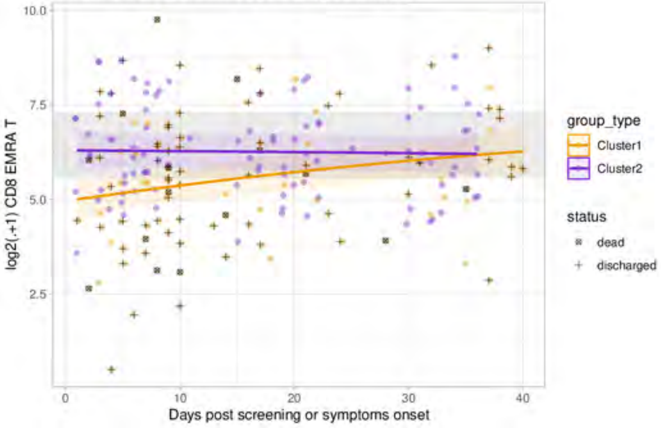

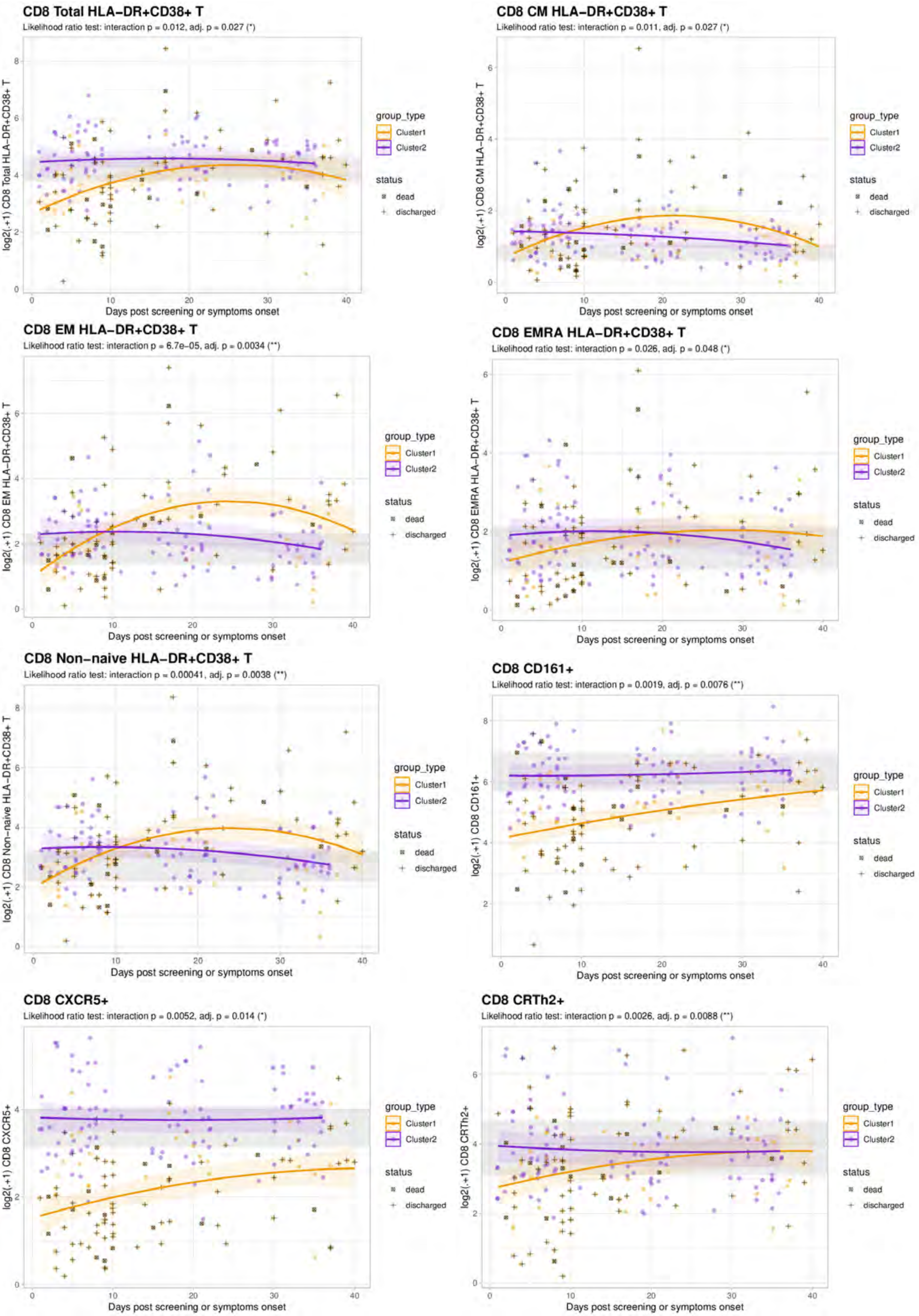
CD8 Total HLA−DR+CD38+ T
Likelihood ratio test: interaction p = 0.012, adj. p = 0.027 (*)
log2(.+1) CD8 Total HLA−DR+CD38+ T
Days post screening or symptoms onset
group_type
Cluster1
Cluster2
status
dead
discharged
CD8 CM HLA−DR+CD38+ T
Likelihood ratio test: interaction p = 0.011, adj. p = 0.027 (*)
log2(.+1) CD8 CM HLA−DR+CD38+ T
Days post screening or symptoms onset
group_type
Cluster1
Cluster2
status
dead
discharged
CD8 EM HLA−DR+CD38+ T
Likelihood ratio test: interaction p = 6.7e−05, adj. p = 0.0034 (**)
log2(.+1) CD8 EM HLA−DR+CD38+ T
Days post screening or symptoms onset
group_type
Cluster1
Cluster2
status
dead
discharged
CD8 EMRA HLA−DR+CD38+ T
Likelihood ratio test: interaction p = 0.026, adj. p = 0.048 (*)
log2(.+1) CD8 EMRA HLA−DR+CD38+ T
Days post screening or symptoms onset
group_type
Cluster1
Cluster2
status
dead
discharged
CD8 Non−naive HLA−DR+CD38+ T
Likelihood ratio test: interaction p = 0.00041, adj. p = 0.0038 (**)
log2(.+1) CD8 Non−naive HLA−DR+CD38+ T
Days post screening or symptoms onset
group_type
Cluster1
Cluster2
status
dead
discharged
CD8 CD161+
Likelihood ratio test: interaction p = 0.0019, adj. p = 0.0076 (**)
log2(.+1) CD8 CD161+
Days post screening or symptoms onset
group_type
Cluster1
Cluster2
status
dead
discharged
CD8 CXCR5+
Likelihood ratio test: interaction p = 0.0052, adj. p = 0.014 (*)
log2(.+1) CD8 CXCR5+
Days post screening or symptoms onset
group_type
Cluster1
Cluster2
status
dead
discharged
CD8 CRTh2+
Likelihood ratio test: interaction p = 0.0026, adj. p = 0.0088 (**)
log2(.+1) CD8 CRTh2+
Days post screening or symptoms onset
group_type
Cluster1
Cluster2
status
dead
discharged

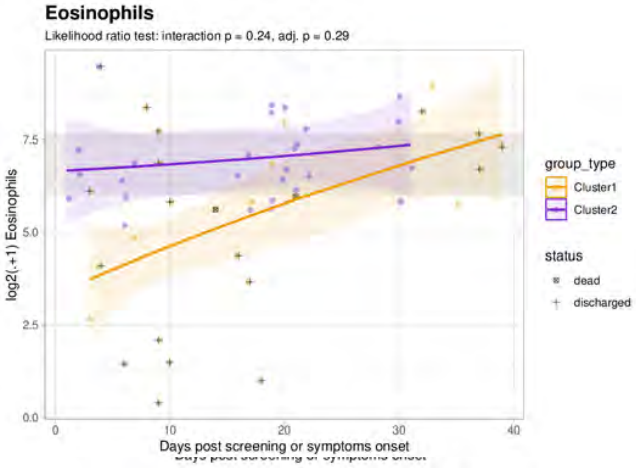
Eosinophils
Likelihood ratio test: interaction p = 0.24, adj. p = 0.29
log2(.+1) Eosinophils
Days post screening or symptoms onset
group_type
Cluster1
Cluster2
status
dead
discharged

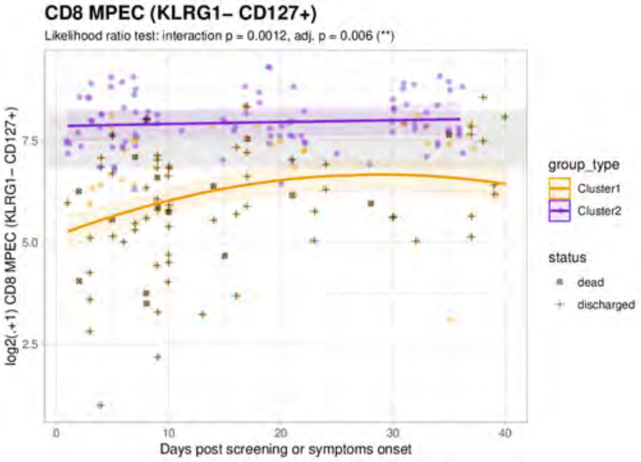
CD8 MPEC (KLRG1– CD127+)
Likelihood ratio test: interaction p = 0.0012, adj. p = 0.006 (**)
log2(.+1) CD8 MPEC (KLRG1– CD127+)
Days post screening or symptoms onset
group_type
Cluster1
Cluster2
status
dead
discharged

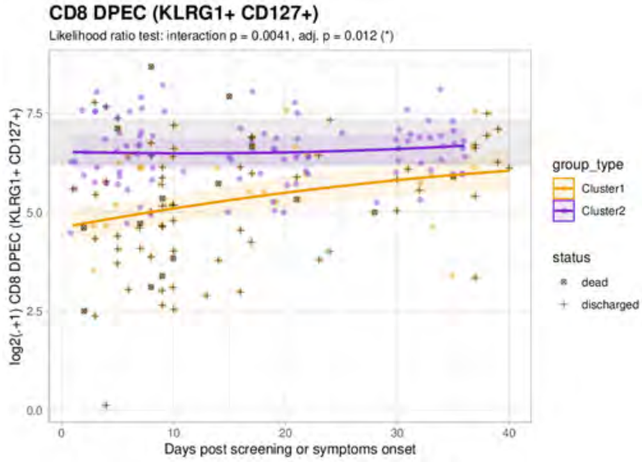
CD8 DPEC (KLRG1+ CD127+)
Likelihood ratio test: interaction p = 0.0041, adj. p = 0.012 (*)
log2(.+1) CD8 DPEC (KLRG1+ CD127+)
Days post screening or symptoms onset
group_type
Cluster1
Cluster2
status
dead
discharged

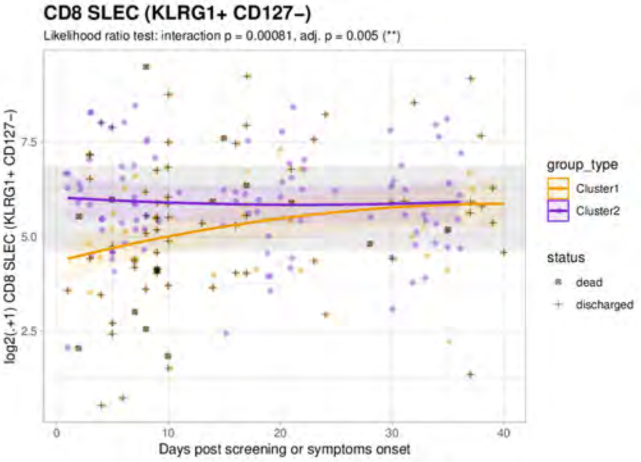
CD8 SLEC (KLRG1+ CD127–)
Likelihood ratio test: interaction p = 0.00081, adj. p = 0.005 (**)
log2(.+1) CD8 SLEC (KLRG1+ CD127–)
Days post screening or symptoms onset
group_type
Cluster1
Cluster2
status
dead
discharged

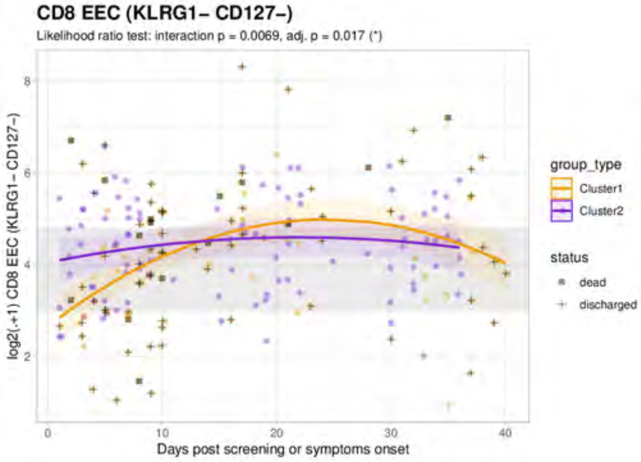
CD8 EEC (KLRG1– CD127–)
Likelihood ratio test: interaction p = 0.0069, adj. p = 0.017 (*)
log2(.+1) CD8 EEC (KLRG1– CD127–)
Days post screening or symptoms onset
group_type
Cluster1
Cluster2
status
dead
discharged

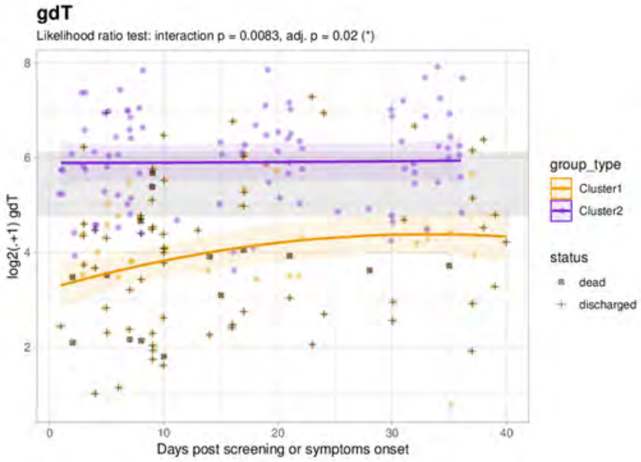
gdT
Likelihood ratio test: interaction p = 0.0083, adj. p = 0.02 (*)
log2(.+1) gdT
Days post screening or symptoms onset
group_type
Cluster1
Cluster2
status
dead
discharged

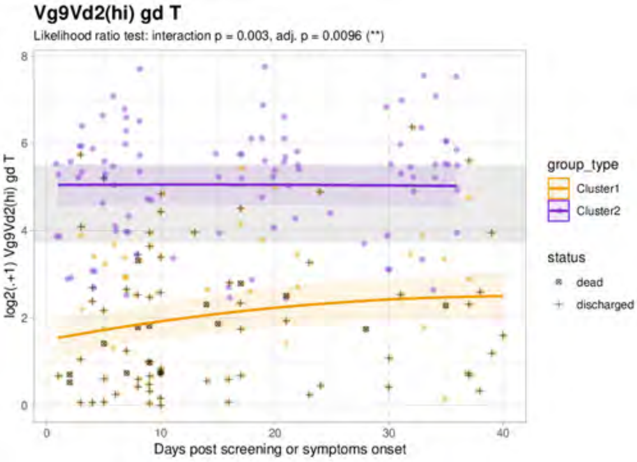
Vg9Vd2(hi) gd T
Likelihood ratio test: interaction p = 0.003, adj. p = 0.0096 (**)
log2(.+1) Vg9Vd2(hi) gd T
Days post screening or symptoms onset
group_type
Cluster1
Cluster2
status
dead
discharged

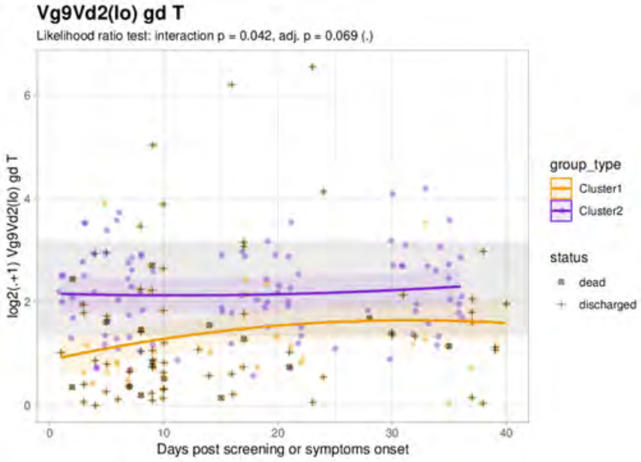
Vg9Vd2(lo) gd T
Likelihood ratio test: interaction p = 0.042, adj. p = 0.069 (.)
log2(.+1) Vg9Vd2(lo) gd T
Days post screening or symptoms onset
group_type
Cluster1
Cluster2
status
dead
discharged

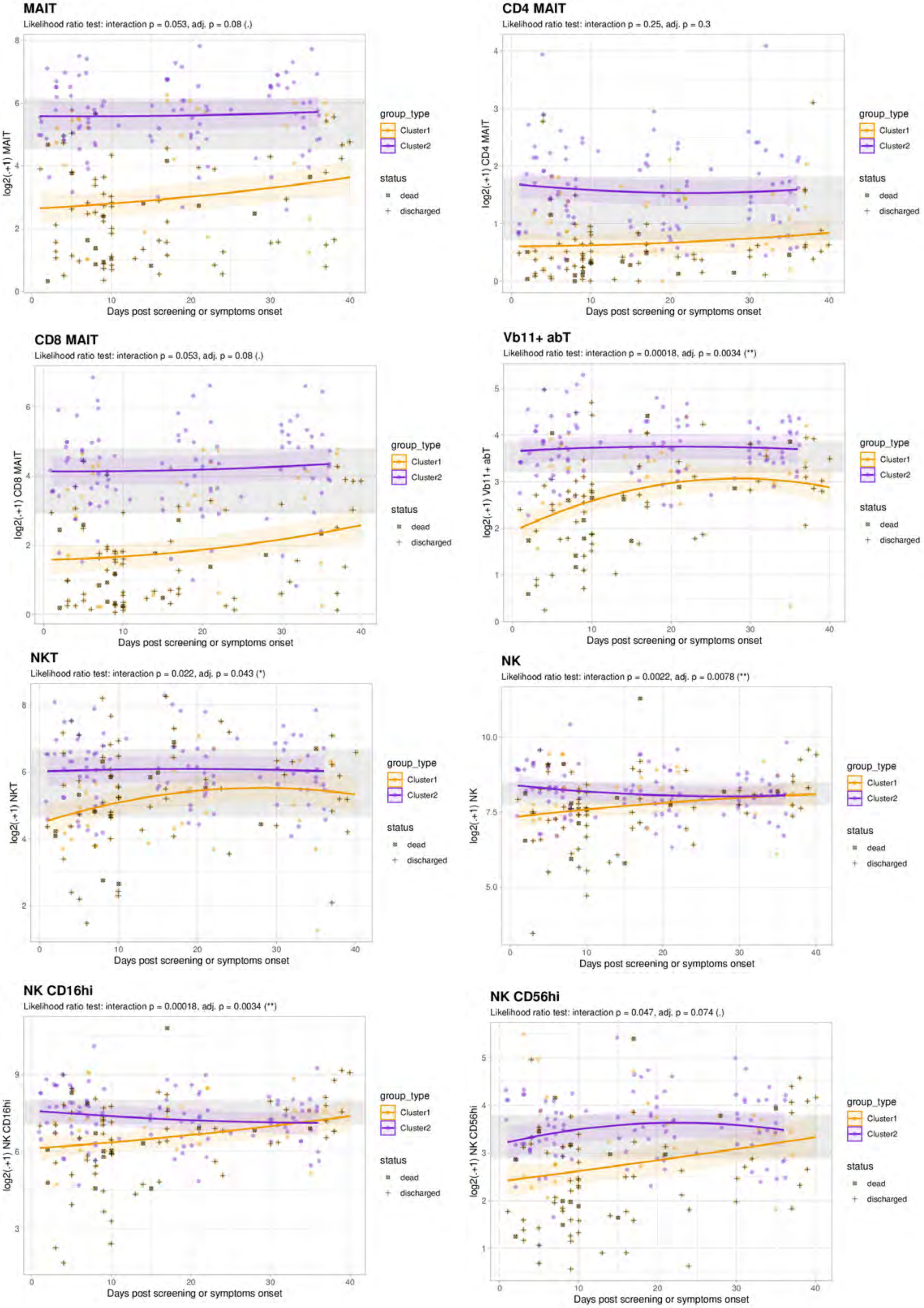
MAIT
Likelihood ratio test: interaction p = 0.053, adj. p = 0.08 (.)
log2(.+1) MAIT
CD4 MAIT
Likelihood ratio test: interaction p = 0.25, adj. p = 0.3
log2(.+1) CD4 MAIT
CD8 MAIT
Likelihood ratio test: interaction p = 0.053, adj. p = 0.08 (.)
log2(.+1) CD8 MAIT
Vb11+ abT
Likelihood ratio test: interaction p = 0.00018, adj. p = 0.0034 (**)
log2(.+1) Vb11+ abT
NKT
Likelihood ratio test: interaction p = 0.022, adj. p = 0.043 (*)
log2(.+1) NKT
NK
Likelihood ratio test: interaction p = 0.0022, adj. p = 0.0078 (**)
log2(.+1) NK
NK CD16hi
Likelihood ratio test: interaction p = 0.00018, adj. p = 0.0034 (**)
log2(.+1) NK CD16hi
NK CD56hi
Likelihood ratio test: interaction p = 0.047, adj. p = 0.074 (.)
log2(.+1) NK CD56hi
Days post screening or symptoms onset
group_type
Cluster1
Cluster2
status
dead
discharged

mDCs

Likelihood ratio test: interaction p = 7e−04, adj. p = 0.0049 (**)

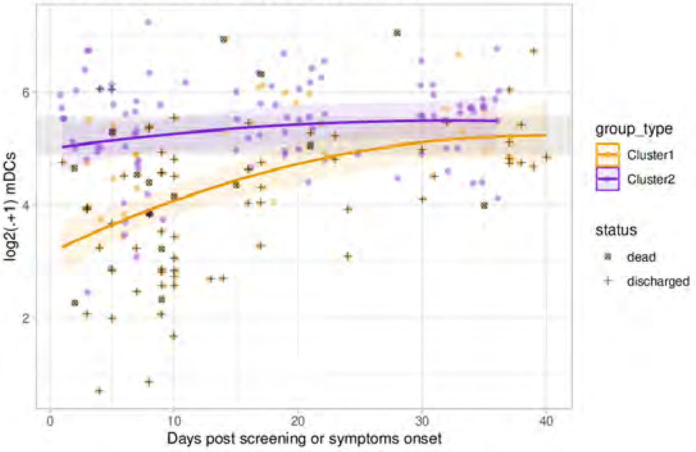

mDC CD141+

Likelihood ratio test: interaction p = 0.91, adj. p = 0.91

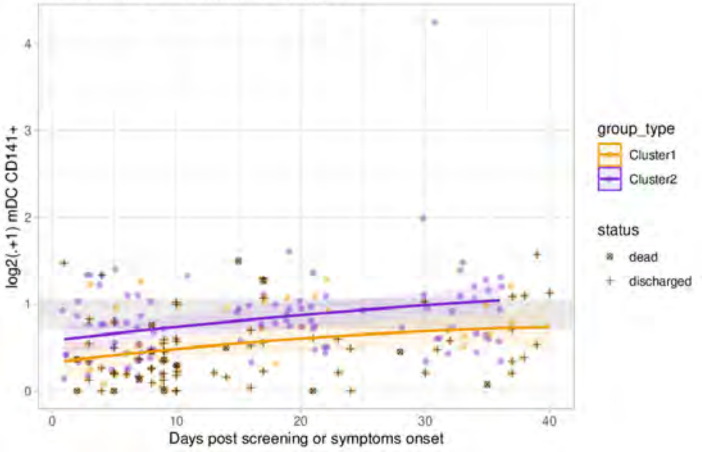

mDC CD1c+

Likelihood ratio test: interaction p = 0.0089, adj. p = 0.021 (*)

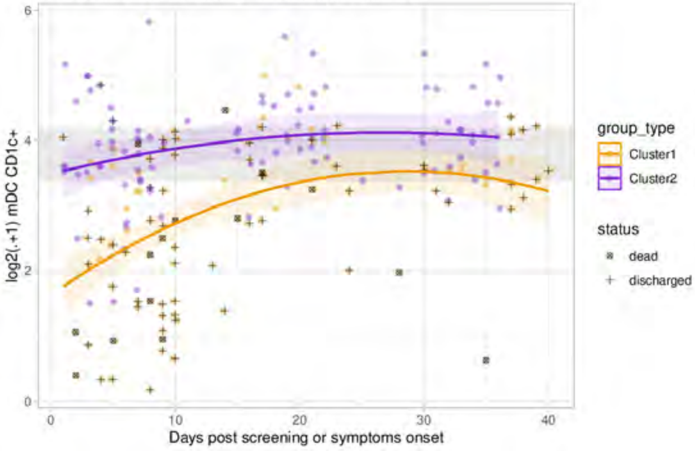

Classical mon.

Likelihood ratio test: interaction p = 0.074, adj. p = 0.11

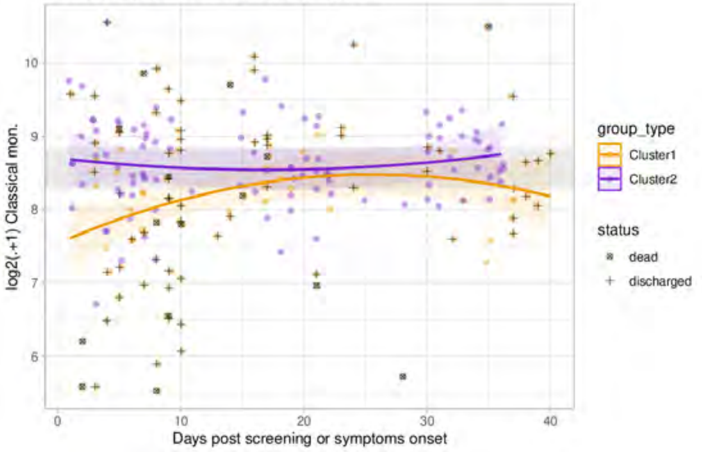

Intermediate mon.

Likelihood ratio test: interaction p = 0.41, adj. p = 0.44

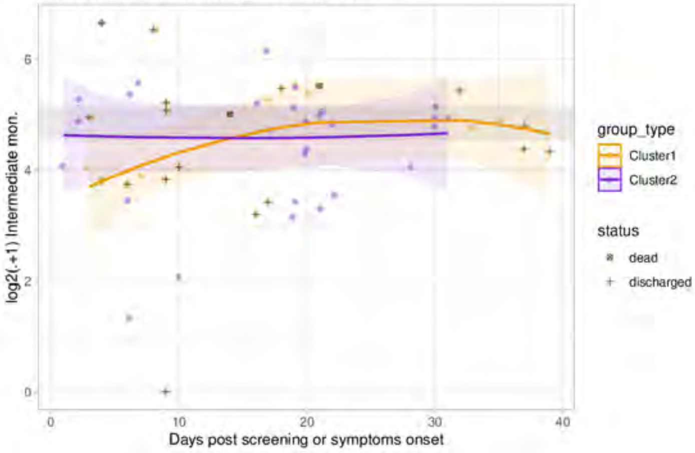

Non−Classical mon.

Likelihood ratio test: interaction p = 0.39, adj. p = 0.43

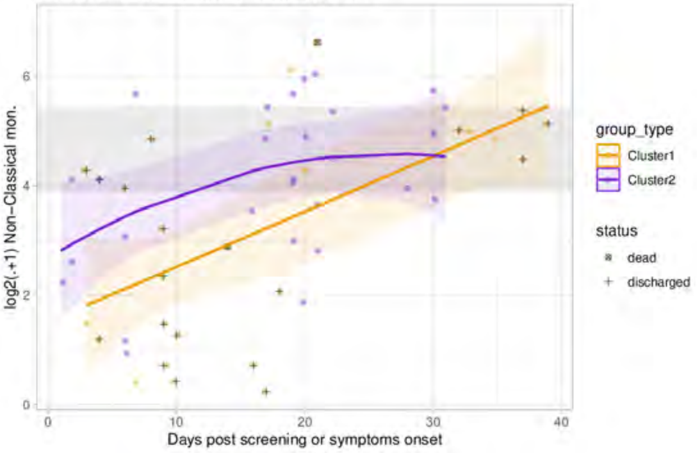

Neutrophils

Likelihood ratio test: interaction p = 0.36, adj. p = 0.41

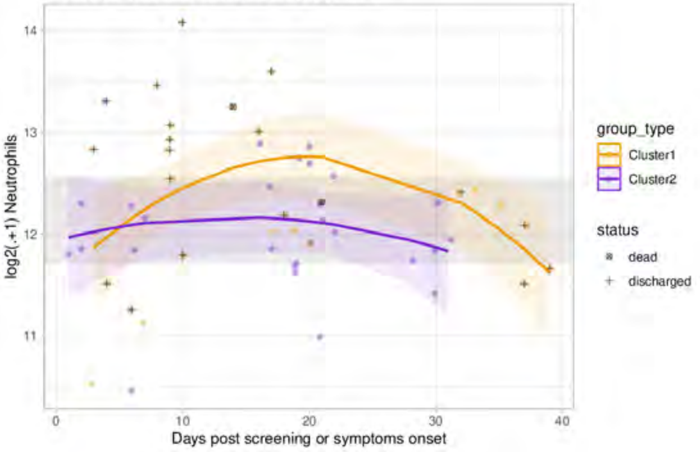

CD66b− Neutrophils

Likelihood ratio test: interaction p = 0.36, adj. p = 0.41

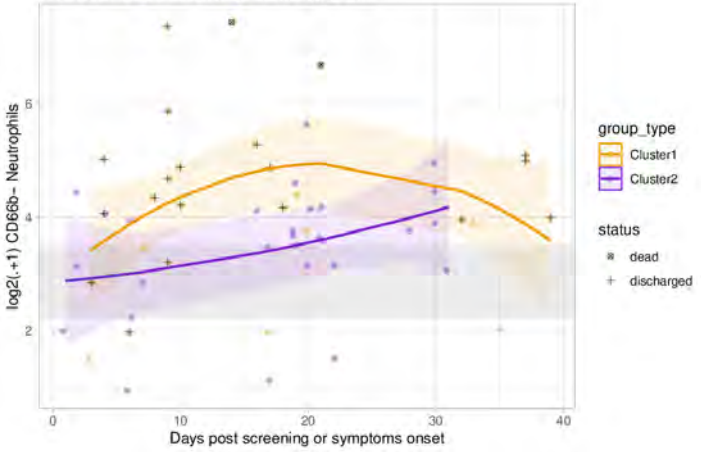

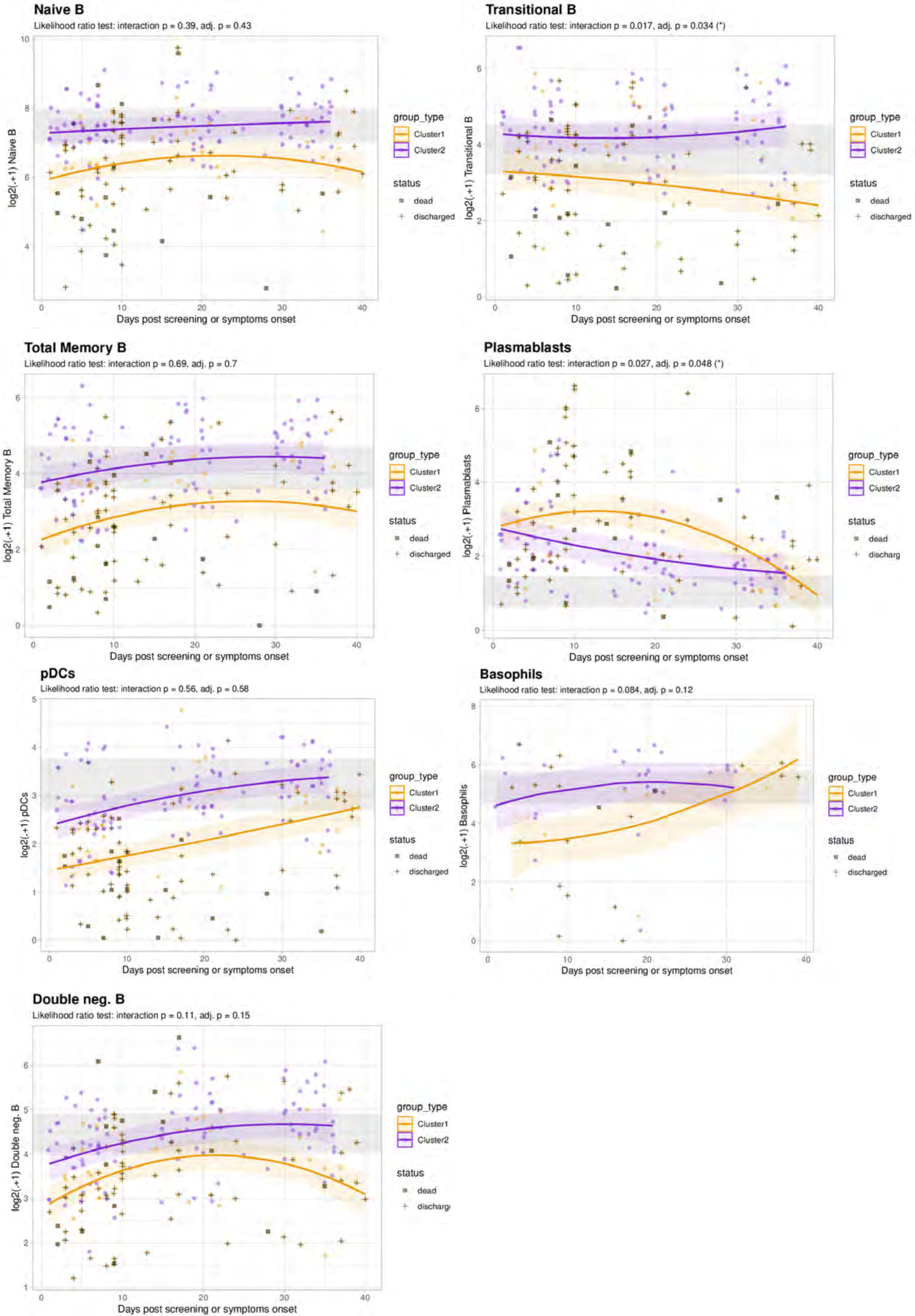
Naive B
Likelihood ratio test: interaction p = 0.39, adj. p = 0.43
log2(.+1) Naive B
Days post screening or symptoms onset
Transitional B
Likelihood ratio test: interaction p = 0.017, adj. p = 0.034 (*)
log2(.+1) Transitional B
Days post screening or symptoms onset
Total Memory B
Likelihood ratio test: interaction p = 0.69, adj. p = 0.7
log2(.+1) Total Memory B
Days post screening or symptoms onset
Plasmablasts
Likelihood ratio test: interaction p = 0.027, adj. p = 0.048 (*)
log2(.+1) Plasmablasts
Days post screening or symptoms onset
pDCs
Likelihood ratio test: interaction p = 0.56, adj. p = 0.58
log2(.+1) pDCs
Days post screening or symptoms onset
Basophils
Likelihood ratio test: interaction p = 0.084, adj. p = 0.12
log2(.+1) Basophils
Days post screening or symptoms onset
Double neg. B
Likelihood ratio test: interaction p = 0.11, adj. p = 0.15
log2(.+1) Double neg. B
Days post screening or symptoms onset
group_type
Cluster1
Cluster2
status
dead
discharged

Data S4: Flow cytometry gating strategy, related to STAR Methods

A) B cell, **B)** non-conventional T cell, **C)** DCs and monocyte, **D)** T regulatory cells, and **E)** conventional T cell panels.

A

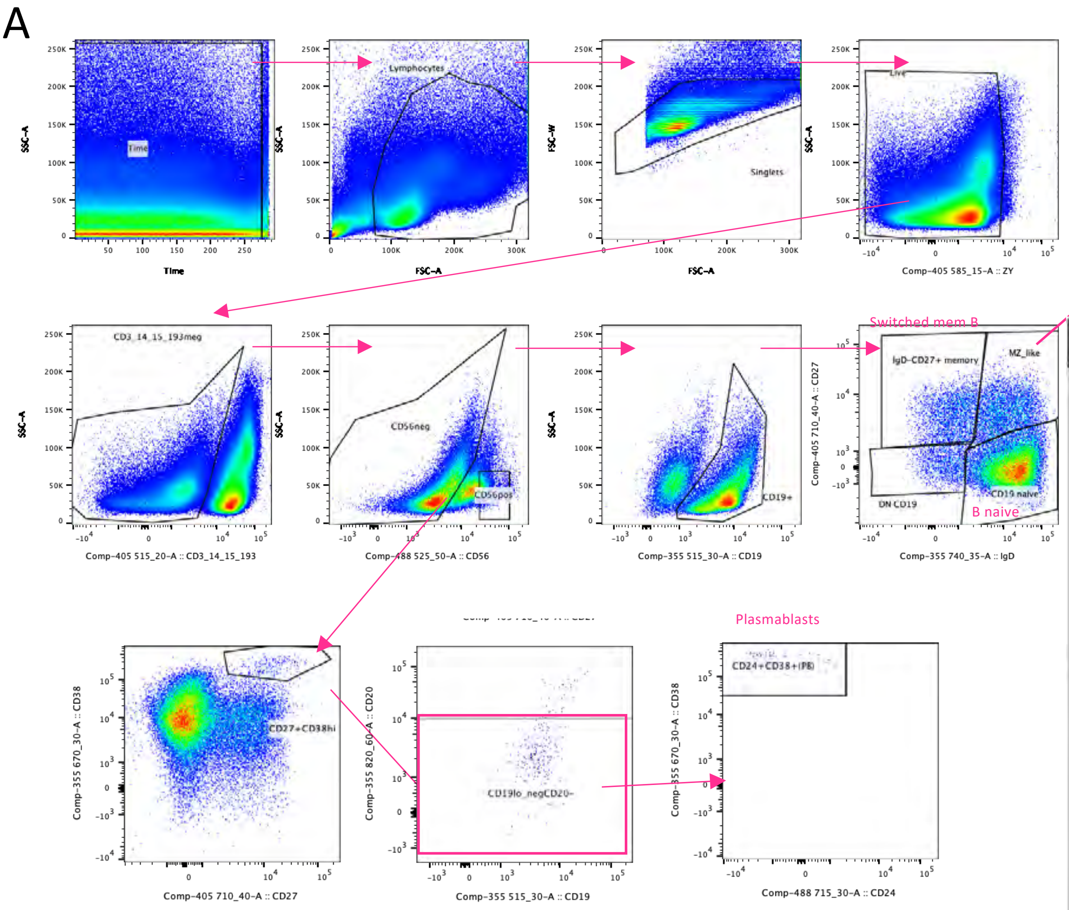

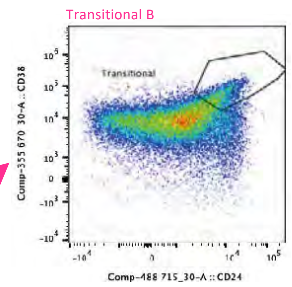

Panel	Laser	BPF	Antigen	Fluorophore
B cells	355	379/28		
		515/30	CD19	BUV496
		560/40		
		670/30	CD38	BUV661
		740/35		
		820/60	CD20	BUV805
	405	450/50	CD39	BV421
		515/20	CD3, CD14, CD15, CD193	BV510
		585/15	Zombie Yellow	
		605/40	HLADR	BV605
		670/30	CD71	BV650
		710/40	CD27	BV711
		741/40		
		780/60	CD73	BV785
	488	525/50	CD56	FITC
		610/20		
		685/35		
		715/30	CD24	BB700
		780/60		
	561	585/15		
		610/20		
		670/30	CD80	PECy5
		710/40		
		780/60	CD86	PECy7
	640	670/30	GLUT1	AF647
		730/45	CXCR5	APC-R700
		780/60		

B

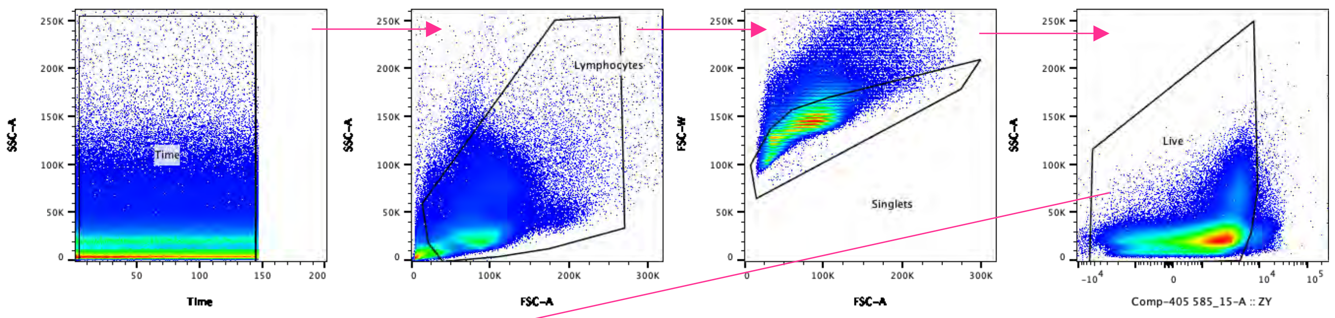

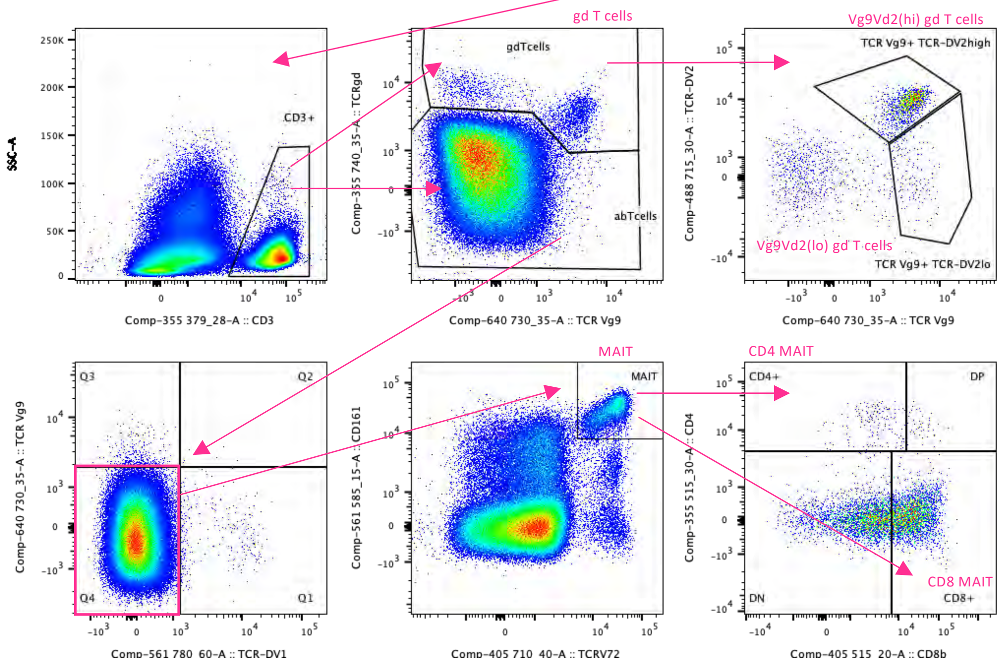

Panel	Laser	BPF	Antigen	Fluorophore
non-conventional T cells	355	379/28	CD3	BUV395
		515/30	CD4	BUV496
		560/40		
		670/30		
		740/35	TCRgd	BUV737
		820/60	CD28	BUV805
	405	450/50	PD-1	BV421
		515/20	CD8b	BV480
		585/15	Zombie Yellow	
		605/40	HLADR	BV605
		670/30	CD69	BV650
		710/40	TCRV7.2	BV711
		741/40		
		780/60		
	488	525/50	KLRG1	FITC
		610/20		
		685/35		
		715/30	TCR-DV2	PerCPCy5.5
		780/60		
	561	585/15	CD161	PE
		610/20		
		670/30	CD127	PECy5
		710/40		
		780/60	TCR-DV1	PECy7
	640	670/30	CCR5	AF647
		730/45	TCR Vγ9	AF700
		780/60	Vb11	APCVio770

C

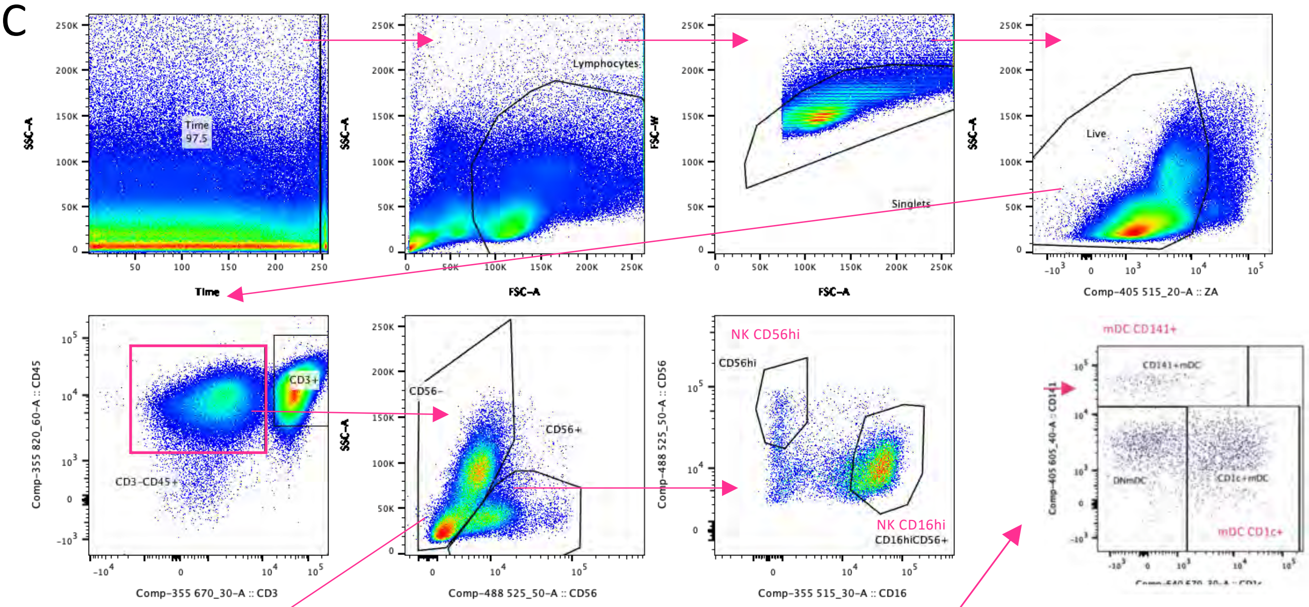

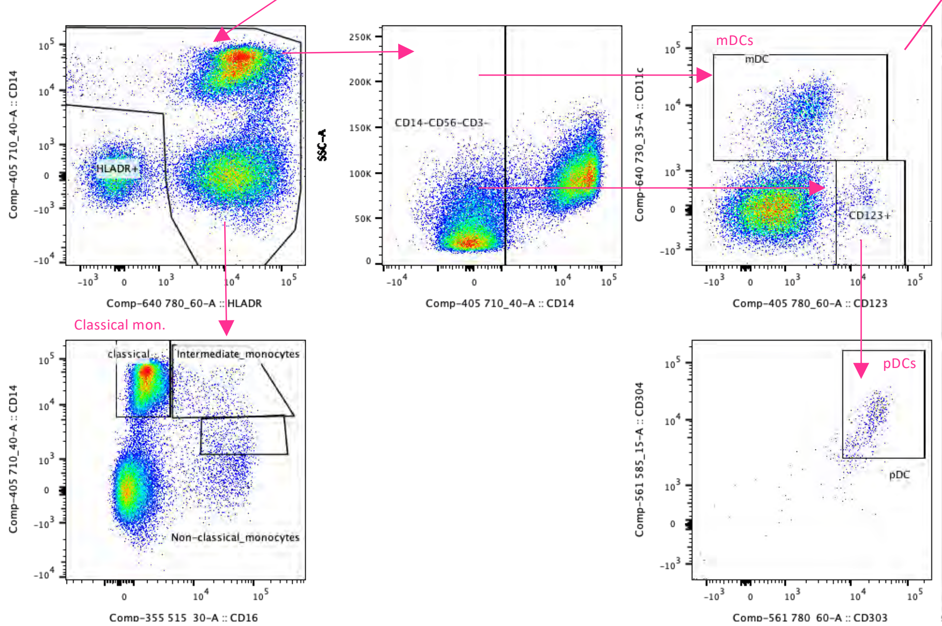

Panel	Laser	BPF	Antigen	Fluorophore
DC's and monocytes	355	379/28	CD40	BUV395
		515/30	CD16	BUV496
		560/40		
		670/30	CD3	BUV661
		740/35	CD86	BUV737
		820/60	CD45	BUV805
	405	450/50		
		515/20	Zombie Aqua	
		585/15	CD45RA	BV570
		605/40	CD141	BV605
		670/30	CCR7	BV650
		710/40	CD14	BV711
		741/40		
		780/60	CD123	BV786
	488	525/50	CD56	FITC
		610/20		
		685/35		
		715/30	CD32	BB700
		780/60		
	561	585/15	CD304	PE
		610/20	CD163	PE-CF594
		670/30	CD80	PECy5
		710/40		
		780/60	CD303	PE-Vio770
	640	670/30	CD1c	AF647
		730/45	CD11c	AF700
		780/60	HLA-DR	APC-H7

D

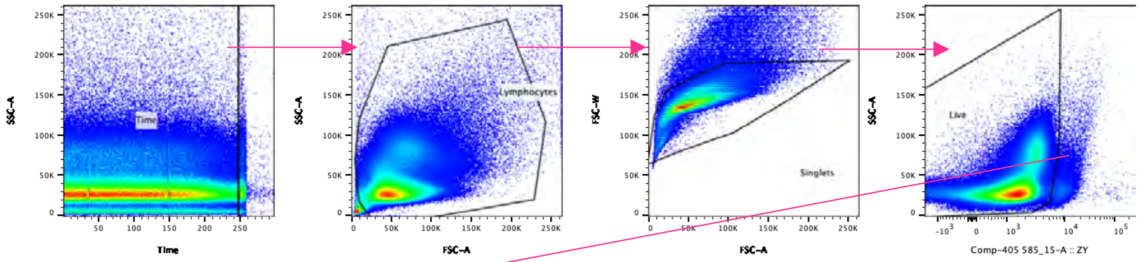

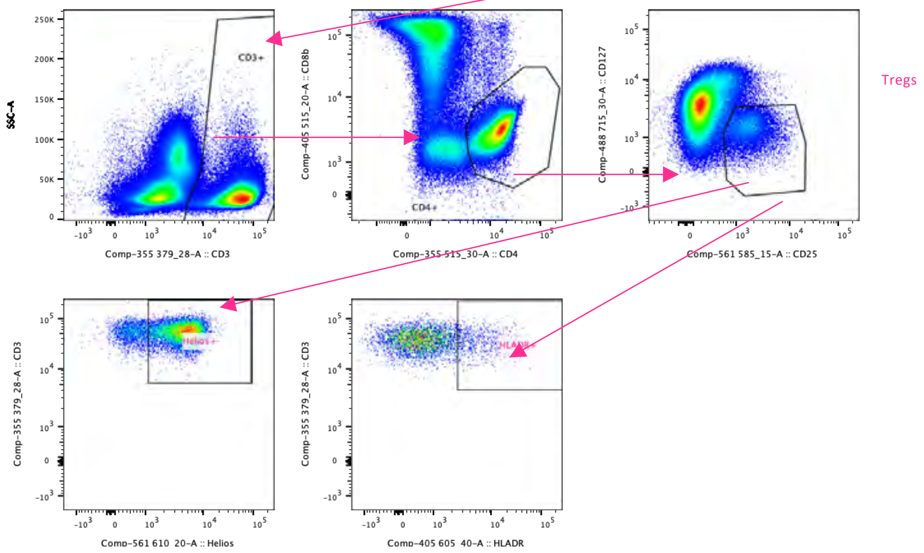

Panel	Laser	BPF	Antigen	Fluorophore
T regulatory cells	355	379/28	CD3	BUV395
		515/30	CD4	BUV496
		560/40		
		670/30		
		740/35		
		820/60	CD45RA	BUV805
	405	450/50	PD-1	BV421
		515/20	CD8b	BV480
		585/15	Zombie Yellow	
		605/40	HLADR	BV605
		670/30	CCR7	BV650
		710/40		
		741/40		
		780/60	CD73	Brilliant Violet 785™
	488	525/50		
		610/20		
		685/35		
		715/30	CD127	PerCP efluor710
		780/60		
	561	585/15	CD25	PE
		610/20	Helios	Pedazzle
		670/30		
		710/40		
		780/60	CCR4	PEVio770
	640	670/30	FoxP3	APC
		730/45	CXCR5	APC-R700
		780/60	CD39	APC-fire

E

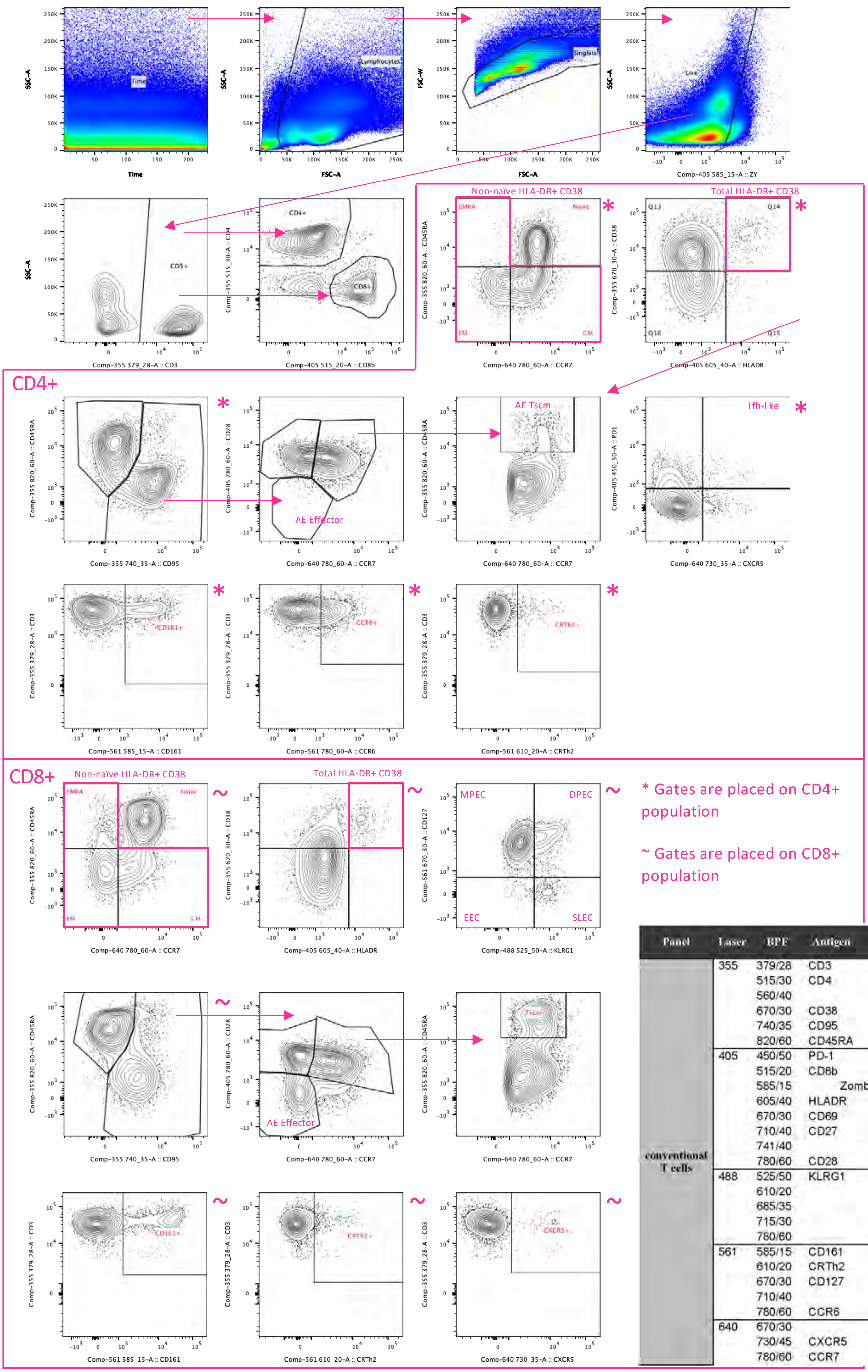

Panel	Laser	BPF	Antigen	Fluorophore
conventional T cells	355	379/28	CD3	BUV395
		515/30	CD4	BUV496
		560/40		
		670/30	CD38	BUV661
		740/35	CD95	BUV737
		820/60	CD45RA	BUV805
	405	450/50	PD-1	BV421
		515/20	CD8b	BV480
		585/15		Zombie Yellow
		605/40	HLADR	BV605
		670/30	CD69	BV650
		710/40	CD27	BV711
		741/40		
		780/60	CD28	BV785
	488	525/50	KLRG1	FITC
		610/20		
		685/35		
		715/30		
		780/60		
	561	585/15	CD161	PE
		610/20	CRTh2	PE-dazzle
		670/30	CD127	PECy5
		710/40		
		780/60	CCR6	PECy7
	640	670/30		
		730/45	CXCR5	APC-R700
		780/60	CCR7	APC-fire
